# Bis(4‐benzhydryl‐benzoxazol‐2‐yl)methane – from a Bulky NacNac Alternative to a Trianion in Alkali Metal Complexes

**DOI:** 10.1002/chem.202100616

**Published:** 2021-05-25

**Authors:** Johannes Kretsch, Anne‐Kathrin Kreyenschmidt, Timo Schillmöller, Märt Lõkov, Regine Herbst‐Irmer, Ivo Leito, Dietmar Stalke

**Affiliations:** ^1^ Institut für Anorganische Chemie Georg-August-Universität Göttingen Tammannstraße 4 37077 Göttingen Germany; ^2^ Institute of Chemistry University of Tartu Ravila 14a 50411 Tartu Estonia

**Keywords:** Ligand design, Metalation, Methanides, NacNac, Potassium, s-Block chemistry

## Abstract

A novel sterically demanding bis(4‐benzhydryl‐benzoxazol‐2‐yl)methane ligand **6** (^4−BzhH2^BoxCH_2_) was gained in a straightforward six‐step synthesis. Starting from this ligand monomeric [*M*(^4‐BzhH2^BoxCH)] (*M*=Na (**7**), K (**8_1_**)) and dimeric [{*M*(^4‐BzhH2^BoxCH)}_2_] (*M*=K (**8_2_**), Rb (**9**), Cs (**10**)) alkali metal complexes were synthesised by deprotonation. Abstraction of the potassium ion of **8** by reaction with 18‐crown‐6 resulted in the solvent separated ion pair [{(THF)_2_K@(18‐crown‐6)}{bis(4‐benzhydryl‐benzoxazol‐2‐yl)methanide}] (**11**), including the energetically favoured monoanionic (*E*,*E*)‐(^4‐BzhH2^BoxCH) ligand. Further reaction of ^4−BzhH2^BoxCH_2_ with three equivalents KH and two equivalents 18‐crown‐6 yielded polymeric [{(THF)_2_K@(18‐crown‐6)}{K@(18‐crown‐6)K(^4‐Bzh^BoxCH)}]_*n*_ (*n*→∞) (**12**) containing a trianionic ligand. The neutral ligand and herein reported alkali complexes were characterised by single X‐ray analyses identifying the latter as a promising precursor for low‐valent main group complexes.

## Graphical abstract

**Bis(4‐benzhydryl‐benzoxazol‐2‐yl)methane (^4−BzhH2^BoxCH_2_)** provides a versatile platform for monomeric ligands in [*M*(^4‐BzhH2^BoxCH)] (*M*=Na, K) and dimeric [{*M*(^4‐BzhH2^BoxCH)}_2_] (*M*=K, Rb, Cs) alkali metal complexes. Even more remarkable the reaction of ^4−BzhH2^BoxCH_2_ with three equivalents KH and two equivalents 18‐crown‐6 yields polymeric [{(THF)_2_K@(18‐crown‐6)}{K@(18‐crown‐6)K(^4‐BzhH2^BoxCH)}]_*n*_ (*n*→∞), containing a trianionic ligand. While the monoanions only provide *N*,*N*‐chelation like NacNac the latter trianion potentially provides *C*,*N*,*N*,*C*‐chelation and should be another promising ligand platform.

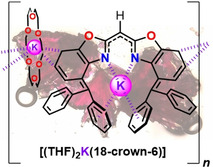

## Introduction

**Scheme 1:**
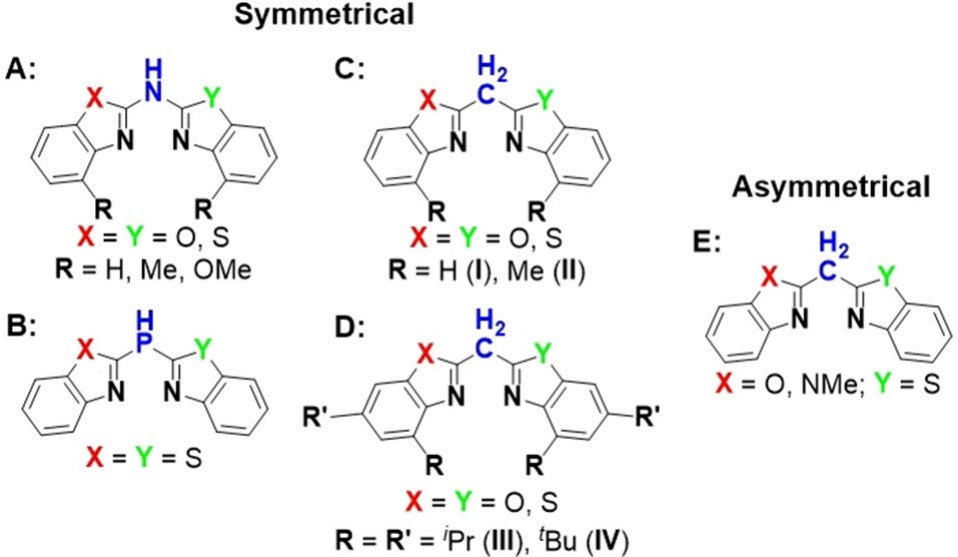
Symmetrical bis(heterocyclo)amine (**A**), ‐phosphane (**B**), and ‐methane (**C**+**D**) ligands. Bis(benzoxazol‐2‐yl)methanes **C** (**I**, **II**) are (un)substituted in ortho‐position to imine group whereas **D** is ^*i*^Pr (**III**) or ^*t*^Bu (**IV**) substituted in ortho‐ and para‐position. Asymmetrical and unsubstituted bis(heterocylo)methanes (**E**).

Over the last decades, monoanionic bidentate *N*,*N’*‐ligands like β‐diketiminates^[1]^ or amidinates^[2]^ gained interest due to their relative ease of synthesis and versatility with regards to steric and electronic properties. Based on these ligands a great variety of interesting transition metal,[^1b^, ^3^] main group^[4]^ and lanthanide^[5]^ compounds were synthesised in the last two decades. For example, in the field of main group chemistry, catalytically active β‐diketiminate‐derived group 13 metal complexes were synthesised.^[6]^ Further remarkable results were the synthesis of low‐oxidation‐state magnesium(I)^[7]^ and aluminium(I)^[8]^ complexes, which were facilitated by bulky β‐diketiminate ligands e. g. ^Dipp^NacNac (Dipp=2,6‐diisopropyl‐phenyl). These findings initiated research into other ligand platforms, which mimic the coordination abilities of the ubiquitous NacNac ligand, in particular the *κ* ^2^‐*N*,*N*‐coordination of the two imine nitrogen atoms to a metal ion to form six‐membered metallaheterocycles. Starting from the bis(2‐pyridyl)methane ligand and its isoelectronically N,^[9]^ P^[10]^ and As^[11]^ bridged derivatives, the 2‐pyridyl moieties were exchanged for benzannulated oxazoline or thiazole sidearms.^[12]^ Due to this variation of the bridging backbone and therefore connected residues, multiple symmetrical and asymmetrical ligand platforms were developed (Scheme 1).^[13]^ Although additional chalcogene donor sites (O or S) as well as an optional donating bridging unit (N, P or CH) are available for coordination, in most cases, the oxygen or sulfur do not share the same good Lewis‐donor abilities of the ring nitrogen atoms. Hence a six‐membered NacNac‐like coordination motif (C_3_N_2_ *M*, *M*=cationic metal fragment) is maintained. In recent years the focus has been on symmetrical bis(benzoxazole‐2‐yl)methanides **I** to **IV** in Scheme 1 that can be straightforwardly synthesised by cyclocondensation reaction of two equivalents of corresponding 2‐aminophenol derivatives and one equivalent of the C_3_‐linker unit ethylbisimidate dihydrochloride derived from malonic acid.^[14]^ In general, the synthesised bis(benzoxal‐2‐yl)methanes are utilised as neutral or as monoanionic ligands.[^12a^, ^15^] Deprotonation is accomplished with a rather strong Lewis base e. g. *n*‐butyllithium or potassium hydride.^[16]^ Furthermore, these alkali metal complexes can be used as precursors in salt elimination reactions for metal complexes e. g. [*X*_2_Al(**I^−^**)]^[17]^ (*X*=Cl, I). Despite of the steric demand provided by ^*i*^Pr‐ or ^*t*^Bu‐residues at C4‐position close to the coordination pocket in **III** or **IV**, mimicking the bulkiness provided by NacNac ligands, low‐oxidation or low‐valent Group 2 or 13 elements could so far not be synthesised. Therefore, we decided to introduce bulkier benzhydryl groups at the ligand scaffold inspired by compounds containing 2,6‐dibenzhydrylphenyl residues e. g. [:*M*H(*L*)]^[18]^ (*M*=Ge or Sn, (*L*)=‐N(*Ar*)(Si^*i*^Pr_3_), *Ar*=4‐^*i*^Pr‐C_6_H_2_Bzh_2_) or [M(*Ar*N)_2_CN=C^*t*^Bu_2_]^[19]^ (*Ar*=4‐^*t*^Bu‐C_6_H_2_Bzh_2_). In the following paragraph, the six‐step synthesis of bis(4‐benzhydryl‐benzoxazol‐2‐yl)methane (**6**, ^4‐BzhH2^BoxCH_2_) and its behaviour in alkali metal coordination are discussed.

## Results and Discussion

### Ligand synthesis and characterisation

**Figure 1:**
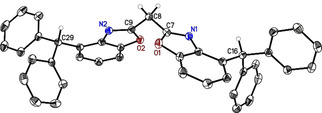
Molecular structure of bis(4‐benzhydryl‐benzoxazol‐2‐yl)methane (**6**, ^4‐BzhH2^BoxCH_2_). Anisotropic displacement parameters are depicted at the 50 % probability level. All hydrogen atoms are omitted for clarity except for the bridging (methylene) and benzylic ones. Selected bond lengths [Å] and bond angles [°]: N1−C7 1.289(2), N2−C9 1.291(2), C7−C8 1.491(2), C8−C9 1.497(2), C7−C8−C9 115.71(14), N1/2−C7−C8−C9=103.5(2)°/−26.38(9)°; O1/2−C7−C8−C9=31.47(10)°/−71.05(19)°.

To obtain the sterically demanding bis(4‐benzhydryl‐benzoxazol‐2‐yl)methane (**6**, ^4‐BzhH2^BoxCH_2_), 2‐amino‐3‐benzhydrylphenol was first synthesised according to the synthetic protocols of Quaranta *et al*.^[20]^ that were slightly modified (for details of intermediates **1**–**5** see Supporting Information). Thereafter, two equivalents of 2‐amino‐3‐benzhydrylphenol and one equivalent of ethyl cyanoacetimidate hydrochloride C_3_‐linker unit were reacted by cyclocondensation reaction.[^14^, ^16a^, ^16b^, ^21^] For this purpose, starting materials were heated in MeOH under reflux (85 °C) for at least 3 d. Ligand **6** and side product 2‐(4‐benzhydrylbenzoxazol‐2‐yl) acetamide (**6 a**) precipitated and were separated by filtration. Further work‐up (see Supporting Information) and purification by column chromatography on silica gel (THF/hexane 3 : 2; *R*_f_ (**6**)=0.9; *R*_f_ (**6 a**)=0.48) led to ^4‐BzhH2^BoxCH_2_ (**6**) in decent yields (YLD(**6**): 36 %; overall YLD: 13 % ; YLD(**6 a**): 13 %). All starting materials and ^4‐BzhH2^BoxCH_2_ ligand (**6**) were analysed by NMR spectroscopy, mass spectrometry, and elemental analysis. Crystals of **6** suitable for single XRD experiments were grown out of a saturated ethyl acetate solution by slow cooling or by vapor diffusion of pentane. It crystallises in the monoclinic space group *P*2_1_/*c* with one molecule in the asymmetric unit (Figure 1). The bridging carbon atom C8 is coordinated like other symmetrically substituted bisheterocyclo methanes[^12a^, ^21^, ^22^] in a distorted tetrahedral fashion with a slightly increased C7‐C8‐C9 angle of 115.71(14)° (related ligands: **II**=110.79(12)° to **I**=111.23(9)°).[^12a^, ^21^] Furthermore, the two benzoxazole moieties are less twisted 63.25(4)° relative to each other compared to related unsubstituted **I**=89.34(13)°^[12a]^ and 4‐methyl‐substituted **II**=81.14(4)°^[21]^ which exhibit an almost orthogonal alignment of the heteroaromatic planes.[^12a^, ^21^] This is attributed to the bulkier benzhydryl groups. These groups cause wider torsion angles between the C_3_‐linker unit and the nitrogen or oxygen atoms (N1/2‐C7‐C8‐C9=103.5(2)°/ −26.38(9)°; O1/2‐C7‐C8‐C9=31.47(10)°/−71.05(19)°), more similar to 4,6‐^*t*^butyl‐substituted **III** (twist=76.50(7)°, N1/N2‐C17‐C1‐C2=−22.06(9)°/133.20(18)°, O1/O2‐C17‐C1‐C2=23.05(10)°/−46.69(19)°).^[16b]^ Moreover, the solid‐state structure of ^4‐BzhH2^BoxCH_2_ (6) reveals intermolecular hydrogen bonds, in which one of the acidic methylene hydrogen atom interacts with the imine nitrogen atom (N1⋅⋅⋅H8’A 2.49 Å) of an adjacent molecule and vice versa. In contrast, single crystal structure determination of ligand **II** (N1⋅⋅⋅H8’A 2.39 Å, N1⋅⋅⋅H8’’B 2.63 Å) and **III** (N1⋅⋅⋅H8’A 2.58 Å, N1⋅⋅⋅H8’’B 2.64 Å) exhibit intermolecular C−H⋅⋅⋅N hydrogen bonds of both methylene hydrogen atoms. In addition, an interaction of the less acidic benzylic hydrogen atoms (p*K*_a_=33)^[23]^ in **6** with the carbon atom of the benzoxazol‐2‐yl (C9) unit of a neighbouring molecule is observed (C9⋅⋅⋅H16’ 2.81 Å). The acidity of the methylene protons correlates with the substitution or rather the electronic properties of related benzoxazole moieties.^[13]^ UV/Vis spectrophotometric titration experiments^[24]^ in acetonitrile unveiled a p*K*_a_ value of 26.61(6), supporting that ^4‐BzhH2^BoxCH_2_ is a somewhat stronger acid than **I** (26.89(3)), **II** (27.59(3)) and **III** (28.85(3)) but slightly weaker than bis(benzo‐thiazol‐2‐yl)methane (26.14(3)).^[13]^

### Alkali metal complexes of ^4‐BzhH2^BoxCH_2_

**Scheme 2:**
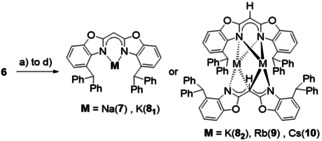
Synthesis of monomeric complexes **7** to **10** were carried out in toluene and ambient temperature under the following conditions (yield=YLD): a) 1.04 eq. Na, 5 d, YLD: 98 %, b) 1.15 eq. KH, 1 d, YLD: 89 %, c) 1.10 eq. Rb, 1 d, YLD: 76 %, d) 1.10 eq. Cs, 6 h, YLD: 74 %.

**Figure 2:**
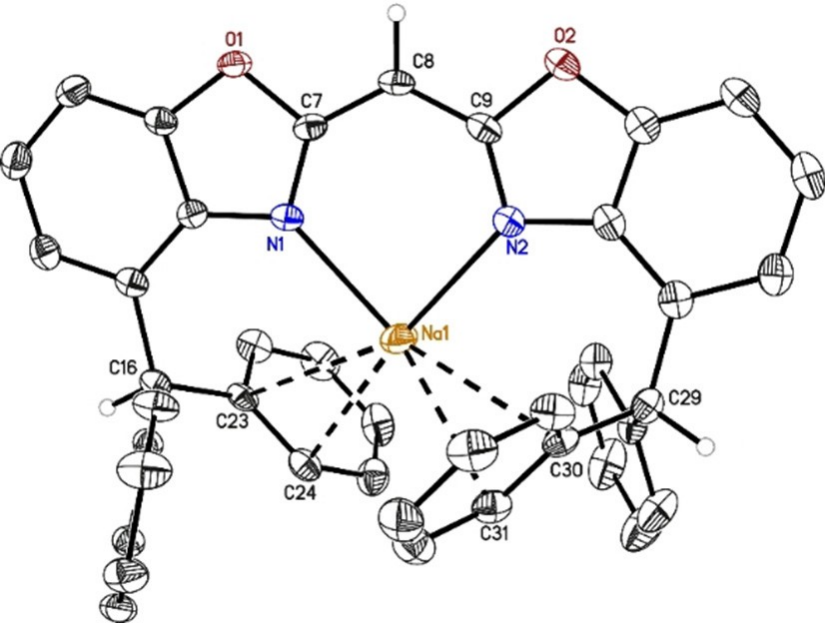
Molecular structure of {bis(4‐benzhydryl‐benzoxazol‐2‐yl)methanide} sodium (**7**, [Na(^4‐BzhH2^BoxCH)]). Anisotropic displacement parameters are depicted at the 50 % probability level. All hydrogen atoms are omitted for clarity except for the bridging (methylene) and benzylic ones.

^4‐BzhH2^BoxCH_2_ (**6**) was reacted with alkali metal‐based deprotonation or reduction agents to better understand its properties and establish an appropriate precursor for salt elimination reactions. All reactions were carried out in toluene at ambient temperature, whereas reaction times and input equivalents of alkali metal (hydrides) were adjusted to the reaction requirements (Scheme 2). In the case of bis(4‐benzhydryl‐benzoxazol‐2‐yl)methanide} sodium ([Na(^4‐BzhH2^BoxCH)]) (**7**), starting material **6** and neat sodium metal were vigorously stirred until all metal was consumed (5 d). After all volatiles had been removed under reduced pressure, **7** was isolated as a white powder in excellent yields (98 %). Crystals suitable for single crystal XRD experiments were grown from a saturated toluene solution at −28 °C. Donor‐free **7** crystallises in the monoclinic space group *P*2_1_/*n* with one molecule in the asymmetric unit (Figure 2). The solid‐state structure of monomeric [Na(^4‐BzhH2^BoxCH)] (**7**) exhibits a distorted *κ* ^2^‐*N*,*N*’‐coordination of the sodium ion by the imine ring nitrogen atoms. A negligible dislocation of the cation from the C_3_N_2_‐plane of 0.117(2) Å and a minor butterfly folding angle of 4.82(6)° between the two benzoxazol‐2‐yl moieties is observed (Table 1). Measured nitrogen sodium bond lengths of **7** (Na1−N1 2.3392(16) Å; Na1−N2 2.3456(15) Å) are similar to the Na1−N1 2.358(6) Å bond of the six‐membered sodium β‐diketiminate complex [(THF)_2_NaL]^[25]^ (L={N(SiMe_3_)C(Ph)}_2_CH) but are slightly shorter than neutral five‐membered TMEDA based [[{Na{N(SiMe_3_)(Dipp)}(TMEDA)}_2_] (Na1−N2 2.4726(17) Å; Na1−N3 2.461(2) Å)^[26]^ or four‐membered guadinate complex [[Na{(^*i*^PrNC(N*Ar*)(HN^*i*^Pr)}(THF)]_2_ (*Ar*=2,6‐Me_2_C_6_H_3_) (Na1−N1 2.453(1) Å, Na1−N2 2.558(2) Å).^[27]^ To complete the coordination sphere around Na^+^ ion *π*‐arene, interactions are formed to one phenyl group of the benzhydryl groups each.^[28]^ This way the benzyl hydrogen atoms are facing away from the metal. The Na⋅⋅⋅C_*Ar*_ distances ranging from 2.8305(18) (C_*ortho*_) to 3.0414(18) Å (C_*ipso*_) indicate a dihaptic arene coordination.^[29]^ ^1^H NMR spectra of **6** and deprotonated species **7** show a significant deshielding in toluene and simultaneous downfield shift of the methanide bridge from *δ*(−*H*_2_C−)=3.85 ppm to *δ*(−*H*C−)=5.38 ppm whereas benzylic protons experience a significant upfield shift from *δ*(−*H*CPh_2_)=6.41 ppm to 5.27 ppm. Furthermore, arene protons (Ph and NCOC_6_H_3_) are slightly upfield shifted in sodium complex **7** compared to ligand **6**. Elemental analysis and mass spectrometry (LIFDI, toluene) confirmed the synthesis of bulk [Na(^4‐BzhH2^BoxCH)] (**7**).

**Table 1:** Selected bond lengths, distances, and angles of complex **7** to **10**.

	(*Z*,*Z*)‐(^4‐BzhH2^BoxCH)					(*E*,*E*)‐(^4‐BzhH2^BoxCH)		
	**7**	**8_1_**	**8_2_**	**9**	**10**		**8_2_**	**9**	**10**
M−N [Å]	2.3392(16),	2.7768(18),	2.812(2) to	2.924(5) to	3.050(3) to	M−N [Å]	2.775(2),	2.905(5),	3.033(3),
	2.3456(15)	2.6982(18)	3.015(2)	3.063(4)	3.285(3)		2.812(2)	2.948(5)	3.202(3)
N−M−N [°]	86.53(5)	70.73(5)	67.57(7),	65.15(13),	62.76(8) to	M⋅⋅⋅C_3_N_2_ [Å]	3.170(3) to	3.297(6) to	2.603(10) to
			64.07(6)	63.34(12)	63.52(8)		3.493(3)	3.633(6)	1.252(19)
M⋅⋅⋅C_3_N_2_ [Å]	0.117(2)	1.571(3)	1.877(4)	2.036(7),	2.302(5) to	M⋅⋅⋅HC_2_N [Å]	2.18(3),	2.196(9),	2.60(5) to
			2.282(3)	2.303(7)	2.430(5)		0.85(5)	0.699(13)	1.16(8)
Folding	4.82(6)	5.54(8)	23.22(9)	23.63(19)	20.65(14),	Folding	16.75(8)	15.55(14)	17.85(11),
angle [°]					25.24(13)	angle [°]			15.22(10)

**Figure 3:**
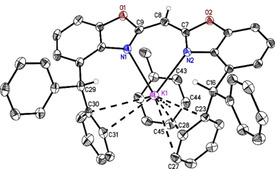
Molecular structure of monomeric {bis(4‐benzhydryl‐benzoxazol‐2‐yl)methanide} potassium (**8_1_**, [K(^4‐BzhH2^BoxCH)]). Anisotropic displacement parameters are depicted at the 50 % probability level. All hydrogen atoms are omitted for clarity except for the bridging (methylene) and benzylic ones.

The reaction of ligand **6** with potassium hydride (Scheme 2) resulted in two different modifications of [K(^4‐BzhH2^BoxCH)]_*n*_ with *n*=1 (**8_1_**) and 2 (**8_2_**), (YLD: 89 %). Colourless crystals were grown from a saturated toluene solution at −30 °C after 1 d. Single crystal XRD experiments revealed a monomeric as well as a dimeric species in the solid state. Monomeric [(toluene)K(^4‐BzhH2^BoxCH)] crystallises in the triclinic space group *P* $\bar{1}$ with one molecule and half a toluene molecule in the asymmetric unit (Figure 3). Compound **8_1_** consists of a distorted *κ* ^2^‐*N*,*N*’‐coordinated potassium cation, which features additional weak K⋅⋅⋅*π*‐arene interactions to one toluene molecule and one phenyl group of the benzhydryl moieties, respectively. In comparison with the sodium complex **6**, a fairly prominent deviation of the cation from the C_3_N_2_ plane of 1.571(3) Å is present. Due to the larger radius of potassium, longer K−N (K1−N1 2.7768(18) Å; K1−N2 2.6982(18) Å) and a less acute N1−K1−N2 of 70.73(5)° are observed. This is accompanied by an increasing butterfly folding angle to 5.54(8)°. Furthermore, potassium phenyl carbon distances suggest that the cation is *η* ^2^‐(K1−C30 3.467(2) Å, K1−C31 3.224(2) Å) and *η* ^3^‐coordinated (K1−C23 3.314(2) Å, K1−C27 3.503(2) Å, K1−C28 3.236(2) Å) by one phenyl residue of each benzhydryl group (A search in the CCDC version 5.41 (Aug. 2020)^[30]^ for structures with K and phenyl groups excluding *η* ^6^‐coordinated ones provided a range of short contacts between K and C_*ortho*_ of 2.77 to 3.76 Å).[^29c^, ^30^] In addition, a toluene molecule is capping the cation in a *η* ^3^‐fashion (K1−C43 3.344(2) Å, K1−C44 3.098(2) Å, K1−C45 3.152(2) Å) which prevents the formation of a coordination polymer, frequently observed for related potassium complexes based on **I–IV**[^16a^, ^16b^, ^16d^] or H^Dipp^NacNac.^[31]^

**Figure 4:**
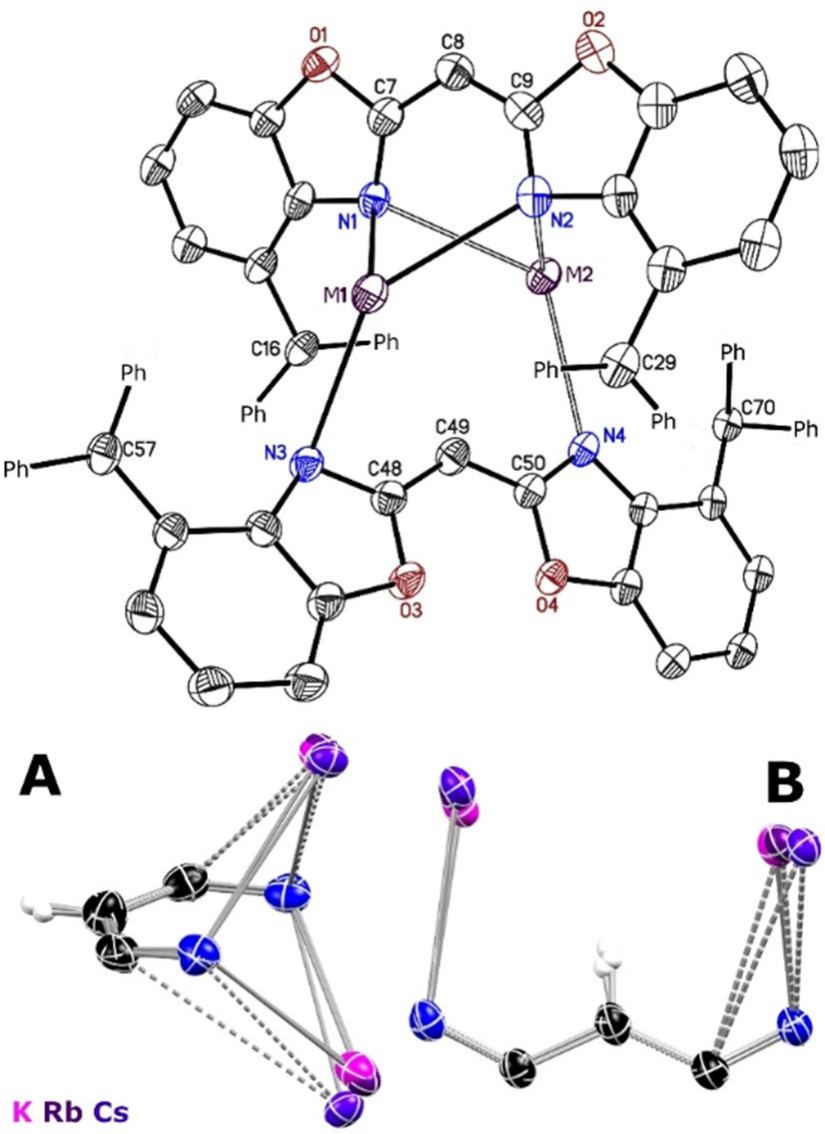
Molecular structure of dimeric {bis(4‐benzhydryl‐benzoxazol‐2‐yl)methanide} alkali metal complexes ([{*M*(^4‐BzhH2^BoxCH)}_2_], *M*=K (**8_2_**), Rb (**9**), Cs (**10**)). Anisotropic displacement parameters are depicted at the 50 % probability level. All hydrogen atoms are omitted for clarity. Superposition of **8_2_** ‐**10** showing NacNac‐like C_3_N_2_ unit of (*E*,*E*)‐ (^4‐BzhH2^BoxCH) (**A**) as well as twisted (*E*,*E*)‐(^4‐BzhH2^BoxCH) (**B**) and their coordinating alkali metal ion, respectively.

The second species derived from **8** is the dimeric donor‐base free **8_2_** in the monoclinic space group *P*2_1_/*c*. The three toluene lattice molecules in the asymmetric unit do not coordinate to the metal. Isostructural molecular structures were observed for the rubidium (**9**) (YLD: 76 %) and caesium (**10**) (YLD: 74 %) complexes (Figure 4, Table 1). They were synthesised via facile deprotonation of ligand **6** with neat rubidium and caesium metal (Scheme 2). All three compounds show a monoanionic (*E*,*E*)‐bis(4‐benzhydryl‐benzoxazol‐2‐yl)methanide} moiety coordinating two metal ions in *κ* ^1^,*η* ^2^‐fashion (N1−N2−C9; N1−N2−C7) by the NacNac‐like C_3_N_2_ unit (Figure 4A), respectively.^[32]^ In addition, a single phenyl group of each benzhydryl substituent forms an additional *η* ^6^‐ or *η* ^2^‐coordination.^[28]^ A second deprotonated (*Z*,*Z*)‐isomer of **6** (Figure 4B) bridges both metals in an *η* ^3^‐fashion (C49−C48−N3; N4−C50−C49). The coordination sphere of the cations is again satiated by *π*‐arene interactions of the adjacent phenyl groups (*η* ^4^ and *η* ^2^).^[28]^ Moreover, close interactions of the potassium ion and a suitable arranged methanide group in dimeric **8_2_** culminate in rather short potassium hydrogen distances (K1−H49 2.89(3) Å, K2−H49 2.77(3) Å) and an elongated C−H bond to 1.01(3) Å.^[33]^ The K−H49‐C49 angle of 102(2)° for K1 and 127(2)° for K2 also witness these interactions, because they are similar to e. g. [{K(THF)_4_}_2_Ln{(*μ*‐H)_2_BC_8_H_14_}_4_] (Ln=Eu, Yb) (K−H 2.64(4) to 3.37(3) Å, K−H‐C 117.8 to 120.7°)^[34]^ or [K(*Ar*)_2_] [*M*{N(SiMe_3_)_2_}_3_] (*M*=Mg, *Ar*=benzene, toluene, *p*‐xylene; M=Zn, *Ar*=*o*‐xylene) (K−H 2.81 to 3.07 Å, K−H‐C 85 to 145°).^[35]^ The solid‐state structures of **9** and **10** also display interactions of metal ions and methanide bridges, but due to the increased residual electron density for the heavier homologues the determination of the freely refined hydrogen atom positions is not that reliable. Rising effective ionic radii,^[36]^ increasing polarizability and softness of the cations are encountered moving down the alkali metal group on account of the shielding effect.[^28a^, ^37^]

These features (Table 1) cause rising metal nitrogen bonds for deprotonated (*Z*,*Z*)‐ and (*E*,*E*)‐(^4‐BzhH2^BoxCH) (**8_2_**: 2.775(2) to 3.015(2) Å; **9**: 2.905(5) to 3.063(4) Å; **10**: 3.033(3) to 3.285(3) Å), more acute average N−M−N angles (**8_2_**: 65.8°; **9**: 64.3°; **10**: 62.6°) as well as a growing dislocation of the metal from the C_3_N_2_ plane (**8_2_**: 1.877(4) to 2.282(3) Å; **9**: 2.036(7) to 2.303(7) Å; **10**: 2.302(5) to 2.430(5) Å) as well as from the −HC_2_N unit (**8_2_**: 0.85(5) to 2.18(3) Å; **9**: 0.699(13) to 2.196(9) Å; **10**: 1.16(8) to 2.60(5) Å). Interestingly, the detected butterfly folding angles in both isomers (*Z*,*Z*)‐(^4‐BzhH2^BoxCH)≈23°; (*E*,*E*)‐(^4‐BzhH2^BoxCH)≈16°) are negligibly affected while coordinating the alkali metal ions. Additionally, all alkali metal complexes **8_2_**–**10** were studied by ^1^H and ^13^C{^1^H} NMR spectroscopy in [D_8_]THF. Recorded ^1^H NMR spectra show a distinctive pattern of chemical shifts arising from the *C*_2v_ symmetry of monoanionic bis(benzoxazol‐2‐yl)methanide scaffold and the benzylic bound protons, whereas phenylic protons of the benzhydryl groups could not be clearly assigned due to their peak overlap. Deprotonation of **6** results in an accumulation of electron density in the benzene periphery of both benzoxazol‐2‐yl moieties that again entails a general shielding of corresponding protons and an upfield shift. Concerning the ^1^H NMR shifts for the C−H bridging position, a minute but continuous decline along K (4.66 ppm)>Rb (4.65 ppm)>Cs (4.64 ppm) complexes is noticed. The opposite is found for para positioned protons (3‐, 13‐H) and benzylic protons. These protons exhibit a small but noticeable downfield shift (*δ*(3‐, 13‐H)=6.33 ppm to 6.43 ppm; *δ*(16‐, 29‐H)=6.03 ppm to 6.17 ppm) which is in tune with the negative charge being restored in the phenylic benzhydryl groups yielding a growing metal arene interaction. Remaining benzoxazol‐2‐yl protons in ortho‐ and meta‐position shifts (4‐,12‐ and 5‐, 11‐H) do not seem to be influenced by varying alkali cations. Although ^1^H NMR spectra of **8**–**10** ([D_8_]THF) and sodium complex **7** ([D_8_]toluene) were measured in different solvents, benzylic protons (16‐, 29‐H) display a remarkable change of the average chemical shifts (▵*δ*=0.82 ppm). The ^87^Rb NMR spectrum of **9** from THF solution shows two singlets. Most probably [{Rb(^4‐BzhH2^BoxCH)}_2_] rearranges to give a THF‐solvated rubidium cation [Rb(THF)_*n*_] (*δ*=−1.69 ppm) and a bis(4‐benzhydryl‐benzoxazol‐2‐yl)methanide} based anion [Rb(^4‐BzhH2^BoxCH)_2_]^−^ (*δ*=−254.69 ppm). The ^133^Cs NMR spectrum of **10** displays only one signal at *δ*=−31.12 ppm. Additionally, elemental analysis and mass spectrometry (LIFDI, THF) confirmed the synthesis of heavier alkali complexes **8**–**10**.

**Scheme 3:**
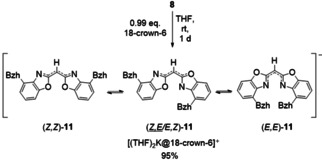
Reaction of monomeric complexes **8** to solvent separated ion [(THF)_2_K(18‐crown‐6)]^+^[^4‐BzhH2^BoxCH]^−^ (**11**) via addition of one equivalent 18‐crown‐6. In solution permanent rearrangement of the (*Z*,*Z*)‐, (*Z*,*E*/*E,Z*)‐, and (*E*,*E*)‐(^4‐BzhH2^BoxCH) isomers is ubiquitous.

**Figure 5:**
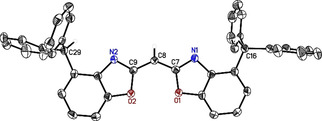
Molecular structure of {bis(4‐benzhydryl‐benzoxazol‐2‐yl)methanide} anion from **11**, [(THF)_2_K(18‐crown‐6)]^+^[^4‐BzhH2^BoxCH]^−^. Anisotropic displacement parameters are depicted at the 50 % probability level. All hydrogen atoms are omitted for clarity except for the bridging (methylene) and benzylic ones. Selected bond lengths [Å] and bond angles [°]: N1−C7 1.329(2), N2−C9 1.327(2), C7−C8 1.390(2), C8−C9 1.389(2), C9−C8−C7 131.34(15).

On the basis of reported (*Z*,*Z*)‐ and (*E*,*E*)‐isomers of complex **8**–**10** and based on former gained knowledge of sterically less demanding bis(benzoxazol‐2‐yl)methanide potassium complexes[^16a^, ^16b^] and their related 18‐crown‐6 derivatives,[^16c^, ^16d^, ^38^] we were curious whether the reaction of **8** with 18‐crown‐6 would prefer the formation of a (*Z*,*Z*), (*E*,*E*) or (*Z*,*E*) isomeric species. Therefore bis(4‐benzhydryl‐benzoxazol‐2‐yl)methane was first deprotonated with an excess of potassium hydride in THF at ambient temperature (Scheme 3). After the reaction mixture had been stirred for 1 d, excess of hydride was removed by filtration and 18‐crown‐6 was added to the solution. Within seconds a strong blue fluorescence (*λ*_max_=454 nm, *λ*_ex_=350 nm, detail see Supporting Information) of the dark red solution becomes entrenched. The reaction mixture was further stirred for 1 d at ambient temperature, volatiles were removed in vacuum and the solvent separated ion pair [(THF)_2_K(18‐crown‐6)]^+^ [^4‐BzhH2^BoxCH]^−^ (**11**) was isolated as a reddish solid in excellent yields (95 %). Crystals suitable for single XRD experiments were grown by vapour diffusion of pentane to a saturated THF solution of **11** at ambient temperature after 3 d. The colourless crystals in the triclinic space group *P* $\bar{1}$ contain one bis(4‐benzhydryl‐benzoxazol‐2‐yl)methanide anion and two halve (THF)_2_K(18‐crown‐6)} cations in the asymmetric unit (Figure 5). In contrast to [(*L*)K(18‐crown‐6)] (*L*=**I** ^−^, **III** ^−^)[^16c^, ^16d^, ^38^] that develop contact ion pairs in the solid, complex **11** was found to form a solvent separated ion pair. Expectedly, the (*Z*,*Z*)‐(^4‐BzhH2^BoxCH) isomer seems to be preferred over the (*Z*,*E*/*E,Z*)‐ or (*E*,*E*)‐(^4‐BzhH2^BoxCH) isomers in the solid‐state. In similar manner the formation of a (*Z*,*Z*)‐isomer accompanied by only an oxygen atom coordination at the metal is found in [(**III** ^−^)K(18‐crown‐6) ⋅ H_2_O].[^16c^, ^38^] The molecular structure of (*Z*,*Z*)‐(^4‐BzhH2^BoxCH) (**11**) displays a butterfly folding angle of 5.03(9)°, which is almost consistent to the folding angles observed in monomeric species of **8_1_** (5.54(8)°) as well as **7** (4.82(6)°). This obtuse‐angled benzoxazol‐2‐zyl moieties and the bond length of C_3_N_2_ unit indicate a fully conjugated system extended throughout the virtually planar ligand.^[39]^ Based on these findings, the Gibbs free energy differences ▵*G* of the monoanionic (*E,E*)‐, (*Z,E*/*E,Z*)‐ or (*E,E*)‐(^4−Bzh^BoxCH) isomers were calculated by density functional theory at the BP86/def2‐SVP level (see details in Supporting Information). Calculated Gibbs free energy differences is for all configurational isomers ▵*G*<14 kcal/mol. Hence in solution most likely the permanent rotation around the methanide linker, accompanied by rearrangement of the isomers (Scheme 3), is ubiquitous. Moreover, the synthesis of [{(THF)_2_K(18‐crown‐6)}(^4‐BzhH2^BoxCH)] (**11**) was confirmed by mass spectrometry (ESI[−],THF) and elemental analysis.

**Scheme 4:**
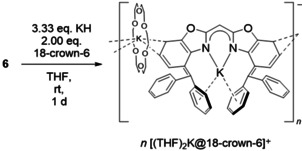
Optimised synthesis of polymeric [{(THF)_2_K(18‐crown‐6)}{K(18‐crown‐6)K(^4‐Bzh^BoxCH)}]_*n*_ (*n*→∞) (**12**) by addition of 3.33 equivalents of KH and two equivalents of 18‐crown‐6.

**Figure 6:**
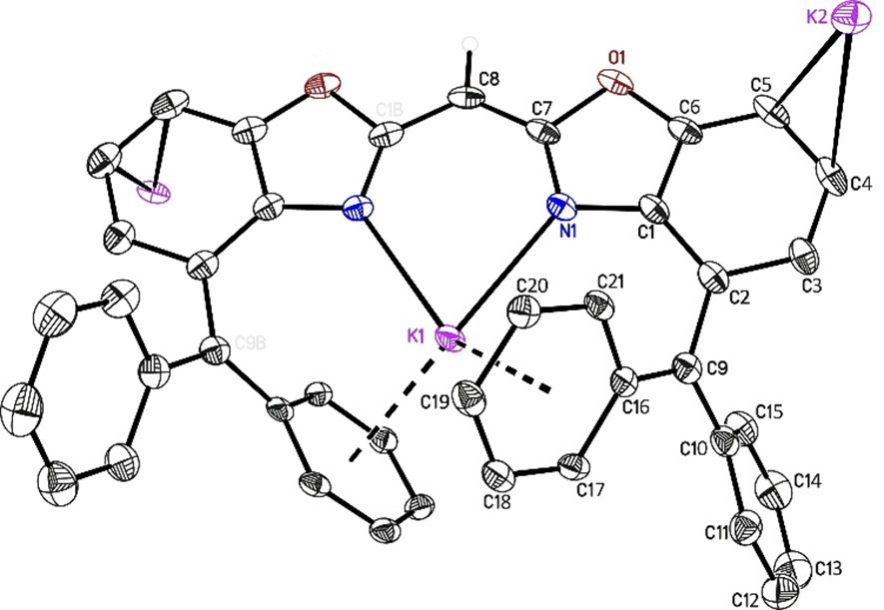
Molecular structure of [{(THF)_2_K@(18‐crown‐6)}{K@(18‐crown‐6) K‐(^4‐Bzh^BoxCH)}]_*n*_ (*n*→∞) (**12**). Anisotropic displacement parameters are depicted at the 50 % probability level. All hydrogen atoms are omitted for clarity except for the bridging (methylene) one. K1−N1 2.686(3), K1−C16 2.930(3), K1−C17 3.084(3), K1−C18 3.255(3), K1−C19 3.291(3), K1−C20 3.154(3), K1−C21 2.993(3), N1−C7 1.324(4), C7−C8 1.393(4), C2−C9 1.478(4), C9−C10 1.459(5), C9−C16 1.426(4), C4−K2 3.329(3) Å, C5−K2 3.245(8) Å, N1−K1−N1A 72.78(11), C1−C2−C3 114.2(3), C6−C5−C4 115.1(4), C15−C10−C11 115.7(3), C17−C16−C21 114.4(3).

Since Brown or Buncel and Menon ascertained that triphenylmethane is deprotonated in THF by potassium hydride when DMF^[40]^ or 18‐crown‐6^[41]^ is added,^[42]^ we wanted to examine whether deprotonation of one or both benzylic functions can be accomplished via reaction of one or two equivalents of hydride and crown ether, respectively, to a solution of **8**. However, the reactions of different amounts of KH and 18‐crown‐6 all yielded the same complex [{(THF)_2_K(18‐crown‐6)}{K_2_(^4‐Bzh^BoxCH)}]_*n*_ (*n*→∞) **12** as very sensitive dark purple crystals by storing THF solutions at −30 °C. The crystal selection and mounting was challenging due to the dark colour and high sensitivity of compound **12**. Thus, a moderate single crystal XRD data set could be collected after many attempts. Furthermore, the synthesis of **12** was improved by using ligand **6** and the exact stoichiometric quantities (^4‐BzhH2^BoxCH_2_:KH:18‐crown‐6=1 : 3 : 2) (Scheme 4) that are based on the crystal structure. Immediately after adding the starting materials to a solution of **6** in THF, a solid blue fluorescence of the dark red solution was observed. The mixture was stirred at least for 1 d, whereas a red solid precipitated that was separated from the supernatant solution by decantation. The precipitate was washed with pentane, dried under reduced pressure, and stored at −30 °C for further syntheses or analyses (YLD: 95 %). Obtained THF solution was cooled to −30 °C for crystallisation. Complex **12** crystallises in the monoclinic space group *P*2/*n* with half a formula unit in the asymmetric unit (Figure 6). The coordination polymer comprises solvent‐separated potassium ion (K3) solely coordinated by the oxygen atoms of 18‐crown‐6 and two THF molecules. This is omitted in Figure 6 for clarity. A second potassium ion (K2) is *η* ^2^‐coordinated (C4−K2 3.329(3) Å, C5−K2 3.245(8) Å) to the aryl moieties of two adjacent (*E*,*E*)‐trianions and also coordinated by an equatorial crown ether molecule. The *η* ^2^‐coordination provides the link in the polymeric structure. Finally, a third potassium ion in the coordination pocket is surrounded by two nitrogen atoms in *κ* ^2^‐fashion (N−K1 2.686(3) Å) and a phenyl ring of each benzhydryl moiety in asymmetrically *η* ^6^‐fashion (C_Ph_−K1: 2.930(3) Å to 3.291(3) Å), respectively. Determined nitrogen potassium distances and the perfect planarity of the ligand scaffold with the potassium ion in the plane of the trianion are in good agreement with the unsolvated *κ* ^2^‐*N*,*N*‐[K(^Dipp^NacNac)]^[31]^ complex (N1−K1 2.710(3) Å, N2−K1 2.652(2) Å). The bond lengths to flanking C_ipso_‐atoms are basically shortened (1.426(4) to 1.478(4) Å) compared to *H*C_bzh_−C_ipso_ of complexes **6**–**11** (1.508(8) Å to 1.538(6) Å) because of the deprotonation of the two benzylic positions (C_bzh_) (benzhydryl group). The two anionic C9‐atoms are essentially in plane with the bound C_ipso_‐atoms (C2, C10, C16) similar to previously studied potassium trityl complexes [KCPh_3_(THF)(PMDTA)],^[30]^ [KCPh_3_(PMDTA)]^[43]^ or [KCPh_3_(*L*)]_*n*_ (*L*=diglyme, THF).^[43]^ Angles around C9 deviate slightly from the typical 120° of sp^2^‐hybridised carbon atoms (for details see Supporting Information), while the steric constraints prevent the phenyl rings from being coplanar leading to a propeller‐like arrangement. Within this arrangement, angles *φ* of the phenyl groups with respect to C2−C10−C16 plane (C_3_‐plane) are *φ*=32.38(17)° (C10 to C15), and 49.68(10)° (C1 to C6), whereas the smallest angle and shortest bond (C9−C16 1.426(4) Å) involves the phenyl system to which the cation is coordinated. These parameters correspond to an increased *π*‐electron‐delocalisation that is correlated with the overlap of the C_3_‐plane (C2−C10−C16) and coordinating phenyl ring (C16 to C21) which is dependent on cos(*φ*)^2^ function[^30^, ^44^] of the corresponding plane angles. Although the phenyl group is twisted (*φ*=16.4(2)°) the overlap amounts to about 92 %, that is a more significant overlap than observed for [KCPh_3_(THF)(PMDTA)]^[30]^ (*φ*=17.7°,≈90 %). Further direct analyses of **12** were challenging due to its low solubility in most solvents (toluene, benzene, or THF). To find further evidence for the synthesis of trifold deprotonated anion (^4‐Bzh^BoxCH), suspensions of the dark red precipitate (**12**) were once again protonated (excess≈20 eq. H_2_O or D_2_O) in small scale (NMR experiment). These ^1^H and ^2^D NMR experiments (for details see Supporting Information), as well as mass spectrometry (ESI[−] HRMS, THF), confirmed the previous synthesis of **12**.

## Conclusion

Within this work, the six‐step synthesis and characterisation of a novel bulky bis(benzoxazol‐2‐yl)methane ligand **6** (^4−BzhH2^BoxCH_2_) comprising benzhydryl groups at both C4‐positions (ortho imine positions) in spatial proximity to the coordination pocket was presented. To get a better knowledge of its properties and find a possible precursor complex for subsequent salt metathesis reactions, **6** was deprotonated with alkali metal bases. Obtained products were analysed by NMR spectroscopy, mass spectrometry as well as single crystal XRD experiments. Crystals grown from toluene solutions unveiled monomeric [*M*(^4‐BzhH2^BoxCH)] (*M*=Na (**7**), K (**8_1_**)) and dimeric [{*M*(^4‐BzhH2^BoxCH)}_2_] (*M*=K (**8_2_**), Rb (**9**), Cs (**10**)) species in solid state. Latter alkali metal complexes display distorted *κ* ^2^‐*N*,*N*‐coordinated (*Z*,*Z*)‐ and an (*E*,*E*)‐(^4‐BzhH2^BoxCH) configurational isomers, which display various polyhaptic metal arene interactions. Furthermore, potassium ion sequestration of **8** by 18‐crown‐6 resulted in [{(THF)_2_K(18‐crown‐6)}(^4‐BzhH2^BoxCH)] (**11**) a solvent separated ion pair containing energetically favoured monoanionic (*E*,*E*)‐(^4‐BzhH2^BoxCH) ligand. Additionally, reaction of ^4‐BzhH2^BoxCH_2_ with three equivalents KH and two equivalents 18‐crown‐6 yielded polymeric [{(THF)_2_K@(18‐crown‐6)}{K@(18‐crown‐6)K(^4‐Bzh^BoxCH)}]_*n*_ (*n*→∞) (**12**) featuring a remarkable trianionic ligand. The single crystal X‐ray diffraction experiment of **12** revealed one of the potassium ions to be *η* ^2^‐coordinated by the benzoxazol‐2‐yl scaffold of two adjacent (*E*,*E*)‐ligands plus a crown ether molecule. A second potassium ion is surrounded by two nitrogen atoms in *κ* ^2^‐fashion as well as one phenyl ring of each benzhydryl moiety in a symmetrically *η* ^6^‐fashion, respectively. Future work with regard to the mono‐ (^4‐BzhH2^BoxCH) and trianionic (^4‐Bzh^BoxCH) ligand will focus on the coordination of other metal, i. e. those from Group 13 or lanthanides. The benzhydryl groups attached to the benzoxazol‐2‐yl scaffold seem to exhibit similar qualities to the triphenylmethane, which is able to stabilise carbanionic, radical or carbonium ionic species owing to the extensive *π*‐delocalisation. With this in mind, future research might focus on the synthesis of ^4‐BzhH2^BoxCH_2_ based radical and carbonium ion compounds.

## Experimental section

General procedures: All reactions were carried out under an atmosphere of N2 and Ar by Schlenk techniques. Solvents were distilled from Na, K or NaK before used for synthesis. Starting materials were purchased commercially and used without further purification. 1D (1H, 13C, 87Rb, 133Cs) and 2D (1H,13C HSQC, 1H,13C and 1H,15N HMBC) NMR spectroscopic data were recorded on a Bruker Ascend 500 MHz, 400 MHz, and Avance 300 MHz spectrometer and referenced to the deuterated solvent (benzene‐ *d*_6_, thf‐ *d*_8_, toluene‐ *d*_8_).^**[45]**^ Deuterated benzene and toluene were dried over K (65 °C), THF was pre‐dried with LiAlH_4_ and stored over activated molecular sieve (3 Å) and stored in an argon dry box. Elemental analyses (C, H, N) were performed on a Vario EL3 at the Mikroanalytische Labor, Institut für Anorganische Chemie, University of Göttingen. LIFDI‐MS spectra were measured on a Jeol AccuTOF spectrometer and ESI (HR‐MS) measurements were performed on Bruker maXis spectrometer. All p*K*_a_ measurements in acetonitrile were carried out as in reference [13]. Shock‐cooled crystals were selected from a Schlenk flask under argon atmosphere using the X‐TEMP2 device.^[46]^ The data were collected with a Mo‐ (**6**, **8_1_**, **9**, **10**, **11**) or Ag‐ (**7**, **8_2_**, **12**) IμS microfocus source.^[47]^ All data were integrated with SAINT,^[48]^ and a multiscan absorption correction (SADABS^[49]^) was applied. For structure **6** a 3λ correction was applied.^[50]^ The structures were solved by direct methods (SHELXT^[51]^) and refined on F^2^ using the full‐matrix least‐squares methods of SHELXL^[52]^ within the SHELXLE GUI.^[53]^ Deposition Numbers 2031907 (for **6**),2031908 (for **7**), 2031909 (for **8**_**1**_), 2031910 (for **8**_**2**_), 2031911 (for **9**), 2031912 (for **10**), 2031913 (for **11**), and 2031914 (for **12**) contains the supplementary crystallographic data for this paper. These data are provided free of charge by the joint Cambridge Crystallographic Data Centre and Fachinformationszentrum Karlsruhe Access Structures service www.ccdc.cam.ac.uk/structures.

## Conflict of interest

The authors declare no conflict of interest.

## Supporting information

As a service to our authors and readers, this journal provides supporting information supplied by the authors. Such materials are peer reviewed and may be re‐organized for online delivery, but are not copy‐edited or typeset. Technical support issues arising from supporting information (other than missing files) should be addressed to the authors.

Supplementary
