# Supplementary material for: Bis(4‐benzhydryl‐benzoxazol‐2‐yl)methane – from a Bulky NacNac Alternative to a Trianion in Alkali Metal Complexes
Source: Chemistry. 2021 May 25;27(38):9858–65. doi: 10.1002/chem.202100616 (PMC8361911; doi:10.1002/chem.202100616)
Supplement: Supplementary file 1 — Supplementary [file CHEM-27-9858-s001.pdf]

Chemistry–A European Journal

Supporting Information

Bis(4-benzhydryl-benzoxazol-2-yl)methane – from a Bulky NacNac Alternative to a Trianion in Alkali Metal Complexes

Johannes Kretsch, Anne-Kathrin Kreyenschmidt, Timo Schillmöller, Märt Lõkov, Regine Herbst-Irmer, Ivo Leito, and Dietmar Stalke*

Inhalt

Compound 1: Synthesis and Analytical Data 3
NMR spectroscopy 4
Mass spectrometry 6
Compound 2: Synthesis and Analytical Data 8
NMR spectroscopy 9
Mass spectrometry 11
Compound 3: Synthesis and Analytical Data 13
NMR spectroscopy 14
Mass spectrometry 16
Compound 4: Synthesis and Analytical Data 17
NMR spectroscopy 18
Mass spectrometry 20
Compound 5: Synthesis and Analytical Data 22
NMR spectroscopy 23
Mass spectrometry 25
Compound 6: Synthesis and Analytical Data 27
NMR spectroscopy 29
Mass spectrometry 37
Compound 7: Synthesis and Analytical Data 39
NMR spectroscopy 40
Mass spectrometry 43
Compound 8: Synthesis and Analytical Data 44
NMR spectroscopy 45
Mass spectrometry 47
Compound 9: Synthesis and Analytical Data 48
NMR spectroscopy 49
Mass spectrometry 52
Compound 10: Synthesis and Analytical Data 53
NMR spectroscopy 54
Mass spectrometry 57
Compound 11: Synthesis and Analytical Data 58
NMR spectroscopy 60

Mass spectrometry 65
Compound 12: Synthesis and Analytical Data 68
Mass spectrometry 70
Experimental setup and methodology for pK_a determination 72
Fluorescence measurements 73
Computational studies 74
Xray Crystallographic Analysis 80
XRAY: Compound 6 83
XRAY: Compound 7 86
XRAY: Compound 8m 90
XRAY: Compound 8d 95
XRAY: Compound 9 104
XRAY: Compound 10 114
XRAY: Compound 11 132
XRAY: Compound 12 137
References 145

Compound 1: Synthesis and Analytical Data

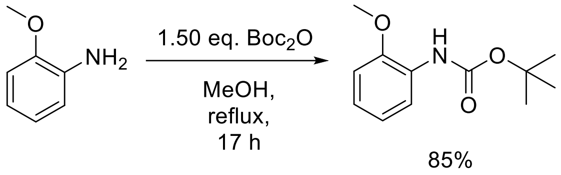

Alternative Synthesis of tert-butyl-(2-methoxyphenyl)-carbamate (1): 2-Anisidine (20.93 g, 19.20 mL (1.09 g/mL), 169.9 mmol, 1.00 eq.), di-tert-butyl-dicarbamate (55.63 g, 54.54 mL (1.02 g/mL), 254.9 mmol, 1.50 eq.) were dissolved in methanol (300 mL). The slightly brown reaction solution was heated under reflux (~100°C) for 18 h. The mixture was cooled to room temperature, solvent was removed under vacuum and demineralised water (100 mL) was added. The aqueous phase was separated and extracted with DCM (3 × 30 mL). Subsequently, the organic phase was washed with saturated, aqueous sodium chloride solution (1 × 40 mL) and dried over $MgSO_4$. After the organic solvent was removed under reduced pressure a brownish, oily substance was obtained. This substance was distilled under vacuum (fine vacuum) at 130°C and the colourless oil was used without further purification. Yield: 32.25 g (85%).

^{1}H NMR (300 MHz, $CDCl_3$, 298 K): δ = 8.10 (m, 1 H, 2-), 7.12 (s_{br}, 1 H, NH), 7.00-6.91 (m, 2 H, 3-H, 4-H), 6.88-6.81 (m, 1 H, 5-H), 3.84 (s, 3 H, OCH_3), 1.54 (s, 9 H, $C(CH_3)_3$) ppm.

^{13}C{^{1}H} NMR (75 MHz, $CDCl_3$, 298 K): δ = 152.8 (-*C*OO*t*Bu), 147.6 (6-C), 128.2 (1-C), 122.3 (4-C), 121.1 (3-C), 118.1 (2-C), 109.9 (5-C), 80.2 (-*C*$(CH_3)_3$), 55.6 (-O*C*H_3), 28.4 (-C$(CH_3)_3$) ppm.

MS (ESI[+], THF) *m/z* (%): 246.1 (53) $[M+Na]^+$, 224.1 (11) $[M+H]^+$, 168.1 (55) $[M-t\mathrm{Bu}+H]^+$, 124.1 (100) $M-t\mathrm{Bu}-CO_2+H]^+$.

HR-MS (ESI[+], THF) *m/z*: 224.1280 (cal. 224.1280 for $[M+H]^+$, $C_{12}H_{18}NO_3$), 246.1103 (cal. 246.1101 for $[M+Na]^+$, $C_{12}H_{18}NNaO_3$).

Elemental analysis in % (calculated) $C_{14}H_{21}NO_3$ (223.27 g/mol): C 64.71 (64.55), H 7.67 (7.67), N 6.31 (6.27).

NMR spectroscopy

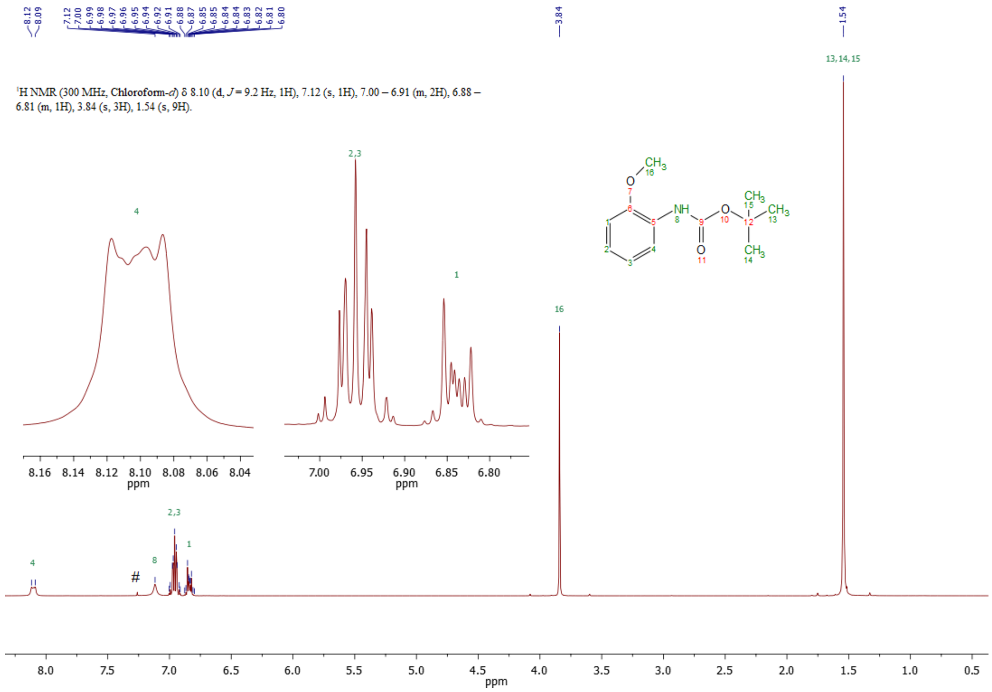

Figure S1. ^{1}H-NMR spectrum of **1** in $CDCl_3$. The residual solvent signal is marked with #.

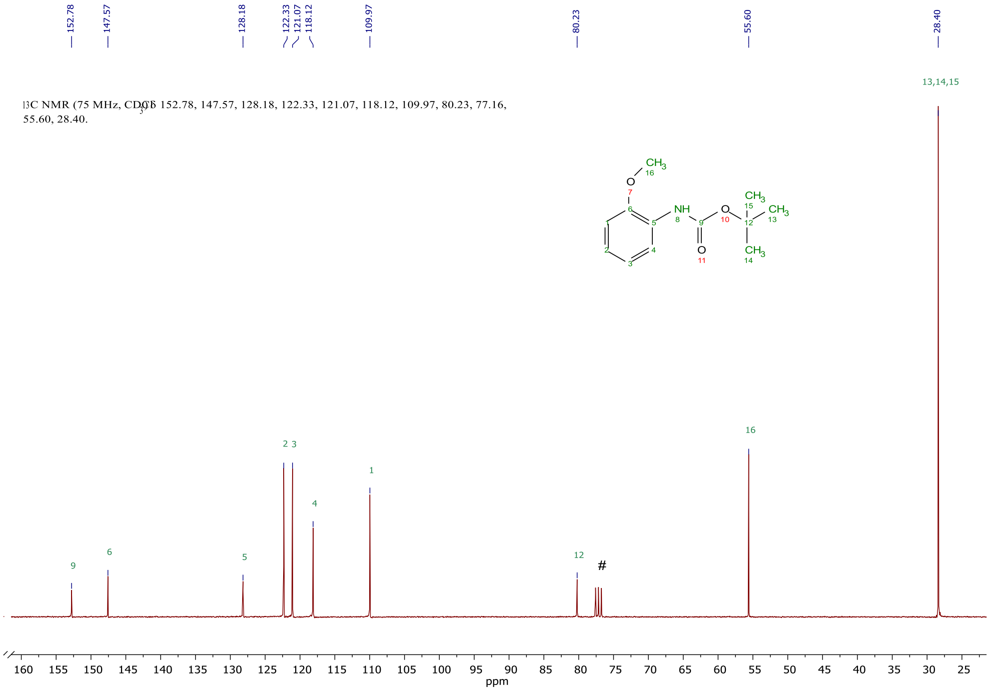

Figure S2. ^{13}C NMR spectrum of **1** in $CDCl_3$. The residual solvent signal is marked with #.

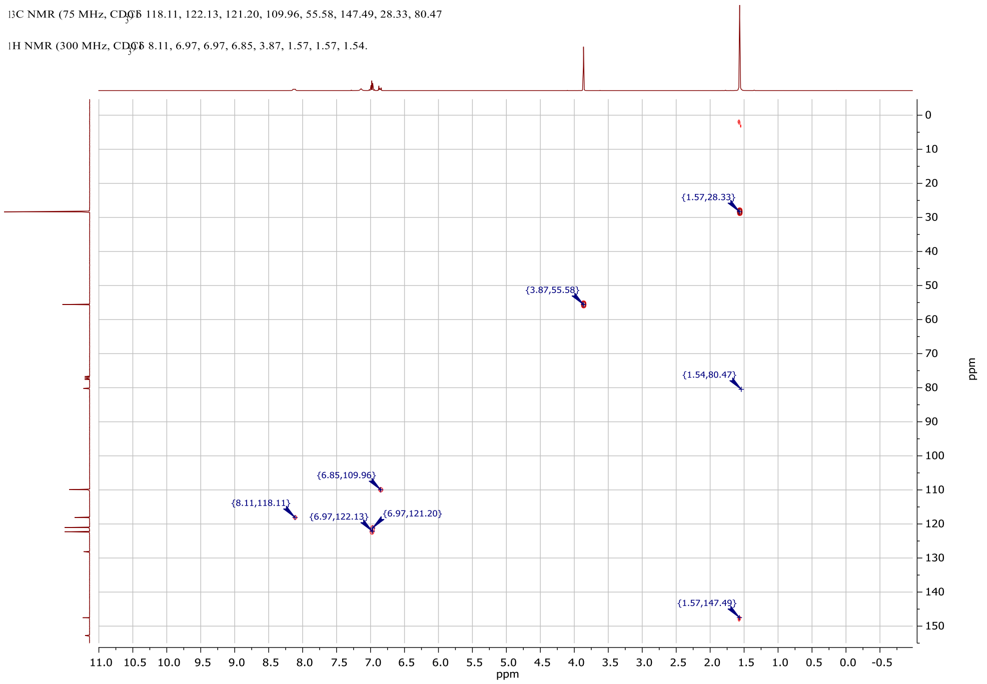

Figure S3. ^{1}H,^{13}C HSQC NMR spectrum of **1** in $CDCl_3$.

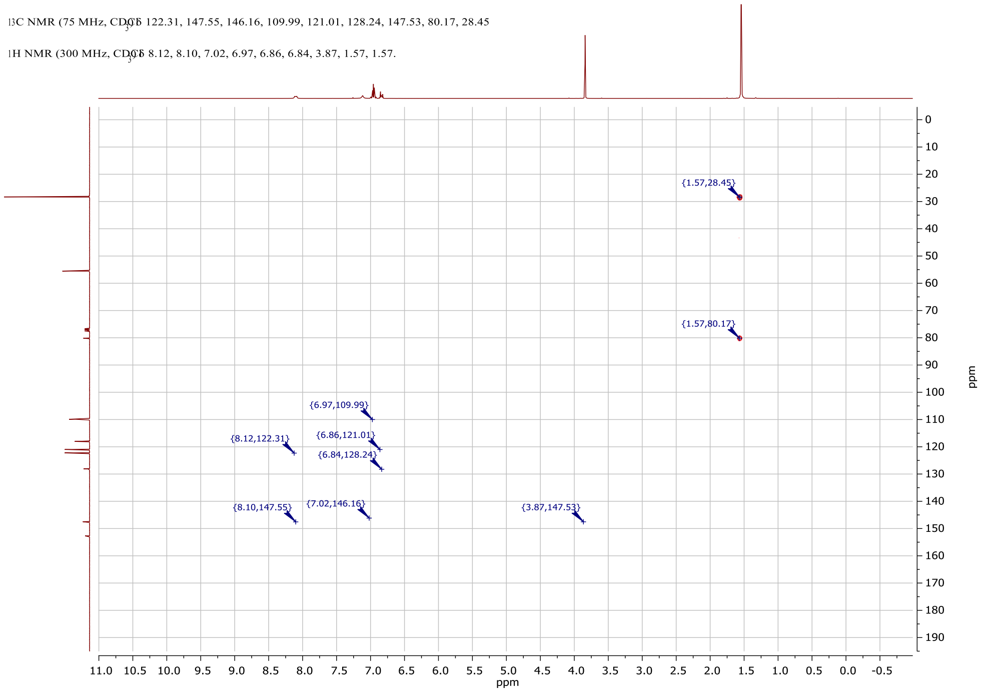

Figure S4. ^{1}H,^{13}C HMBC NMR spectrum of **1** in $CDCl_3$.

Mass spectrometry

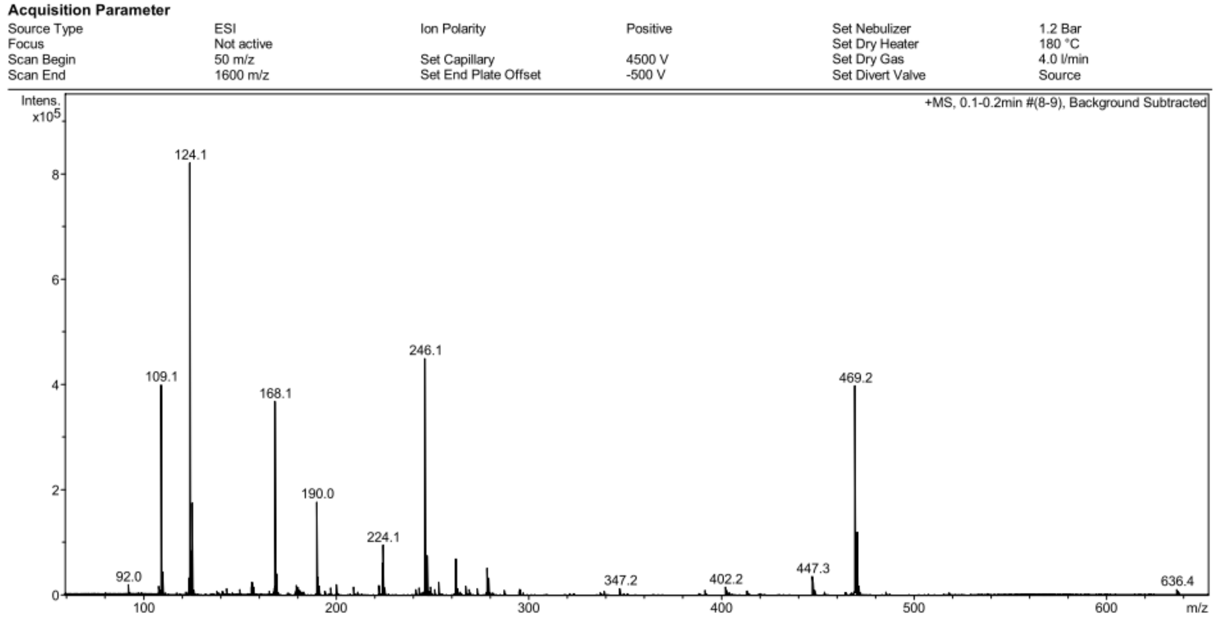

Figure S5. Mass spectrum of **1** (ESI[+], THF) *m/z* (%): 246.1 (53) [*M*+Na]$^+$, 224.1 (11) [*M*+H]$^+$, 168.1 (55) [*M*−*t*Bu+H]$^+$, 124.1 (100) *M*−*t*Bu−CO_2+H]$^+$.

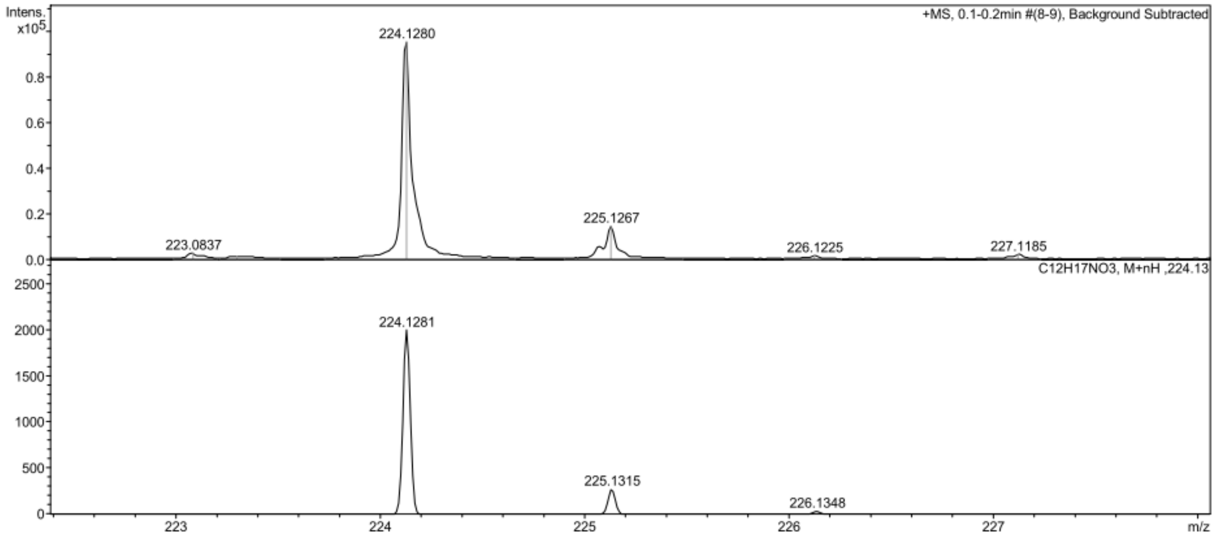

Figure S6. Mass spectrum (HR-MS (ESI[+], THF) of **1** *m/z*: 224.1280 (cal. 224.1280 for [*M*+H]$^+$, $C_{12}H_{18}NO_3$).

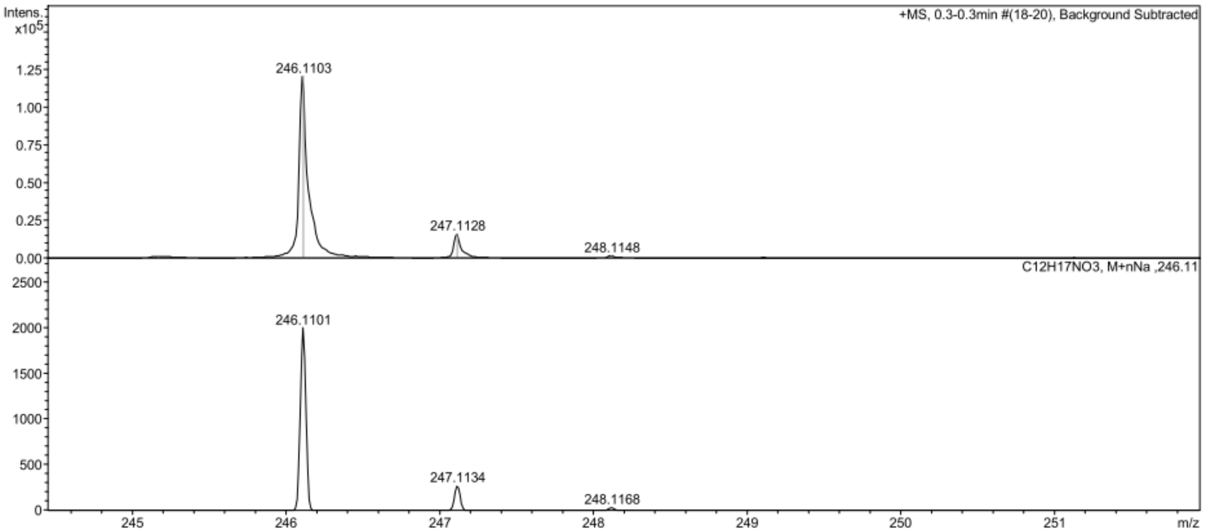

Figure S7. Mass spectrum (HR-MS (ESI[+], THF) of **1** *m/z*: 246.1103 (cal. 246.1101 for $[M+Na]^+$, $C_{12}H_{18}NNaO_3$).

Compound 2: Synthesis and Analytical Data

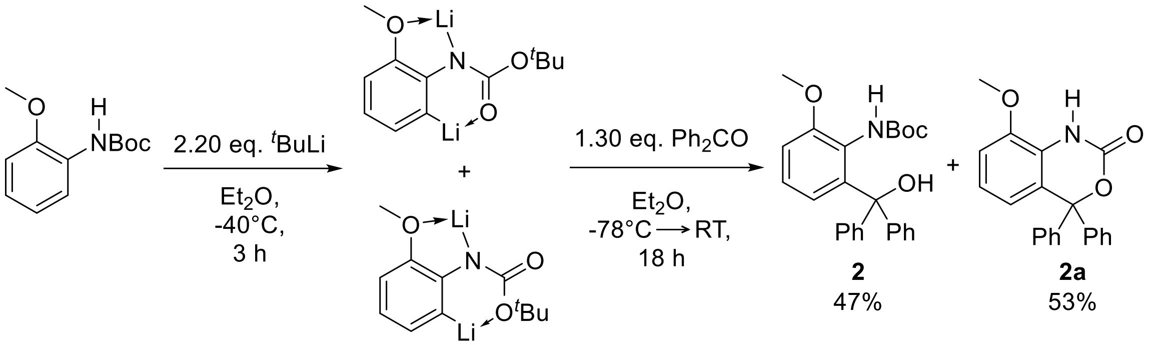

Alternative Synthesis of tert-butyl-(2-hydroxydiphenylmethyl)-6-methoxyphenyl)-carbamateol (2) and 8-methoxy-4,4-diphenyl-1,4-dihydro-2H-benzo[d][1,3]oxazin-2-one (2a): *tert*-butyl(2-methoxyphenyl)-carbamate (10.19 g, 45.64 mmol, 1.00 eq.) was introduced in a Schlenk flask (500 mL) and dry Et_2O (70 mL) was added. The solution was cooled to −45°C ($MeCN/CO_{2(s)}$) and a solution of *tert*-Butyllithium in pentane (53.98 mL (1.9 mol/L), 100.4 mmol, 2.20 eq) was slowly added to the reaction solution. After the reaction mixture was stirred between −40°C to −20°C for 3 h the mixture was cooled to −78°C ($EtOH/CO_{2(s)}$) and a solution of benzophenone (10.91 g, 59.33 mmol, 1.30 eq.) in Et_2O (40 mL) was added. The dark green solution was stirred overnight (~18 h), while it was slowly allowed to warm to room temperature. Subsequently demineralised water (100 mL) was carefully added to the solution whereupon a white solid, a yellow ether and a colourless water phase were observed. The white solid was separated by filtration and the liquid phases were filled in a separating funnel. After this separated white solid had been washed with hexane (200 mL) in an ultrasonic bath (10-15 min), it was again filtered and dried under fine vacuum. The organic phase was removed from the aqueous phase via separation funnel, and the aqueous phase was extracted with Et_2O (3 × 20 mL). United organic phases (Et_2O) were dried with $MgSO_4$. Volatiles were removed under reduced pressure, and the obtained oily, white solid was ultrasonicated with hexane (50 mL), filtered and dried under fine vacuum. Yield: 12.6 g (**2** = 6.56 g (14.56 mmol); **2a** = 6.04 g (18.24 mmol)) (**2**+**2a** = 75%)

Compound **2**: ^{1}H NMR (300 MHz, $CDCl_3$, 298 K): δ = 7.32-7.20 (m, 10 H, Ph), 7.04 (dd, 1 H, $^3J_{HH}$ = 8.1 Hz, 4-H), 6.91 (dd, 1 H, $^3J_{HH}$ = 8.3 Hz, $^4J_{HH}$ = 1.2 Hz, 3-H), 6.31 (dd, 1 H, $^3J_{HH}$ = 7.9 Hz, $^4J_{HH}$ = 1.2 Hz, 5-H), 6.14 (s_{br}, 1 H, NH), 4.38 (s_{br}, 1 H, OH), 3.82 (s, 3 H, -OCH_3), 1.26 (s, 9 H, -$C(CH_3)_3$) ppm.

Compound **2a**: ^{1}H NMR (300 MHz, $CDCl_3$, 298 K): δ = 7.39 (s_{br}, 1 H, NH), 7.34-7.31 (m, 6 H, *m*-Ph, *p*-Ph), 7.23-7.19 (n, 4 H, *o*-Ph), 6.96 (dd, 1 H, $^3J_{HH}$ = 7.7 Hz, 4-H), 6.89 (dd, 1 H, $^3J_{HH}$ = 8.3 Hz, $^4J_{HH}$ = 1.3 Hz, 3-H), 6.34 (dd, 1 H, $^3J_{HH}$ = 7.6 Hz, $^4J_{HH}$ = 0.7 Hz, 5-H), 3.89 (s, 3 H, -OCH_3) ppm.

MS (ESI[+], THF) *m/z* (%): 685.3 (88) [2×(**2a**)+Na]$^+$, 663.3 (12) [2×(**2a**)+H]$^+$, 354.1 (46) [(**2a**)+Na]$^+$, 332.1 (100) [(**2a**)]$^+$, 290.2 (94) [(**2**)-Boc-OH+H]$^+$.

HR-MS (ESI[+], THF) *m/z*: 354.1100 (cal. 354.1101 for [(**2a**)+Na]$^+$, $C_{21}H_{17}NNaO_3$), 332.1282 (cal. 332.1281 for [(**2a**)+H]$^+$, $C_{21}H_{18}NO_3$), 290.1538 (cal. 290.1539 for [(**2**)-Boc-OH+H]$^+$, $C_{12}H_{20}NO$).

NMR spectroscopy

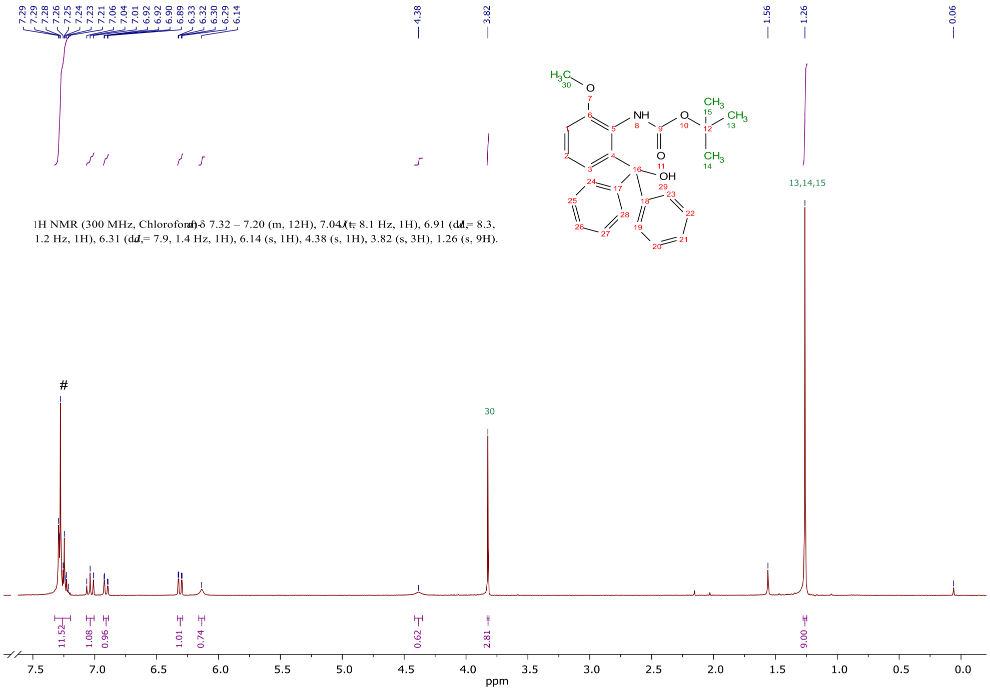

Figure S8. ^{1}H-NMR spectrum of **2** in $CDCl_3$. The residual solvent signal is marked with #.

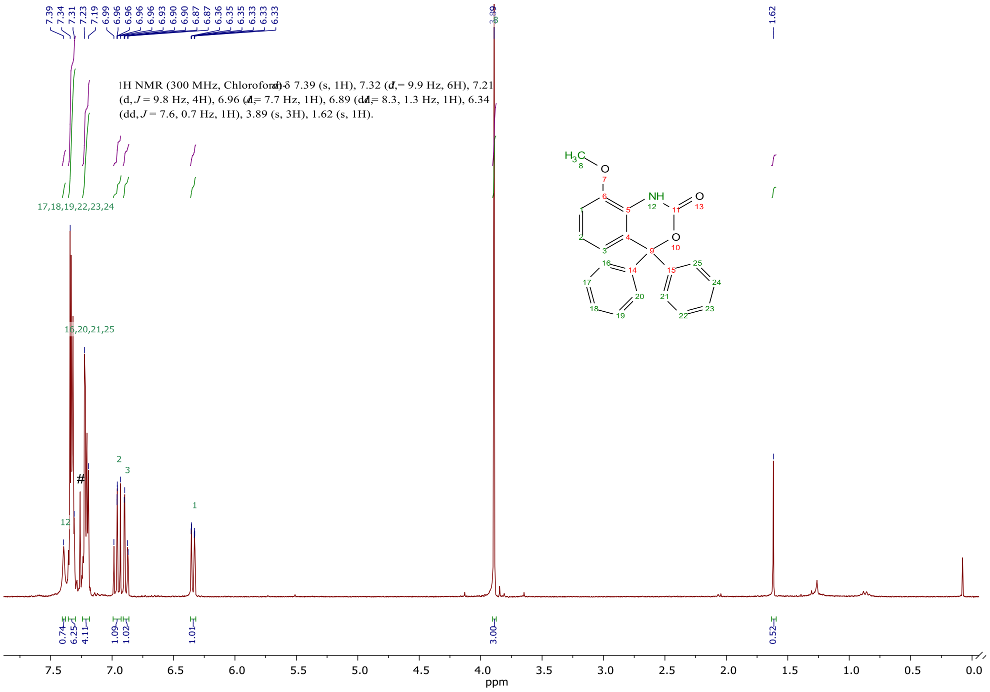

Figure S9. ^{1}H-NMR spectrum of **2a** in $CDCl_3$. The residual solvent signal is marked with #.

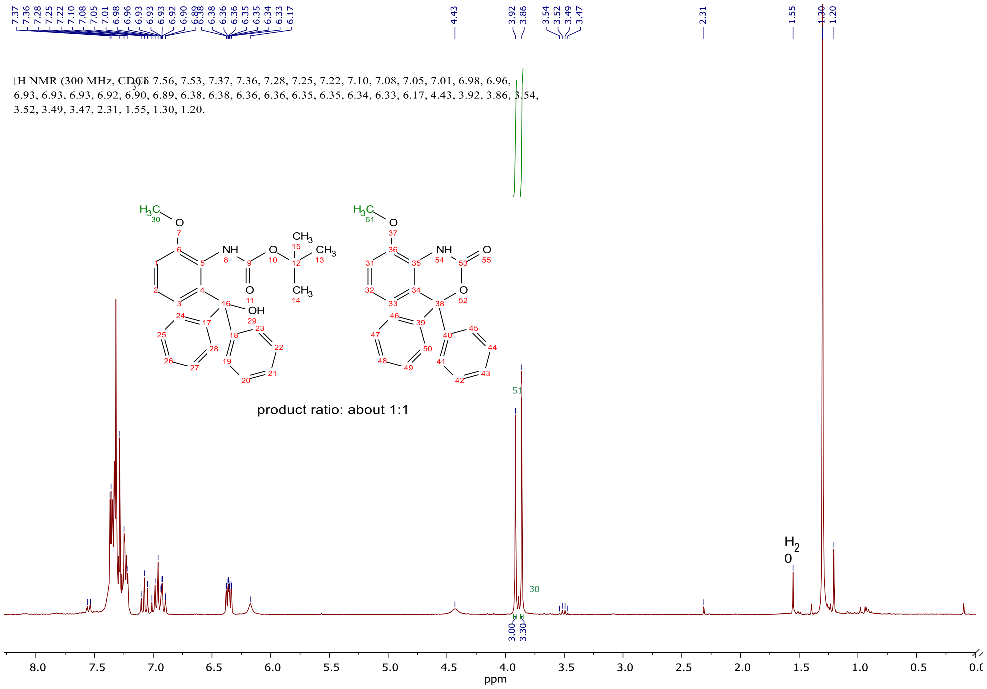

Figure S10. ^{1}H-NMR spectrum of **2** and **2a** in $CDCl_3$.

Mass spectrometry

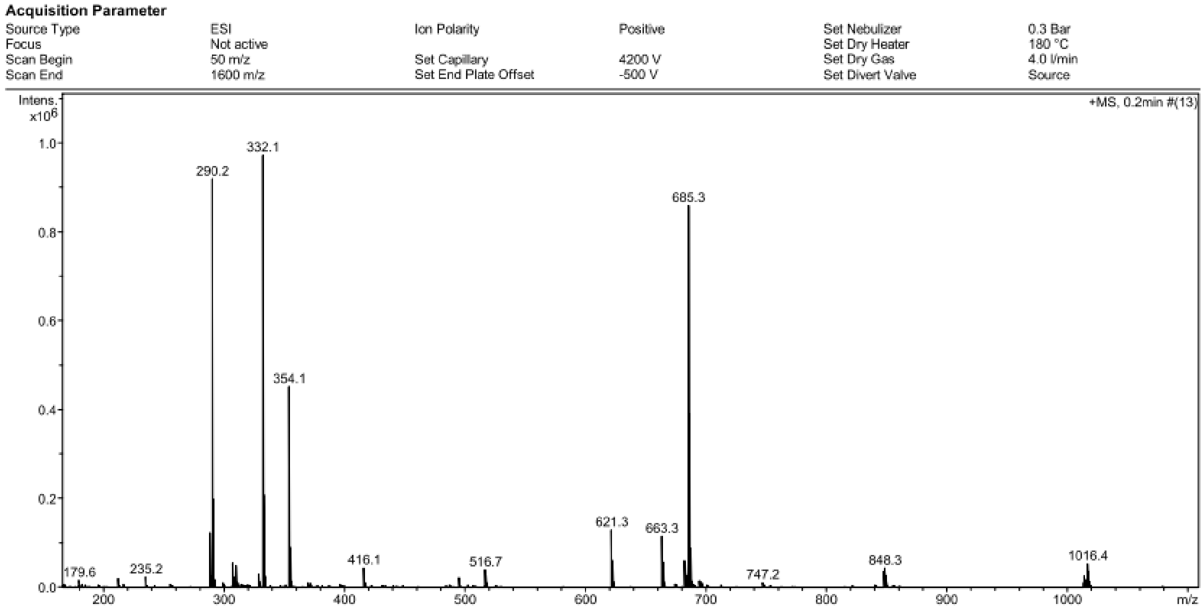

Figure S11. Mass spectrum of **2** and **2a** MS (ESI[+], THF) *m/z* (%): 685.3 (88) [2×(**2a**)+Na]$^+$, 663.3 (12) [2×(**2a**)+H]$^+$, 354.1 (46) [(**2a**)+Na]$^+$, 332.1 (100) [(**2a)**]$^+$, 290.2 (94) [(**2**)]$^+$.

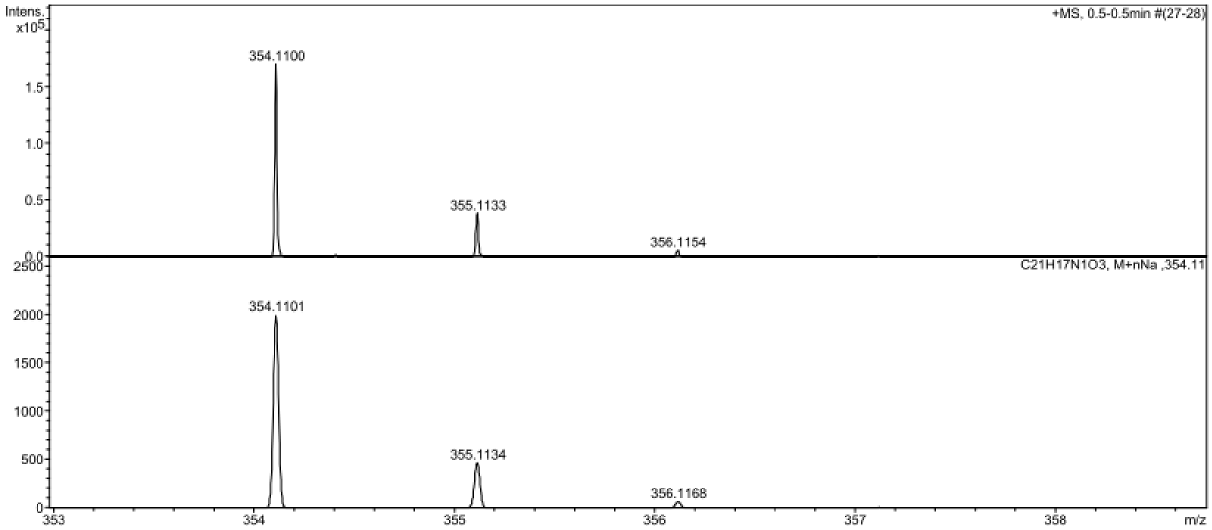

Figure S12. Mass spectrum HR-MS (ESI[+], THF) *m/z*: 354.1100 (cal. 354.1101 for [(**2a**)+Na]$^+$.

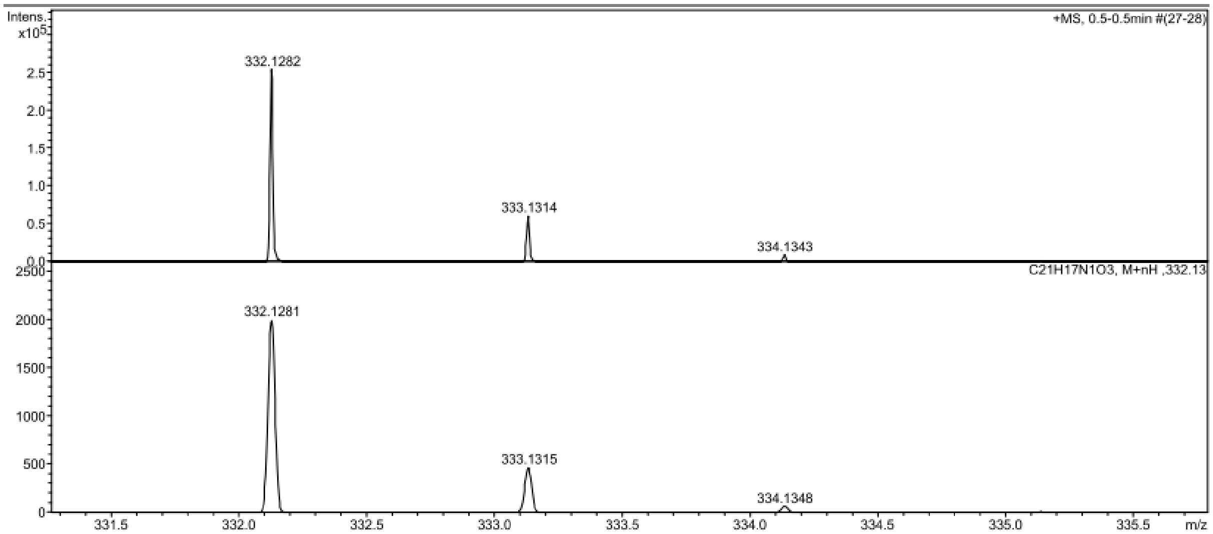

Figure S13. Mass spectrum HR-MS (ESI[+], THF) *m/z*: 332.1282 (cal. 332.1281 for [(**2a**)+H]$^+$, $C_{21}H_{18}NO_3$).

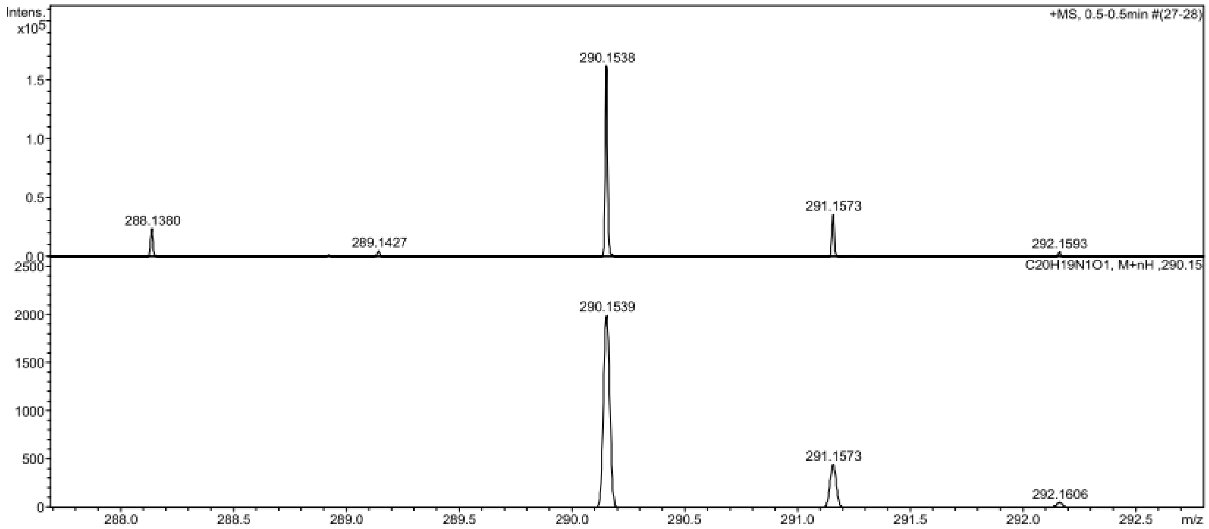

Figure S14. Mass spectrum HR-MS (ESI[+], THF) *m/z*: 290.1538 (cal. 290.1539 for [(**2**)-Boc-OH+H]$^+$, $C_{12}H_{20}NO$).

Compound 3: Synthesis and Analytical Data

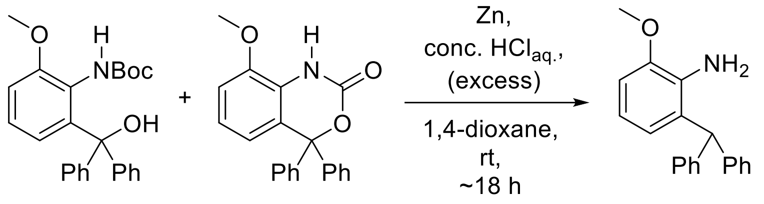

Alternative Synthesis of 2-Benzhydryl-6-methoxyaniline (3): A mixture of **2** (10.65 g, 26.26 mmol, 1.00 eq.) and **2a** (16.67 g, 50.31 mmol, 1.92 eq.) were dissolved in 1,4-dioxane (300 mL). Zinc powder (150 g, 2.30 mol, 30.0 eq.) was added to the yellow solution. Afterwards a concentrated hydrochloric acid (260 mL) was slowly added dropwise to the vigorously stirred suspension by a dropping funnel. The mixture was stirred overnight (~18 h) until gas evolution has ceased. The reaction mixture was adjusted to pH > 7 by aqueous sodium hydroxide solution. Received white suspension was filled in a separation funnel and extracted with Et_2O (4 × 200 mL). The organic phase was dried over $MgSO_4$, solvents were removed under reduced pressure and the obtained yellow oil was dried under fine vacuum (>3 h). Thereafter hexane (300 mL) was added to the oil and the mixture was ultrasonicated for 30 min. The obtained white precipitate was filtered out by a Büchner funnel, washed with pentane (4 × 200 mL) and dried under fine vacuum. Compound **3** was isolated as a white powder. Yield: 14.37 g (65%).

^{1}H NMR (300 MHz, $CDCl_3$, 298 K): δ = 7.39-7.32 (m, 4 H, 19-H, 21-H, 25-H, 27-H), 7.31-7.26 (m, 2 H, 20-H, 26-H), 7.21-7.18 (m, 4 H, 18-H, 22-H, 24-H, 28-H), 6.80 (dd, 1 H, $^3J_{HH}$ = 8.1 Hz, , $^4J_{HH}$ = 1.4 Hz, 5-H), 6.72 (dd, 1 H, $^3J_{HH}$ = 8.1 Hz, 4-H), 6.38 (ddd, 1 H, $^3J_{HH}$ = 8.1 Hz, $^4J_{HH}$ = 1.4 Hz, $^4J_{HH}$ = 0.5 Hz, 3-H), 5.58 (s, 1 H, 16-H), 3.90 (s, 3 H, $-OCH_3$), 3.74 (s_{br}, 2 H, $-NH_2$) ppm.

^{13}C{^{1}H} NMR (75 MHz, $CDCl_3$, 298 K): δ = 147.6 (6-C), 134.2 (1-C), 129.6 (18-C, 22-C, 24-C, 28-C), 129.5 (2-C), 19-H, 128.6 (19-C, 21-C, 25-C, 27-C),126.7 (20-C, 26-C), 122.3 (3-C), 117.6 (4-C), 108.6 (5-C), 55.7 ($-OCH_3$), 52.3 (16-C) ppm.

MS (ESI[+], THF) *m/z* (%): 290.2 (100) $[M+H]^+$, 312.1 (11) $[M+Na]^+$.

HR-MS (ESI[+], THF) *m/z*: 290.1542 (cal. 290.1539 for $[M+H]^+$, $C_{20}H_{19}NO$).

Elemental analysis in % (calculated) $C_{20}H_{19}NO$ (289.38 g/mol): C 82.77 (83.01), H 6.37 (6.62), N 4.67 (4.84).

NMR spectroscopy

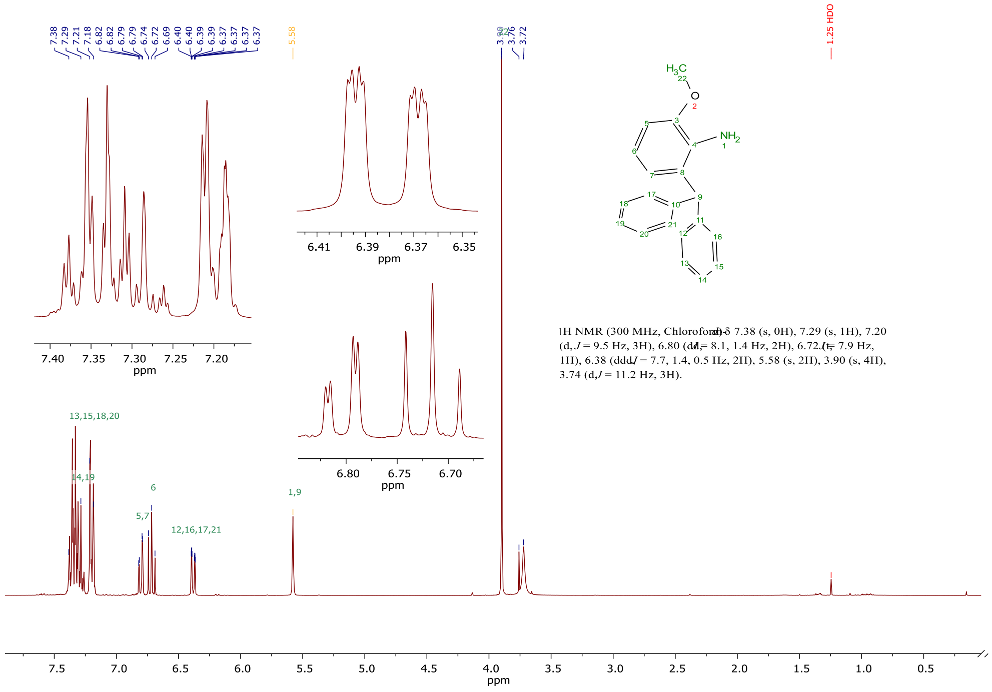

Figure S15. ^{1}H NMR spectrum of **3** in $CDCl_3$.

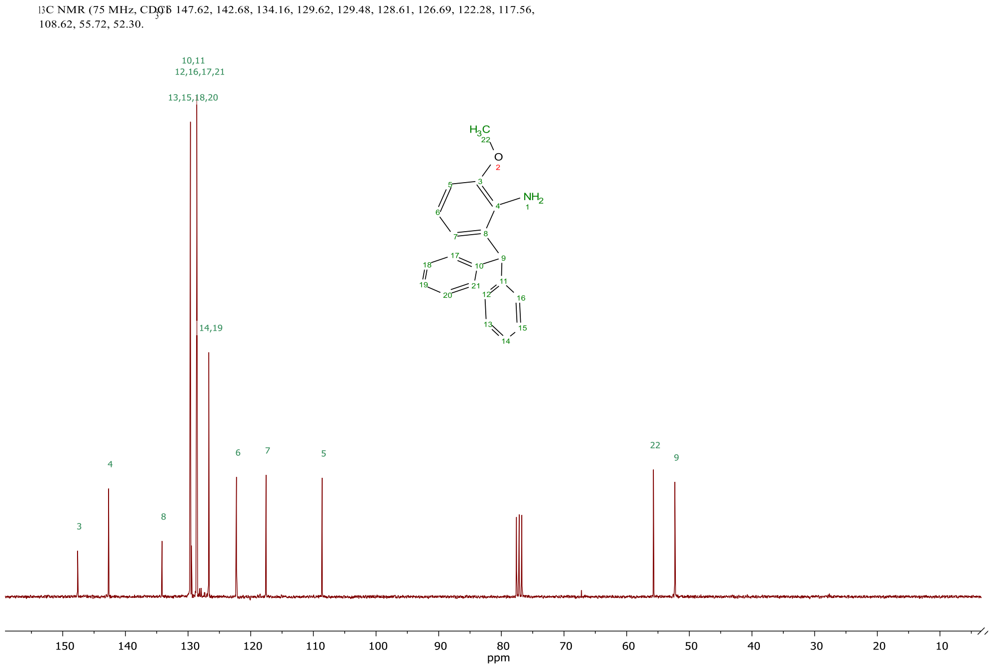

Figure S16. ^{13}C NMR spectrum of **3** in $CDCl_3$.

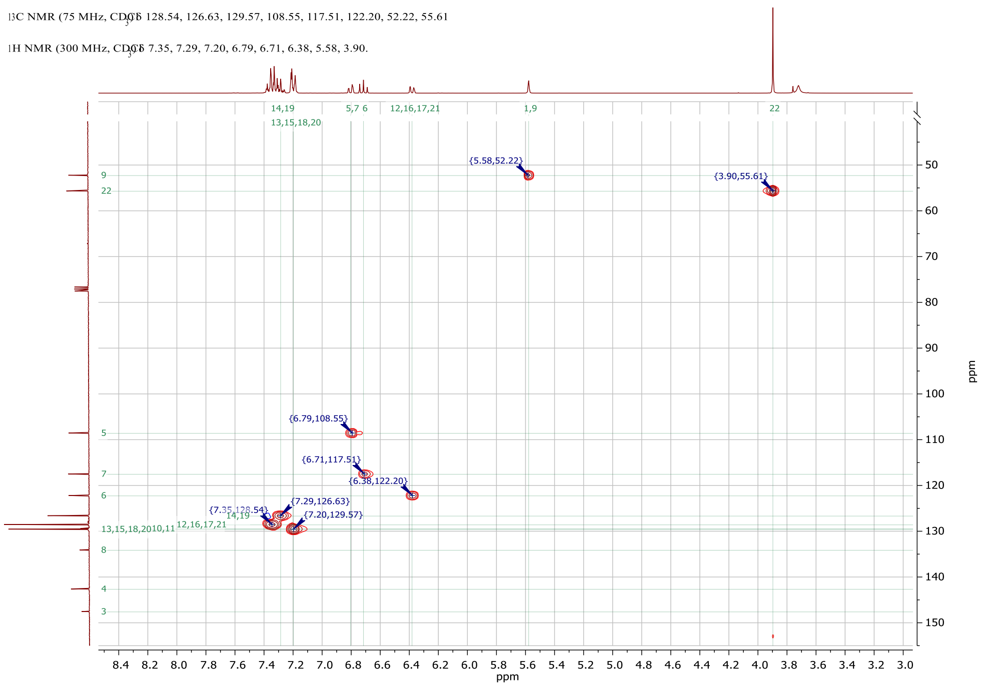

Figure S17. $^{1}H,^{13}C$ HSQC spectrum of **3** in $CDCl_3$.

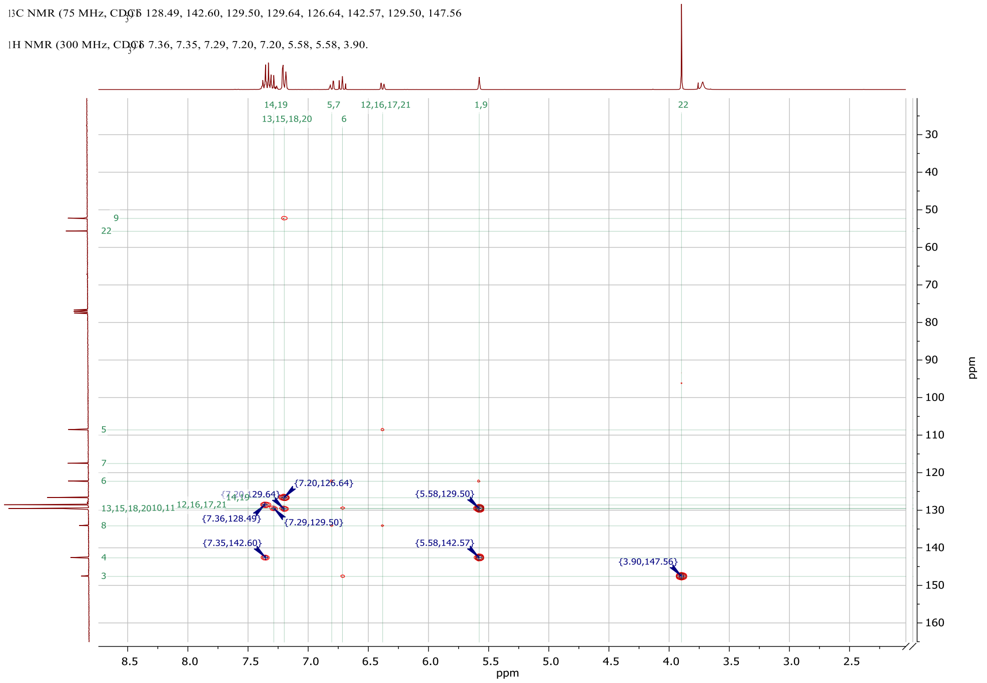

Figure S18. $^{1}H,^{13}C$ HMBC spectrum of **3** in $CDCl_3$.

Mass spectrometry

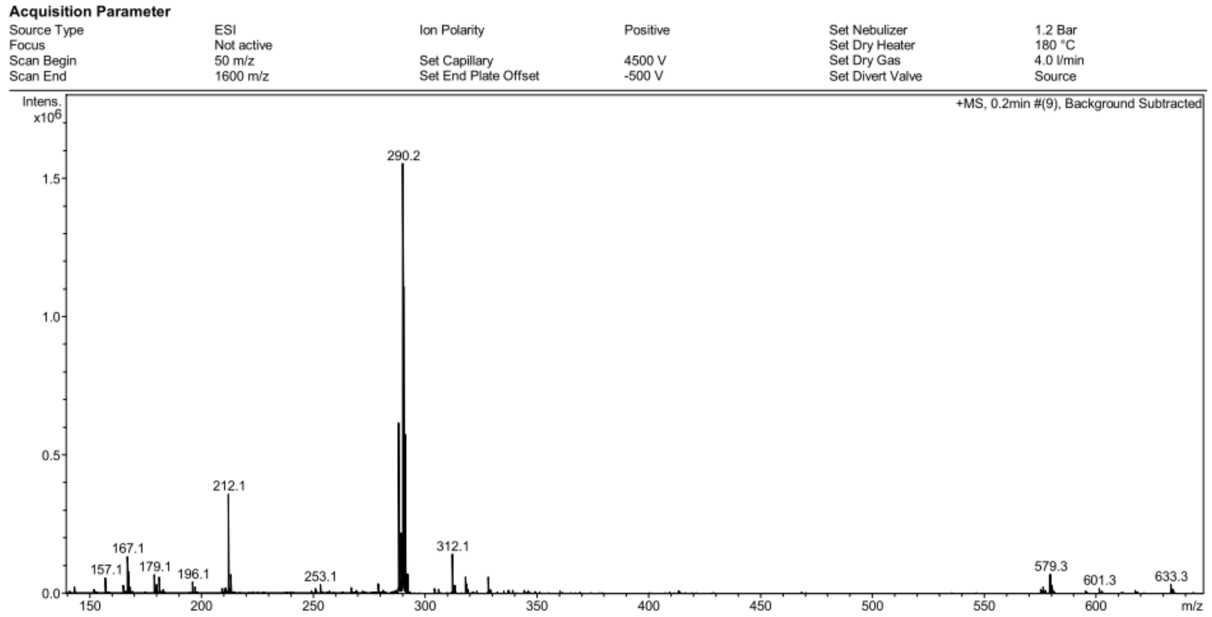

Figure S19. Mass spectrum of **3** (ESI[+], THF) *m/z* (%): 290.2 (100) [*M*+H]$^+$, 312.1 (11) [*M*+Na]$^+$.

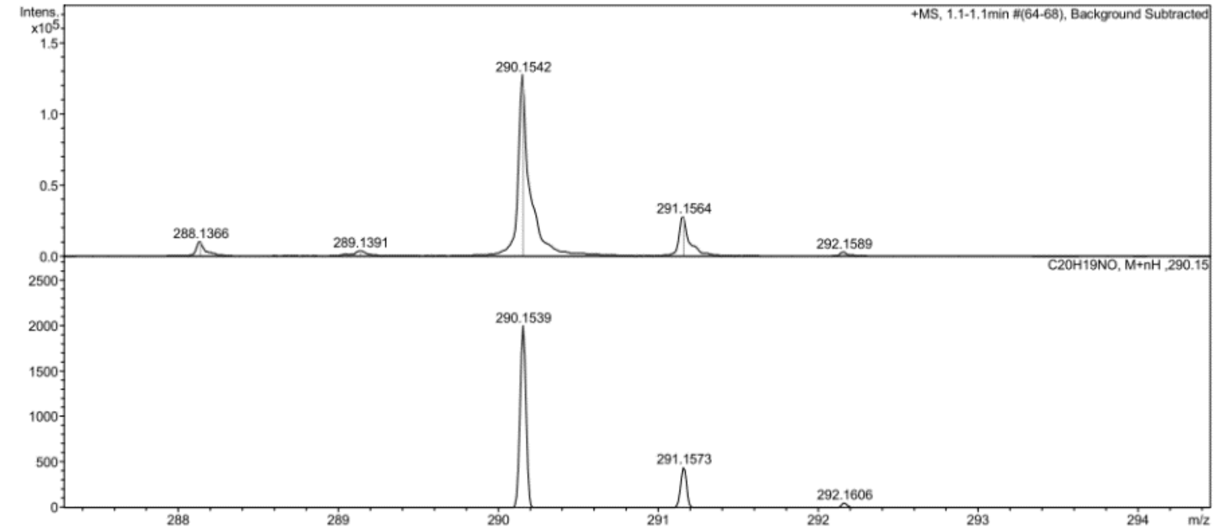

Figure S20. Mass spectrum HR-MS (ESI[+], THF) *m/z*: 290.1542 (cal. 290.1539 for [*M*+H]$^+$, $C_{20}H_{19}NO$).

Compound 4: Synthesis and Analytical Data

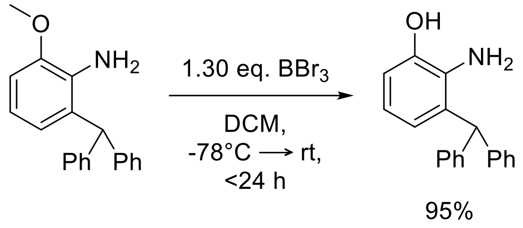

Alternative Synthesis of 2-Amino-3-benzhydrylphenol (4): 2-Benzhydryl-6-methoxyaniline (**3**) (8.32 g, 28.4 mmol, 1.00 eq.) was dissolved in DCM (450 mL). A solution of boron tribromide (9.27 g, 3.51 mL (2.64 g/mL), 37.0 mmol, 1.30 eq.) in DCM (37 mL), which was freshly prepared in a dropping funnel was carefully added at −78°C (EtOH/$CO_{2(s)}$). The olive-green solution was slowly warmed to ambient temperature overnight (~18 h) while stirring. To avoid the formation of by-products the reaction time should not exceed 1 d. Thereafter a saturated solution of sodium hydrogen carbonate in water (240 mL) was added dropwise to the reaction solution. At first the reaction mixture turns yellow and a white precipitate is formed then under gas formation is observed and the precipitate dissolves slowly. After stirring ~3 h the precipitate was completely dissolved (pH = 8-9) and two phases- a aqueous clear and a yellow organic phase- were observed. The organic phase was separated by a separation funnel and the aqueous phase was extracted with ethyl acetate (3 × 80 mL). Thereafter the organic phases were washed with demineralised water (3 × 50 mL) and dried with $MgSO_4$. Solvents were removed under reduced pressure and beige solid was obtained. After drying overnight, the solid was used without further purification. Yield: 7.49 g (95%).

^{1}H NMR (300 MHz, $CDCl_3$, 298 K): δ = 7.31-7.19 (m, 6 H, 19-H, 20-H, 21-H, 25-H, 26-H, 27-H), 7.13-7.09 (m, 4 H, 18-H, 22-H, 24-H, 28-H), 6.64 (dd, 1 H, $^3J_{HH}$ = 7.8 Hz, $^4J_{HH}$ = 1.4 Hz, 5-H), 6.56 (dd, 1 H, $^3J_{HH}$ = 7.8 Hz, 4-H), 6.27 (dd, 1 H, $^3J_{HH}$ = 7.6 Hz, $^4J_{HH}$ = 1.0 Hz, 3-H), 5.51 (s, 1 H, 16-H), 3.99 (s_{br}, 3 H, -NH_2, -OH) ppm.

^{13}C{^{1}H} NMR (75 MHz, $CDCl_3$, 298 K): δ = 144.5 (6-C), 142.6 (17-C, 23-C), 132.5 (1-C), 131.7 (2-C), 129.6 (18-C, 22-C, 24-C, 28-C), 128.7 (19-C, 21-C, 25-C, 27-C), 126.8 (20-C, 26-C), 122.6 (3-C), 118.8 (4-C), 113.5 (5-C), 52.4 (16-C) ppm.

MS (ESI[+], THF) *m/z* (%): 276.1 (100) $[M+H]^+$, 298.1 (3) $[M+Na]^+$.

HR-MS (ESI[+], THF) *m/z*: 276.1382 (cal. 276.1383 for $[M+H]^+$, $C_{19}H_{18}NO$), 298.1200 (cal. 298.1202 for $[M+Na]^+$, $C_{19}H_{17}NNaO$).

Elemental analysis in % (calculated) $C_{19}H_{17}NO$ (275.13 g/mol): C 82.87 (82.88), H 5.98 (6.22), N 4.90 (5.09).

NMR spectroscopy

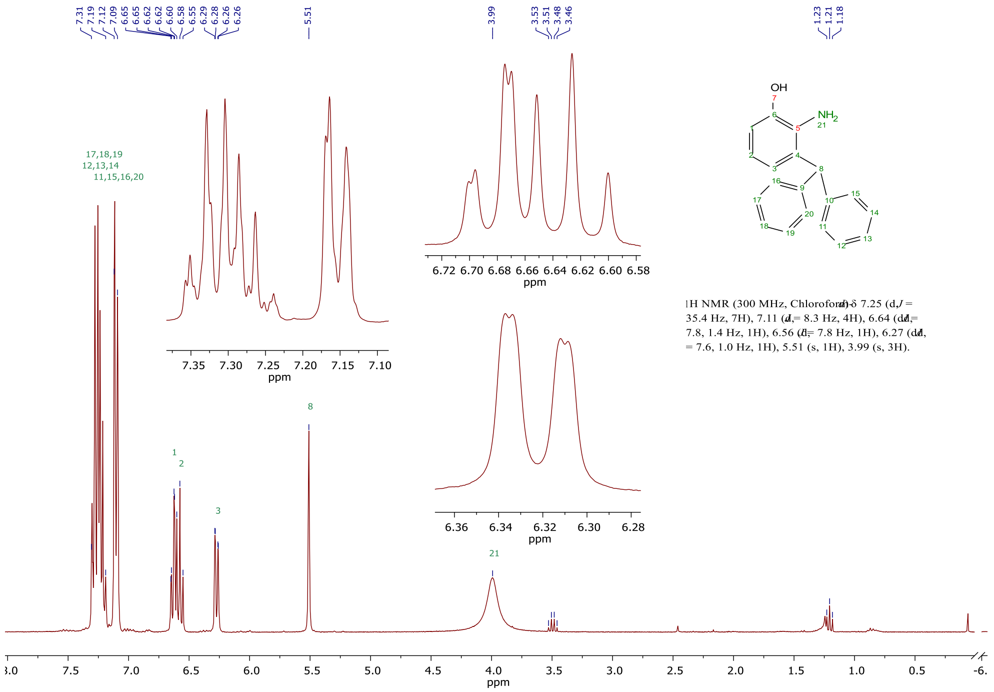

Figure S21. ^{1}H-NMR spectrum of **4** in $CDCl_3$.

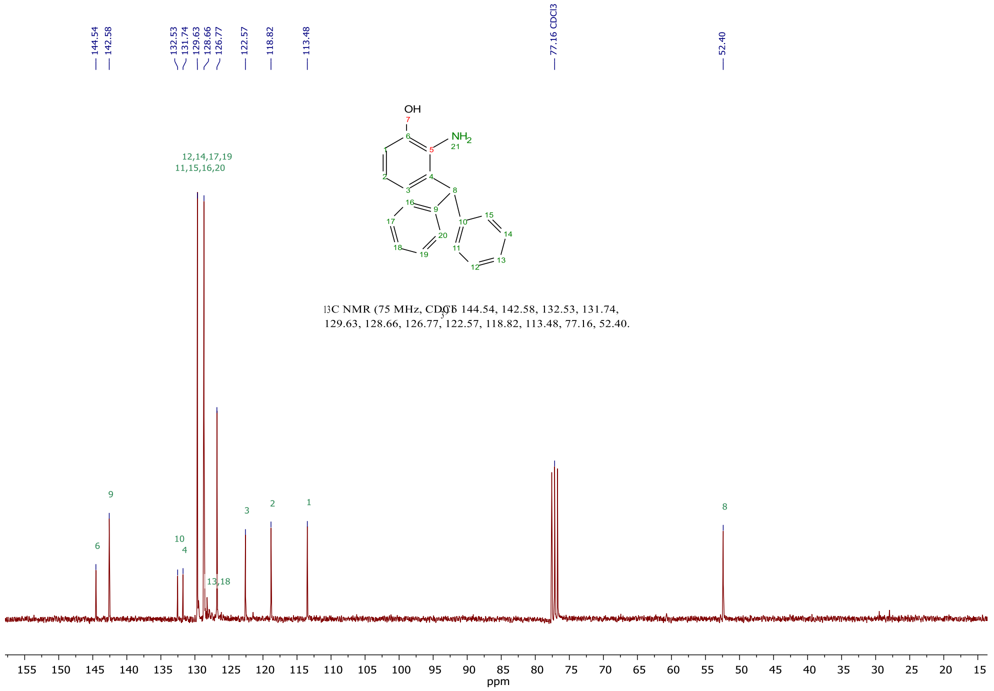

Figure S22. ^{13}C NMR spectrum of **4** in $CDCl_3$.

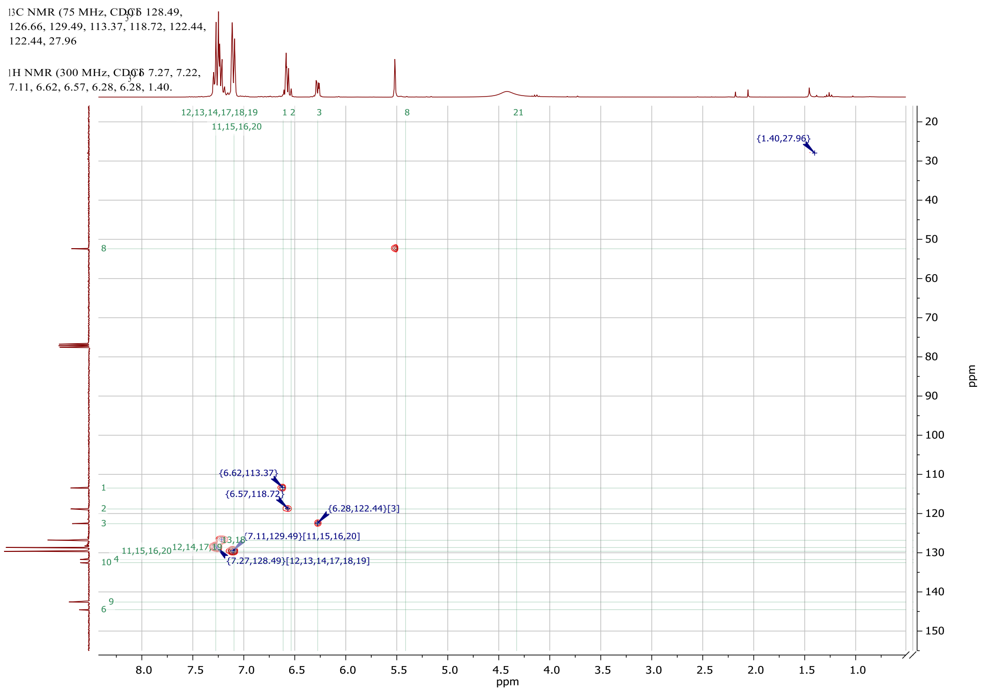

Figure S23. $^{1}H,^{13}C$ HSQC spectrum of **4** in $CDCl_3$.

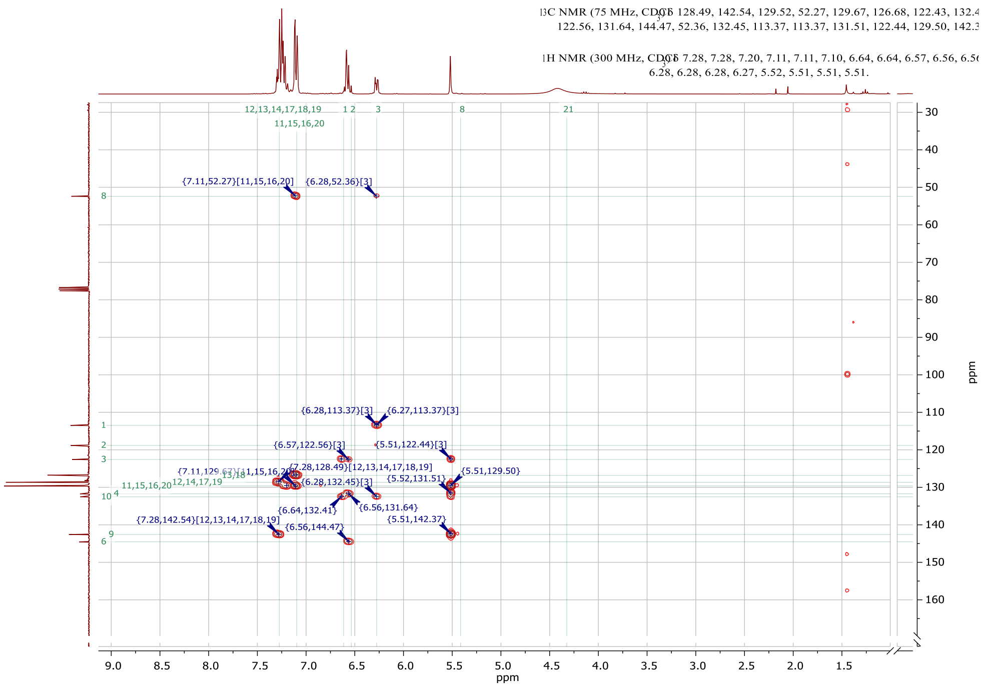

Figure S24. $^{1}H,^{13}C$ HMBC spectrum of **4** in $CDCl_3$.

Mass spectrometry

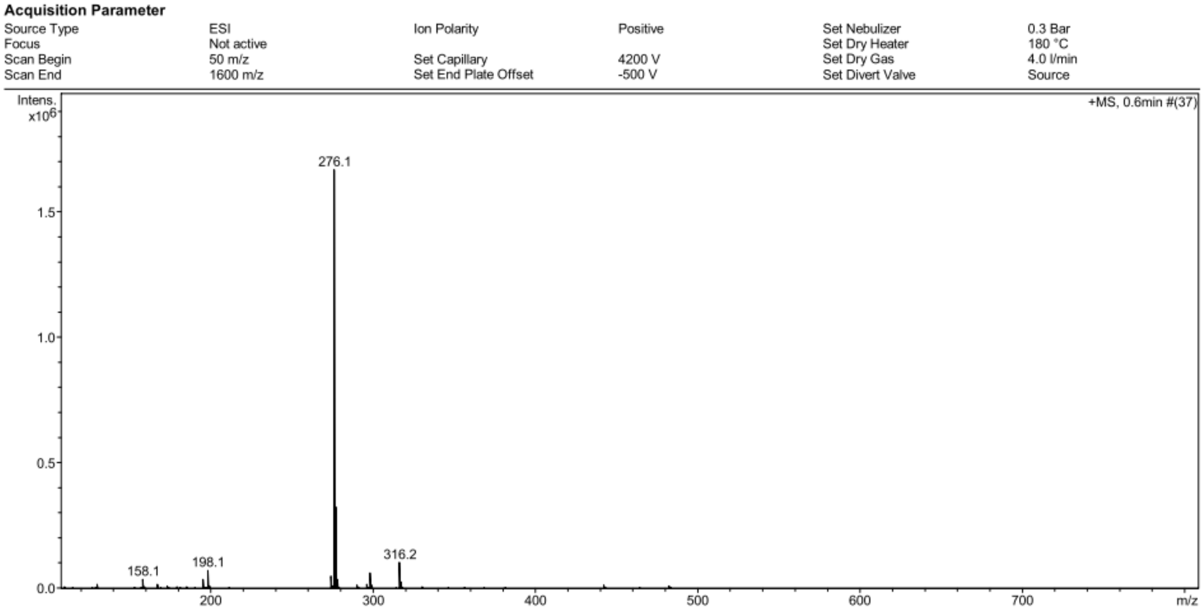

Figure S25. Mass spectrum of **4** (ESI[+], THF) *m/z* (%): 276.1 (100) [*M*+H]$^+$, 298.1 (3) [*M*+Na]$^+$.

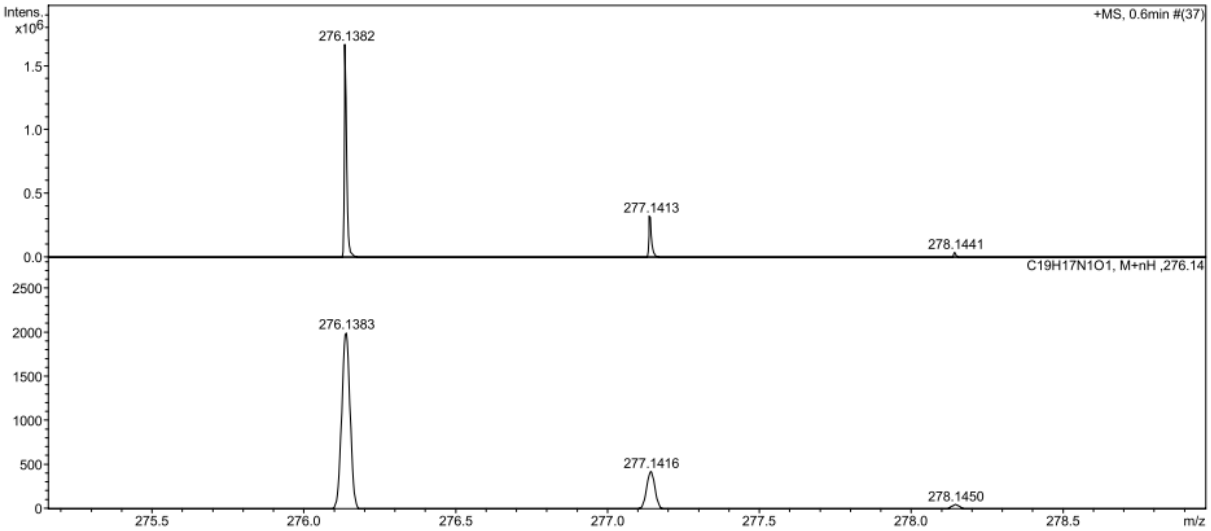

Figure S26. Mass Spectrum HR-MS (ESI[+], THF) *m/z*: 276.1382 (cal. 276.1383 for [*M*+H]$^+$, $C_{19}H_{18}NO$).

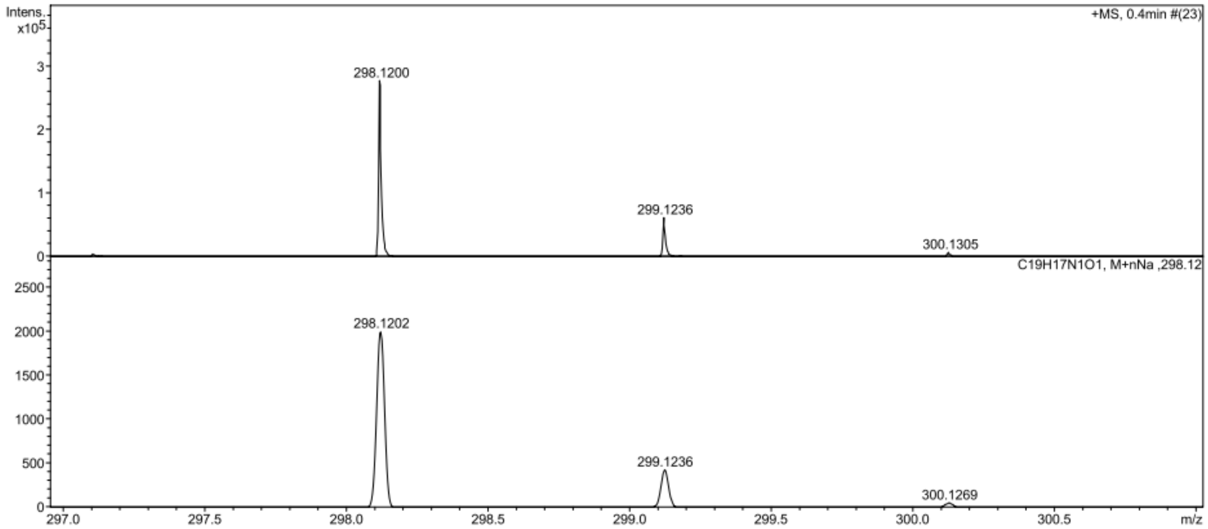

Figure S27. Mass spectrum HR-MS (ESI[+], THF) *m/z*: 298.1200 (cal. 298.1202 for $[M{+}Na]^+$, $C_{19}H_{17}NNaO$).

Compound 5: Synthesis and Analytical Data

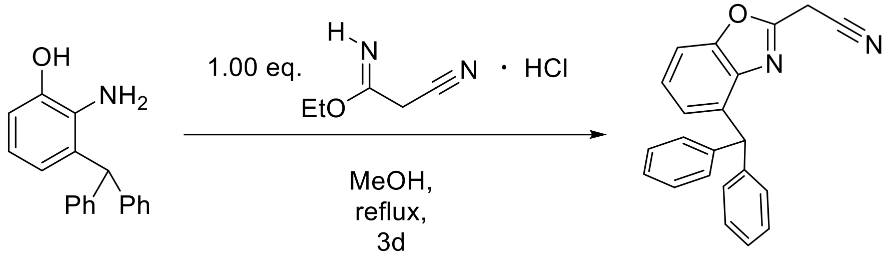

Synthesis of 2-(4-benzhydrylbenzoxazol-2-yl)-acetonitrile (5): 2-Amino-3-benzhydrylphenol (**4**) (6.29 g, 22.8 mmol, 1.00 eq.) and ethyl cyanoacetimidate hydrochloride (3.39 g, 22.8 mmol, 1.00 eq.) were weighed into a Schlenk flask (50 mL). Thereafter anhydrous MeOH (32 mL) was added and the obtained white suspension was heated at 85°C for 3 d, while ethyl cyanoacetimidate hydrochloride was dissolved at ~60°C. Overnight a white precipitate was formed in the reaction mixture. After the mixture was refluxed for two more days the flask was cooled to room temperature and stored for one night at −30°C. The precipitate was filtered by a Buchner funnel and washed with a saturated solution of sodium hydrogen carbonate in water (3 × 12 mL) and demineralised water (3 × 12 mL). Thereafter the white compound was filled in an even number of centrifuge tubes, MeOH (~8 mL) was added until the tubes were balanced and obtained suspensions were ultrasonicated for at least 15 min. Afterwards the tubes were placed in a centrifuge, rotated at 2000 rpm for 4 min, whereupon solvents were decanted. This procedure was repeated two times. The white solid was dried under reduced pressure overnight and was used for analysis without further purification. Yield: 2.82 g (38%).

^{1}H NMR (300 MHz, $CDCl_3$, 298 K): δ = 7.38 (dd, 1 H, $^3J_{HH}$ = 8.2 Hz, $^4J_{HH}$ = 0.9 Hz, 5-H), 7.29-7.24 (dd, 1 H, $^3J_{HH}$ = 7.9 Hz, $^4J_{HH}$ = 0.9 Hz, 4-H), 7.24-7.18 (m, 4 H, 19-H, 21-H, 25-H, 27-H), 7.18 (m, 2 H, 20-H, 26-H), 7.13-7.09 (m, 4 H, 18-H, 22-H, 24-H, 28-H), 6.97 (dd, 1 H, $^3J_{HH}$ = 7.6 Hz, $^4J_{HH}$ = 0.8 Hz, 3-H), 6.21 (s, 1 H, 16-H), 3.96 (s, 2 H, 8-H) ppm.

^{13}C{^{1}H} NMR (75 MHz, $CDCl_3$, 298 K): δ = 154.7 (7-C), 151.2 (6-C), 142.9 (17-C, 23-C), 139.9 (1-C), 129.4 (18-C, 22-C, 24-C, 28-C), 128.5 (19-C, 21-C, 25-C, 27-C), 126.6 (20-C, 26-C), 125.9 (4-C), 125.5 (3-C), 113.2 (9-C), 109.1 (5-C), 51.3 (16-C), 18.9 (8-C) ppm.

MS (ESI[+], THF) *m/z* (%): 325.1 (47) $[M+H]^+$, 347.1 (100) $[M+Na]^+$.

HR-MS (ESI[+], THF) *m/z*: 325.1333 (cal. 325.1335 for $[M+H]^+$, $C_{22}H_{17}N_2O$), 347.1155 (cal. 347.1155 for $[M+Na]^+$, $C_{22}H_{16}N_2NaO$).

Elemental analysis in % (calculated) $C_{19}H_{17}NO$ (324.38 g/mol): C 81.38 (81.46), H 4.95 (4.97), N 8.64 (8.64).

NMR spectroscopy

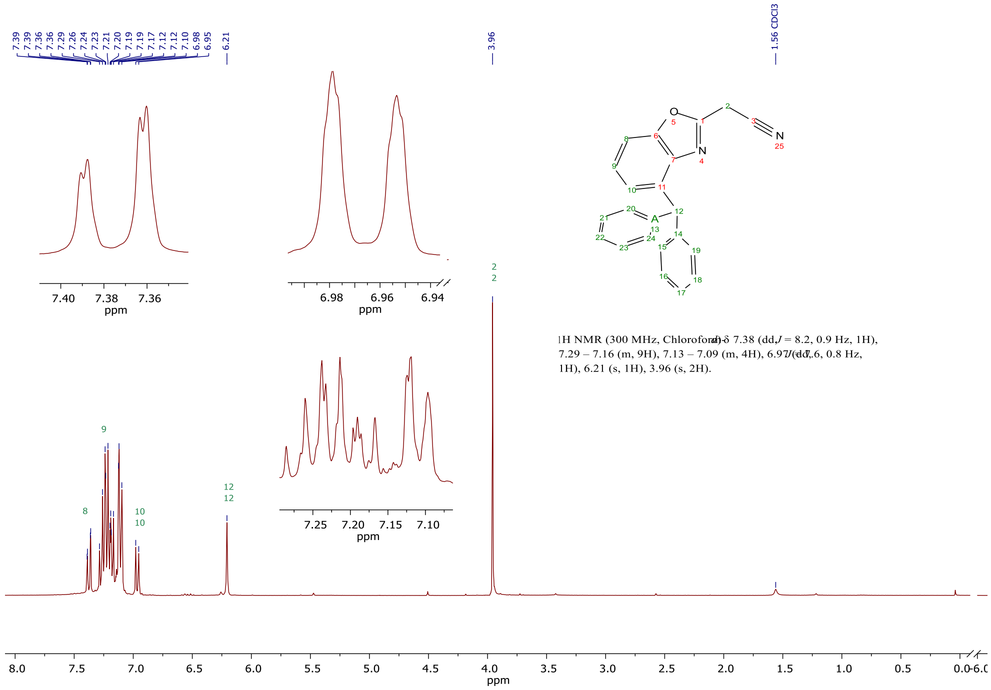

Figure S28. ^{1}H-NMR spectrum of **5** in $CDCl_3$.

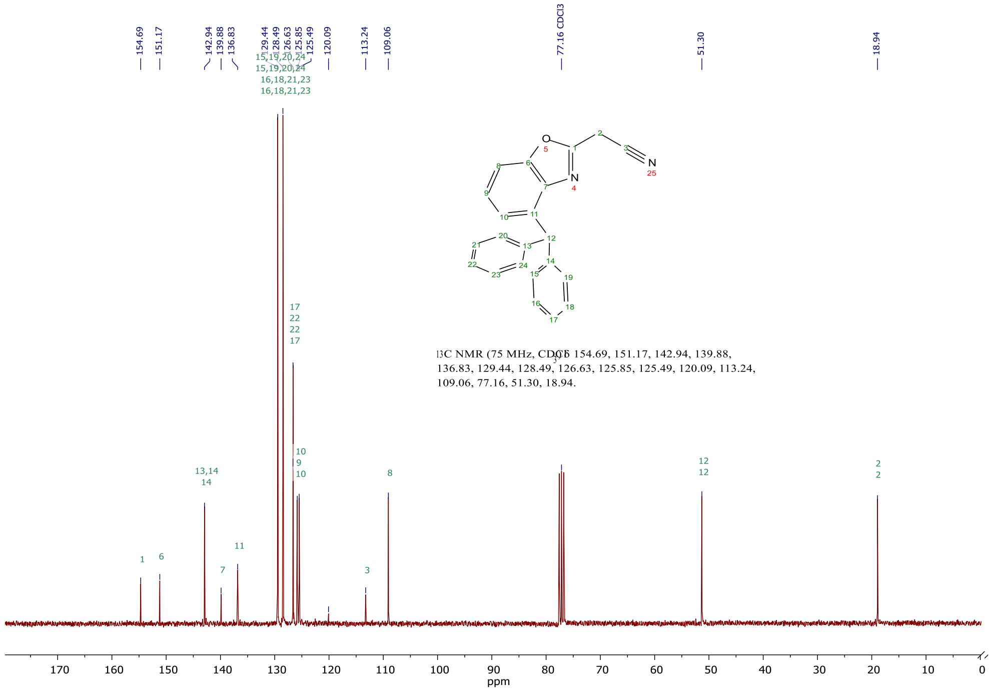

Figure S29. ^{13}C NMR spectrum of **5** in $CDCl_3$.

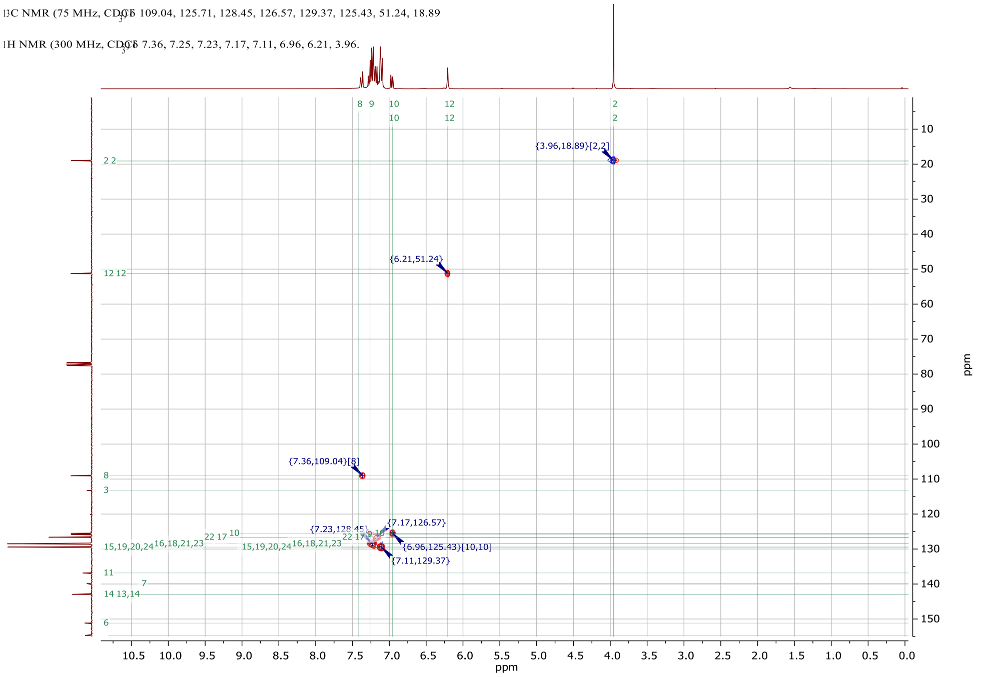

Figure S30. $^{1}H,^{13}C$ HSQC spectrum of **5** in $CDCl_3$.

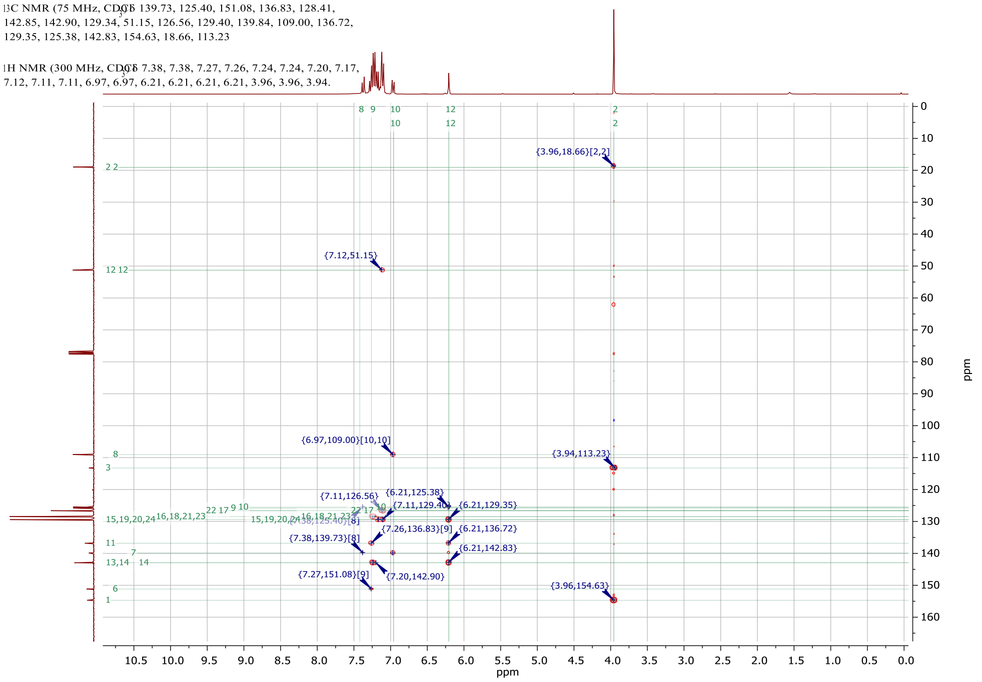

Figure S31. $^{1}H,^{13}C$ HMBC spectrum of **5** in $CDCl_3$.

Mass spectrometry

Acquisition Parameter

Source Type	ESI	Ion Polarity	Positive	Set Nebulizer	0.3 Bar
Focus	Not active			Set Dry Heater	180 °C
Scan Begin	50 m/z	Set Capillary	4200 V	Set Dry Gas	4.0 l/min
Scan End	1600 m/z	Set End Plate Offset	-500 V	Set Divert Valve	Source

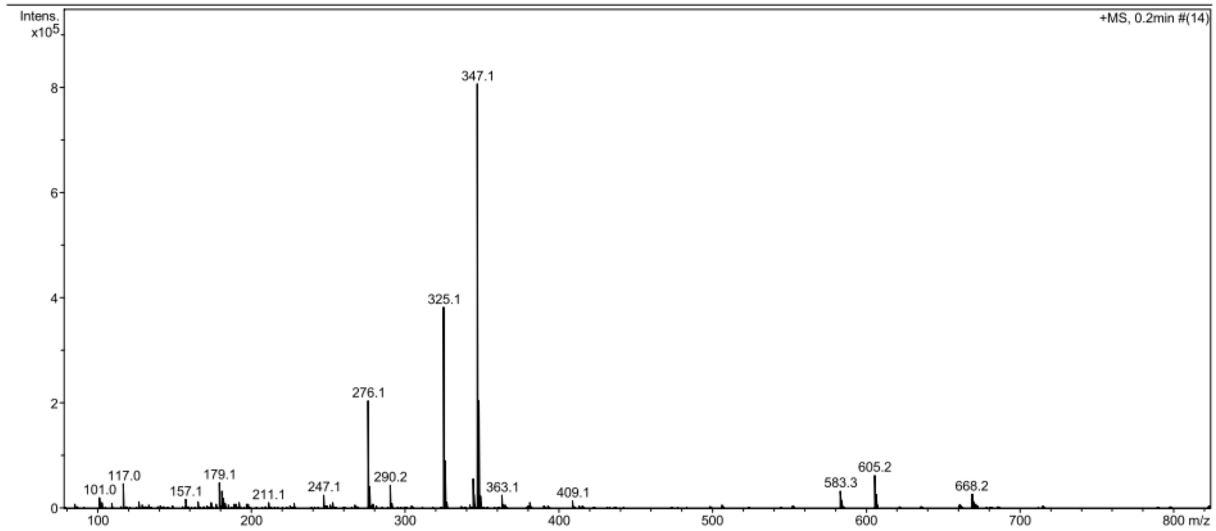

Figure S32. Mass spectrum of **5** (ESI[+], THF) *m/z* (%): 325.1 (47) [*M*+H]$^+$, 347.1 (100) [*M*+Na]$^+$.

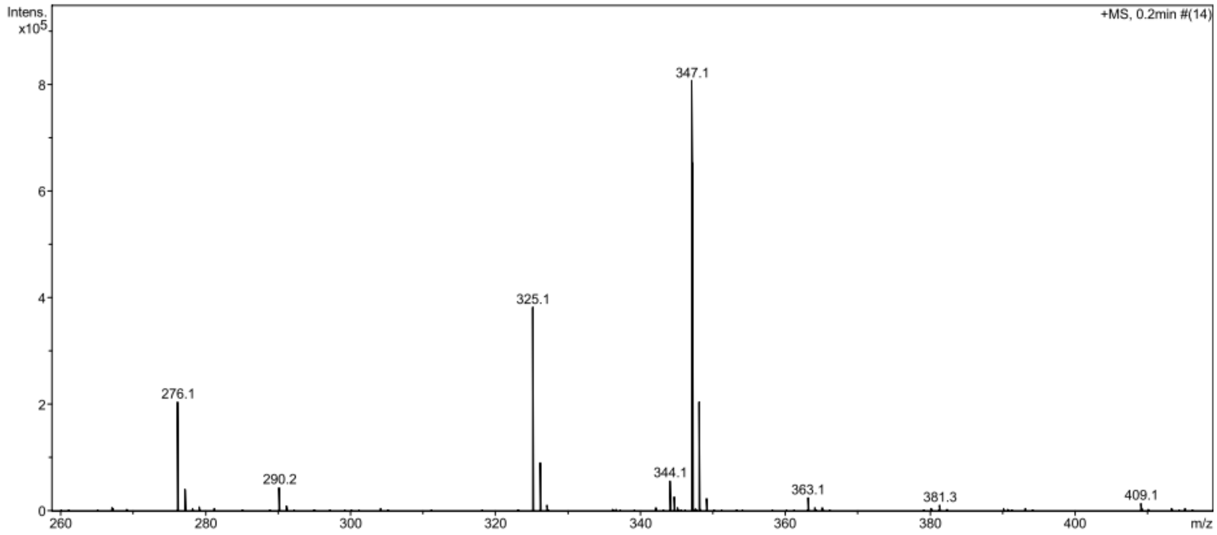

Figure S33. Mass spectrum of **5** (ESI[+], THF) *m/z* (%): 325.1 (47) [*M*+H]$^+$, 347.1 (100) [*M*+Na]$^+$.

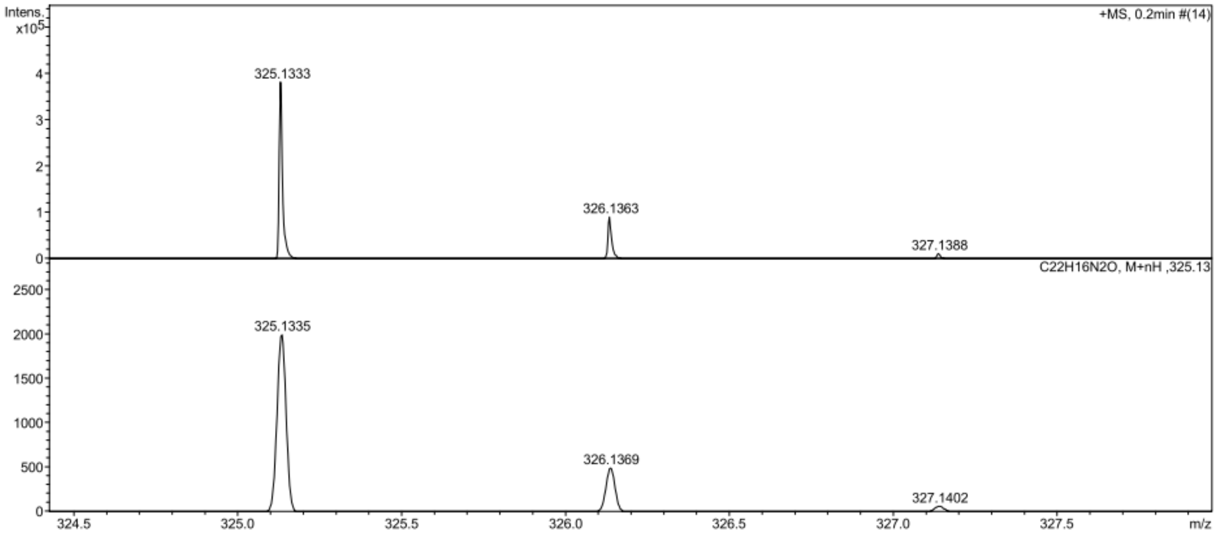

Figure S34. Mass spectrum HR-MS (ESI[+], THF) *m/z*: 325.1333 (cal. 325.1335 for $[M+H]^+$, $C_{22}H_{17}N_2O$).

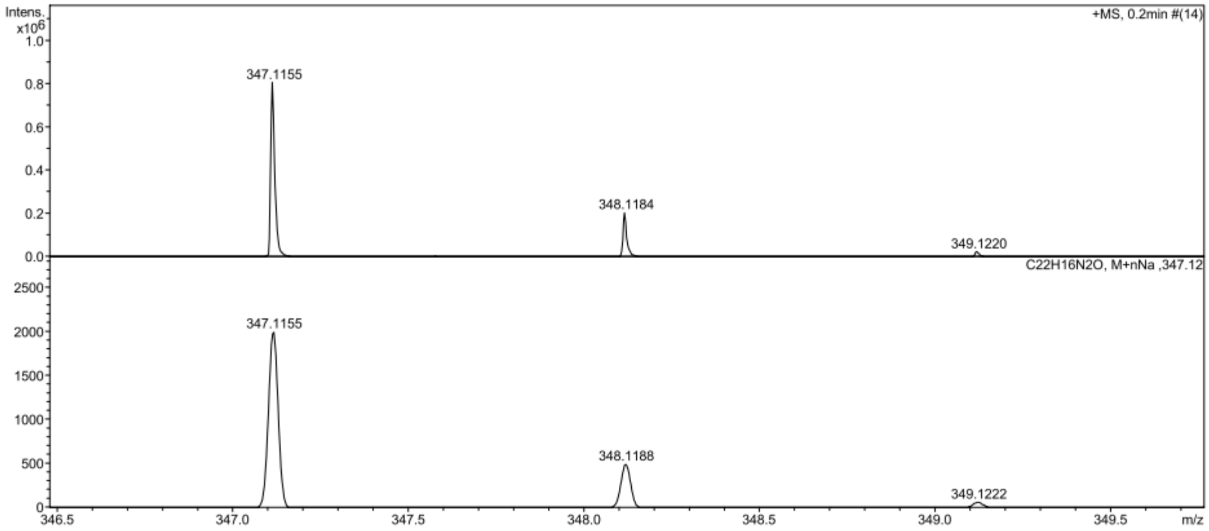

Figure S35. Mass spectrum HR-MS (ESI[+], THF) *m/z*: 347.1155 (cal. 347.1155 for $[M+Na]^+$, $C_{22}H_{16}N_2NaO$).

Compound 6: Synthesis and Analytical Data

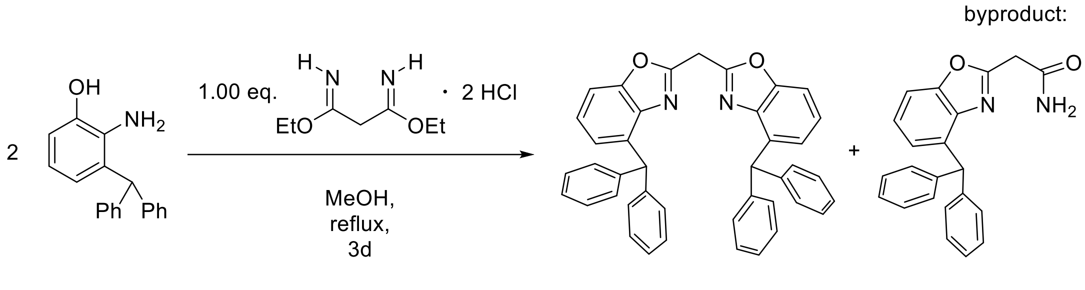

36% 13%

Synthesis of bis(4-benzhydryl-benzoxazol-2-yl)methane (6): 2-Amino-3-benzhydrylphenol (**4**) (15.0 g, 54.5 mmol, 2.00 eq.) and ethylbisimidate dihydrochloride (6.30 g, 27.2 mmol, 1.00 eq.) were weighed into a Schlenk flask (250 mL). Subsequently anhydrous MeOH (75 mL) was added and the obtained white suspension was heated at 85°C for at least 3 d, while ethyl ethylbisimidate dihydrochloride was dissolved at ~60°C. Overnight a white precipitate was formed in the reaction mixture. After the mixture was refluxed for at least two more days the flask was cooled to room temperature and stored for one night at −30°C. The precipitate was filtered by a Buchner funnel and washed with a saturated solution of sodium hydrogen carbonate in water (3 × 30 mL) and demineralised water (3 × 30 mL). Thereafter the white compound was filled in an even number of centrifuge tubes, MeOH (~10 mL) was added until the tubes were balanced and obtained suspensions were ultrasonicated for at least 15 min. Afterwards the tubes were placed in a centrifuge, rotated at 2000 rpm for 4 min, whereupon solvents were decanted. This procedure was repeated two times. The white solid was dried under reduced pressure and purified by column chromatography on silica gel (THF/hexane 3:2; R_f = 0.9; byproduct R_f = 0.48). Yield: 5.72 g (36%).

^{1}H NMR (400 MHz, $CDCl_3$, 298 K): δ = 7.40 (d, 2 H, $^3J_{HH}$ = 8.1 Hz, 5-H, 11-H), 7.34-7.21 (m, 22 H, 4-H, 12-H, 18-H, 19-H, 20-H, 21-H, 22-H, 24-H, 25-H, 26-H, 27-H, 28-H, 31-H, 32-H, 33-H, 34-H, 35-H, 37-H, 38-H, 39-H, 40-H, 41-H), 7.04 (d, 2 H, $^3J_{HH}$ = 7.6 Hz, 3-H, 13-H), 6.36 (s, 2 H, 16-H, 29-H), 4.61 (s, 2 H, 8-H) ppm.

^{1}H NMR (400 MHz, [D$_8$]THF, 298 K): δ = 7.35 (dd, 2 H, $^3J_{HH}$ = 8.3 Hz , $^4J_{HH}$ = 0.8 Hz, 5-H, 11-H), 7.22-7.16 (m, 10 H, $^3J_{HH}$ = 8.1 Hz, 4-H, 12-H,19-H, 21-H, 25-H, 27-H, 32-H, 34-H, 38-H, 40-H), 7.12-7.10 (m, 12 H, 18-H, 20-H, 22-H, 24-H, 26-H, 28-H, 31-H, 33-H, 35-H, 37-H, 39-H, 41-H), 6.95 (d, 2 H, $^3J_{HH}$ = 7.6 Hz , $^4J_{HH}$ = 0.8 Hz, 3-H, 13-H), 6.25 (s, 2 H, 16-H, 29-H), 4.58 (s, 2 H, 8-H) ppm.

^{1}H NMR (400 MHz, [D$_8$]toluene, 298 K): δ = 7.14-7.10 (m, 8 H, 18-H, 20-H, 22-H, 24-H, 26-H, 28-H, 31-H, 33-H, 35-H, 37-H, 39-H, 41-H), 7.08-7.04 (m, 8 H, 19-H, 21-H, 25-H, 27-H, 32-H, 34-H, 38-H, 40-H), 7.02-7.00 (m, 4 H, 20-H, 26-H, 33-H, 39-H), 6.97-6.93 (m, 4 H, 3-H, 5-H, 11-H, 13-H), 6.89-6.85 (dd, 2 H, $^3J_{HH}$ = 7.8 Hz, 4-H, 12-H), 6.41 (s, 2 H, 16-H, 29-H), 3.85 (s, 2 H, 8-H) ppm.

^{13}C{^{1}H} NMR (75 MHz, $CDCl_3$, 298 K): δ = 159.35 (7-C, 9-C), 151.16 (6-C, 10-C), 143.28 (17-C, 23-C, 30-C, 36-C), 140.30 (1-C, 15-C), 136.41 (2-C, 14-C), 129.52 (18-C, 22-C, 24-C, 28-C, 31-C, 35-C, 37-C, 41-C), 128.41 (19-C, 21-C, 25-C, 27-C, 32-C, 34-C, 38-C, 40-C), 126.49 (20-C, 26-C, 33-C, 39-C), 125.11 (4-C, 12-C), 125.04 (3-C, 13-C), 108.90 (5-C, 11-C), 51.13 (16-C, 29-C), 29.73 (8-C) ppm.

^{13}C{^{1}H} NMR (75 MHz, [D$_8$]THF, 298 K): δ = 160.89 (7-C, 9-C), 152.13 (6-C, 10-C), 144.39 (17-C, 23-C, 30-C, 36-C), 141.45 (1-C, 15-C), 137.39 (2-C, 14-C), 130.24 (18-C, 22-C, 24-C, 28-C, 31-C, 35-C, 37-C, 41-C), 128.97 (19-C, 21-C, 25-C, 27-C, 32-C, 34-C, 38-C, 40-C), 127.01 (20-C, 26-C, 33-C, 39-C), 125.64 (4-C, 12-C), 125.57 (3-C, 13-C), 109.35 (5-C, 11-C), 52.16 (16-C, 29-C), 29.74 (8-C) ppm.

$^{13}C\{^1H\}$ NMR (75 MHz, [D$_8$]toluene, 298 K): δ = 159.67 (7-C, 9-C), 151.39 (6-C, 10-C), 143.69 (17-C, 23-C, 30-C, 36-C), 140.94 (1-C, 15-C), 137.12 (2-C, 14-C), 129.81 (18-C, 22-C, 24-C, 28-C, 31-C, 35-C, 37-C, 41-C), 128.49 (18-C, 22-C, 24-C, 28-C, 31-C, 35-C, 37-C, 41-C), 125.05 (4-C, 12-C), 125.01 (3-C, 13-C), 126.50 (20-C, 26-C, 33-C, 39-C), 108.71 (5-C, 11-C), 51.72 (16-C, 29-C), 28.91 (8-C) ppm.

MS (ESI[+], THF) *m/z* (%): 605.2 (14) [*M*+Na]$^+$, 583.2 (100) [*M*+H]$^+$.

HR-MS (ESI[+], THF) *m/z*: 605.2196 (cal. 605.2199 for [*M*+Na]$^+$, $C_{41}H_{30}N_2NaO_2$), 583.2381 (cal. 583.2380 for [*M*+H]$^+$, $C_{22}H_{17}N_2O_2$).

Elemental analysis in % (calculated) $C_{41}H_{30}N_2O_2$ (582.70 g/mol): C 84.70 (84.51), H 5.07 (5.19), N 4.65 (4.81).

2-(4-benzhydrylbenzoxazol-2-yl)acetimidic acid (byproduct):

^1H NMR (300 MHz, [D$_8$]toluene, 298 K): δ = 7.75 (s$_{br}$, 1 H, -N*H*), 7.29 (m, 4 H, 19-H, 21-H, 25-H, 27-H), 7.27 (d, 1 H, $^3J_{HH}$ = 7.2 Hz, 4-H), 6.18 (d, 1 H, $^3J_{HH}$ = 8.1 Hz, 5-H), 7.21 (m, 2 H, 20-H, 26-H), 7.13 (m, 4 H, 18-H, 22-H, 24-H, 28-H), 6.94 (d, 1 H, $^3J_{HH}$ = 7.6 Hz, 3-H), 6.18 (s, 1 H, 16-H), 3.85 (s, 2 H, 4-H) ppm.

$^{13}C\{^1H\}$ NMR (75 MHz, $CDCl_3$, 298 K): δ = 167.73 (9-C), 161.52 (7-C), 150.36 (6-C), 142.98 (17-C, 23-C,), 139.64 (1-C), 135.12 (1-C), 129.00 (18-C, 22-C, 24-C, 28-C), 128.42 (19-C, 21-C, 25-C, 27-C), 126.42 (20-C, 26-C), 124.76 (4-C), 124.26 (3-C), 108.78 (5-C), 50.69 (16-C), 35.93 (8-C) ppm.

MS (ESI[+], THF) *m/z* (%): 343.2 (73) [*M*+H]$^+$, 365.1 (100) [*M*+Na]$^+$.

HR-MS (ESI[+], THF) *m/z*: 343.1438 (cal. 343.1441 for [*M*+H]$^+$, $C_{22}H_{18}N_2O_2$), 365.1253 (cal. 365.1253 for [*M*+Na]$^+$, $C_{22}H_{16}N_2NaO_2$).

Elemental analysis in % (calculated) $C_{41}H_{30}N_2O_2$ (324.40 g/mol): C 76.80 (77.17), H 5.45 (5.30), N 7.85 (8.18).

NMR spectroscopy

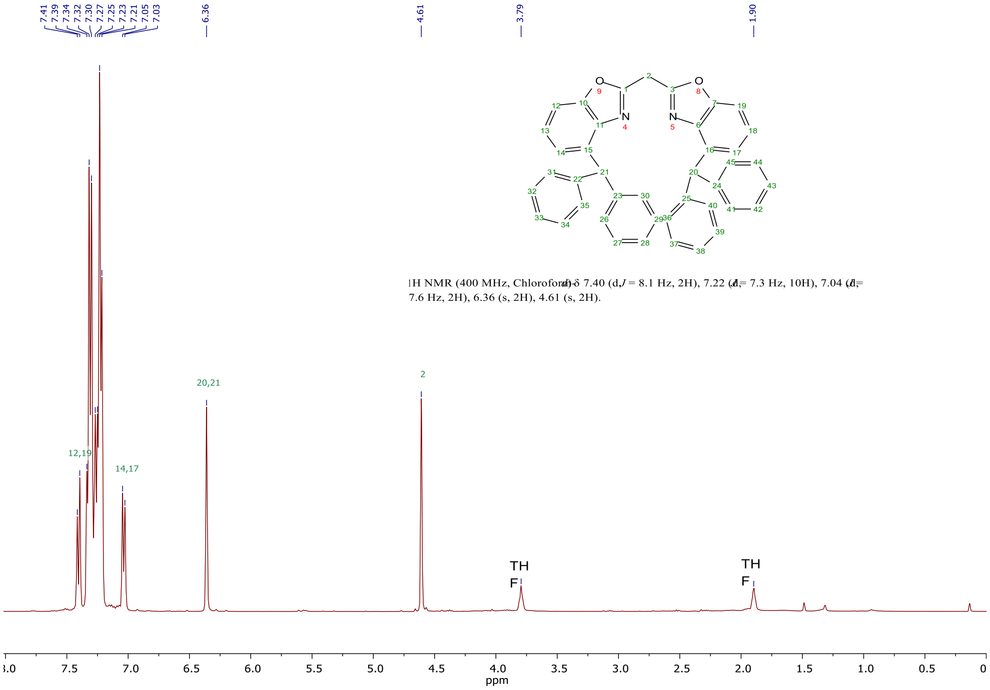

Figure S36. ^{1}H-NMR spectrum of **6** in $CDCl_3$. The residual solvent signal is marked with #.

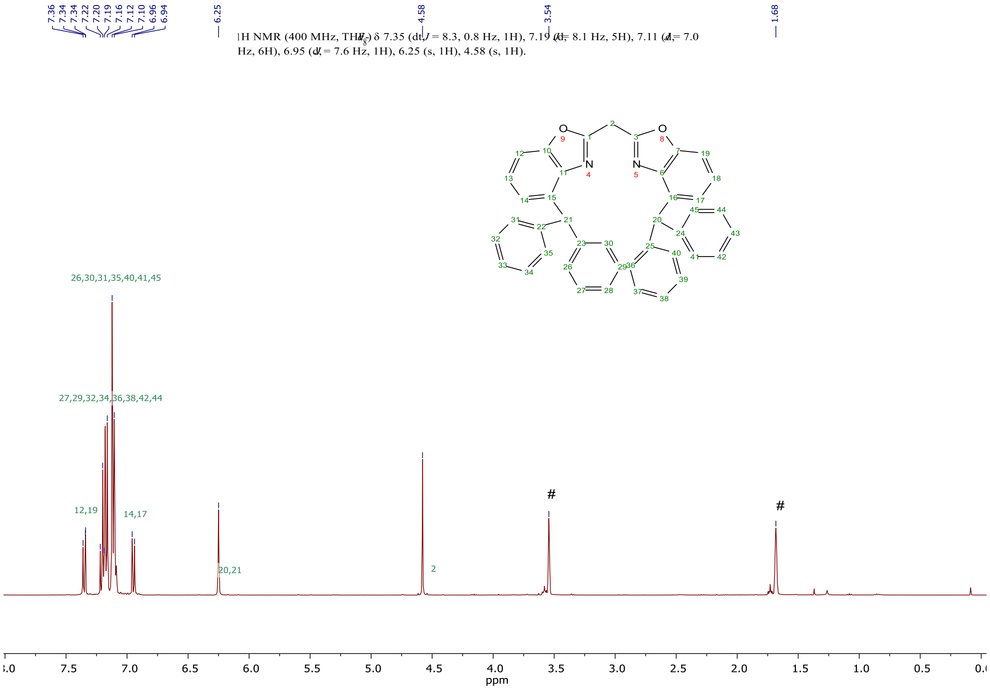

Figure S37. ^{1}H-NMR spectrum of **6** in $[D_8]$THF. The residual solvent signal is marked with #.

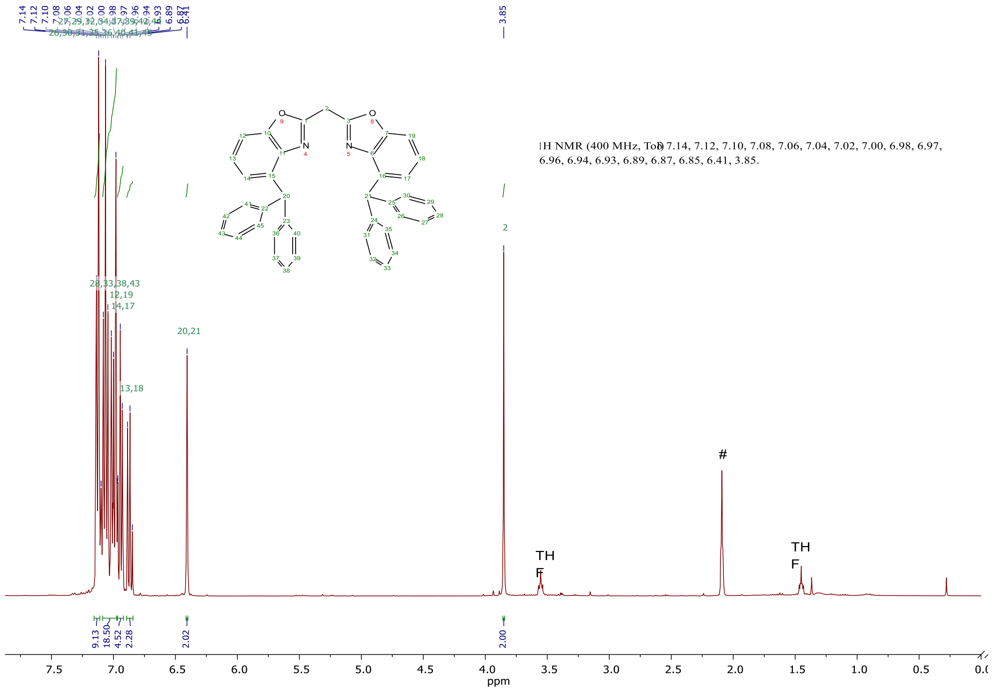

Figure S38. ^{1}H-NMR spectrum of **6** in [D$_8$]toluene. The residual solvent signal is marked with #.

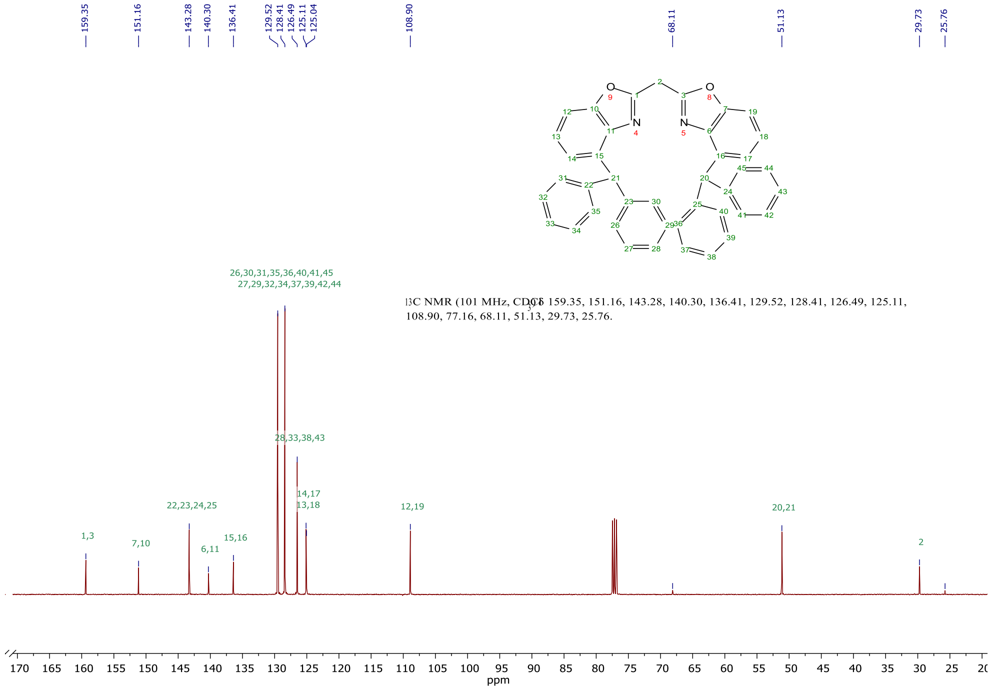

Figure S39. ^{13}C NMR spectrum of **6** in CDCl$_3$.

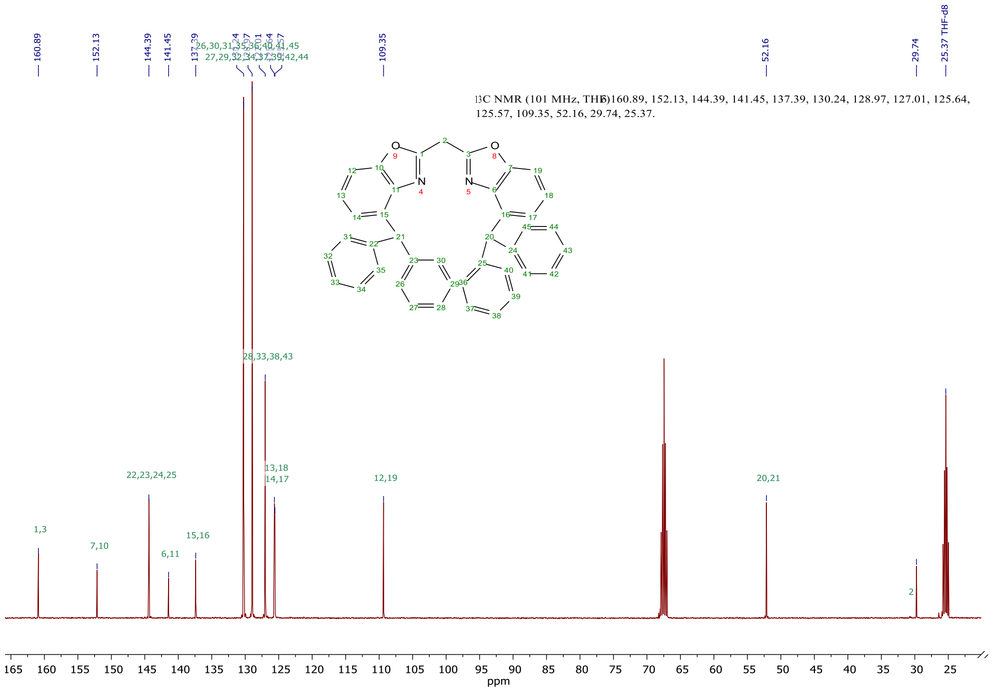

Figure S40. ^{13}C NMR spectrum of **6** in [D$_8$]THF.

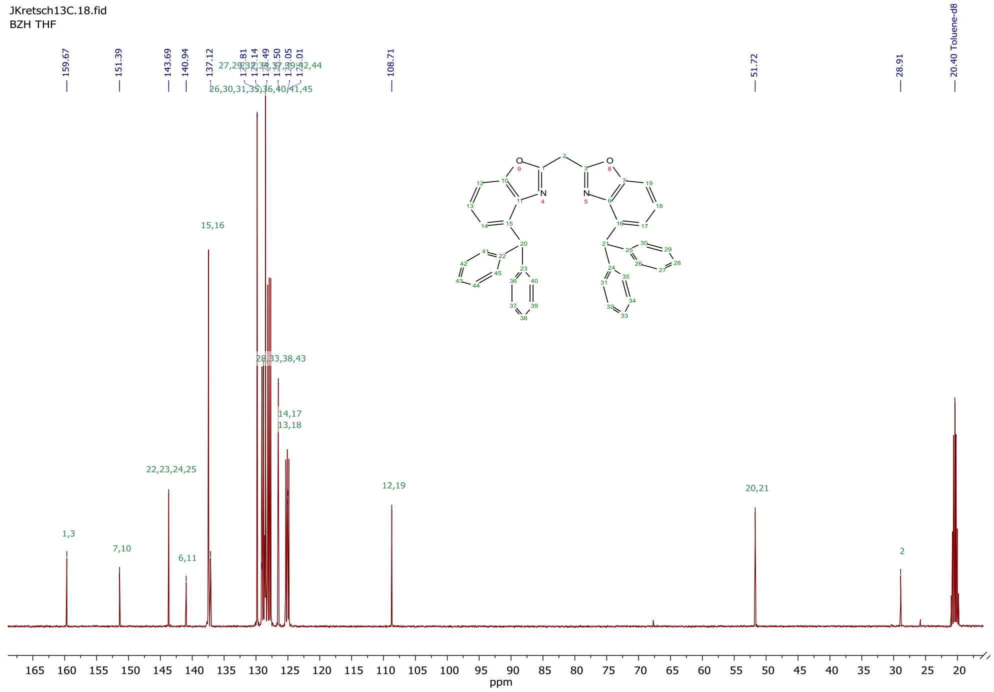

Figure S41. ^{13}C NMR spectrum of **6** in [D$_8$]toluene.

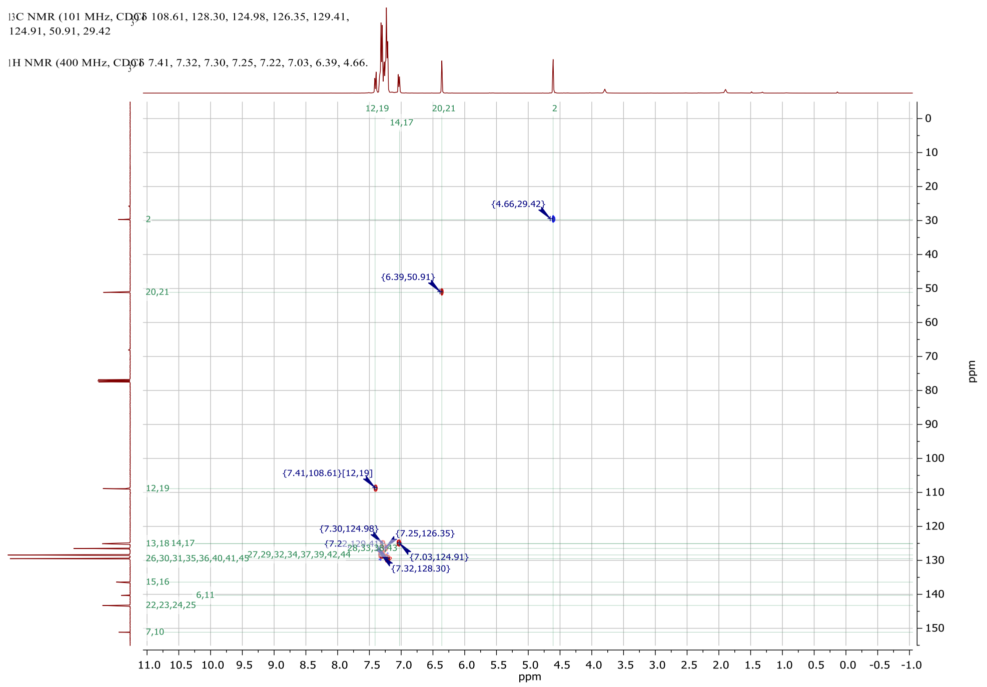

Figure S42. $^{1}H,^{13}C$ HSQC spectrum of **6** in $CDCl_3$.

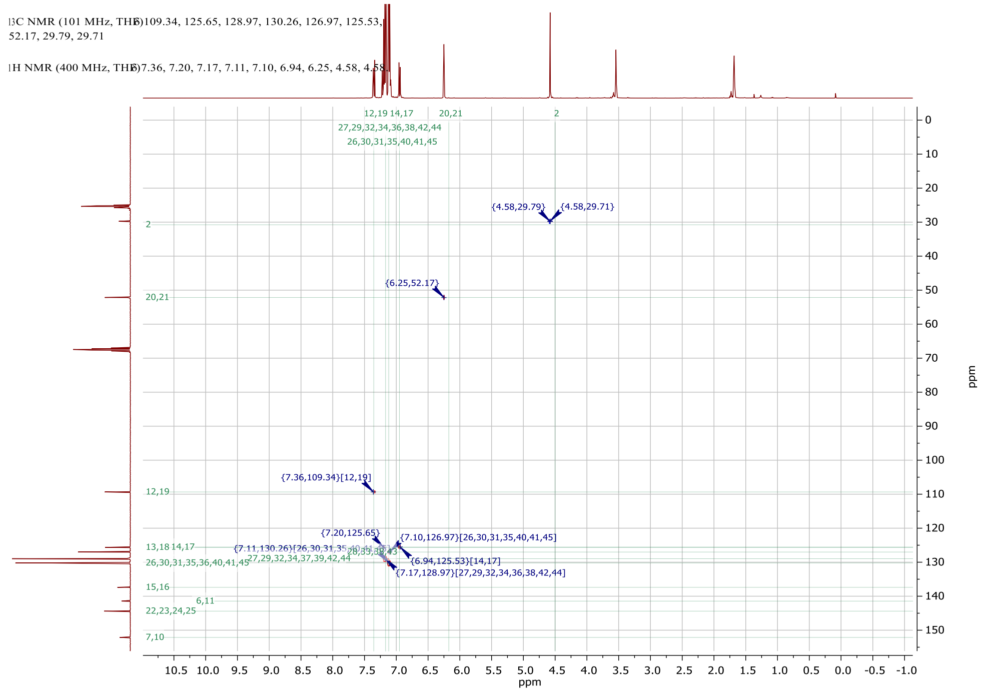

Figure S43. $^{1}H,^{13}C$ HSQC spectrum of **6** in [D8]THF.

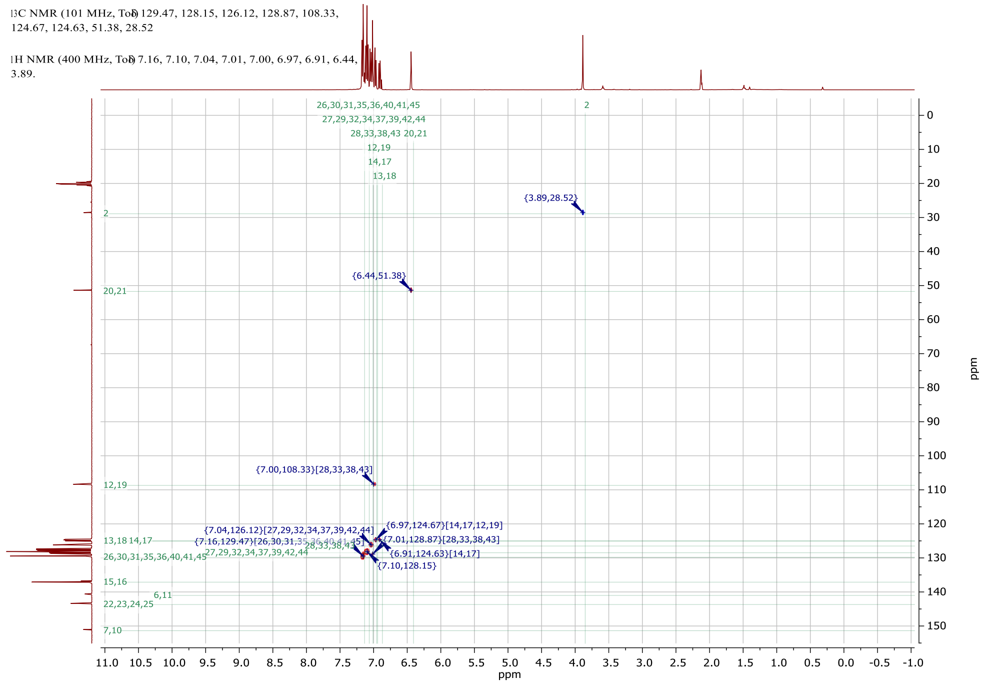

Figure S44. ^{1}H,^{13}C HSQC spectrum of **6** in [D$_8$]toluene.

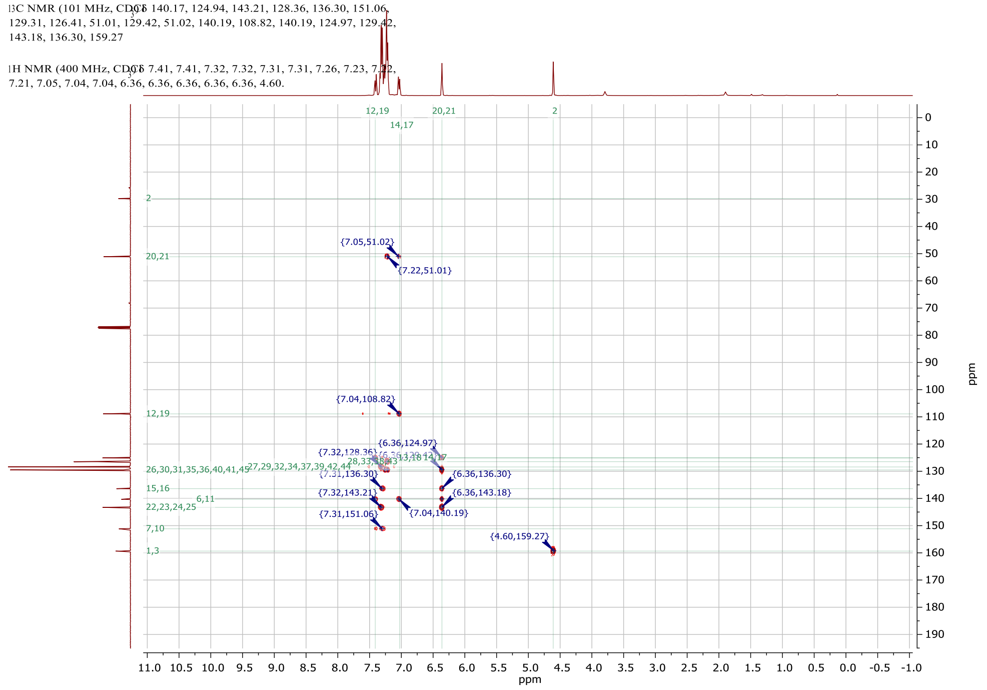

Figure S45. ^{1}H,^{13}C HMBC spectrum of **6** in $CDCl_3$.

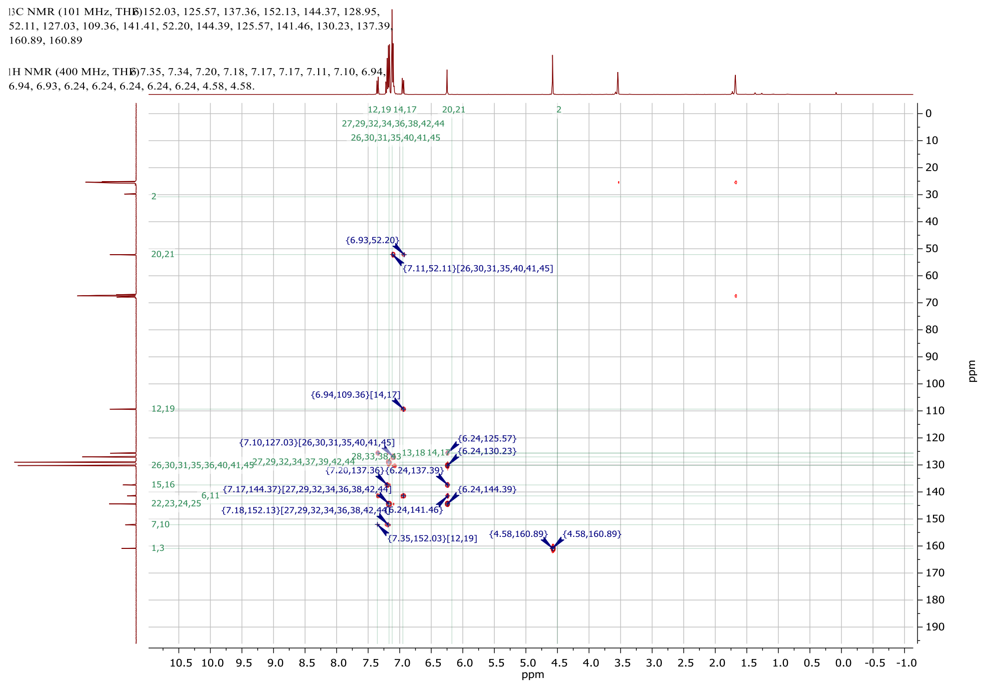

Figure S46. $^{1}H,^{13}C$ HMBC spectrum of **6** in [D$_8$]THF.

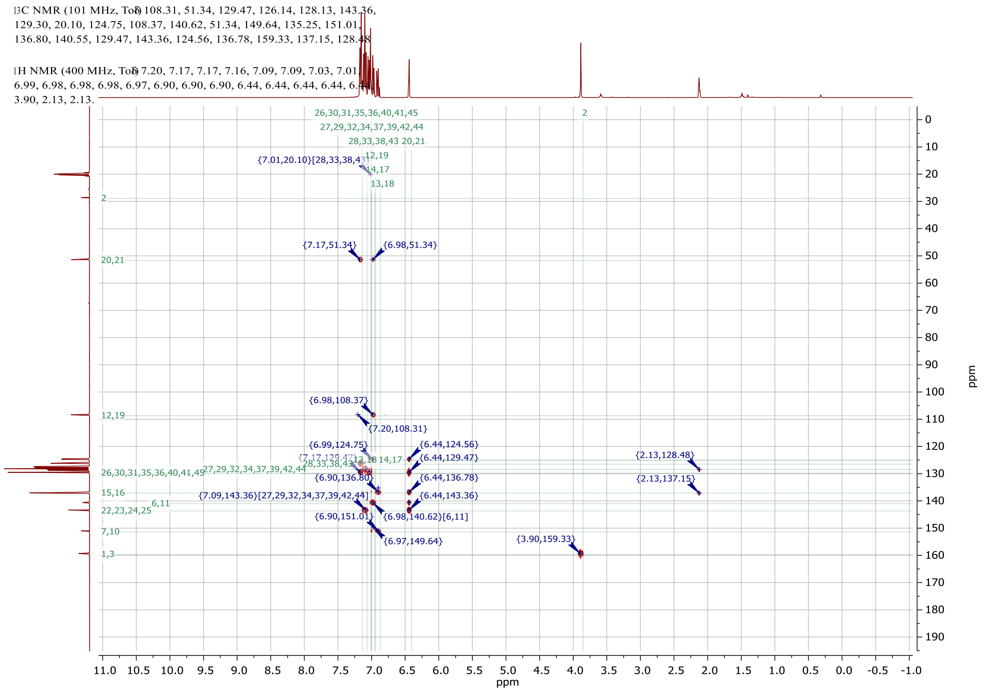

Figure S47. $^{1}H,^{13}C$ HMBC spectrum of **6** in [D$_8$]toluene.

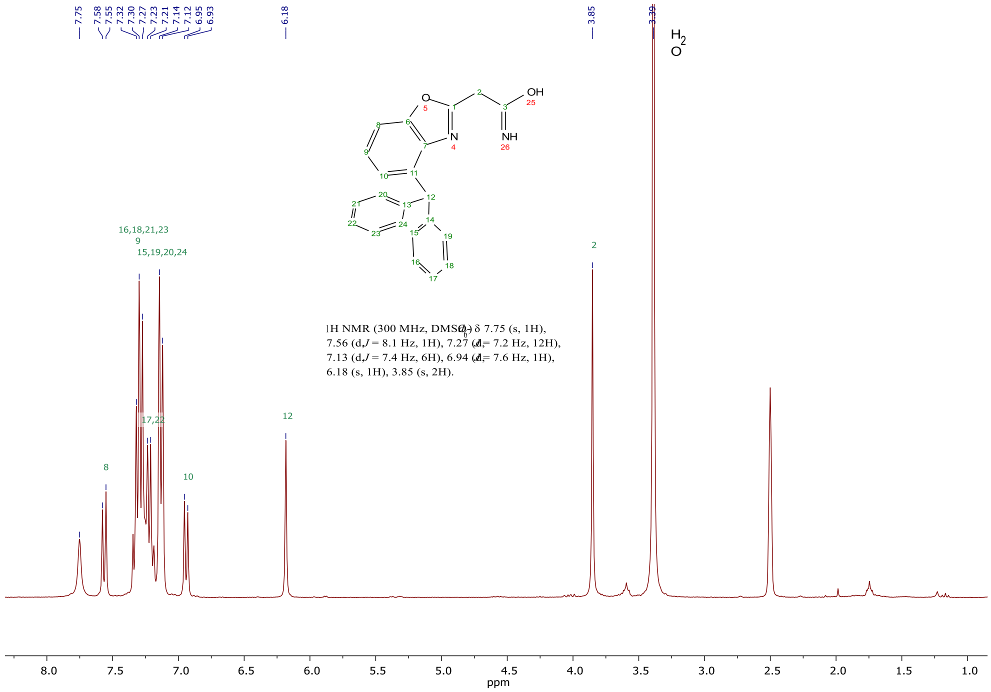

Figure S48. ^{1}H NMR spectrum of 2-(4-benzhydrylbenzoxazol-2-yl)acetimidic acid (byproduct) in [D₆]DMSO.

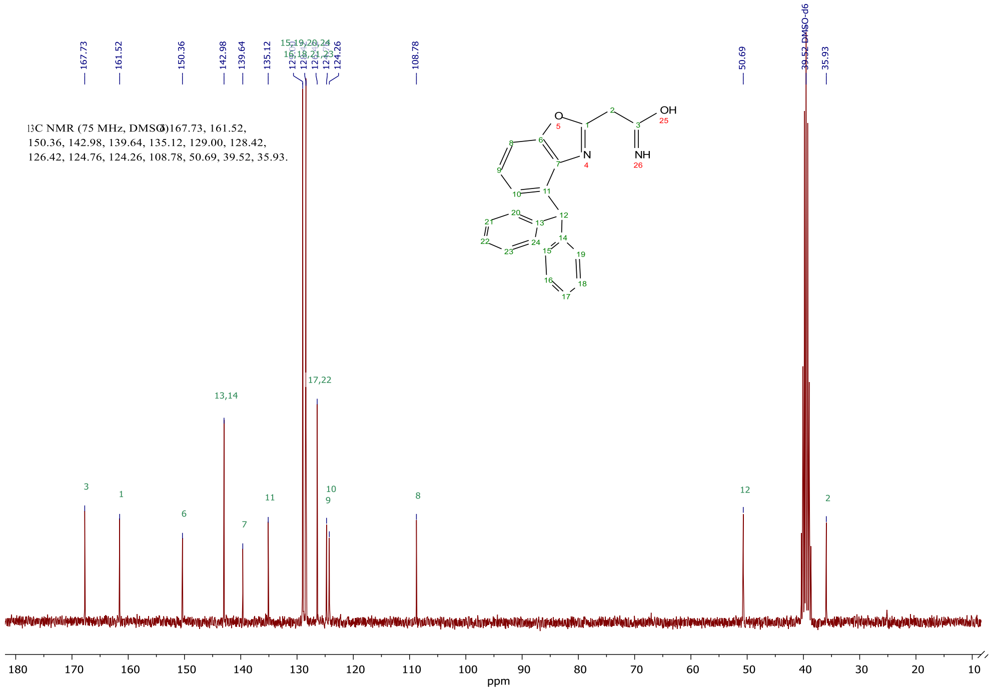

Figure S49. ^{13}C NMR spectrum of 2-(4-benzhydrylbenzoxazol-2-yl)acetimidic acid (byproduct) in [D₆]DMSO.

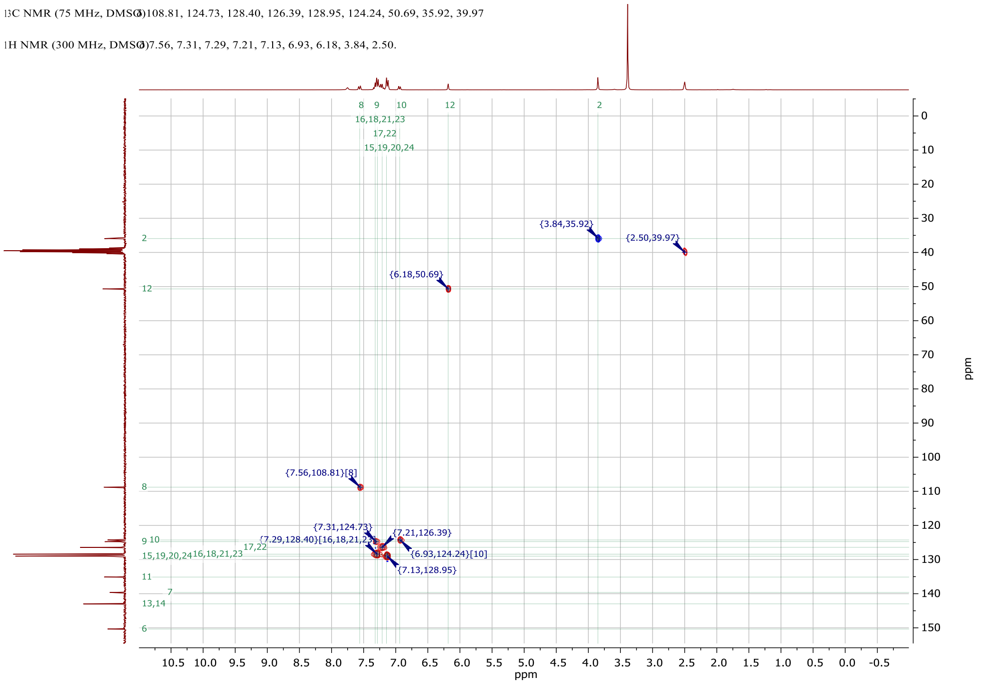

Figure S50. $^{1}H,^{13}C$ HSQC spectrum of 2-(4-benzhydrylbenzoxazol-2-yl)acetimidic acid (byproduct) in [D6]DMSO.

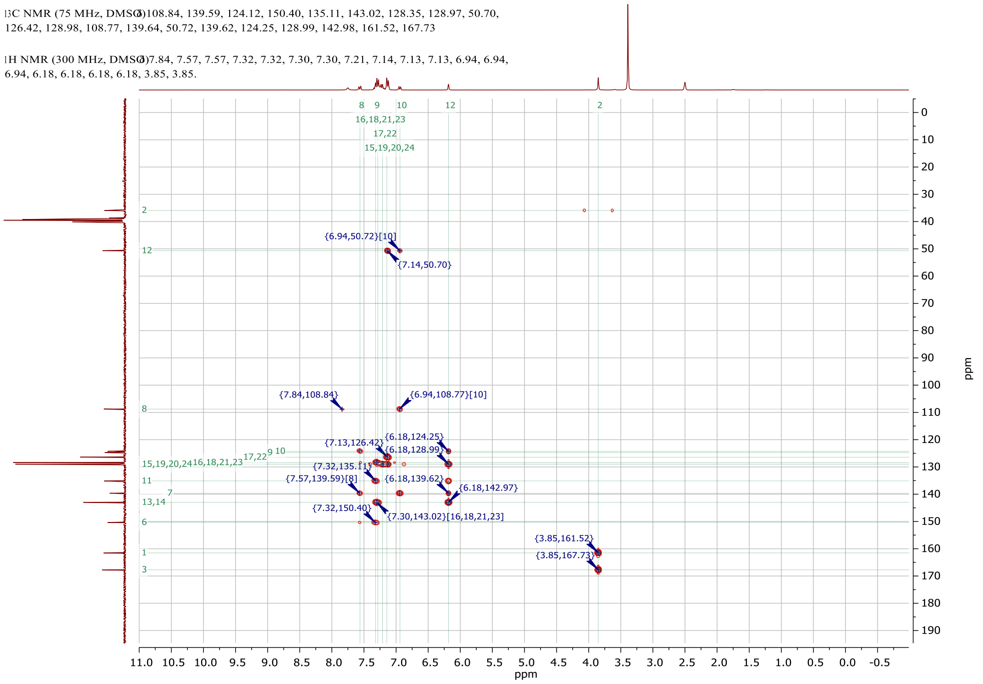

Figure S51. $^{1}H,^{13}C$ HMBC spectrum of 2-(4-benzhydrylbenzoxazol-2-yl)acetimidic acid (byproduct) in [D6]DMSO.

Mass spectrometry

Acquisition Parameter

Source Type	ESI	Ion Polarity	Positive	Set Nebulizer	1.2 Bar
Focus	Not active			Set Dry Heater	180 °C
Scan Begin	50 m/z	Set Capillary	4500 V	Set Dry Gas	4.0 l/min
Scan End	1600 m/z	Set End Plate Offset	-500 V	Set Divert Valve	Source

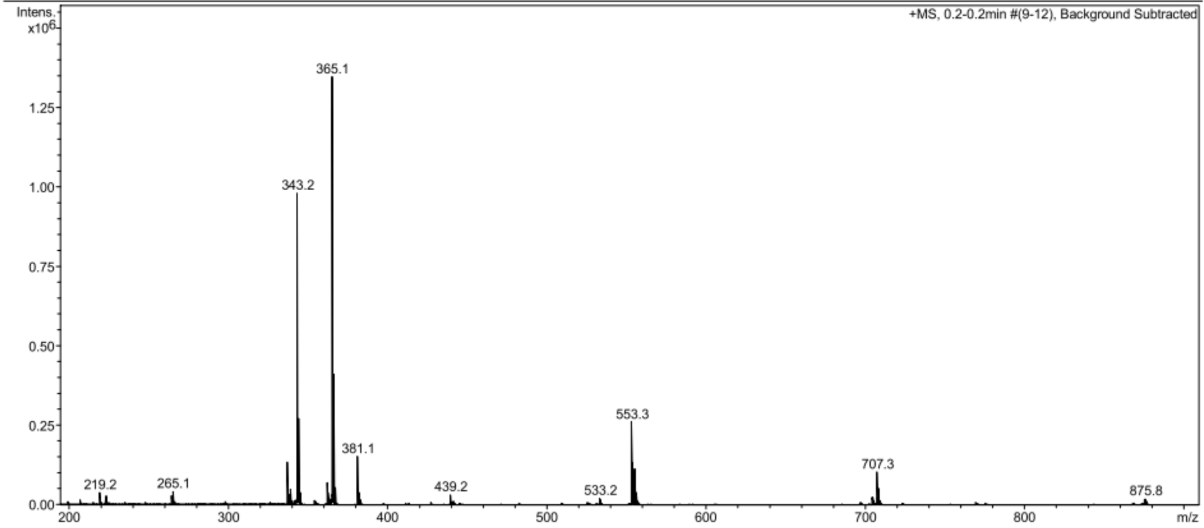

Figure S52. Mass spectrum of 2-(4-benzhydrylbenzoxazol-2-yl)acetimidic acid (ESI[+], THF) *m/z* (%): 365.1 (100) [*M*+Na]$^{+}$, 343.2 (73) [*M*+H]$^{+}$.

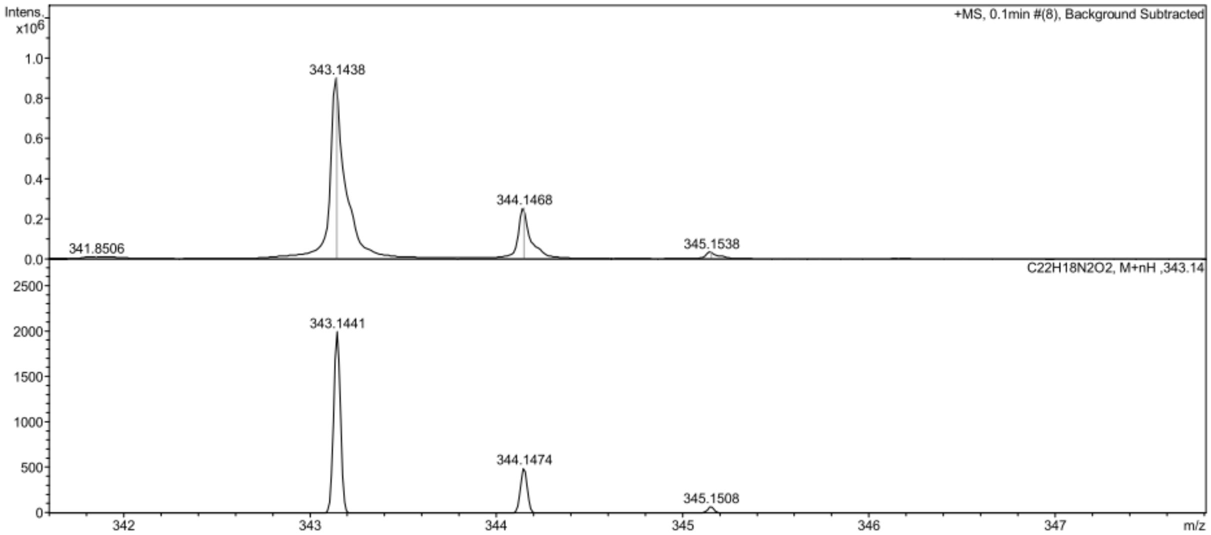

Figure S53. Mass spectrum HR-MS (ESI[+], THF) *m/z*: 343.1438 (cal. 343.1441 for [*M*+H]$^{+}$, $C_{22}H_{18}N_2O_2$).

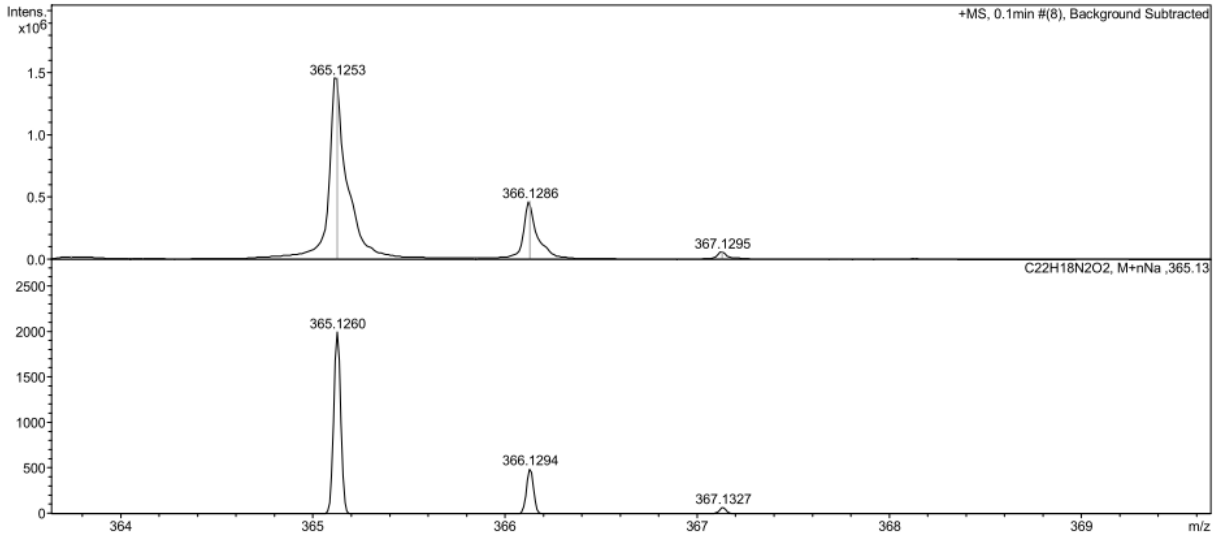

Figure S54. . Mass spectrum HR-MS (ESI[+], THF) *m/z*: 365.1253 (cal. 365.1253 for $[M+Na]^+$, $C_{22}H_{16}N_2NaO_2$).

Compound 7: Synthesis and Analytical Data

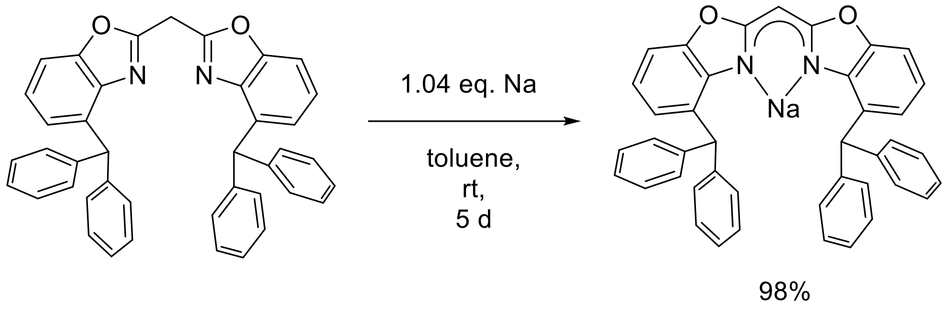

Synthesis of *N,N'*-bis(4-benzhydryl-benzoxazol-2-yl)methanido}sodium (7): Sodium (4.00 mg, 174 µmol, 1.04 eq.) as well as bis(4-benzhydryl-benzoxazol-2-yl)methane (97.4 mg, 167 µmol, 1.00 eq.) were weighed in and dissolved in toluene (6 mL). The reaction mixture was vigorously stirred for 5 After 5 d the sodium was completely consumed, the solvent was removed under reduced pressure. Obtained white solid was used for analyses without further purification. Crystals suitable for single X-ray diffraction experiments were grown from a saturated solution of **7** in toluene at −28°C. Yield: 99 mg (98%).

^{1}H NMR (300 MHz, [D$_8$]toluene, 298 K): δ = 7.01-6.95 (m, 20 H, Bzh-*H*), 7.05 (d, 2 H, $^{3}J_{HH}$ = 7.1 Hz, 5-H, 15-H), 6.71 (dd, 2 H, $^{3}J_{HH}$ = 7.8 Hz, 4-H, 12-H), 6.60 (d, 2 H, $^{3}J_{HH}$ = 7.7 Hz, 3-H, 13-H), 5.38 (s, 1 H, 8-H), 5.27 (s, 2 H, 16-H, 29-H) ppm.

^{13}C{^{1}H} NMR (75 MHz, [D$_8$]toluene, 298 K): δ = 169.77 (7-C, 9-C), 149.77 (6-C, 10-C), 143.78 (1-C, 15-C), 129.44 (18-C, 22-C, 24-C, 28-C, 31-C, 35-C, 37-C, 41-C),129.15 (17-C, 23-C, 30-C, 36-C), 129.07 (19-C, 21-C, 25-C, 27-C, 32-C, 34-C, 38-C, 40-C), 126.91 (20-C, 26-C, 33-C, 39-C), 126.83 (2-C, 14-C), 124.36 (3-C, 13-C), 58.51 (8-C), 53.63 (16-C, 29-C) ppm.

MS (LIFDI[+], toluene) *m/z* (%): 604.1 (100) [*M*]$^{+}$.

Elemental analysis in % (calculated) $C_{41}H_{29}N_2NaO_2$ (604.68 g/mol): C 79.38 (81.44), H 4.97 (4.83), N 4.61 (4.63) .

NMR spectroscopy

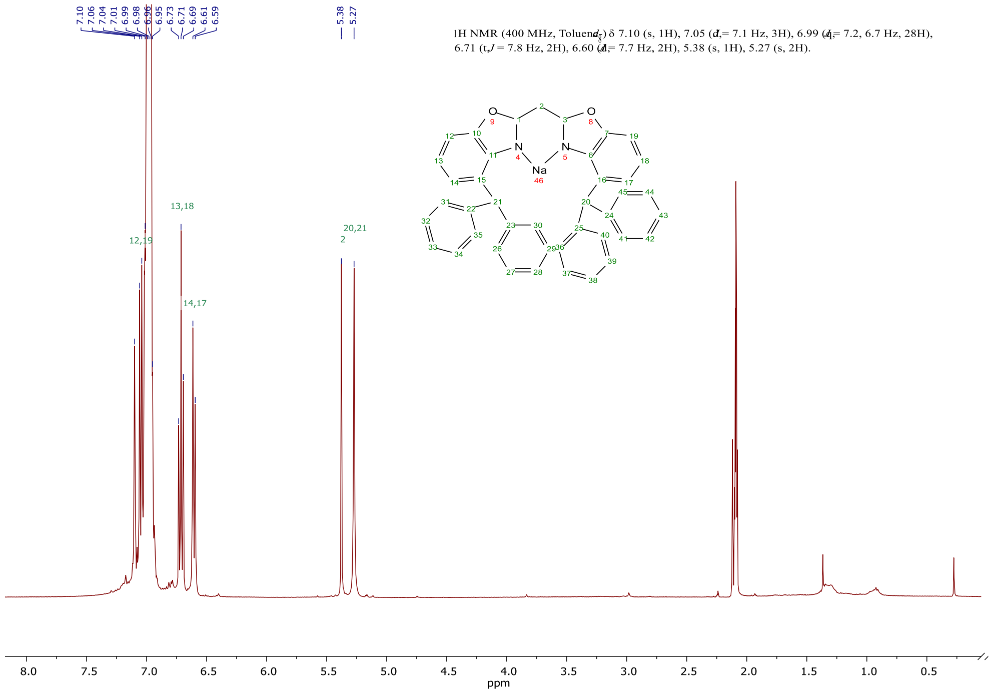

Figure S55. ^{1}H NMR spectrum of **7** in [D8]toluene.

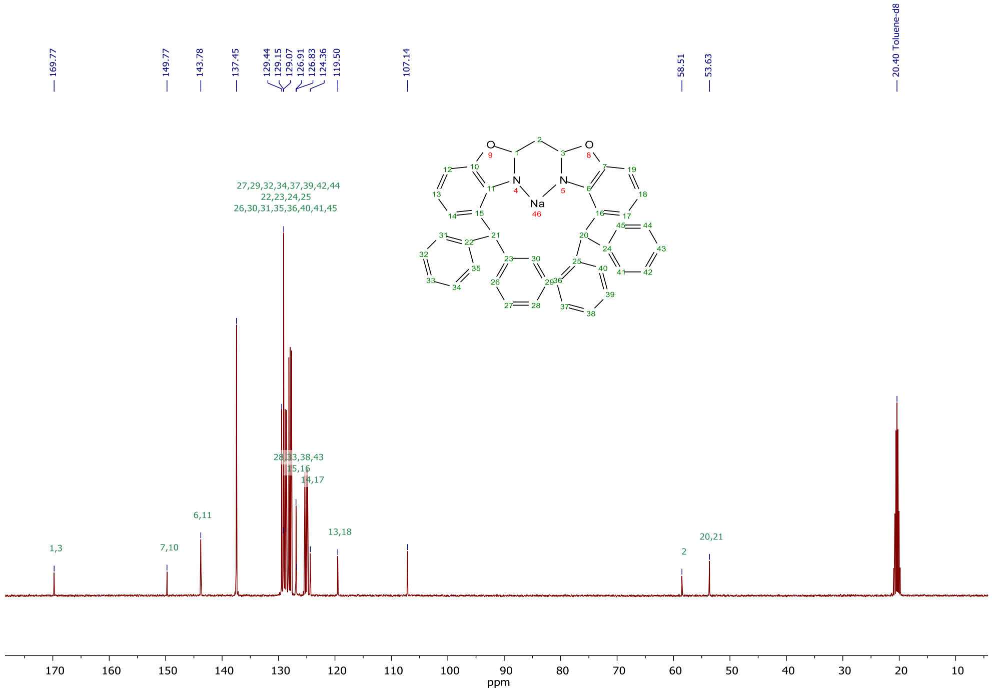

Figure S56. ^{13}C NMR spectrum of **7** in [D8]toluene.

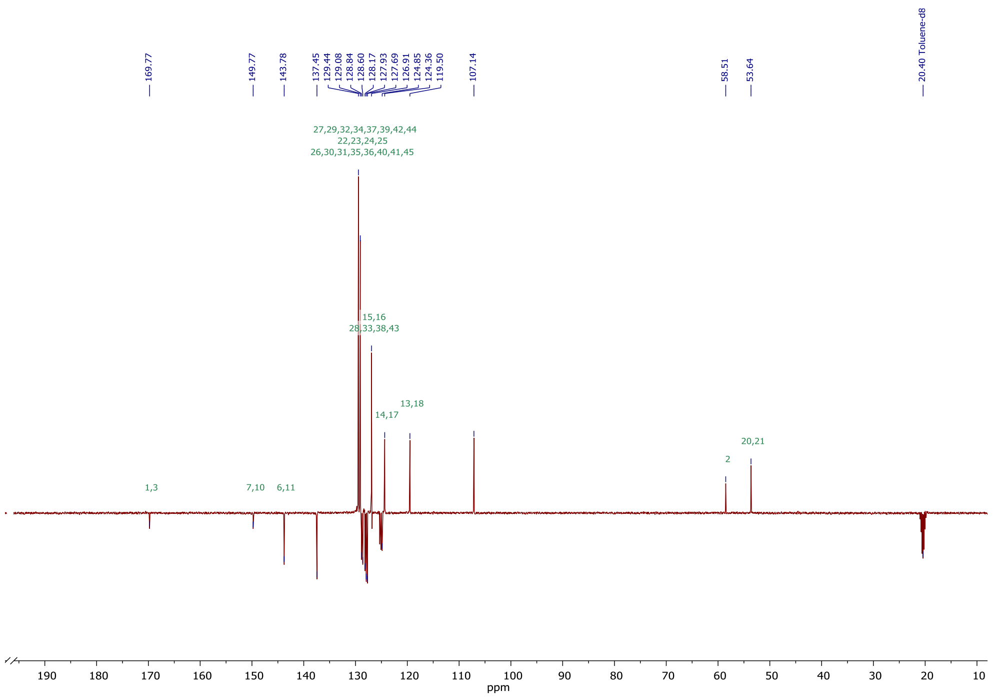

Figure S57. ^{13}C(APT) NMR spectrum of **7** in [D8]toluene.

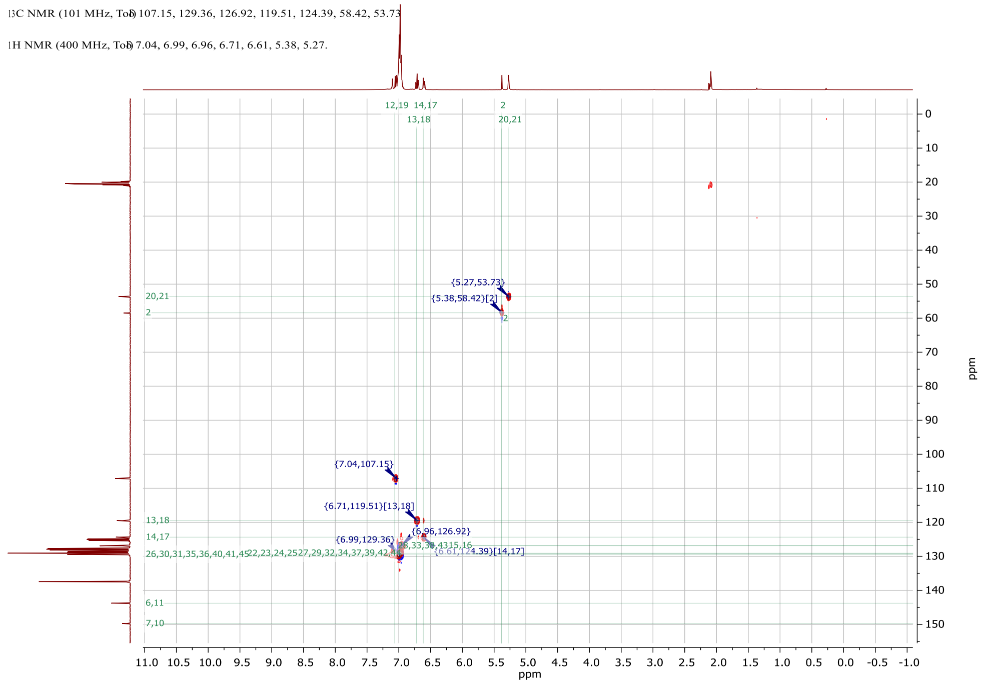

Figure S58. $^{1}H,^{13}C$ HSQC spectrum of **7** in [D8]toluene.

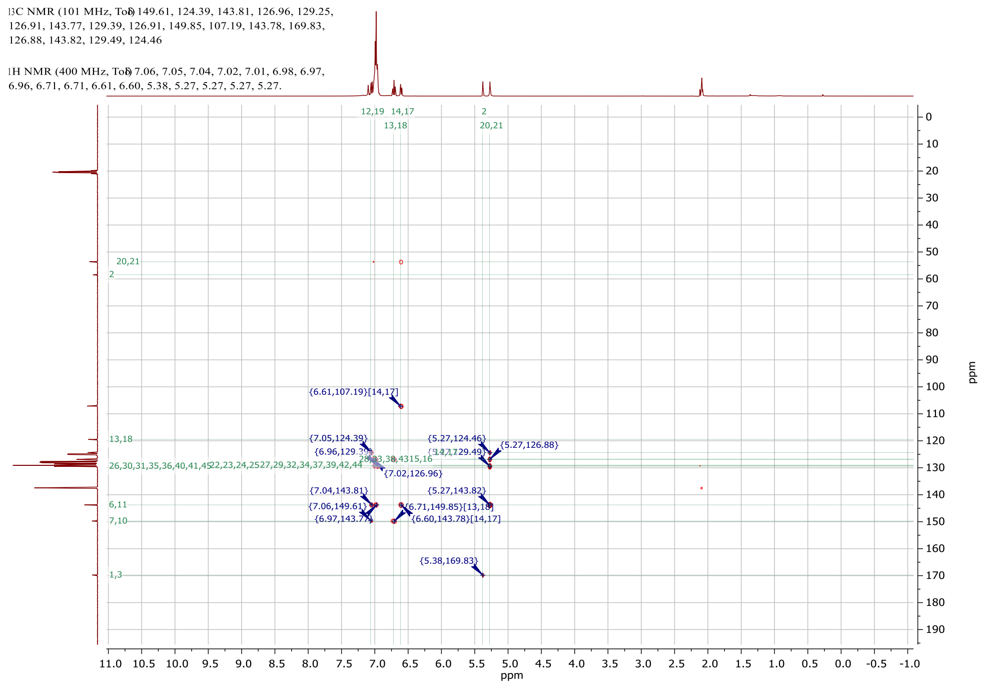

Figure S59. ^{1}H,^{13}C HMBC spectrum of **7** in [D$_8$]toluene.

Mass spectrometry

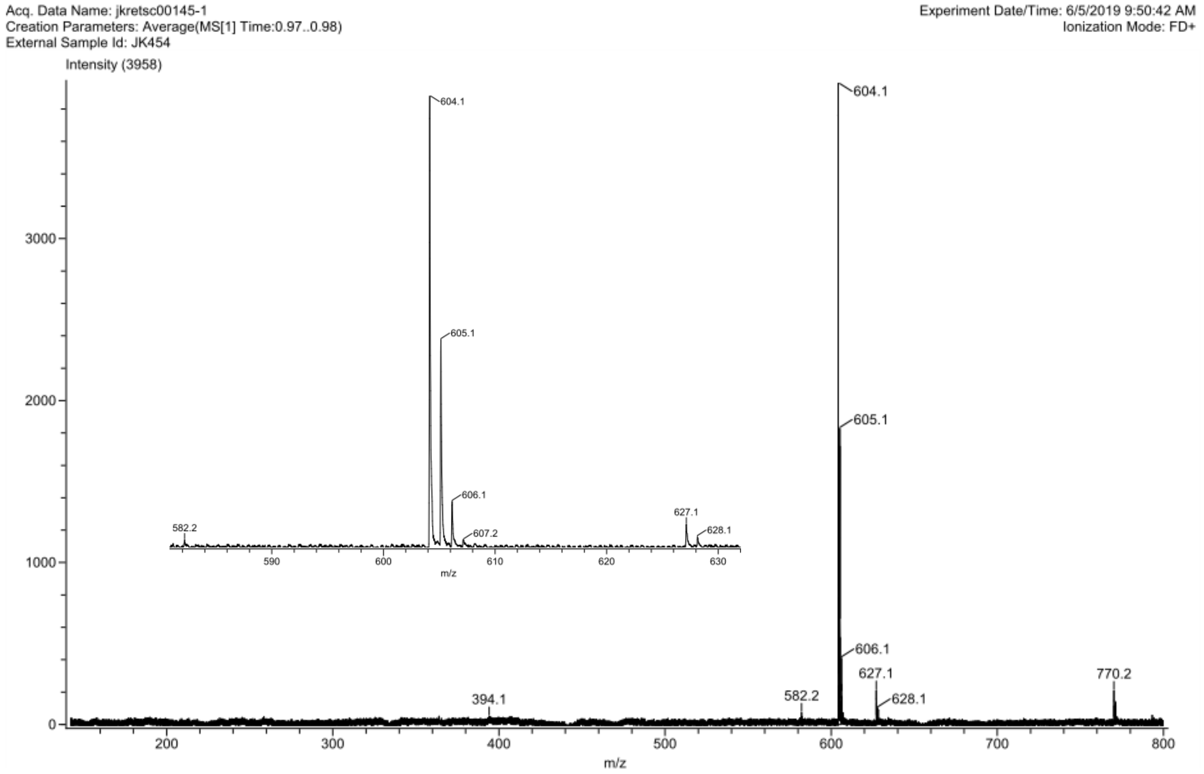

Figure S60. Mass spectrum of **7** (LIFDI[+], toluene) *m/z* (%): 604.1 (100) [*M*]$^+$.

Compound 8: Synthesis and Analytical Data

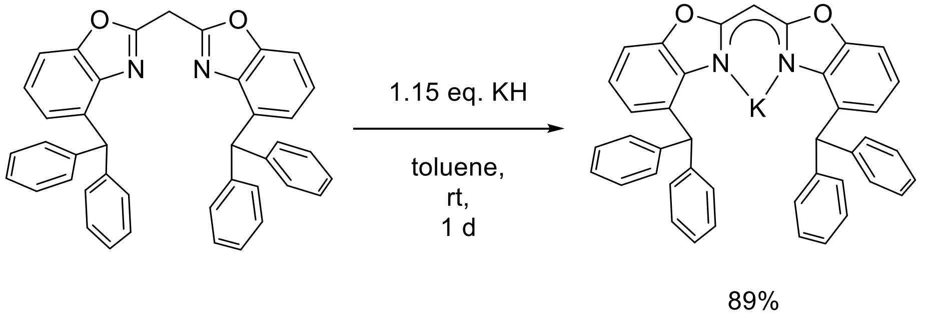

Synthesis of *N,N'*-bis(4-benzhydryl-benzoxazol-2-yl)methanido}potassium (8): Bis(4-benzhydryl-benzoxazol-2-yl)methane (180 mg, 309 µmol, 1.00 eq.) was dissolved in toluene (8 mL). After potassium hydride (14.2 mg, 354 µmol, 1.15 eq.) was added to the solution at room temperature hydrogen formation was observed and the mixture turned immediately red. The reaction mixture was vigorously stirred for 1 d. Afterwards the solvent was removed under reduced pressure. The reddish white powder was used for further syntheses without further purification. Crystals suitable for single crystal X-ray diffraction were obtained out of a saturated toluene solution at −30°C after 1 d. Yield: 196 mg (89%).

^{1}H NMR (300 MHz, $[D_8]$THF, 298 K): δ = 7.32-7.17 (m, 12 H, 19-20- 21-25-26-27-32-33-34- 38-39- 40-H), 7.08-7.06 (m, 8 H, 18-22- 24-28-31-35-37- 41-), 6.93 (dd, 2 H, $^3J_{HH}$ = 7.8 Hz, $^4J_{HH}$ = 1.0 Hz, 5-H, 11-H), 6.63 (dd, 2 H, $^3J_{HH}$ = 7.8 Hz, 4-H, 12-H), 6.33 (dd, 2 H, $^3J_{HH}$ = 7.8 Hz, $^4J_{HH}$ = 1.0 Hz, 3-H, 13-H), 6.03 (s, 2 H, 16-H, 29-H), 4.66 (s, 1 H, 8-H) ppm.

^{13}C{^{1}H} NMR (75 MHz, $[D_8]$THF, 298 K): δ = 169.98 (7-C, 9-C), 149.88 (6-C, 10-C), 145.82 (1-- 145.44 (17-3- 30-36-), 130.52 (18-22- 24-28-31-35-37- 41-), 129.33 (19-21- 25-27- 32-34-38-40-), 128.21 (2-C, 14-C), 127.14, (20-26- 33-39-), 123.68 (3-C, 13-C), 118.70 (4-C, 12-C), 106.22 (5-C, 11-C), 57.52 (8-C), 51.92 (16-C, 29-C) ppm.

MS (LIFDI[+], THF) *m/z* (%): 659.3 (4) $[M+K]^+$, 620.3 (100) $[M]^+$, 582.3 (4) $[M{-}K+H]^+$.

Elemental analysis in % (calculated) $C_{41}H_{29}KN_2O_2$ (620.79 g/mol): C 75.10 (79.33), H 4.71 (4.71), N 4.50 (4.51) (The deviation could be due to minor contaminations by KH or moisture).

NMR spectroscopy

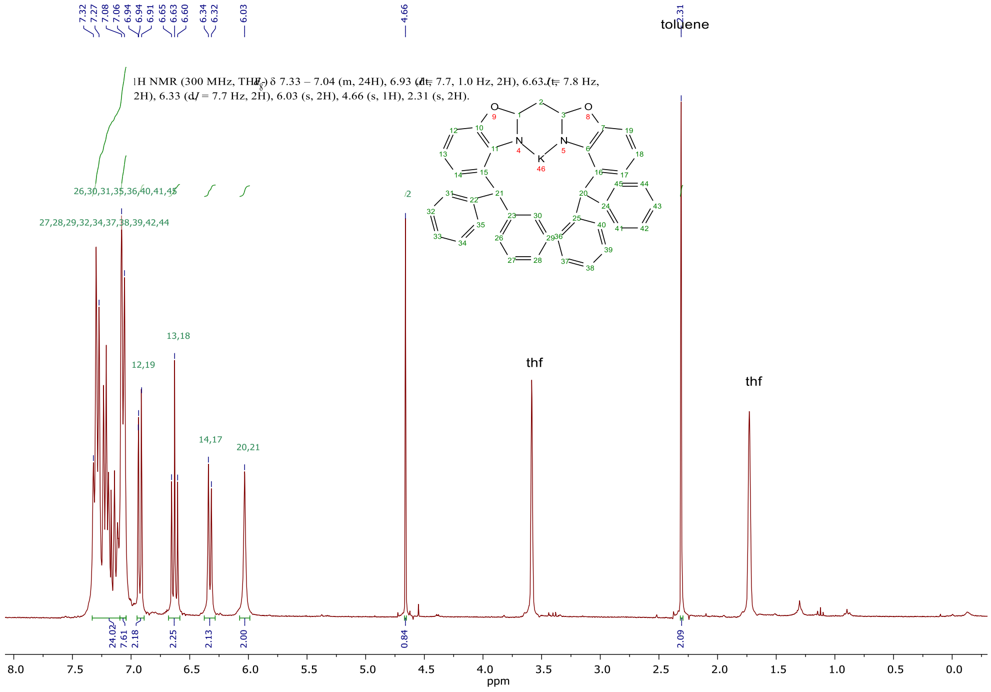

Figure S61. ^{1}H NMR spectrum of **8** in [D8]THF.

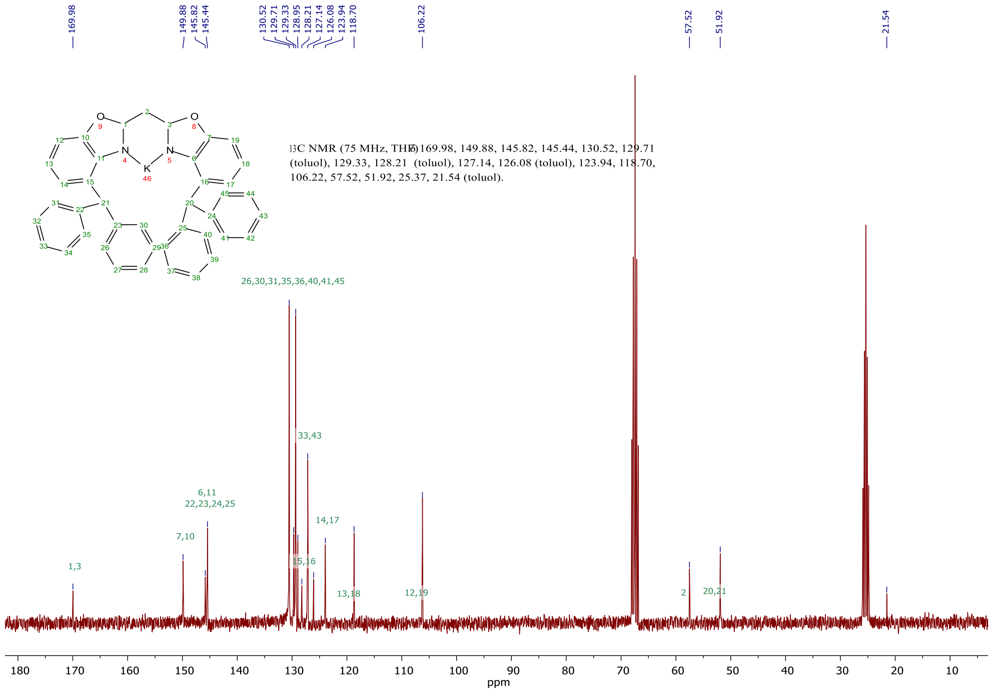

Figure S62. ^{13}C NMR spectrum of **8** in [D8]THF.

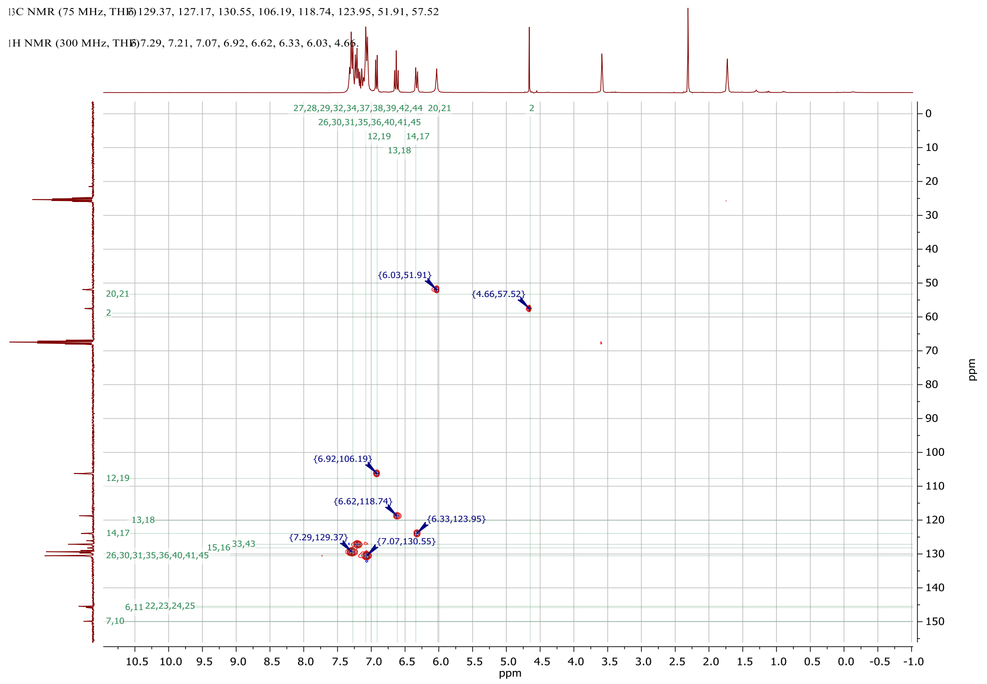

Figure S63. $^{1}H,^{13}C$ HSQC spectrum of **8** in [D₈]THF.

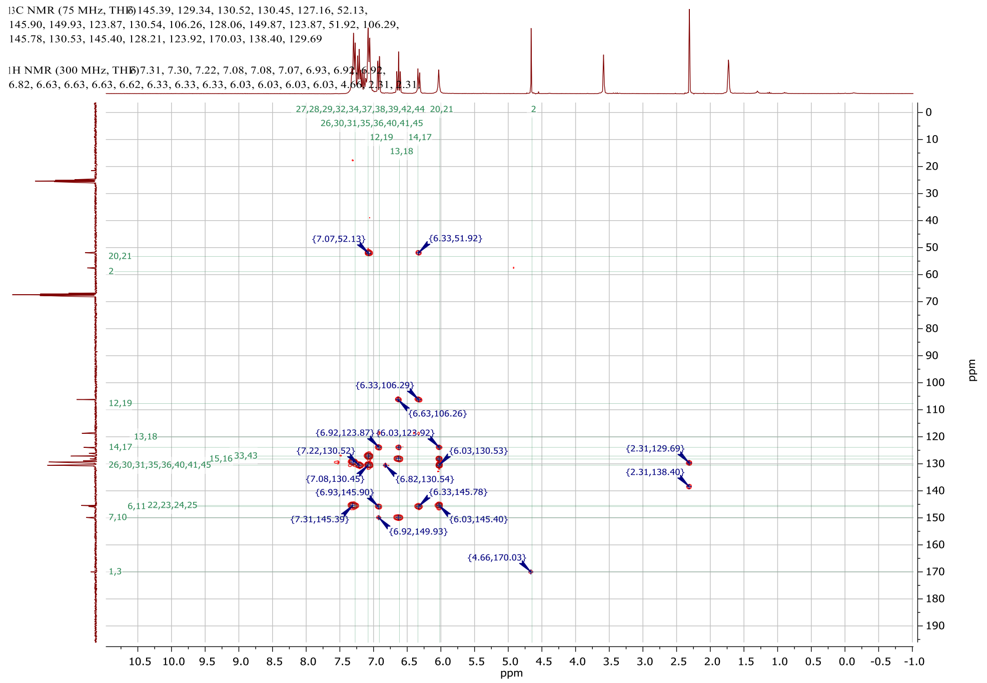

Figure S64. $^{1}H,^{13}C$ HMBC spectrum of **8** in [D₈]THF.

Mass spectrometry

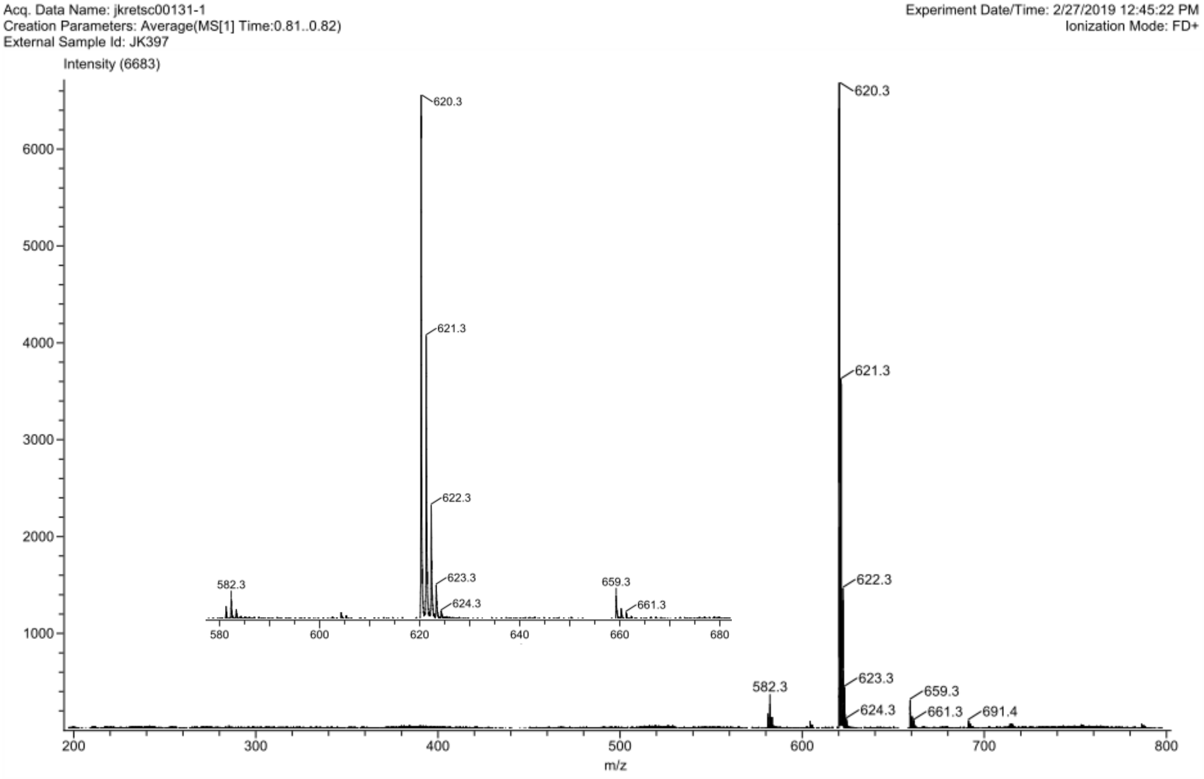

Figure S65. Mass spectrum of **8** (LIFDI[+], THF) *m/z* (%): 659.3 (4) [*M*+K]$^+$, 620.3 (100) [*M*]$^+$, 582.3 (4) [*M*−K+H]$^+$.

Compound 9: Synthesis and Analytical Data

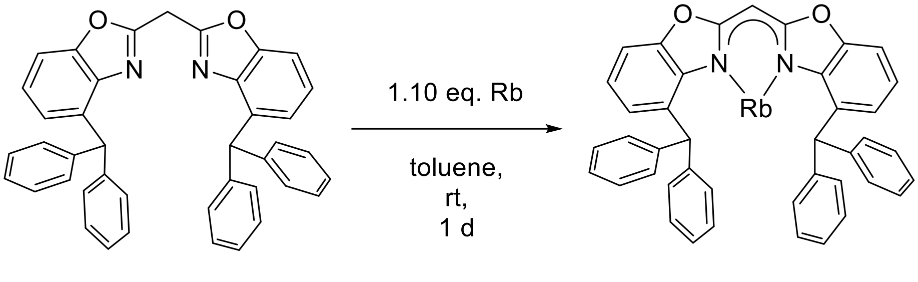

Synthesis of *N,N'*-bis(4-benzhydryl-benzoxazol-2-yl)methanido}rubidium (9): A solution of bis(4-benzhydryl-benzoxazol-2-yl)methane (97.7 mg, 168 µmol, 1.00 eq.) in toluene (6 mL) was added to rubidium (15.8 mg, 185 µmol, 1.10 eq.). The solution turned red immediately and hydrogen formation was observed. The reaction mixture was vigorously stirred at ambient temperature until all rubidium (1 d) was consumed. Afterwards the solvent was removed under reduced pressure. The reddish white powder was used for further analysis without additional workup. Crystals suitable for single XRD were obtained by liquid-liquid diffusion of pentane out in a saturated toluene solution at −30°C after 3 d. Yield: 85.4 mg (76%).

^{1}H NMR (300 MHz, [D$_8$]THF, 298 K): δ = 7.30-7.22 (m, 12 H, 19-20- 21-25-26-27-32-33-34- 38-39- 40-H), 7.12-7.09 (m, 8 H, 18-22- 24-28-31-35-37- 41-), 6.92 (dd, 2 H, $^{3}J_{HH}$ = 7.8 Hz, $^{4}J_{HH}$ = 1.0 Hz, 5-H, 11-H), 6.63 (dd, 2 H, $^{3}J_{HH}$ = 7.8 Hz, 4-H, 12-H), 6.37 (dd, 2 H, $^{3}J_{HH}$ = 7.8 Hz, $^{4}J_{HH}$ = 1.0 Hz, 3-H, 13-H), 6.06 (s, 2 H, 16-H, 29-H), 4.65 (s, 1 H, 8-H) ppm.

^{13}C{^{1}H} NMR (75 MHz, [D$_8$]THF, 298 K): δ = 169.83 (7-C, 9-C), 150.01 (6-C, 10-C), 145.85 (1-- 138.47 (17-3- 30-36-), 130.73 (18-22- 24-28-31-35-37- 41-), 129.27 (19-21- 25-27- 32-34-38-40-), 128.38 (2-C, 14-C), 126.99 (20-26- 33-39-), 123.87 (3-C, 13-C), 118.61 (4-C, 12-C), 106.13 (5-C, 11-C), 57.17 (8-C), 52.14 (16-C, 29-C) ppm.

^{87}Rb NMR (176 MHz, [D$_8$]THF, 298 K): δ = −1.69, −254.69 ppm.

MS (LIFDI[+], THF) *m/z* (%): 751.1 (95) [*M*+Rb]$^{+}$, 667.1 (100) [*M*+H]$^{+}$, 666.1 (50) [*M*]$^{+}$, 582.3 (7) [*M*−Rb+H]$^{+}$.

Elemental analysis in % (calculated) $C_{41}H_{29}N_2O_2Rb$ (667.16 g/mol): C 71.65 (73.81), H 4.69 (4.38), N 4.73 (4.20) (The deviation could be due to minor contaminations by Rb or moisture).

NMR spectroscopy

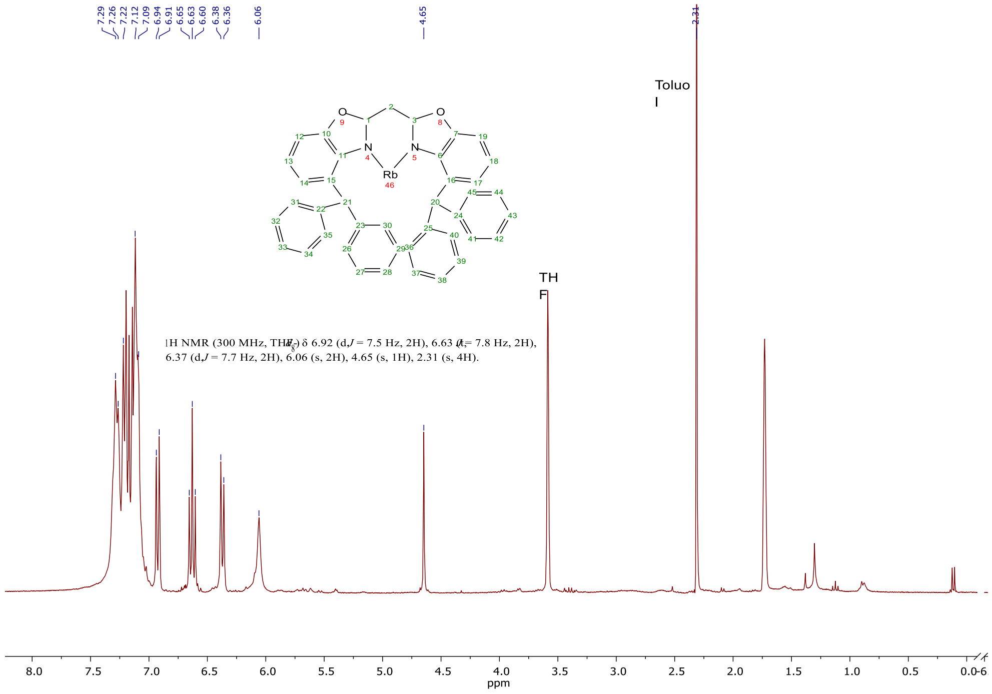

Figure S66. ^{1}H NMR spectrum of **9** in [D8]THF.

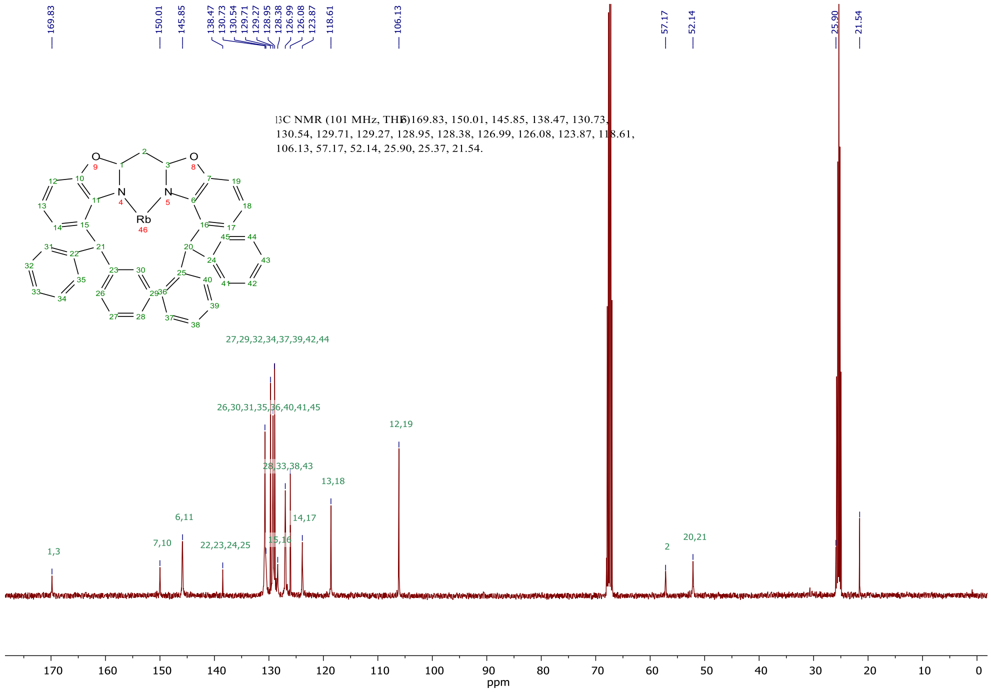

Figure S67. ^{13}C NMR spectrum of **9** in [D8]THF.

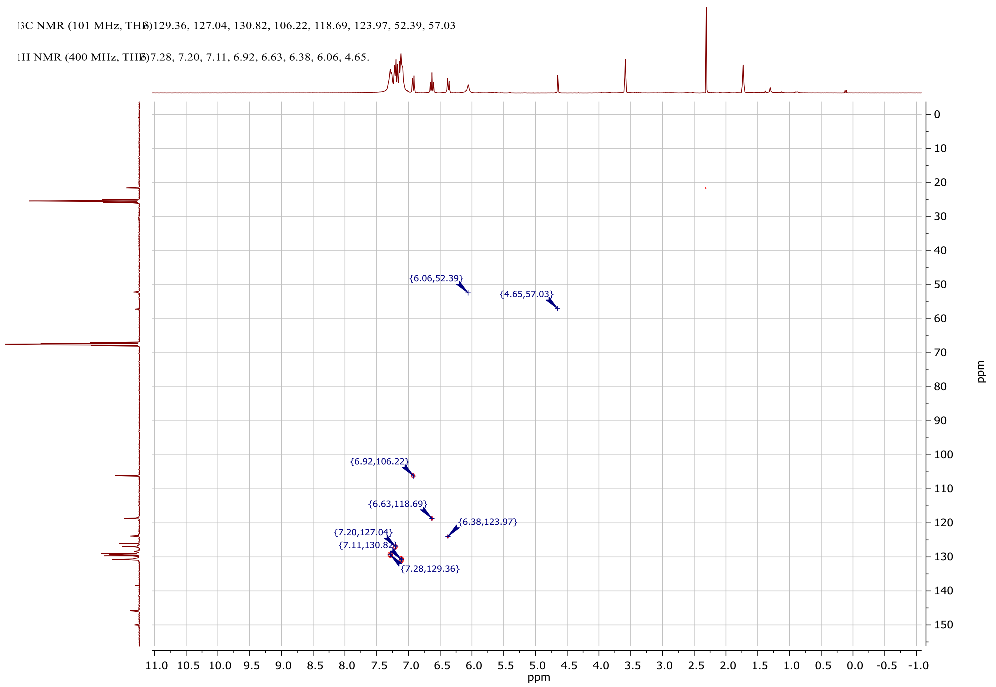

Figure S68. $^{1}H,^{13}C$ HSQC spectrum of **9** in [D8]THF.

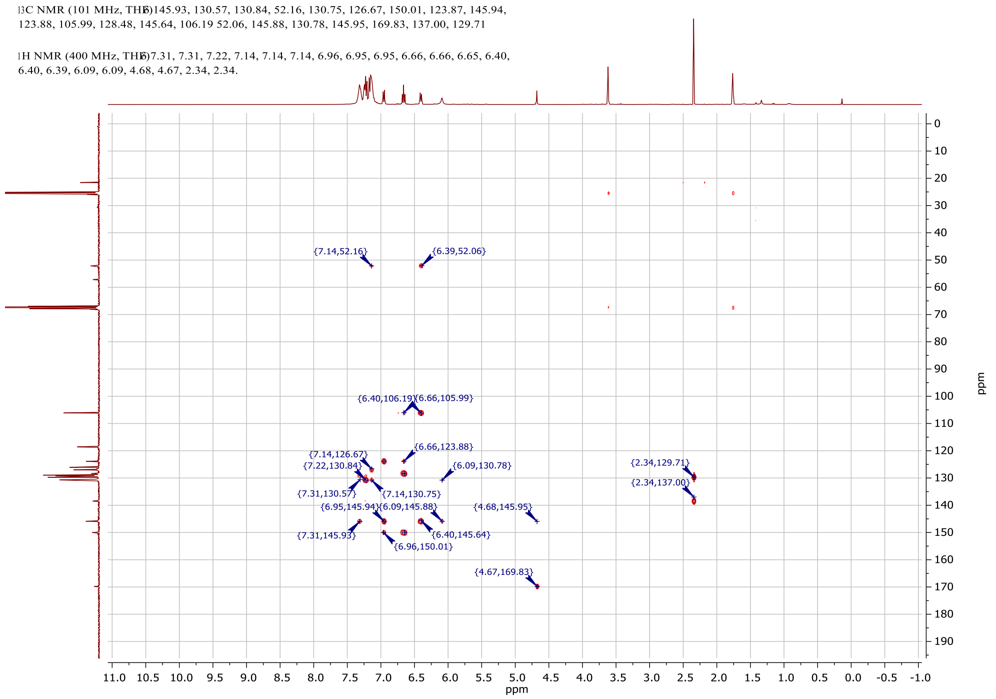

Figure S69. $^{1}H,^{13}C$ HBMC spectrum of **9** in [D8]THF.

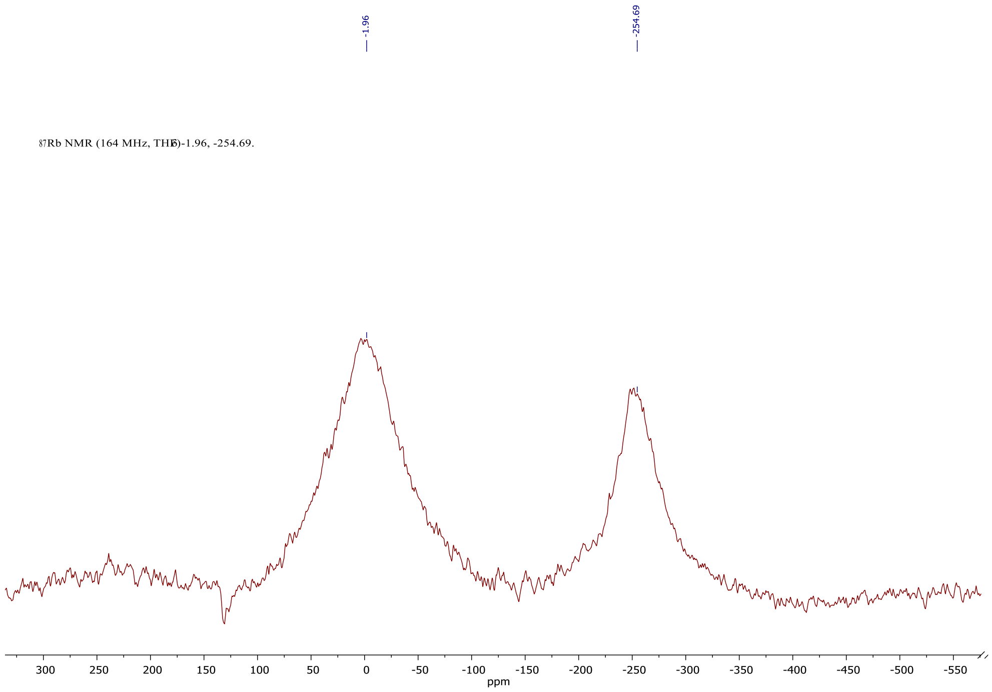

Figure S70. ^{87}Rb NMR spectrum of **9** in [D$_8$]THF.

Mass spectrometry

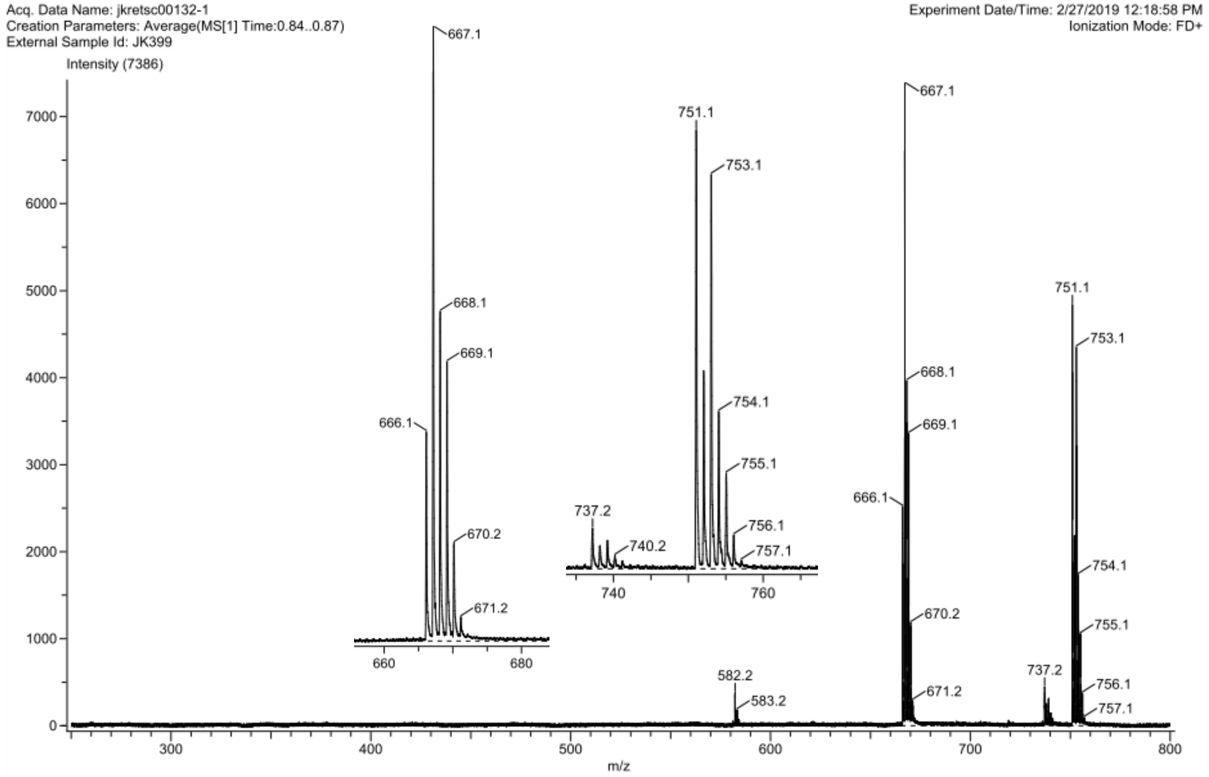

Figure S71. Mass spectrum of **9** (LIFDI[+], THF) *m/z* (%): 751.1 (95) [*M*+Rb]$^+$, 667.1 (100) [*M*+H]$^+$, 666.1 (50) [*M*]$^+$, 582.3 (7) [*M*−Rb+H]$^+$.

Compound 10: Synthesis and Analytical Data

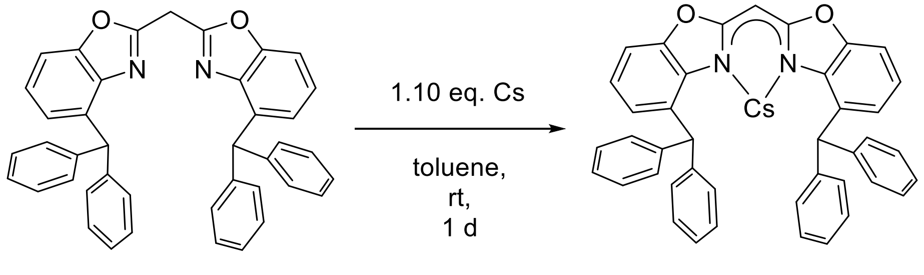

74%

Synthesis of *N*,*N'*-bis(4-benzhydryl-benzoxazol-2-yl)methanido}cesium (10): A solution of bis(4-benzhydryl-benzoxazol-2-yl)methane (102.5 mg, 175.9 µmol, 1.00 eq.) in toluene (6 mL) was added to cesium (25.7 mg, 193. µmol, 1.10 eq.). The solution turned red immediately and hydrogen formation was observed. The reaction mixture was vigorously stirred at ambient temperature until all cesium (~6 h) was consumed. Afterwards the solvent was removed under reduced pressure. The reddish white powder was used for further analysis without additional workup. Crystals suitable for single crystal X-ray diffraction were obtained by liquid-liquid diffusion of pentane out in a saturated toluene solution at −30°C after 1 d. Yield: 93.3 mg (74%).

^{1}H NMR (300 MHz, [D$_8$]THF, 298 K): δ = 7.28-7.16 (m, 12 H, 19-20- 21-25-26-27-32-33-34- 38-39- 40-H), 7.14-7.08 (m, 8 H, 18-22- 24-28-31-35-37- 41-), 6.92 (dd, 2 H, $^{3}J_{HH}$ = 7.7 Hz, $^{4}J_{HH}$ = 1.0 Hz, 5-H, 11-H), 6.63 (dd, 2 H, $^{3}J_{HH}$ = 7.7 Hz, 4-H, 12-H), 6.43 (dd, 2 H, $^{3}J_{HH}$ = 7.7 Hz, $^{4}J_{HH}$ = 1.0 Hz, 3-H, 13-H), 6.17 (s, 2 H, 16-H, 29-H), 4.64 (s, 1 H, 8-H) ppm.

^{13}C{^{1}H} NMR (75 MHz, [D$_8$]THF, 298 K): δ = 169.58 (7-C, 9-C), 150.25 (6-C, 10-C), 146.32 (1-- 145.78 (17-3- 30-36-), 130.77 (18-22- 24-28-31-35-37- 41-), 129.31 (19-21- 25-27- 32-34-38-40-), 129.06 (2-C, 14-C), 126.90 (20-26- 33-39-), 123.81 (3-C, 13-C), 118.38 (4-C, 12-C), 105.98 (5-C, 11-C), 56.64 (8-C), 52.23 (16-C, 29-C) ppm.

^{133}Cs NMR (53 MHz, [D$_8$]THF, 298 K): δ = −31.12 ppm.

MS (LIFDI[+], THF) *m/z* (%): 714.0 (7) [*M*]$^+$, 132.9 (100) [Cs]$^+$.

Elemental analysis in % (calculated) $C_{48}H_{37}N_2O_2Cs$ (714.60 g/mol): C 69.80 (71.46), H 4.48 (4.62), N 4.52 (3.47) (The deviation is due to partial loss of toluene in the drying process and minor contamination with **6**).

NMR spectroscopy

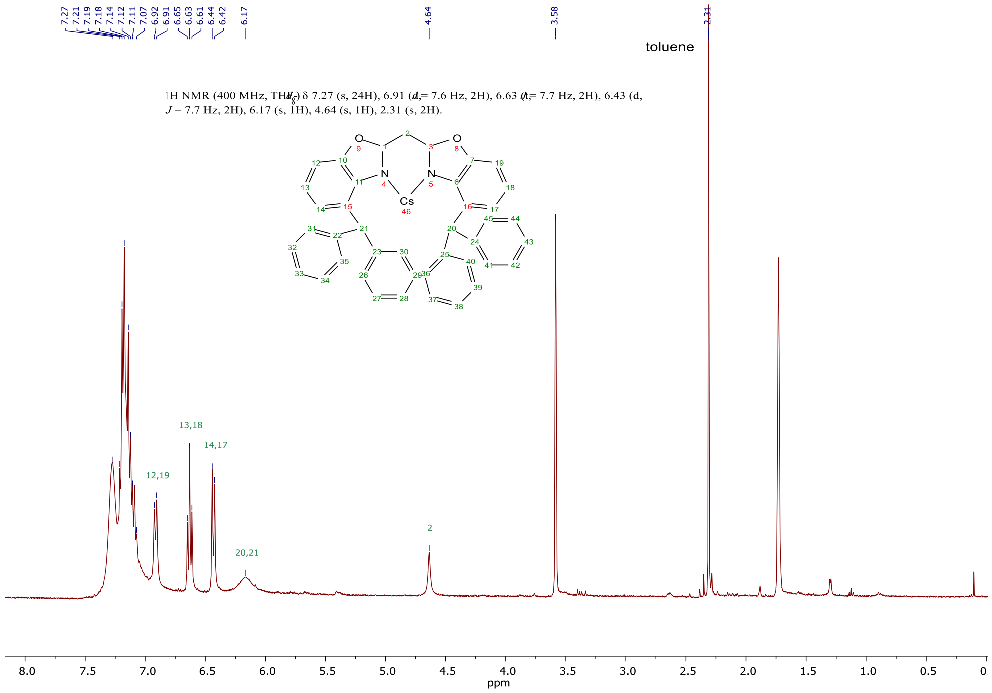

Figure S72. ^{1}H NMR spectrum of **10** in [D8]THF.

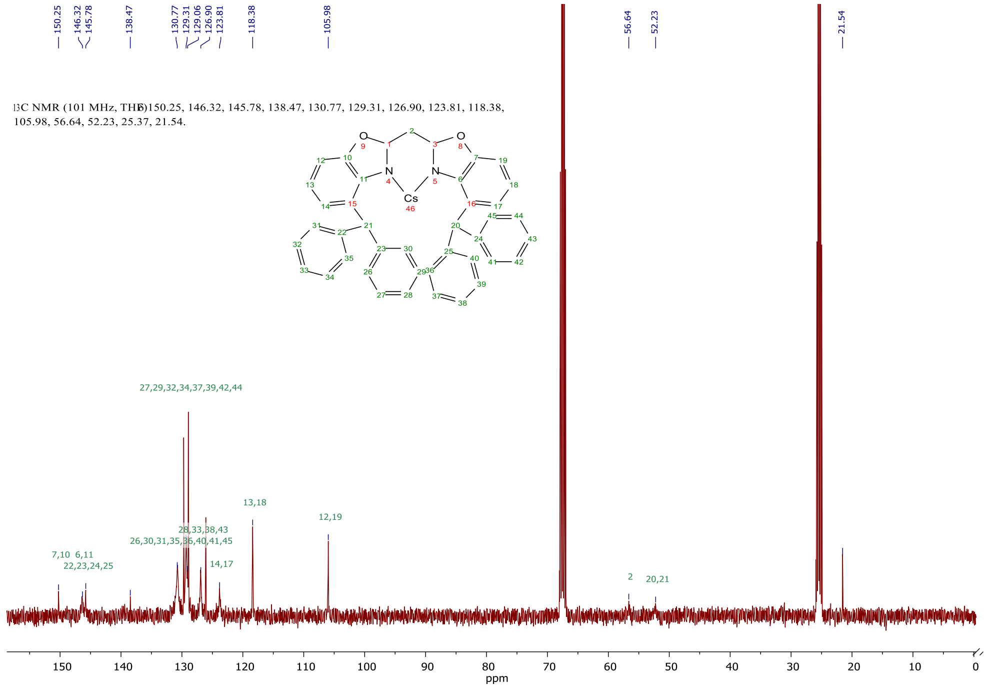

Figure S73. ^{13}C NMR spectrum of **10** in [D8]THF.

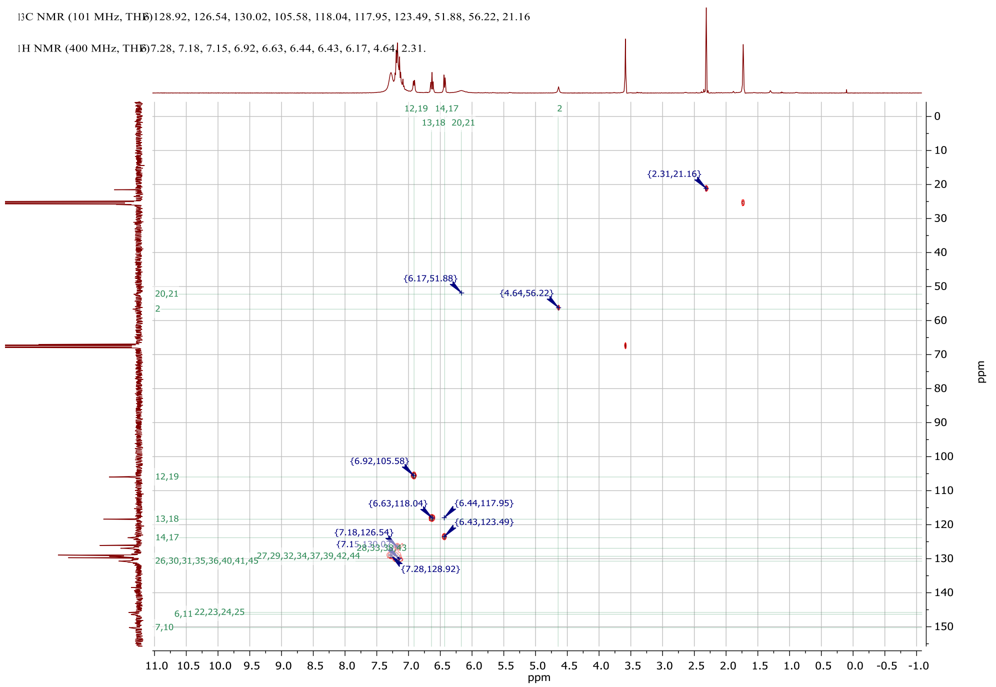

Figure S74. $^{1}H,^{13}C$ HSQC spectrum of **10** in [D8]THF.

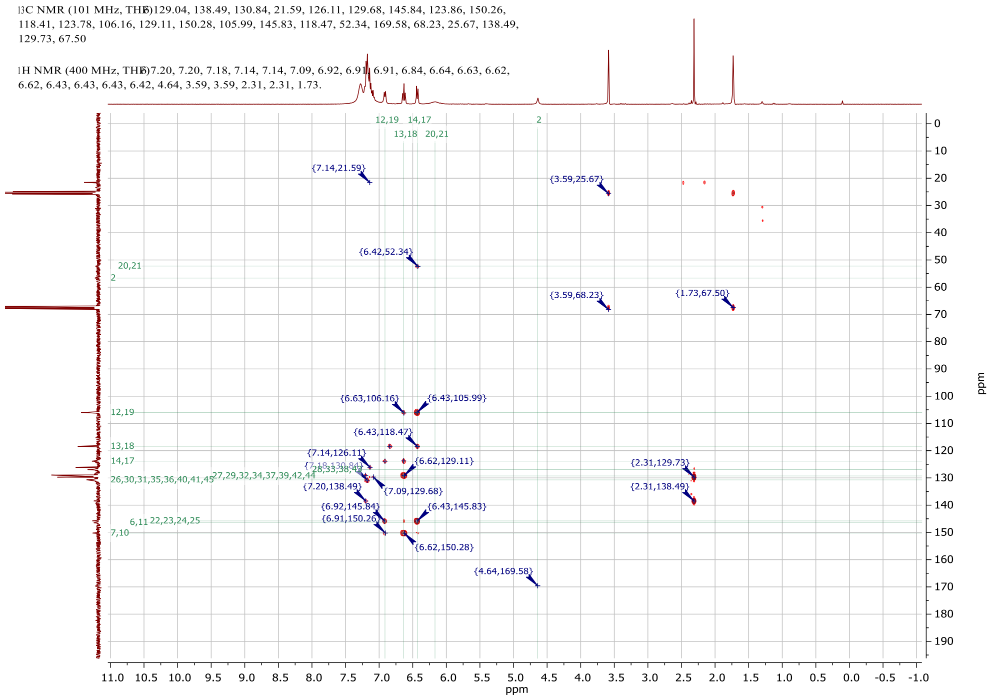

Figure S75. $^{1}H,^{13}C$ HMBC spectrum of **10** in [D8]THF.

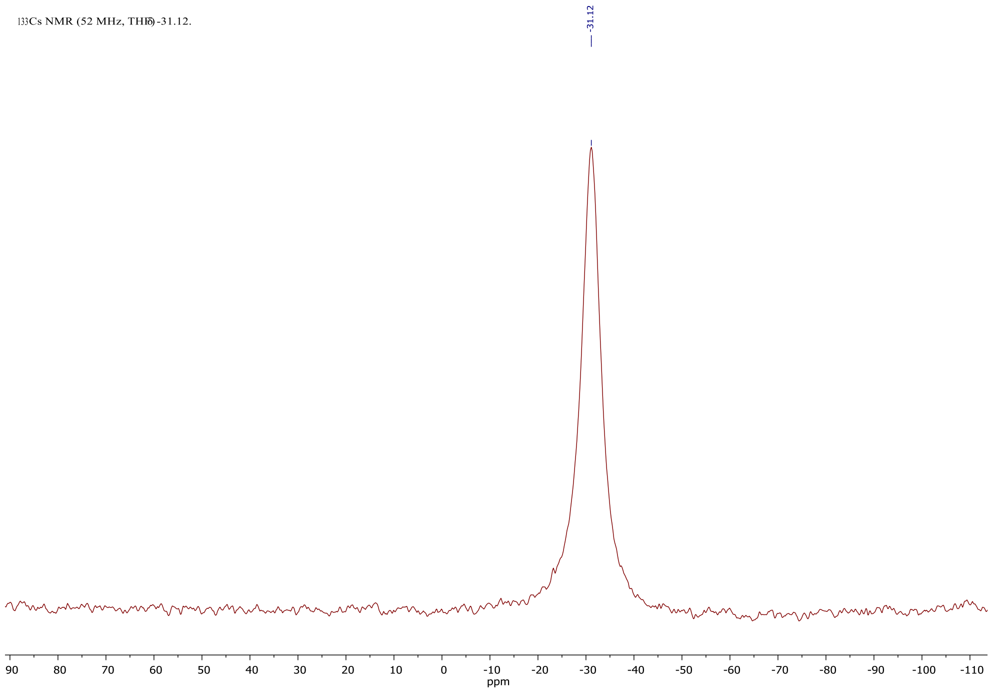

Figure S76. ^{133}Cs NMR spectrum of **10** in [D$_8$]THF.

Mass spectrometry

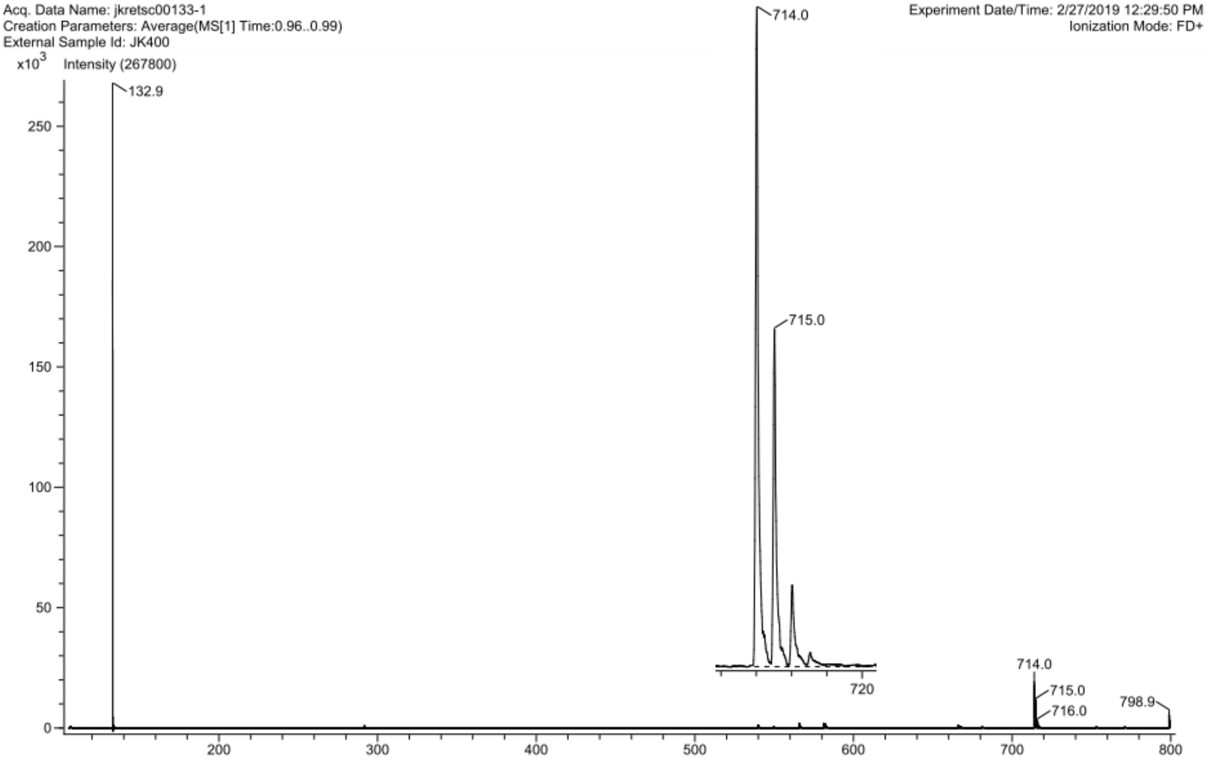

Figure S77. Mass spectrum of **10** (LIFDI[+], THF) *m/z* (%): 714.0 (7) [*M*]$^+$, 132.9 (100) [Cs]$^+$.

Compound 11: Synthesis and Analytical Data

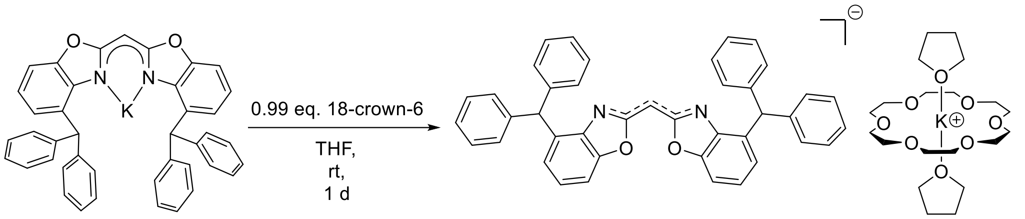

Synthesis of [{(THF)$_2$K@(18-crown-6)}{bis(4-benzhydryl-benzoxazol-2-yl) methanide}] (11)): Bis(4-benzhydryl-benzoxazol-2-yl)methane (146 mg, 251 µmol, 1.00 eq.) was dissolved in THF (6 mL). Potassium hydride (18.3 , 456 µmol, 1.82 eq.) was added to the slightly yellow solution at room temperature. The reaction mixture turned immediately red while the formation of hydrogen gas was observed. After the mixture was stirred for 1 d, it was filtered by a syringe equipped with a glass fiber filter and 18-crown-6 (65.6 mg, 248 µmol, 0.99 eq.) was added to the obtained dark red solution. Within seconds after addition a strong blue fluorescence was noticed. The solution was stirred at ambient temperature for another day, volatiles were removed under reduced pressure and **9** was isolated as a dark-red solid. Crystals (~20 mg) suitable for single X-ray diffraction experiments were grown by vapor diffusion of pentane (1.2 mL) into a THF (0.8 mL) solution. Yield: 245 mg (95%).

^{1}H NMR (300 MHz, [D$_8$]THF, 298 K): 7.18-7.12 (m, 12 H, 18-H, 19-21-22- 24-25-27-28-31-32-34- 35- 37- 38- 40-H, 41-), 7.11-7.04 (m, 4 H, 20-26- 33-39-), 6.89 (d, 2 H, $^3J_{HH}$ = 6.4 Hz, 5-H, 11-H), 6.57 (d, 2 H, $^3J_{HH}$ = 6.2 Hz, 3-H, 13-H), 6.50 (dd, 2 H, $^3J_{HH}$ = 7.8 Hz, 4-H, 12-H), 6.20 (s, 2 H, 16-H, 29-H), 4.59 (s, 1 H, 8-H), 3.62 (m, 8 H, O-CH_2 (THF), 3.34 (m, 24 H, 18-crown-6), 1.78 (m, 8 H, CH_2 (THF)) ppm.

^{1}H NMR (400 MHz, C_6D_6, 298 K): 7.42 (m, 8 H, 18-H, 22- 24-28-31-35- 37- 41-), 7.14-7.12 (m, 10 H, 3-H, 13-H, 19-H21-H 25-H27- 32-H34-H38-H40-H), 7.05 (d, 2 H, $^3J_{HH}$ = 7.0 Hz, 20-H, 26- 33-39-), 7.01 (d, 2 H, $^3J_{HH}$ = 6.6 Hz, 5-H, 11-H), 6.76(dd, 2 H, $^3J_{HH}$ = 7.7 Hz, 4-H, 12-H), 6.66 (s, 2 H, 16-H, 29-H), 5.29 (s, 1 H, 8-H), 3.57 (m, 8 H, O-CH_2 (THF), 2.95 (m, 24 H, 18-crown-6), 1.42 (m, 8 H, CH_2 (THF)) ppm.

^{13}C{^{1}H} NMR (75 MHz, [D$_8$]THF, 298 K): δ = 170.44 (7-C, 9-C), 146.51 (17-3- 30-36-), 130.51 (18-22- 24-28-31-35-37-41-), 128.52 (19-21- 25-27- 32-34-38-40-), 126.17 (20-26- 33-39-), 123.08 (3-C, 13-C), 116.38 (4-C, 12-C), 105.22 (5-C, 11-C), 70.97 (18-crown-6), 68.27 (O-CH$_2$ (THF), 51.68 (16-C, 29-C), 26.43 (CH$_2$ (THF)) ppm. 1-2-C, 6-C, 10-C, 14-C, -
^{13}C{^{1}H} NMR (101 MHz, C_6D_6, 298 K): δ = 169.82 (7-C, 9-C), 151.11 (6-C, 10-C), 145.87 (17-3- 30-36-), 130.18 (18-22-24-28-31-35-37- 41-), 128.35 (19-21- 25-27- 32-34-38-40-), 129.17 (2-C, 14-C), 126.00 (20-26- 33-39-), 123.59 (3-C, 13-C), 117.48 (4-C, 12-C), 105.84 (5-C, 11-C), 69.86 (18-crown-6), 68.84 (O-CH$_2$ (THF), 52.89 (8-C), 51.17 (16-C, 29-C), 25.84 (CH$_2$ (THF)) ppm. (1--could not be observed.
MS (ESI[−], THF) *m/z* (%): 581.2 (100) [H2BZH]$^-$.
HR-MS (ESI[−], THF) *m/z*: 581.2213 (cal. 581.2235 for [H2BZH]$^-$, $C_{41}H_{29}N_2O_2$).

Mass spectra **11** + **D_2O**:
MS (ESI[−], THF) *m/z* (%): 581.2 (100) [H2BZH]$^-$;
HR-MS (ESI[−], THF) *m/z*: 581.2240 (cal. 581.2235 for [($^{4\text{-BzhH2}}$BoxCH)]$^-$, $C_{41}H_{29}N_2O_2$), 582.2296 (cal. 582.2297 for [($^{4\text{-BzhH2}}$BoxCD)]$^-$, $C_{41}H_{28}DN_2O_2$)).

Elemental analysis in % (calculated) $C_{61}H_{69}KN_2O_{10}$ (1029.33 g/mol): C 69.44 (71.18), H 6.28 (6.76), N 2.83 (2.72) (The deviation is due to partial loss of THF in the drying process).

NMR spectroscopy

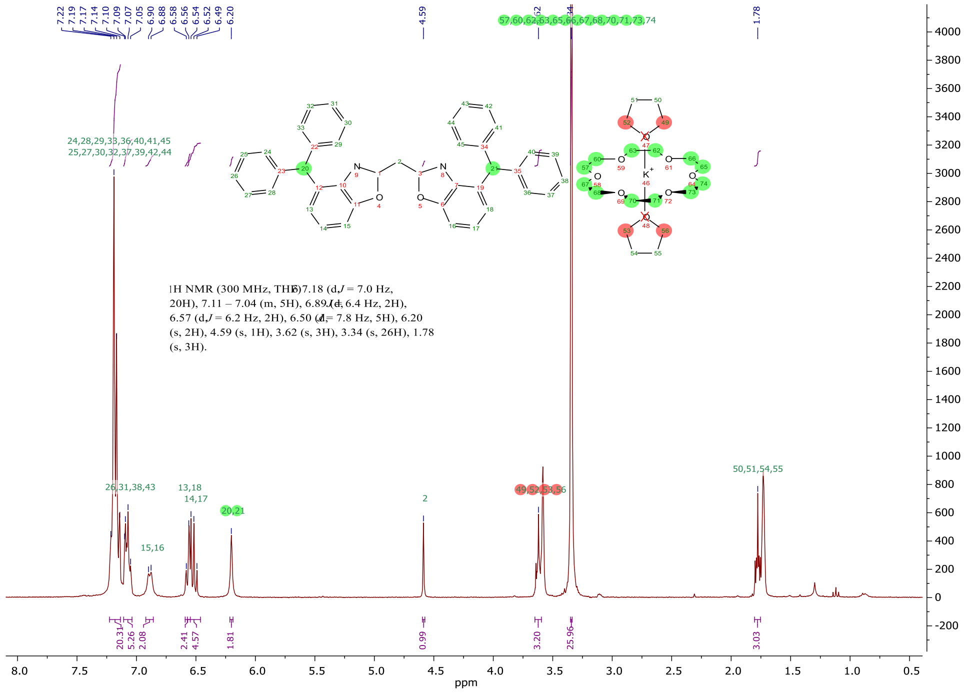

Figure S78. ^{1}H NMR spectrum of **11** in [D$_8$]THF.

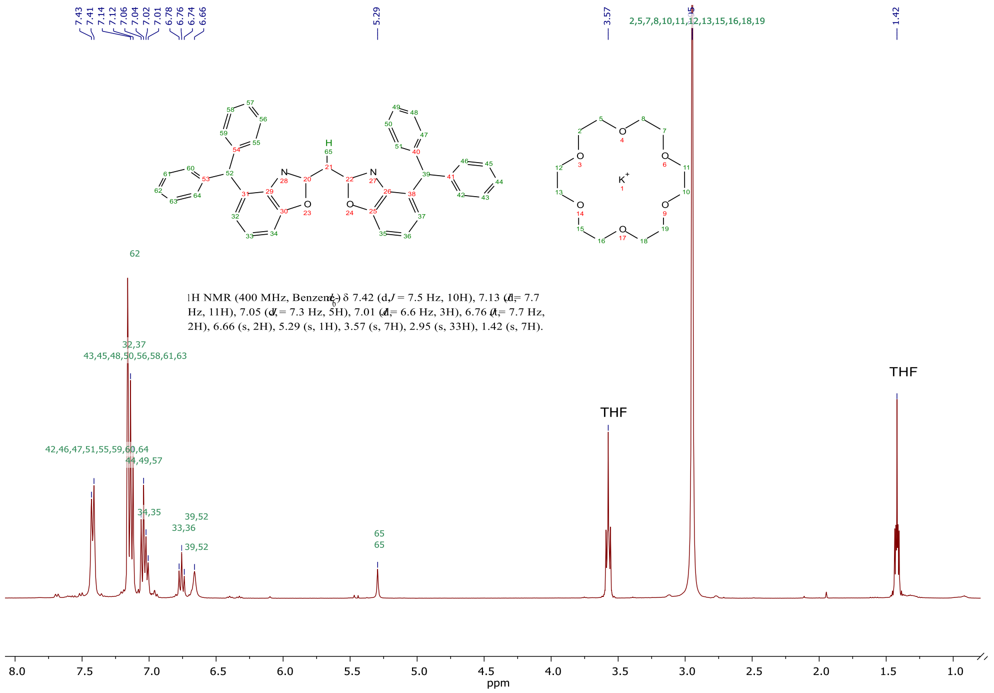

Figure S79. ^{1}H NMR spectrum of **11** in C_6D_6.

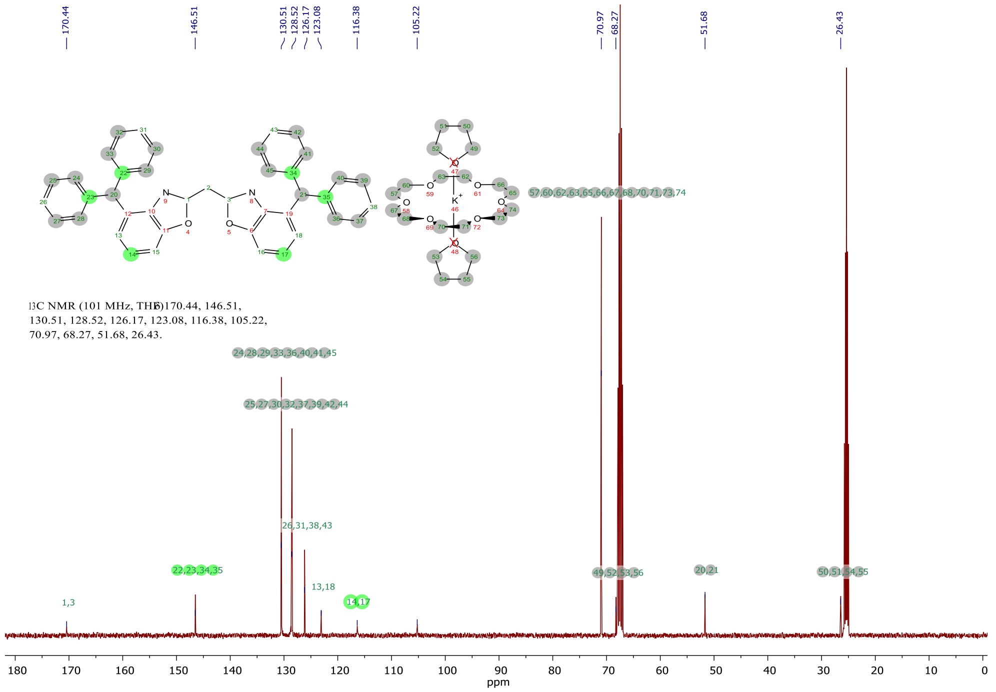

Figure S80. ^{13}C NMR spectrum of **11** in [D$_8$]THF.

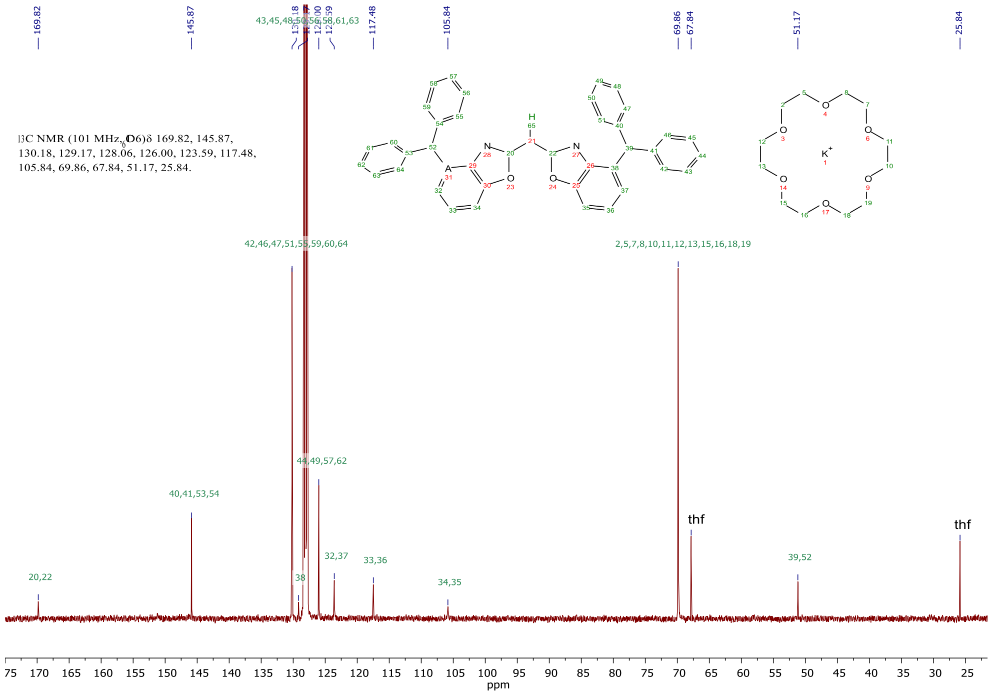

Figure S81. ^{13}C NMR spectrum of **11** in C_6D_6.

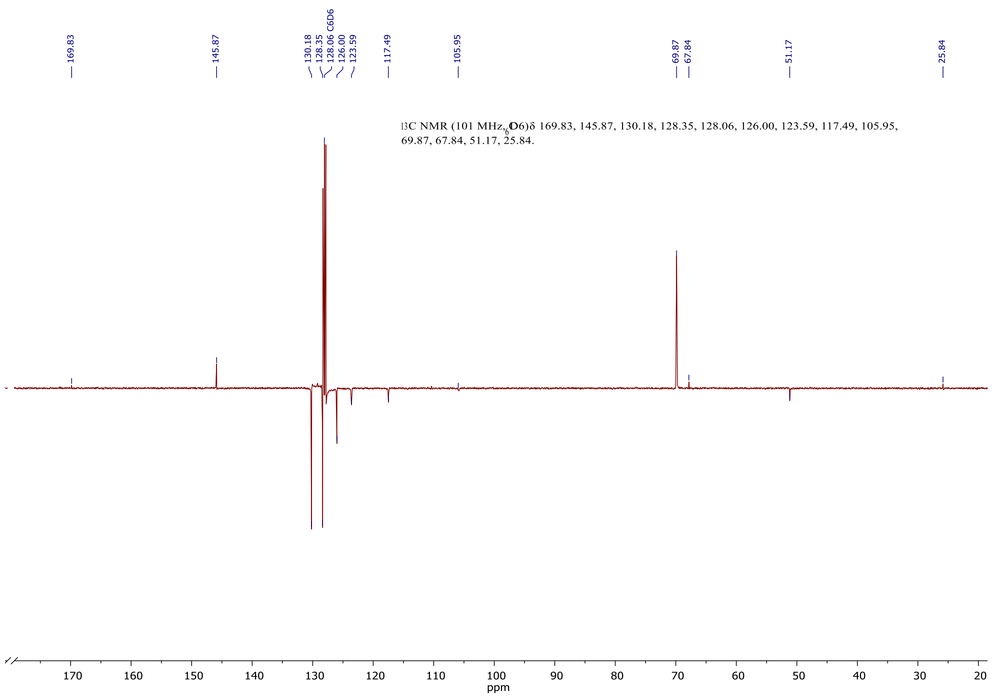

Figure S82. ^{13}C(ATP) NMR spectrum of **11** in C_6D_6.

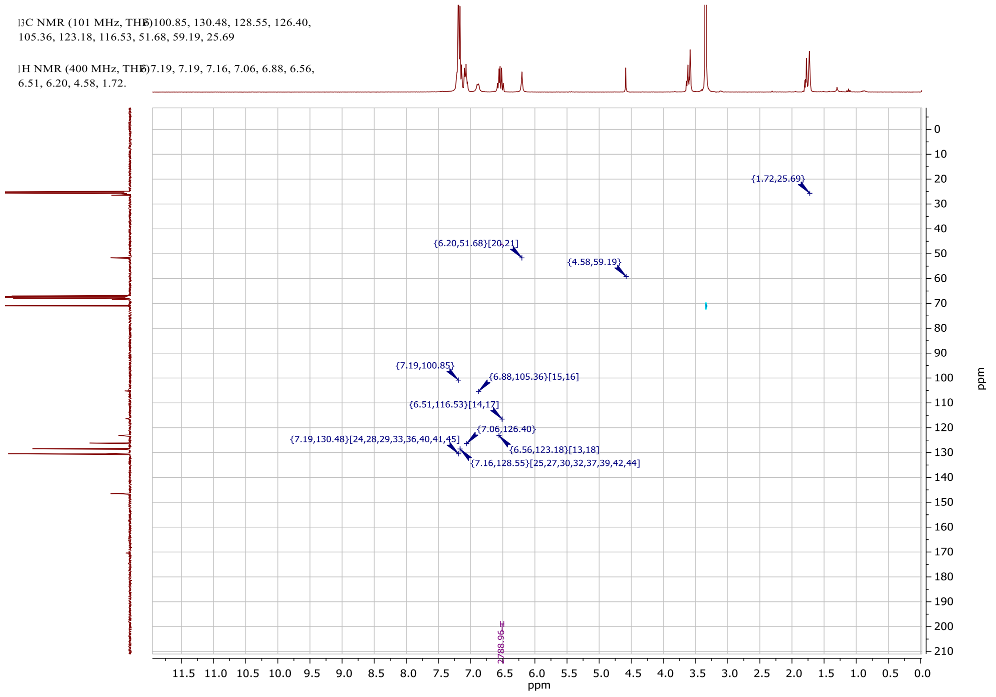

Figure S83. ^{1}H,^{13}C HSQC spectrum of **11** in [D$_8$]THF.

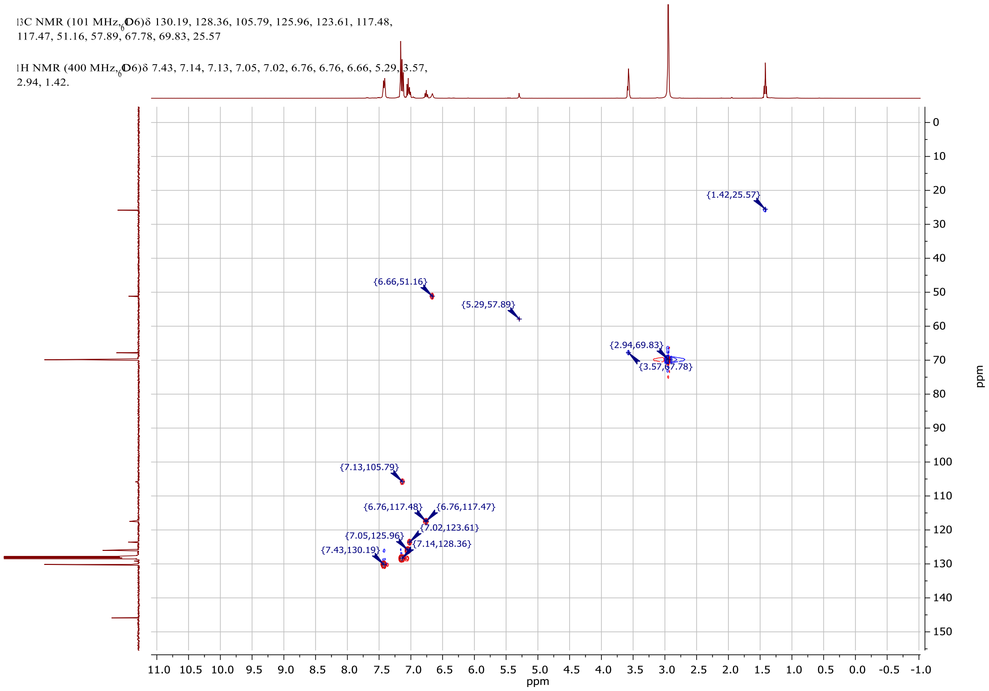

Figure S84. $^{1}H,^{13}C$ HSQC spectrum of **11** in C_6D_6.

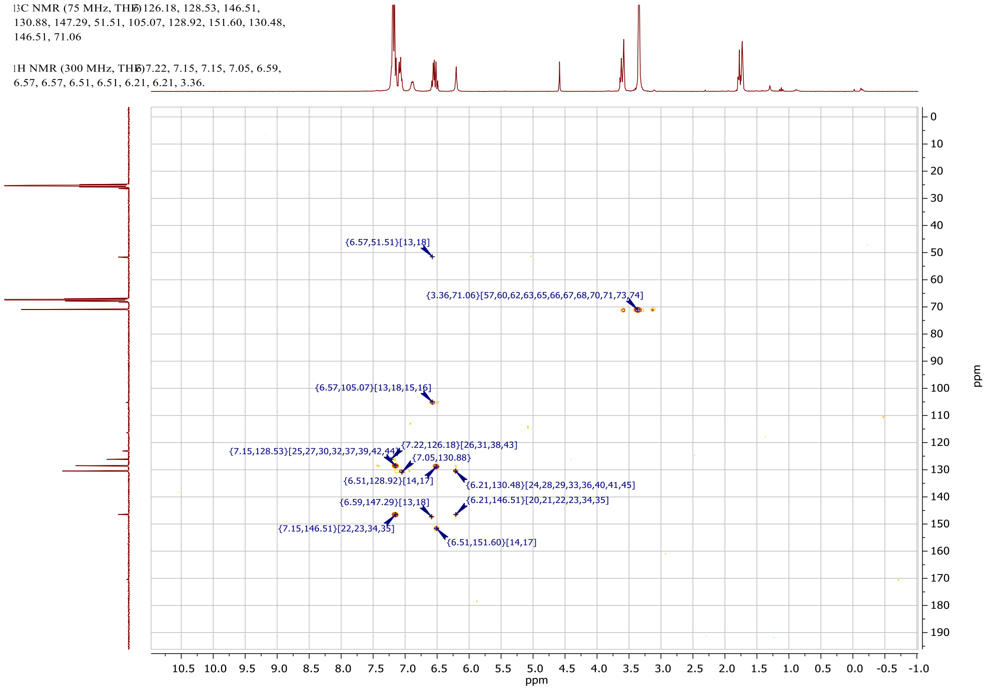

Figure S85. $^{1}H,^{13}C$ HMBC spectrum of **11** in $[D_8]THF$.

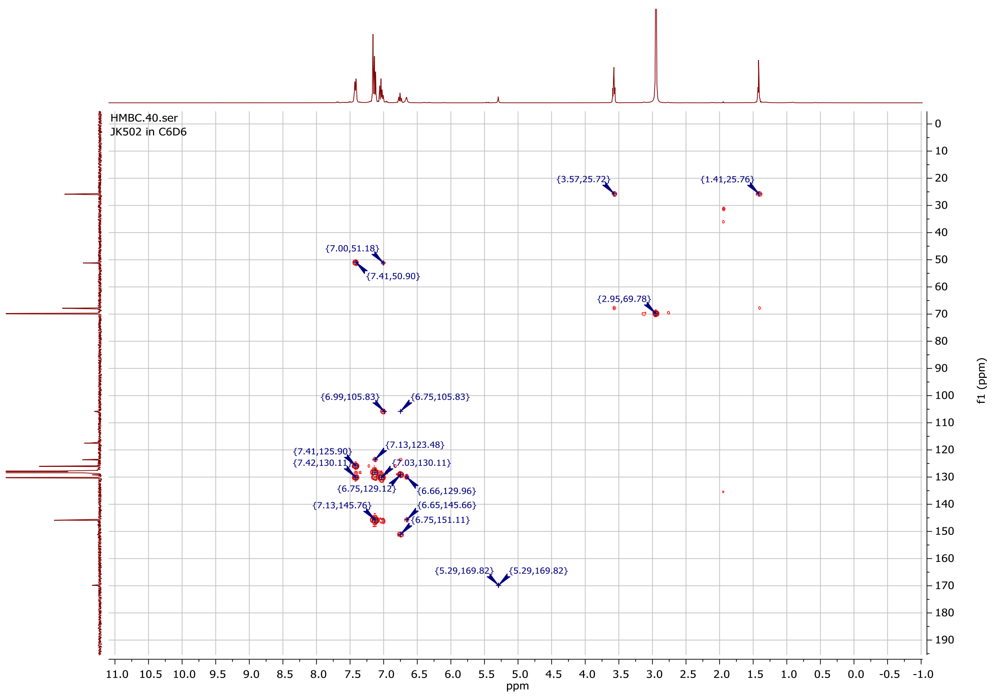

Figure S86. $^{1}H,^{13}C$ HMBC spectrum of **11** in C_6D_6.

Mass spectrometry

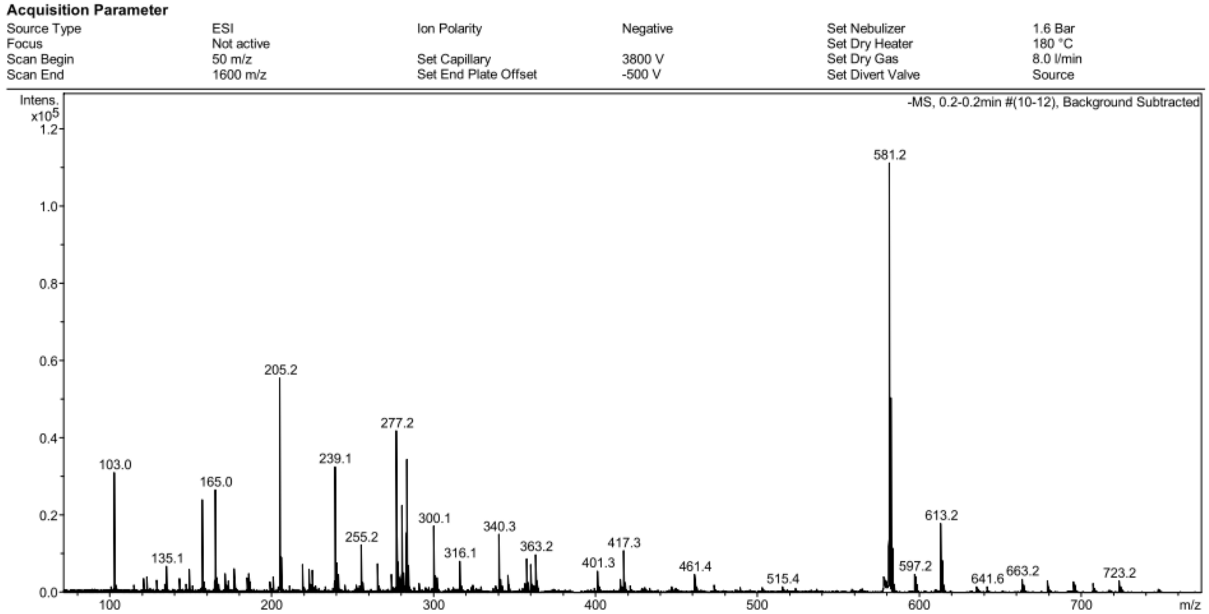

Figure S87. Mass spectrum of **11** (ESI[−], THF) *m/z* (%): 581.2 (100) [(${}^{4\text{-BzhH2}}$BoxCH)]$^-$. HR-MS (ESI[−], THF) *m/z*: 581.2213 (cal. 581.2235 for [(${}^{4\text{-BzhH2}}$BoxCH)]$^-$, $C_{41}H_{29}N_2O_2$).

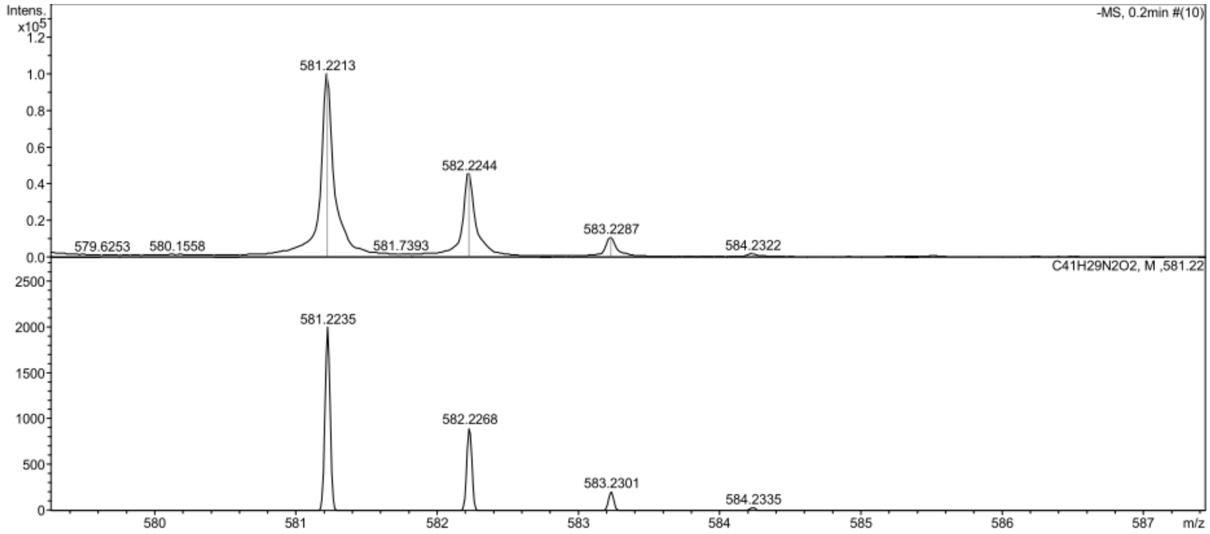

Figure S88. Mass spectrum HR-MS (ESI[−], THF) *m/z*: 581.2213 (cal. 581.2235 for [(${}^{4\text{-BzhH2}}$BoxCH)]$^-$, $C_{41}H_{29}N_2O_2$).

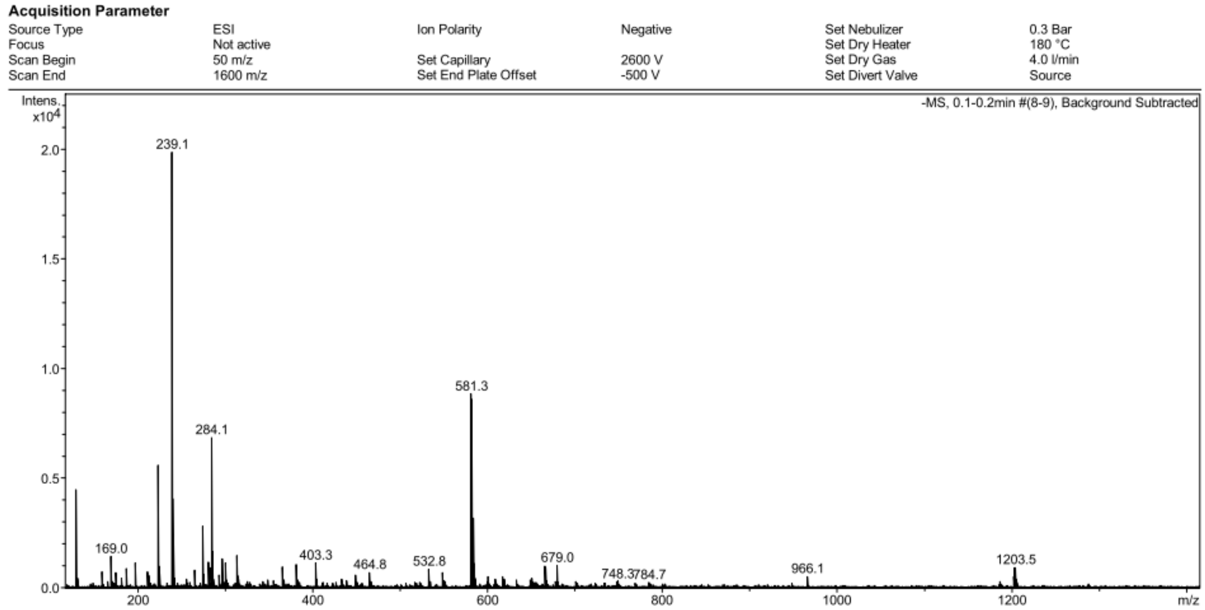

Figure S89. Mass of **11** + **D_2O** MS (ESI[−], THF) *m/z* (%): 581.2 (100) [($^{4\text{-BzhH2}}$BoxCH)]$^-$.

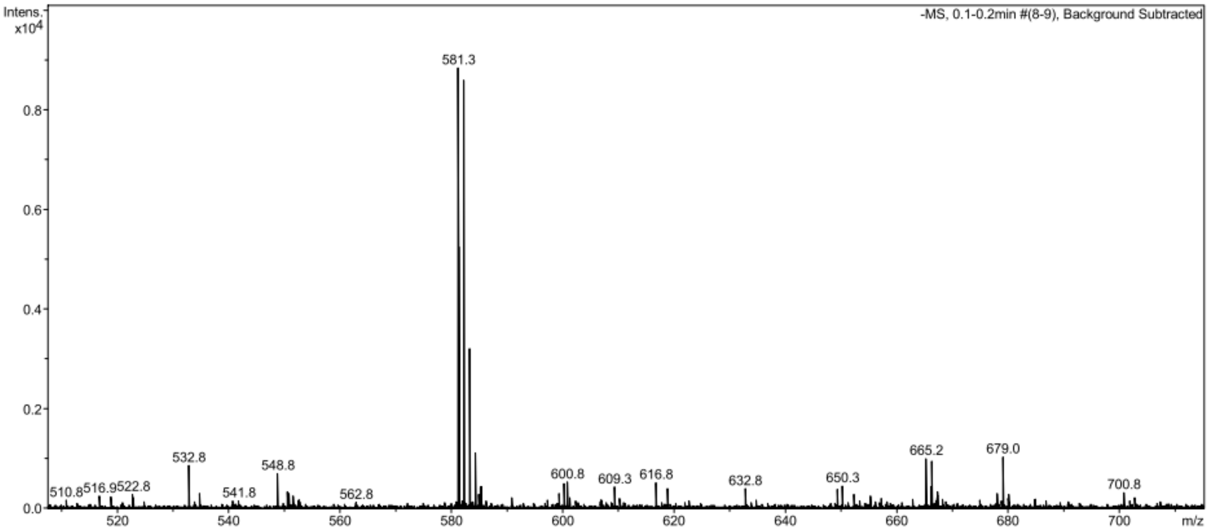

Figure S90. Mass spectrum HR-MS (ESI[−], THF) *m/z*: 581.2240 (cal. 581.2235 for [($^{4\text{-BzhH2}}$BoxCH)]$^-$, $C_{41}H_{29}N_2O_2$), 582.2296 (cal. 582.2297 for [($^{4\text{-BzhH2}}$BoxCD)]$^-$, $C_{41}H_{28}DN_2O_2$)).

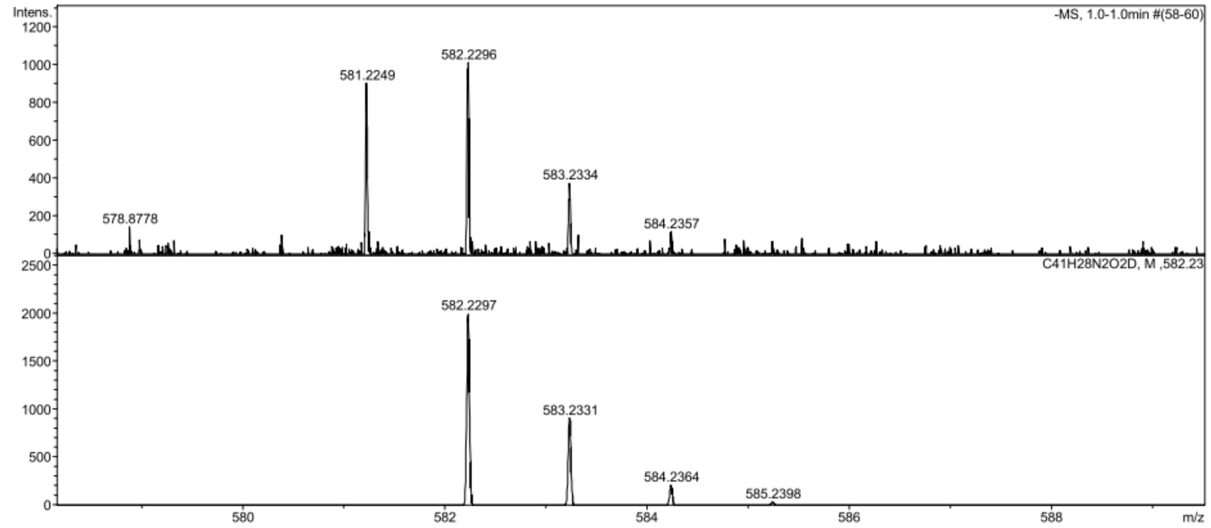

Figure S91. Mass spectrum HR-MS (ESI[−], THF) *m/z*: 582.2296 (cal. 582.2297 for $[(^{4\text{-BzhH2}}\text{BoxCD})]^{-}$, $C_{41}H_{28}DN_2O_2$)).

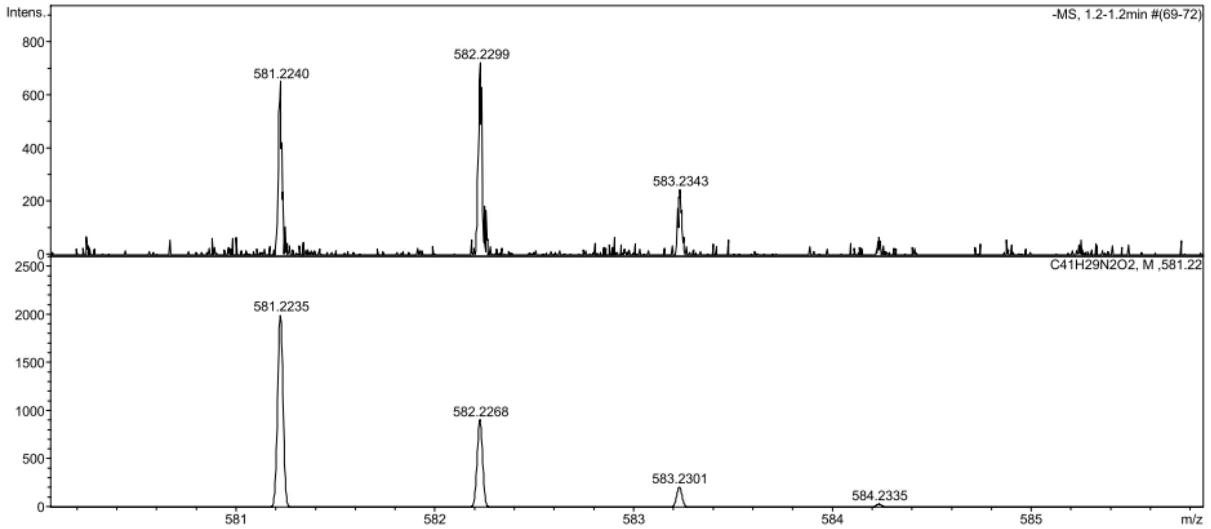

Figure S92. Mass spectrum HR-MS (ESI[−], THF) *m/z*: 581.2240 (cal. 581.2235 for $[(^{4\text{-BzhH2}}\text{BoxCH})]^{-}$, $C_{41}H_{29}N_2O_2$).

Compound 12: Synthesis and Analytical Data

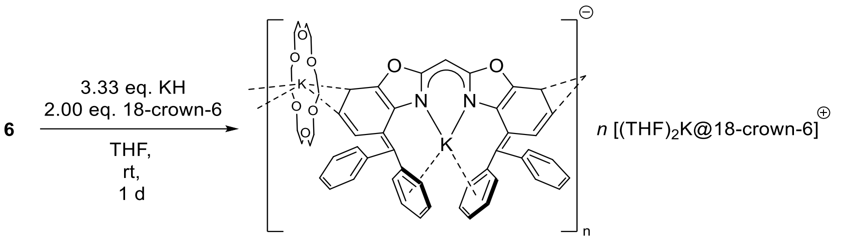

Synthesis of [{(THF)$_2$K@(18-crown-6)}{K@(18-crown-6)K($^{4\text{-Bzh}}$BoxCH)}]$_n$ ($n\rightarrow\infty$) (12): Bis(4-benzhydryl-benzoxazol-2-yl)methane (220.6 mg, 379 µmol, 1.00 eq.) was dissolved in THF (2 mL). Potassium hydride (50.6 mg, 1.26 mmol, 3.33 eq.) and 18-crown-6 (200.0 mg, 757 µmol, 2.00 eq.) were added to the somewhat yellow solution at room temperature. The reaction mixture turned immediately red, while the formation of hydrogen gas was noticed. After the mixture had been stirred for at least 24 h, a red solution with a strong blue fluorescence and dark-red solid was observed. The solution was removed via syringe, and the dark-red solid was washed with pentane (3 × 3 mL). Afterwards the obtained solid was dried under reduced pressure. Dark purple crystals suitable for single XRD experiments were grown out of a saturated THF solution at −30°C after 5 d. Yield: 245 mg (95%).

Elemental analysis in % (calculated) $C_{73}H_{91}K_3N_2O_{16}$ (1369.82 g/mol): C 62.34 (64.01), H 6.67 (6.70), N 2.17 (2.05) (The deviation is due to partial loss of THF in the drying process).

Further analyses of **12** were challenging due to its low solubility in most solvents (toluene, benzene or THF). To find further evidence for the synthesis of the triply deprotonated anion ($^{4\text{-Bzh}}$BoxCH), suspensions of the dark red precipitate (**12**) were once again protonated (excess ≈20 eq. H_2O or D_2O) in small scale (NMR experiment) with *a*) H_2O + [D$_8$]THF, *b*) D_2O + [D$_8$]THF and *c*) D_2O + THF. Measured ^{1}H NMR (**Figure S93** *a*) + *b*)) as well as ^{2}H NMR spectra (**Figure S93** *c*)) were compared to a ^{1}H NMR spectra of **11** in *d*) [D$_8$]THF, *e*) H_2O + [D$_8$]THF and *f*) D_2O + [D$_8$]THF. The successful reprotonation of the benzylic position (C9) was observed in a) as a singlet at 6.24 ppm while in b) the deuteration led to a significantly reduced signal.

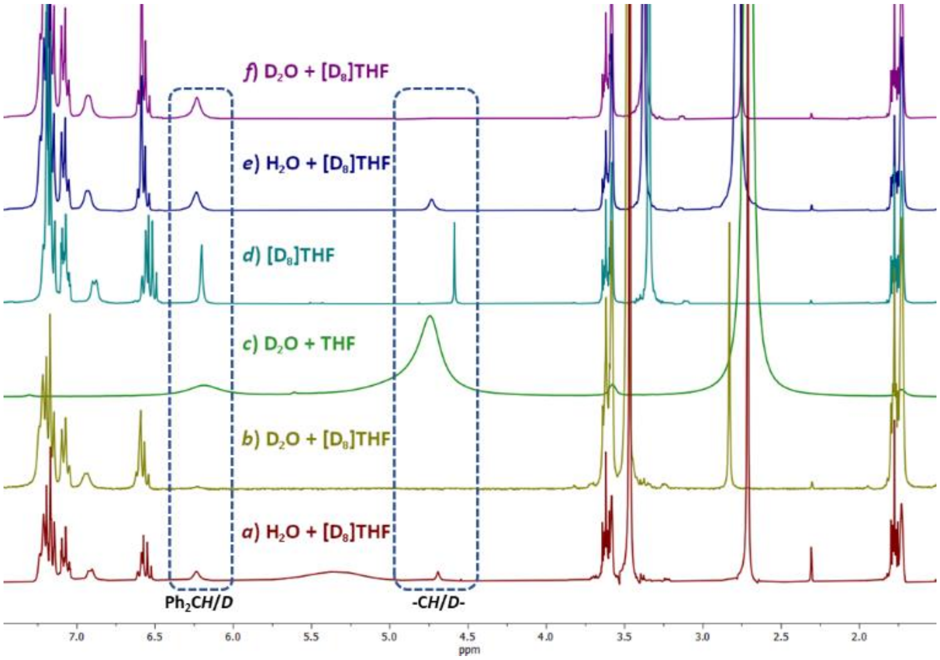

Figure S93. ^{1}H NMR spectra of **12** and *a*) H_2O + [D_8]THF, *b*) D_2O + [D_8]THF as well as ^{2}H NMR spectra *c*) D_2O + THF. ^{1}H NMR spectra of complex **11** and *d*) [D_8]THF , *e*) H_2O + [D_8]THF and *f*) D_2O + [D_8]THF.

The benzylic positions of complex **11** are not influenced by the addition of *e*) H_2O or *f*) D_2O due to its the pK_a value[1][1] of about 33. Moreover, the protonation by D_2O and consequently deprotonation of *c*) **12** and was confirmed via ^{2}H NMR spectra showing a broad singlet at 6.20 ppm. The singlet of the backbone (-C*H*-) was detected at 4.73 ppm in *a*) and *e*) but vanished in *b*) and *f*) as soon as D_2O was added. These observations combined with the distinctive singlet in ^{2}H NMR spectra *c*) and at 4.73 ppm hint at an equilibrium between water and the linking methylene or methanide unit.

Mass spectra **12** + **D_2O**:

MS (ESI[−], THF) *m/z* (%): 582.2 (19) $[(^{4\text{-BzhH2}}\text{BoxCD})]^-$, 583.2 (100) $[(^{4\text{-BzhHD}}\text{BoxCD})]^-$, 584.2 (44) $[(^{4\text{-BzhD2}}\text{BoxCD})]^-$;
HR-MS (ESI[−], THF) *m/z*: 582.2274 (cal. 582.2282 for, $C_{41}H_{28}DN_2O_2$), 583.2335 (cal. 583.2380 for $[(^{4\text{-BzhHD}}\text{BoxCD})]^-$, $C_{41}H_{27}D_2N_2O_2$, 584.2380 (cal. 584.2417 for $[(^{4\text{-BzhD2}}\text{BoxCD})]^-$, $C_{41}H_{26}D_3N_2O_2$).

Mass spectrometry

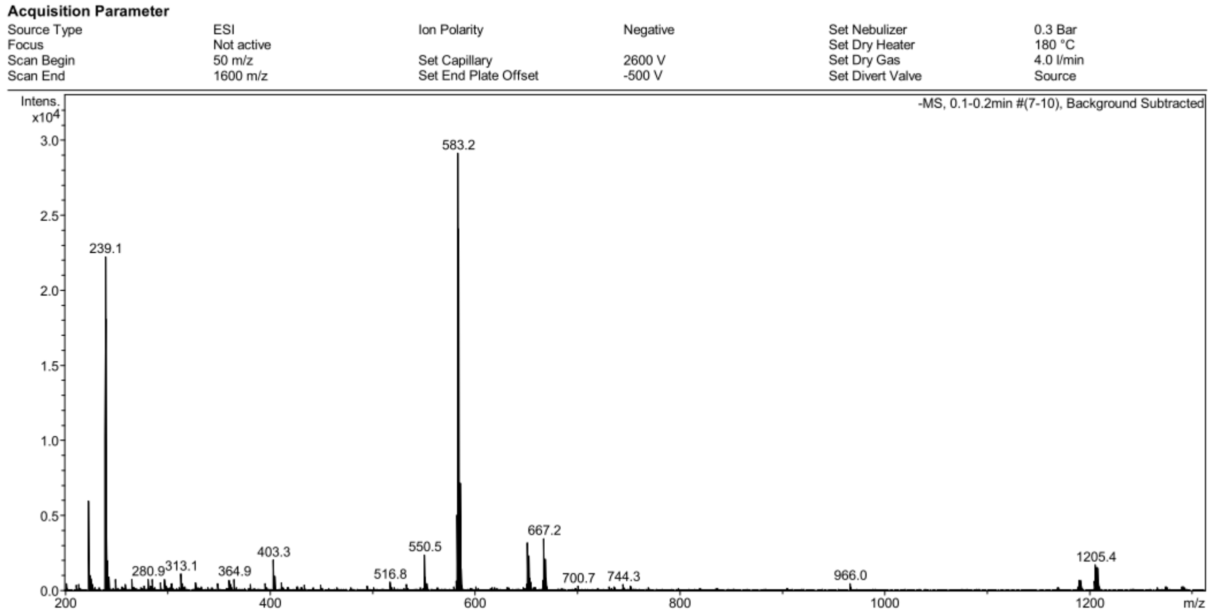

Figure S94. Mass spectrum of **12** + **D_2O** MS (ESI[−], THF) *m/z* (%): 582.2 (19) [($^{4\text{-BzhH2}}$BoxCD)]$^-$, 583.2 (100) [($^{4\text{-BzhHD}}$BoxCD)]$^-$, 584.2 (44) [($^{4\text{-BzhD2}}$BoxCD)]$^-$.

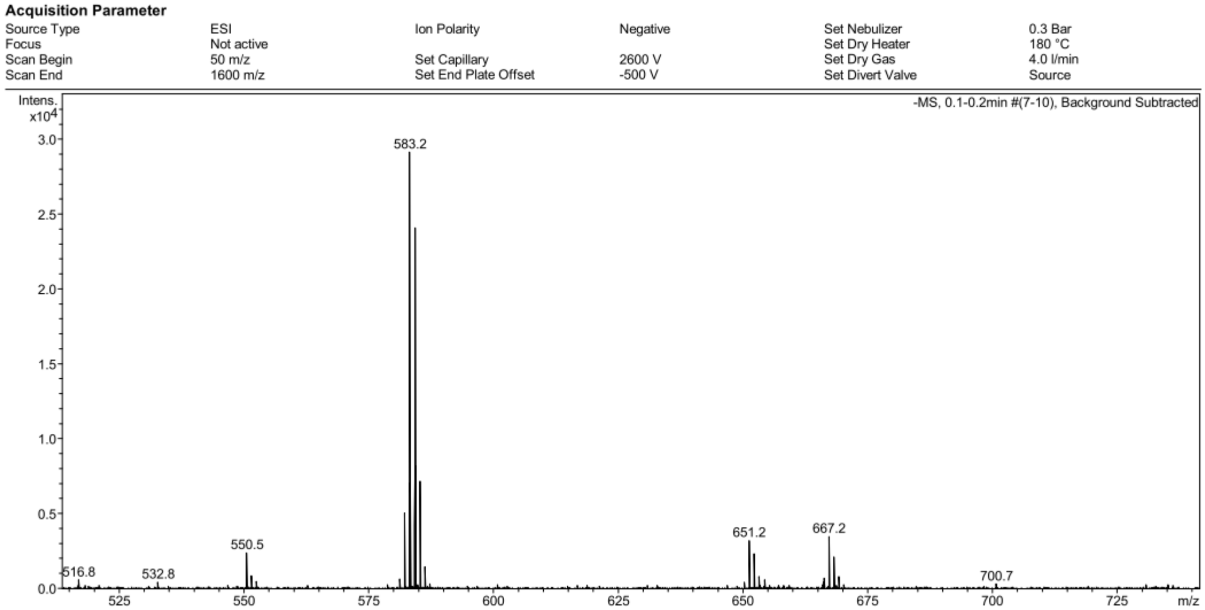

Figure S95. Mass spectrum of **12** + **D_2O** MS (ESI[−], THF) *m/z* (%): 582.2 (19) [($^{4\text{-BzhH2}}$BoxCD)]$^-$, 583.2 (100) [($^{4\text{-BzhHD}}$BoxCD)]$^-$, 584.2 (44) [($^{4\text{-BzhD2}}$BoxCD)]$^-$.

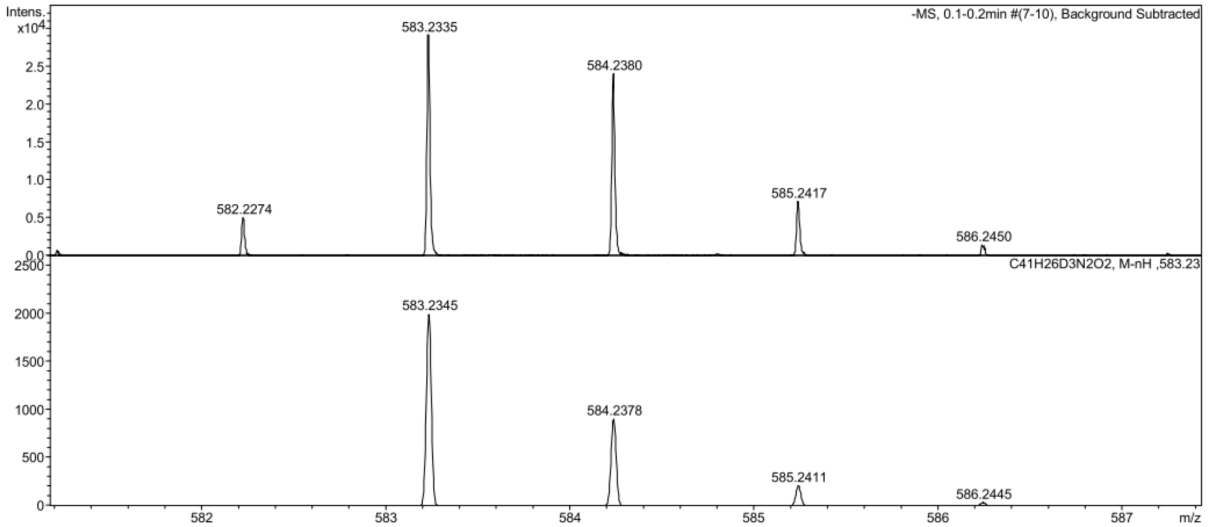

Figure S96. Mass spectrum HR-MS (ESI[−], THF) *m/z*: 583.2335 (cal. 583.2380 for [($^{4\text{-BzhHD}}$BoxCD)]$^{-}$, $C_{41}H_{27}D_2N_2O_2$.

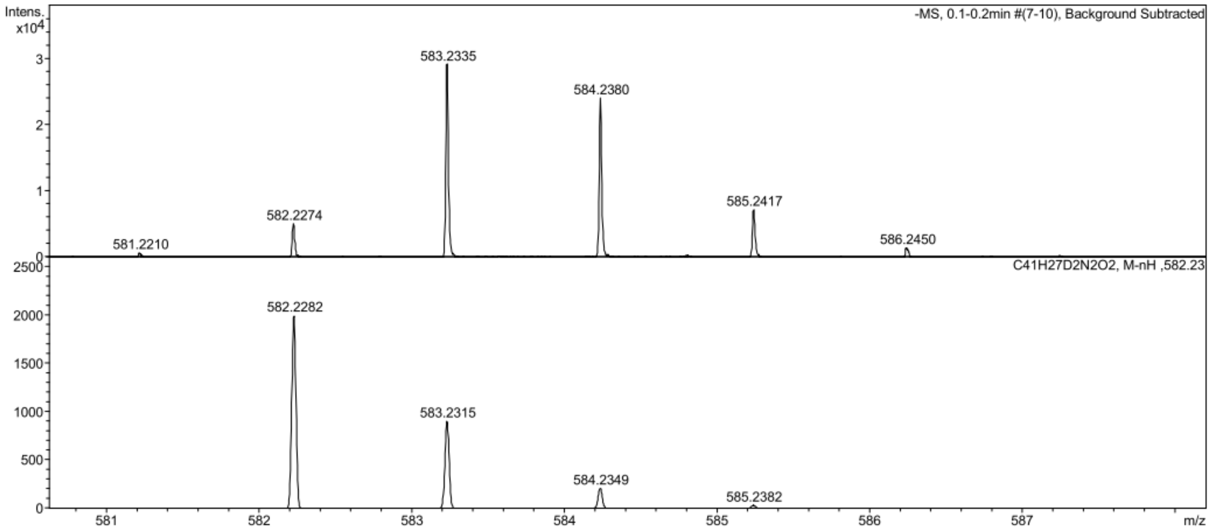

Figure S97. Mass spectrum HR-MS (ESI[−], THF) *m/z*: 582.2274 (cal. 582.2282 for, $C_{41}H_{28}DN_2O_2$).

Experimental setup and methodology for pK_a determination

The experimental setup and methodology for the pK_a determination of $^{4\text{-BzhH2}}Box_2CH_2$ (**6**) in acetonitrile was essentially the same as described in detail in previous publications.[2] A brief description will follow.

The pK_a determination in acetonitrile is based on the determination of differences of pK_a values of two acids. One compound is a reference acid with a previously known pK_a value and the other acid is $^{4\text{-BzhH2}}Box_2CH_2$ (**6**). This compound, as well as the references are separately titrated in order to obtain the UV-Vis spectra of the free acids and their deprotonated forms. Then the same titration is then done with a mixture of the compound (**6**) and a reference acid in the same solution. After mathematically treating the spectral data obtained from the titration of the mixture at multiple wavelengths using multilinear regression analysis the dissociation levels (α) of both acids in all the mixtures formed during titration are calculated and are then in turn used to calculate the differences of pK_a values (ΔpK_a) of $^{4\text{-BzhH2}}Box_2CH_2$ (**6**) and the used reference acids according to the following equation:

$$\Delta \mathrm{p}K_a = \log\frac{\alpha_1(1-\alpha_2)}{\alpha_2(1-\alpha_1)}$$

The pK_a value of $^{4\text{-BzhH2}}Box_2CH_2$ (**6**) in acetonitrile is estimated as a result of ΔpK_a measurements against four different reference acids with previously published pK_a values in literature.[2a,3] All pK_a determination results are presented in Table S1.

An Agilent Cary 60 spectrophotometer connected with optical fibre cables to an external cell compartment inside a MBraun Unilab glovebox filled with 99.999% pure argon was used for the spectrophotometric titrations. This setup ensured that during all titrations the moisture and oxygen contents in the argon atmosphere inside the glovebox were always under 10 ppm.

Trifluoromethanesulfonic acid (Aldrich, 99+%) and *tert*-butylimino-tris(pyrrolidino)phosphorane (Aldrich, ≥97%) were used to prepare the acidic and basic titrant solutions, respectively. For the titration involving 4-CN-C_6F_4-NH_2, a stronger Phosphazene base P_2-Et (CAS number 165535-45-5, Sigma-Aldrich, 98%) was used as the basic titrant. The concentrations of the titrant solutions were in the range of 1 - $4{\cdot}10^{-3}$ mol·L^{-1} and the concentrations of the studied and reference acids were between 0.6 - $9{\cdot}10^{-5}$ mol·L^{-1} during the titrations. Acetonitrile (Romil 190 SpS far UV/gradient quality) was used as solvent after drying with molecular sieves (3 Å) for at least 12 hours, which lowered the water content to under 6 ppm.

Table S1. pK_a measurement results in acetonitrile.

Acid	Reference Acid	pK_a (Ref)	Δ pK_a	pK_a (Acid)	Assigned pK_a
**$^{4\text{-BzhH2}}Box_2CH_2$ (6)**	4-CN-C_6F_4-NH_2	28.76	2.20	26.56	**26.59(6)**
	(4-Me-C_6F_4)(C_6H_5)CHCN	26.96	0.40	26.56	
	C_6F_5-NHCOCH$_3$	26.43	−0.17	26.60	
	(C_6H_5)(C_6F_5)CHCN	26.14	−0.50	26.64	

Fluorescence measurements

The fluorescence measurements were carried out by Timo Schillmöller on a Horiba Jobin-Yvon Fluoromax-4 spectrometer.

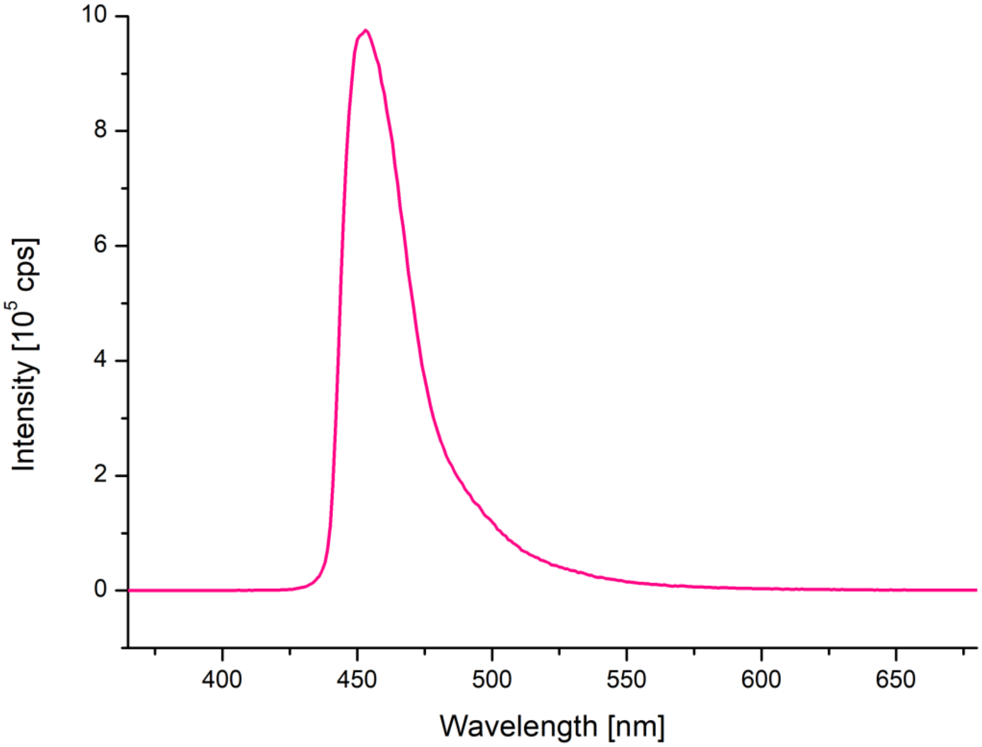

Figure S98. Fluorescence of **12** (1.0 mM) in THF displays a maximum λ_{max} = 454 nm (λ_{ex} = 350 nm).

Computational studies

Computations using density functional theory were performed using ORCA (version 4.1.).[4] All structures were optimized on RI-BP86-D3BJ[5]-def2SVP/J model chemistry[6] in the gas phase followed by a frequency calculation on the same level of theory and thermochemical corrections were taken from these frequency calculations. The influence of the solvent on the anions was completed by single point calculations using the CPCM formalism[7] with THF as solvent. All anionic structural isomers were preoptimized in Avogardo: an open-source molecular builder and visualization tool. Version 1.2.0[8] by UFF[9] except [(*Z*,*Z*)-($^{4\text{-BzhH2}}\text{Box}_2\text{CH}$)]$^-$ (**11h**) that was obtained from single XRD experiments. All structures were verified as true minima on the potential energy surface by the absence of imaginary frequency. Molecular images of **11a** to **11i** were created with IboView v20150427.[10]

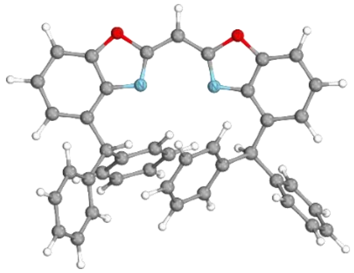

[(*E*,*E*)-($^{4\text{-BzhH2}}\text{Box}_2\text{CH}$)]$^-$ (**11a**)

(G = –1838.81947112 E_h)

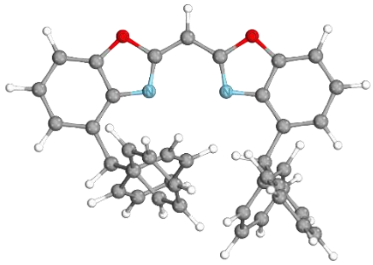

[(*E*,*E*)-($^{4\text{-BzhH2}}\text{Box}_2\text{CH}$)]$^-$ (**11b**)

(G = –1838.82344912 E_h)

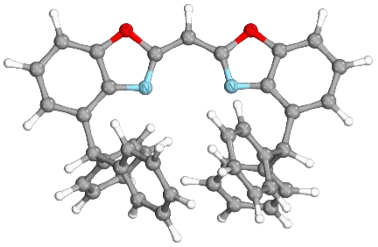

[(*E*,*E*)-($^{4\text{-BzhH2}}\text{Box}_2\text{CH}$)]$^-$ (**11c**)

(G = –1838.80202399 E_h)

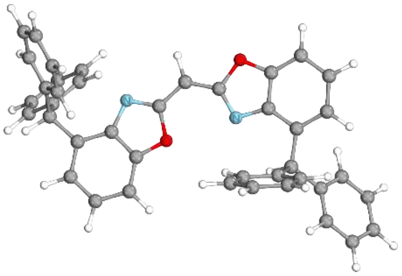

[(*Z*,*E*)-($^{4\text{-BzhH2}}\text{Box}_2\text{CH}$)]$^-$ (**11d**)

(G = –1838.81572179 E_h)

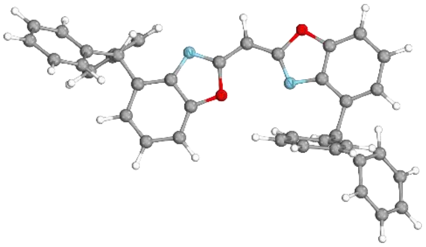

[(*Z*,*E*)-($^{4\text{-BzhH2}}\text{Box}_2\text{CH}$)]$^-$ (**11e**)

(G = –1838.81481865 E_h)

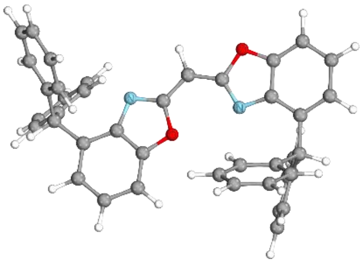

[(*Z*,*E*)-($^{4\text{-BzhH2}}\text{Box}_2\text{CH}$)]$^-$ (**11f**)

(G = –1838.81659250 E_h)

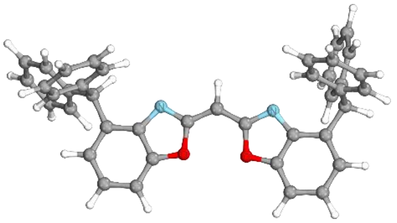

[(*Z*,*Z*)-($^{4\text{-BzhH2}}\text{Box}_2\text{CH}$)]$^-$ (**11g**)

(G = –1838.81589933 E_h)

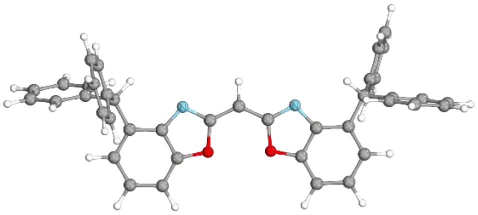

[(*Z*,*Z*)-($^{4\text{-BzhH2}}\text{Box}_2\text{CH}$)]$^-$ (**11h**)

(G = –1838.81756699 E_h)

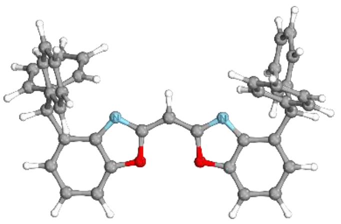

[(*Z*,*Z*)-($^{4\text{-BzhH2}}\text{Box}_2\text{CH}$)]$^-$ (**11i**)

(G = –1838.81127688 E_h)

[(*E*,*E*)-($^{4\text{-BzhH2}}$Box$_2$CH)]$^-$ (11a) (G = –1838.81947112 E_h)

O	11.27910003300764	6.98831665419482	16.37034066260602
N	10.20240522784659	7.72299294792003	14.48286978093241
C	10.06914527701304	6.36390842445571	14.58029303923854
O	11.52206223005848	11.75078115731195	15.51457868016282
N	9.92685903875233	10.64551425194594	14.28811275009763
C	9.38426407892457	5.48454917594334	13.71693030877093
C	9.38520818449969	4.12009230804733	14.07465129571831
H	8.85923094792630	3.39467748972464	13.43361192107960
C	10.05727514038988	3.66135577139691	15.23002456335982
H	10.03854140115567	2.58642739080916	15.46952861421999
C	10.75149521777271	4.54555056470950	16.09588172230550
H	11.26722487861472	4.18448835557550	16.99811286251071
C	10.73309567470946	5.89359589786988	15.74294225435684
C	10.90474184595812	8.09356895083632	15.55093333410857
C	10.86390558013863	10.51976581721690	15.23368849446654
C	11.30410398267251	9.38527847203510	15.94185960488197
C	9.56334053787027	13.99170535676474	12.67129521968315
H	9.01642165659309	14.55198377938455	11.89845506246501
C	8.64824566162164	6.13877285627331	12.55288802055240
H	9.27682384067777	7.01482734046983	12.27496839718649
C	10.96762422217188	12.65017396385847	14.64164965783805
C	10.54007001171612	14.66197824267544	13.43752171525103
H	10.73993994437594	15.72684018357014	13.24048798240774
C	11.26728582500495	13.99704064233153	14.45440050166646
H	12.03188331135807	14.51048810497882	15.05602382319262
C	9.25604060704609	12.62718794491256	12.87097430709328
C	9.98196406240643	11.95701509501225	13.88531698559424
C	7.34645550941222	6.73756326687319	13.07932046883262
C	6.20260816661089	5.94257801210186	13.29908500973285
H	6.23669011384383	4.86788873344125	13.05893094633899
C	5.02655119302860	6.50467740594210	13.82138784343988
H	4.13861580549060	5.87084900897437	13.97781336797634
C	6.13588893105386	8.65914091165573	13.97319186829172
H	6.13070403490471	9.72241254856209	14.26144850669058
C	4.98690420238390	7.87067928657884	14.15338739962298
H	4.06654851411577	8.31364234557100	14.56696155545954
C	8.54442042617093	5.29851278005929	11.28943647262964
C	7.30969387987145	8.10179132974973	13.44079751038227
H	8.22317116419843	8.71154583217970	13.35670088288476
C	7.40122251050628	4.63370850126616	9.22454540613905
H	6.50578963261392	4.67930620355442	8.58405254982472
C	7.41497128701121	5.32922577059664	10.44550869797484
H	6.53767290216857	5.91821784211854	10.74794513264712
C	9.65483805062742	3.85231704229332	9.64740187263133
H	10.54387418702084	3.27786846673871	9.34120333482223
C	8.52010735195853	3.88974868613569	8.81769802975700
H	8.51006596983755	3.34400529792542	7.86093102263841
C	8.17424009684169	11.86174028757035	12.11605254840217
H	7.52983211996792	11.40842980579812	12.90450701135229
C	9.66426217812896	4.54778841735428	10.86542770423405
H	10.55638435776809	4.52048912486717	11.50917298146774
C	8.66836181692201	10.66920307974840	11.28833569641975
C	7.70608299932240	9.83906221385069	10.67429184756595
H	6.63791955644709	10.08326842137721	10.78555185081378
C	8.08779459253922	8.70058638462845	9.95317557991598
H	7.32270714592473	8.05455832930657	9.49783961788961
C	9.44757082820613	8.36202145611233	9.83938805756325
H	9.74361457495572	7.44597569821032	9.30554722293116
C	10.41242670026672	9.18705885995630	10.43776615837094
H	11.47993328030214	8.92564957251487	10.37017308240006
C	10.02644006737884	10.33495375875913	11.15225693020542
H	10.78593458972008	10.96416418740264	11.63607601366132
C	7.29032937070605	12.78680851058247	11.29355649955621
C	7.62787418986817	13.12438618395374	9.96684466670880
H	8.51999250608094	12.66570302932217	9.51370673714306
C	6.83886308546788	14.02182827289701	9.23036927995605
H	7.11908531601719	14.27354861103921	8.19504181072141
C	5.69254311227243	14.59513821354638	9.80784806429901
H	5.06936743576370	15.29471820140182	9.22856628401661
C	5.34528312030269	14.26418856896423	11.12865380884197
H	4.44688877572966	14.70391734477620	11.59039297579196
C	6.14110267455620	13.36971188859491	11.86258287825814
H	5.87400058445198	13.11566969394887	12.90088705964816
H	12.00617267097735	9.51059547097155	16.77400819109904

[(*E*,*E*)-($^{4\text{-BzhH2}}$Box$_2$CH)]$^-$ (11b) (G = –1838.82344912 E_h)

O	10.18780090526187	6.49451825074220	16.88387732374113
N	9.37728781578009	7.20524493035112	14.86250274882119
C	8.86994728779452	5.96390741983466	15.13424886410970
O	11.51610255260932	10.96439783811232	15.53466046407684
N	10.10647110781860	9.89036253071874	14.07480629276993
C	7.97922719051962	5.17110467888461	14.37546035071352
C	7.64480142278946	3.91266198633515	14.92112435134164
H	6.95025572945783	3.26326740074836	14.36229371293967
C	8.16488512012408	3.46323824372400	16.15406909757908
H	7.87255686032610	2.47152605713123	16.53336119377847
C	9.05028846115487	4.26272808402316	16.91986917163608
H	9.45163345797427	3.92210372304189	17.88589178277103
C	9.37744283294655	5.50489928148082	16.38165823064601
C	10.13744975096566	7.52857390251644	15.90584077492716
C	10.76912559622466	9.78952181399975	15.22978897936652
C	10.83863805126865	8.72399326779158	16.14827998152247
C	10.53799370859929	13.02504145831030	12.10032785480779
H	10.27539280114187	13.51673613523039	11.15003915377607
C	7.38126175759923	5.70509498349054	13.07974278465170
H	6.78198303184829	4.87284028844887	12.64539120790223
C	11.28715856664445	11.80680000545952	14.47682396386128
C	11.37851323319027	13.69266615325885	13.01534975308968
H	11.74684829506463	14.70167893981295	12.77231310771824
C	11.77703972735198	13.08741447206574	14.23497550682496
H	12.44599833642646	13.59738425079944	14.94403264893422
C	10.03493715032184	11.73091770426720	12.35925500865774
C	10.41866775379171	11.12896266483499	13.57641931928342
C	6.39688818868196	6.84457757378579	13.36575224014111
C	5.01286088766139	6.58514226733064	13.39555995118912
H	4.65305499331421	5.56133209507264	13.19777076022808
C	4.09087656887938	7.61044269082074	13.67049131301622
H	3.01125276631520	7.38846993503768	13.68252870913681
C	5.92933936046769	9.17998383535187	13.89540833471592
H	6.30046034415268	10.19993845140759	14.07637540361263
C	4.54956941278210	8.91366725291921	13.92246898958817
H	3.83424755524764	9.72590624950664	14.12731467680806
C	8.41570503421576	6.09579079621778	12.01647910213862
C	6.85052763612964	8.16078814796459	13.61552656088014
H	7.93218320172259	8.37421053600191	13.62224954538152
C	8.95118808297633	7.26963087855818	9.93234159809510
H	8.63998624205488	7.92627028168002	9.10463330994634
C	8.03236743021432	6.91106542915650	10.93009372659301
H	6.99906397990682	7.28702129853803	10.88464978864946
C	10.66711404995180	5.98861545103485	11.06867919909609
H	11.70666815098292	5.63096184663366	11.13963983599075
C	10.27713763625981	6.80878100809718	9.99801249539965
H	11.00154937512161	7.10130174175250	9.22240171620497
C	9.13200537578282	10.94426213104496	11.42156804447796
H	9.21945760463433	9.88536258663564	11.75541610978221
C	9.74526507325205	5.63820451560394	12.06840773225075
H	10.06222016792625	5.01411018331966	12.91654934100879
C	9.65658584081602	11.00411796718274	9.99174332414181
C	10.79715644710988	10.24331224498061	9.65726041058940
H	11.24976885885100	9.60316740065913	10.42982793722653
C	11.34217661668174	10.29314701953066	8.36708368689624
H	12.22842710101012	9.68517842385302	8.12454065429908
C	10.75858927846728	11.11306908087173	7.38316535756559
H	11.18402443146179	11.15138590978198	6.36780448339224
C	9.62851989303632	11.88022688512691	7.70759603788243
H	9.16392978399687	12.52751732731052	6.94640329265248
C	9.08136622397067	11.82444851650800	9.00198002025236
H	8.18734102209350	12.41677680575588	9.24942045440161
C	7.64983657226141	11.29036148633704	11.53184055943735

C	6.70954103030270	10.49183764476183	10.84503257601096
H	7.07183167873172	9.64256791276347	10.24708760562080
C	5.33734963575125	10.75647825094701	10.92945926535639
H	4.62146219113072	10.10533929306440	10.40458402947249
C	4.87356503512672	11.83104126115292	11.70898166713373
H	3.79393919994875	12.03401569035273	11.78700943911400
C	5.79747564875510	12.62813462786500	12.40191379587747
H	5.44573681547234	13.46521539174981	13.02596406735245
C	7.17562814831281	12.35909562864708	12.31396792777397
H	7.90062182901443	12.97564914293362	12.86569393377086
H	11.43427909406357	8.83519243897392	17.06098735919635

[(*E*,*E*)-($^{4\text{-BzhH2}}$Box$_2$CH)]$^-$ (11c) (G = –1838.80202399 E_h)

O	10.47025208610705	6.69816682712634	16.12628223694060
N	9.17231176136695	7.46847098089840	14.40013673888845
C	8.88574292069861	6.13553625316356	14.62600032937317
O	11.48995967419057	11.25843965706247	15.40212659006757
N	10.29780096759868	10.47687390222186	13.60773923842285
C	7.96914900609373	5.25037900066836	14.00250250839196
C	7.93912938252768	3.92297521330183	14.48882664281402
H	7.23394428839015	3.21646509806885	14.02043473528711
C	8.75879389240508	3.47375501409614	15.54155941649942
H	8.69186361634422	2.42617129760126	15.87395071944982
C	9.66337292475938	4.35301540227871	16.18014074644140
H	10.30724119556448	4.03107960088283	17.01164371925038
C	9.69687377975630	5.65773882219262	15.69593605809341
C	10.11431199100799	7.79329823062025	15.29001937269896
C	10.79181922809230	10.17033269672424	14.80946722211100
C	10.79664259431480	8.98880485219544	15.57086071485708
C	11.02881401647341	13.93305419473698	12.33787534249235
H	10.88794023533949	14.61338263144811	11.48154798084921
C	6.98056487644934	5.68958783158988	12.93090479035964
H	6.54289703190901	4.75333815564189	12.51681383074986
C	11.41170498600117	12.26976630373849	14.48731792754685
C	11.74551742449036	14.39020730855679	13.45969099720914
H	12.14901813763354	15.41472470780163	13.46669248653896
C	11.95680259123464	13.54728014371422	14.57431164901004
H	12.52315287445513	13.87255767554284	15.45920875062802
C	10.47772922073961	12.63263832727889	12.24730963396400
C	10.66686251704743	11.78758596356931	13.37052822301669
C	5.82035279643440	6.45670037565554	13.56374871721905
C	4.51387799197900	5.93144191976739	13.53914093232518
H	4.33595105516144	4.95672191034194	13.05531131928679
C	3.44144543990840	6.63425510172035	14.11596799981946
H	2.42544690892194	6.20884020304164	14.08226143074298
C	4.97026874557172	8.40575492456284	14.76371895241456
H	5.15924979645456	9.38227244185029	15.23625074694281
C	3.66709857561168	7.87681508261612	14.72994481906522
H	2.82966900058623	8.43301883051693	15.18058598566881
C	7.58706588219227	6.44003549369106	11.74045205433182
C	6.03975217738732	7.70570139927966	14.18595832623396
H	7.06583578186413	8.11127206229511	14.21050672560123
C	7.27825426367259	7.85328596616408	9.76488212610027
H	6.61145831894376	8.45108544233038	9.12448129851596
C	6.75097740399634	7.17204466326874	10.87303645280533
H	5.67268573391527	7.23060862650507	11.08772278114330
C	9.49088961579236	7.05731633906493	10.34216037466944
H	10.57232756005421	7.02405886799626	10.14647078249570
C	8.65523916019694	7.80662453704069	9.49873175616562
H	9.08519269570883	8.37169016932445	8.65881930014248
C	9.71068119580370	12.23986054738506	10.98976187258897
H	9.67490676502627	13.16632173834482	10.37883528024692
C	8.96379538230506	6.38189512545997	11.45223642291590
H	9.62973669455146	5.81842517676868	12.12058940976983
C	10.39719525098004	11.20847612781699	10.09208587423577
C	11.29685161476020	10.23675314307713	10.57054200675642
H	11.46680423103745	10.14550054017731	11.65196048946648
C	11.94280846734898	9.36373745435244	9.67871511510618
H	12.64903734709157	8.61705268304836	10.07480418439961
C	11.69171762046303	9.43134821923128	8.29915962568083
H	12.19998674664151	8.74346337131389	7.60514239722545
C	10.77396501250709	10.38009772708268	7.81392121408705
H	10.55821219000556	10.44119686163363	6.73509912518665
C	10.13795056810177	11.25708977577649	8.70502935258190
H	9.42320039808966	12.00419998338240	8.32216068507421
C	8.24735459786058	11.89654211185336	11.27804619304178
C	7.84578690309856	10.62447384839658	11.73182530448100
H	8.59314694359535	9.83684487614015	11.90174506437156
C	6.49195652277853	10.37575471044396	12.00653939736178
H	6.19423393705537	9.38241048961881	12.36569244500359
C	5.52408756189693	11.37860379463459	11.83450986272653
H	4.46638559756419	11.16649859715329	12.05830653536109
C	5.91623461373775	12.64820816054337	11.37803545991354
H	5.16901236324523	13.44561853131697	11.23436936564041
C	7.26992368034320	12.89875973442914	11.10350474085896
H	7.58372681615546	13.89560013484497	10.75080908695979
H	11.42511885261002	8.99176608601697	16.47011020531434

[(*Z*,*E*)-($^{4\text{-BzhH2}}$Box$_2$CH)]$^-$ (11d) (G = –1838.81572179 E_h)

O	8.36486386047036	9.34334193778860	12.65922993037629
N	8.98938757580361	7.93864926495868	14.35456958334191
C	8.25648315901819	7.22745267068083	13.42956020767712
O	10.53276530013702	12.53117983210485	14.68166103969221
N	9.17934796441147	12.15465402293175	12.86515044733137
C	7.88937441406411	5.85875498448089	13.37003499670055
C	7.12906824660399	5.45365137518998	12.24971281372629
H	6.82993611803203	4.39443504175037	12.18059539346974
C	6.74775600463935	6.34119430372201	11.22420981921932
H	6.15689014540022	5.96701402508999	10.37394577438631
C	7.11918447800783	7.70537071734830	11.26913386662365
H	6.83848279380088	8.41730090337715	10.47965052666410
C	7.86776922927393	8.10070819034070	12.37328786558672
C	9.04586643083096	9.19442257242621	13.88252274926442
C	9.74028044241402	11.60333435109393	13.94588466661975
C	9.69564262139207	10.29742519219430	14.46179965712932
H	10.23681847364938	10.09813945569365	15.39399928549589
C	9.95913404293419	15.74260756400086	12.16791345889470
H	9.78208702387850	16.56942136439040	11.46196771850841
C	8.26613930745618	4.85997865821665	14.45591622499452
H	7.99293569587283	3.85802891071746	14.05409576216739
C	10.44749638402238	13.70170428651339	13.97764058232345
C	10.78888182548154	15.96040678300915	13.28696363731938
H	11.24638781395054	16.95127342192759	13.43382540090863
C	11.05166167894024	14.93148038392484	14.22583830997035
H	11.70294217979658	15.09196307652110	15.09752647817194
C	9.33614897695773	14.49672239627099	11.93464766182291
C	9.60532919329803	13.46257136481955	12.85754749900932
C	7.42182997271767	5.05537205988969	15.71311833903858
C	6.44473790206430	4.10564450564658	16.06903707935057
H	6.30366093631411	3.21602484863277	15.43300064949988
C	5.65368120445164	4.27741464487002	17.21838770592250
H	4.89539271572611	3.52212515671152	17.48055075354119
C	6.80497973760148	6.36500915920749	17.67787598837415
H	6.94679838709141	7.26076824769315	18.30335203244697
C	5.83246505438631	5.41022508455413	18.02827886269346
H	5.21428385411451	5.55089010669383	18.92932017220419
C	9.76911207431501	4.79113889235492	14.75381723871735
C	7.59684186964267	6.19232614983750	16.53342341082156
H	8.34545073062712	6.94487344996968	16.23242088057951
C	12.09432414755877	5.07144959368177	14.05010977122889
H	12.81909233540490	5.41090649884819	13.29330829782121
C	10.71998030147290	5.19955167407630	13.79808512116244
H	10.37092186322491	5.63551761311895	12.85071873737842
C	11.60710044943435	4.11878031285933	16.22537089208453
H	11.94601494009900	3.69828078457654	17.18582627935369
C	12.54626213556574	4.53106725519041	15.26566978498802
H	13.62532435820006	4.44023894588110	15.46749050630754
C	8.49211071276611	14.14342250695125	10.71681034849246
H	7.84933022105026	13.29929275644345	11.05339421740440
C	10.23264132172066	4.24957534813088	15.97025924987851
H	9.50222682989106	3.93643429241475	16.73176357450061
C	9.42353972535129	13.56304670336563	9.65548990646907
C	9.60614106809868	12.16651140542628	9.58143653368142
H	9.06463368946181	11.52248936886264	10.29200246925542
C	10.51248784192888	11.61445604099864	8.66116777766339
H	10.64695087201615	10.52198881861542	8.62190061077387

C	11.24995416556277	12.44768147734363	7.80362257359452
H	11.95923477886695	12.01358823894598	7.08106605726019
C	11.08545514814073	13.84229303013959	7.88316023390898
H	11.66845743460956	14.50576616043806	7.22424684072050
C	10.18460760450421	14.39400089010352	8.80650902925126
H	10.06678693758724	15.48691571040714	8.87318711192399
C	7.54753828195495	15.23814025830522	10.23161472820352
C	7.15484047671529	15.32933379487236	8.87892145843278
H	7.58909311099081	14.63314480296063	8.14636894486088
C	6.22080289582302	16.28970535653720	8.45698034284717
H	5.93459015507932	16.33921160410602	7.39412614582486
C	5.65271708930942	17.18111049469794	9.38153814341357
H	4.92357300082412	17.93782037339502	9.05134415415539
C	6.02309459248233	17.09210206997876	10.73418908062869
H	5.58021682880342	17.77771559131090	11.47423691010861
C	6.95718230732390	16.13180729375356	11.15232615714899
H	7.24304565858454	16.06711357571603	12.21255353868441

[(*Z*,*E*)-($^{4\text{-BzhH2}}$Box$_2$CH)]$^-$ (11e) (G = –1838.81481865 E_h)

O	8.82354722867908	9.86547531815580	12.90089525766751
N	9.56149314838731	8.70269786819011	14.73420648371687
C	8.96587646611527	7.83090400682956	13.85047658218387
O	10.47287910152626	13.47317776306895	14.69877161082335
N	9.25511088859509	12.77119801981613	12.88247895697380
C	8.76945923170778	6.43426542055184	13.93077775438415
C	8.07388231089893	5.81988847694870	12.86559331572383
H	7.88828452652043	4.73470799495131	12.90561947676501
C	7.62110744853299	6.55264626120164	11.75019427910762
H	7.09160237757993	6.02915562766908	10.93902722237259
C	7.82873388187974	7.95112950303713	11.65212910124875
H	7.47533386427057	8.53512554461495	10.79010536952473
C	8.49902219675599	8.54875904443233	12.71483631438109
C	9.47246614444183	9.91015537503005	14.15298361405484
C	9.84030874984474	12.38966034082306	14.02206624180756
C	9.94870813165999	11.13674498544889	14.64769325243575
H	10.47180551577912	11.09198879531011	15.61003111532573
C	9.57671537835472	16.36469998741582	11.90653012529875
H	9.31909987496636	17.10044751060141	11.12829567658105
C	9.22339148407380	5.71796646241968	15.19515317440304
H	9.94186121062092	6.42085400222370	15.67333443193647
C	10.26063507689860	14.56068726827975	13.89561686365244
C	10.32506290973334	16.78237831579784	13.02612008778832
H	10.64025920796935	17.83483310494447	13.10158491062509
C	10.68514813923741	15.87712432954246	14.05583819760781
H	11.27404918238543	16.19407034941964	14.92907919751413
C	9.13502758819213	15.03091986436929	11.76200429901714
C	9.50304359874151	14.12002240790188	12.77618859030256
C	8.02512105596530	5.62496712979448	16.13626934870119
C	7.11754646752845	4.54627646597270	16.08898904234061
H	7.30949609318741	3.70844361066252	15.40094953814938
C	5.98031877884857	4.52985617305113	16.91145632888042
H	5.28351320656048	3.67766248839785	16.86291158818105
C	6.61832746697975	6.68302041451281	17.83351843565366
H	6.42327621240633	7.53115197108531	18.50881727234845
C	5.72703446220041	5.59714153285382	17.79074300349855
H	4.83403600844760	5.58495033475729	18.43550546567688
C	9.97801156089863	4.40935658859191	14.98085890895641
C	7.75714796791240	6.69701093211614	17.01295716165492
H	8.44111360596215	7.56082595134746	17.01049849069910
C	11.41541605886574	2.97549684411231	13.60807076858378
H	11.93217338390034	2.79352612859208	12.65224685381218
C	10.65491675977941	4.14485512001552	13.77157652702637
H	10.57808500828153	4.87208644555234	12.95026219449608
C	10.86024649442702	2.29904627493369	15.86743268106961
H	10.93767772544836	1.58171943186429	16.70019749880468
C	11.51861865422780	2.04253076959191	14.65277234961092
H	12.11054235201080	1.12271357666394	14.52306928250631
C	8.39414224349518	14.47045025695178	10.55484804414046
H	7.85974767106489	13.57406993599078	10.94208751214152
C	10.10210896232490	3.46926750351798	16.02793218785348
H	9.59270104680694	3.66190598881779	16.98421811485370
C	9.43590786793944	13.94425769733163	9.57066009414975
C	9.81110942774200	12.58555792914906	9.61827822855040
H	9.33597940354788	11.93010880934198	10.36499125415995
C	10.81905127531950	12.09485085385980	8.77189333194129
H	11.10348135992948	11.03225138701664	8.82741129071510
C	11.46733419022502	12.95276590602515	7.86787294300704
H	12.25644756209006	12.56649238411208	7.20326614750415
C	11.10997805232682	14.31250252986914	7.82624819223795
H	11.62094837075697	14.99693170456082	7.13001945563829
C	10.10736659406788	14.80387769112162	8.67585445175866
H	9.83802232586432	15.87132903279048	8.64761492312861
C	7.32889138942745	15.38255434466597	9.95540702925620
C	6.97713718735150	15.30955637630732	8.59035696476231
H	7.52773161204674	14.62285763104229	7.93066262282235
C	5.93821536068345	16.09528857013985	8.06502611546507
H	5.68693933731715	16.01908504782880	6.99494057539789
C	5.22174333777332	16.97184417275695	8.89600206801546
H	4.40977088477581	17.59206800857423	8.48452061709244
C	5.55109624998491	17.04392511843871	10.26020103226777
H	4.99308418467542	17.71962204242802	10.92799185908601
C	6.59022540271694	16.25792840538320	10.78175759214119
H	6.84327451155831	16.31882853851191	11.85050310803758

[(*Z*,*E*)-($^{4\text{-BzhH2}}$Box$_2$CH)]$^-$ (11f) (G = –1838.81659250 E_h)

O	8.72864849962856	10.07700694408930	12.38968061042666
N	9.04150113213675	8.60328735295930	14.11085229091953
C	8.40659340970501	7.95455240719293	13.07273408445551
O	10.80306800007309	13.10939832438014	14.67127645181432
N	9.60804590999321	12.79018806329531	12.74221097385592
C	7.98208760912651	6.61010965152392	12.92016667555714
C	7.36456696452590	6.27512678568983	11.69368195478552
H	7.02453320181752	5.23584164150201	11.55058442045225
C	7.17401267766475	7.20716354769702	10.65422280455076
H	6.68735325461522	6.88649927118456	9.72018070906619
C	7.60363217801535	8.54758299940204	10.79244808751340
H	7.47003906855282	9.29617806300322	9.99834871892558
C	8.21209294507659	8.87200372559622	12.00083419191049
C	9.22855284817658	9.86330862349407	13.68814841078232
C	10.03180645815117	12.22023582825983	13.87289460406801
C	9.85798229144247	10.91889864254402	14.37150038712646
H	10.27430778475392	10.68007069071266	15.35680505393402
C	10.64518229476332	16.32840287224428	12.12247526426997
H	10.57030487865204	17.16331611347892	11.40587061582885
C	8.15475356894203	5.56550664150483	14.01454200340009
H	7.88918010816206	4.59025773991998	13.54725353502390
C	10.85594533052201	14.27235139179491	13.95000142432610
C	11.37137328526250	16.51219297499465	13.31647081514977
H	11.85386414356983	17.48213987188590	13.51348200995088
C	11.48983878939869	15.47033182807234	14.26787887763139
H	12.05130694842720	15.59639541872605	15.20523419111983
C	9.99864983724030	15.11325048788328	11.80333268429024
C	10.11988019040986	14.06533338535698	12.74805199932197
C	7.15703837033071	5.77523700196806	15.15118331361208
C	6.09524939127980	4.87008231690742	15.34247436750784
H	5.99894522998862	4.00448923044220	14.66620698424810
C	5.16395439819512	5.05596755759288	16.37902824549945
H	4.34081824812474	4.33574864861187	16.51292048212934
C	6.34238576596917	7.06862844694908	17.05362662978143
H	6.44091600103320	7.94071390539115	17.71956706527437
C	5.28562067439067	6.15828005551997	17.23994743018863
H	4.55771237948419	6.30998134814791	18.05293838308622
C	9.59730907331437	5.40772980377525	14.51125020349127
C	7.27372627702106	6.88171548786411	16.02199929455250
H	8.09231908350226	7.60084461274750	15.84831780300906
C	12.00815914376029	5.57706909407378	14.13745912929952
H	12.84589252037724	5.89388164495276	13.49628943660394
C	10.68950990863598	5.78569340844782	13.70579834006294
H	10.49595454169649	6.26196339936397	12.73362704292529
C	11.18024145809257	4.60241000314280	16.19691704578170
H	11.36283798863089	4.14198601963665	17.18121715320449
C	12.26155711733485	4.98489745612403	15.38608129168727
H	13.29685716862638	4.83065734487205	15.72921149252162
C	9.22456665943488	14.93767106086774	10.50470212668867
H	9.24551376692568	15.93041445972896	10.00082195623324
C	9.86200132753692	4.81378092704104	15.76224019804523

H	9.01933024893673	4.52340835478228	16.40801415163854
C	9.92618004992974	13.96483549042259	9.55425136733505
C	9.84805784776938	12.56698798756668	9.74779940724902
H	9.30037549079757	12.17859855566793	10.62083780439472
C	10.51041558552312	11.69551216884095	8.87043241895180
H	10.44625333342875	10.60967870876366	9.04415010896145
C	11.25393160227773	12.19673332042388	7.78645394414152
H	11.77002357770339	11.50685166531433	7.09960077240350
C	11.33546313792675	13.58460076991813	7.58739696917778
H	11.91368049950447	13.99117967348613	6.74194461339394
C	10.67503098781931	14.45855344504277	8.46828437384737
H	10.73943584090611	15.54827778477863	8.31126759763832
C	7.74166251916132	14.59577115178002	10.69832163511556
C	6.97834103602789	14.13651241728667	9.60442549997985
H	7.47390560012878	13.97654296858568	8.63445566351285
C	5.60871570868611	13.86449007779170	9.74211575374747
H	5.03475702381127	13.49853100943486	8.87572874081838
C	4.97424813167668	14.04495276363951	10.98286128897266
H	3.90262662636759	13.81807802067290	11.09820176279494
C	5.72460012979304	14.50414650200387	12.07727286070084
H	5.24355085596335	14.63777107301106	13.05911403182517
C	7.09430137966638	14.77747014214026	11.93500086280730
H	7.67961268370259	15.12486142608735	12.79838910469979

[(*Z*,*Z*)-($^{4\text{-BzhH2}}$Box$_2$CH)]$^-$ (11g) (G = –1838.81589933 E_h)

O	10.13705398596025	10.15808594042297	14.60648106378664
N	9.10549747355198	8.29026946462931	13.76498004021540
C	9.87504977240829	7.93692998059060	14.84969751147555
O	9.68517260155366	12.57341255827826	13.35440587876580
N	8.29116615965852	12.60881175938165	11.53880904348672
C	10.08653449235225	6.67706976417591	15.45356399528761
C	10.92029096712789	6.64072883551026	16.59332649964254
H	11.09803695964359	5.67516052318277	17.09303467446444
C	11.53939607195492	7.80004297502779	17.10129425211042
H	12.18380048618986	7.72223207903543	17.99072546473619
C	11.34412728756455	9.06632273697087	16.49507938208838
H	11.81721669402865	9.97947079470493	16.88392334873640
C	10.51259323810143	9.09273067143487	15.37976049711761
C	9.27327659618051	9.61605715257409	13.62889722464824
C	8.85595688541589	11.83834913581779	12.48316674884746
C	8.69851565680083	10.45379703005752	12.65811736276824
H	8.04605093215356	9.95643267836436	11.93076884258334
C	9.16576889669516	16.25453513162703	11.62034216095287
H	9.00128365787477	17.22030025053958	11.11409911775078
C	9.32235058447497	5.48236910912391	14.90051659029368
H	9.03756357630202	5.78388454843381	13.86762962179780
C	9.61700783488571	13.86521755891467	12.90773750393109
C	10.00980590435038	16.20910329565333	12.74726833997637
H	10.48734845115924	17.13387944301831	13.10545615382539
C	10.25386471670457	14.98997773436650	13.42250124559911
H	10.90768088662669	14.92663239575207	14.30440288518433
C	8.51746970449171	15.11059870316124	11.10180309504376
C	8.74858553131493	13.88537708859669	11.77836801433987
C	8.01834838133275	5.35583006934095	15.68314645380337
C	6.86528642042389	6.01828358886687	15.21388209314010
H	6.93468451264638	6.59538560083011	14.27839979575256
C	5.67047544306274	5.98505808976038	15.95120928130786
H	4.78087333324069	6.51153495825729	15.57118458926586
C	6.75843966683756	4.63910918835157	17.65401066827862
H	6.72608490034910	4.10337653012500	18.61591683196655
C	5.60953569540341	5.29052668875168	17.17114651889962
H	4.67216498844404	5.26422207138373	17.74918698004019
C	10.12725734573816	4.19398331035976	14.76348650216351
C	7.95268152420070	4.67596001073419	16.91784132939241
H	8.85096752872596	4.16960395459463	17.30338121790414
C	12.25506532148144	3.06215284328296	14.31477923499569
H	13.34017701904709	3.12614096990541	14.13501147564409
C	11.51972747620636	4.23744625542630	14.53490675670365
H	12.02602918822923	5.21404688788103	14.53341153039668
C	10.22796730712751	1.75406893771274	14.53740386125813
H	9.70839645766771	0.78222626133725	14.53687129668939
C	11.61429614244000	1.81175382075830	14.31924849128645
H	12.19069654422741	0.88870064588237	14.14963316247668
C	7.59460525848526	15.21890241996718	9.89510197159438
H	7.75014926390174	16.24223918896463	9.48342856564065
C	9.49469066234142	2.93200664005531	14.75421539660883
H	8.40845806242579	2.87445917009104	14.92029381587948
C	7.93638828311340	14.25505253699401	8.75293416553634
C	6.96801509637565	13.91673371499619	7.78569543624209
H	5.94316921005078	14.30272708378656	7.89301499726180
C	7.29103848948625	13.08596224468018	6.70036158936439
H	6.51565513139487	12.82752755361144	5.96125240076517
C	8.59296585759872	12.57684287582683	6.56309273696807
H	8.84663160550184	11.91599071531852	5.71898247204754
C	9.56581237637389	12.91019806906380	7.52062897903891
H	10.58706273852133	12.50726569708499	7.43340274152824
C	9.23930717107147	13.74041251409122	8.60383175280953
H	9.99933446570639	13.98730134282533	9.35967970785649
C	6.12751742702720	15.15072249521761	10.31306127097394
C	5.31312798970582	16.29774739599871	10.24424593998759
H	5.74456093490098	17.24098114926409	9.87040264351733
C	3.96588632167825	16.25375269686630	10.64362105162114
H	3.34538757938109	17.16229092884060	10.58053709133234
C	3.41599293070879	15.05330301235685	11.12129426983929
H	2.36257704141178	15.01397511677942	11.44066086886957
C	4.22332704167915	13.90274927040431	11.19299714039362
H	3.80273371716173	12.95832003256929	11.57337594674840
C	5.56609686405032	13.94510514033403	10.79077761777820
H	6.21672727758663	13.05698097112182	10.86492479897426

[(*Z*,*Z*)-($^{4\text{-BzhH2}}$Box$_2$CH)]$^-$ (11h) (G = –1838.81756699 E_h)

O	10.18048224816381	10.01802607661765	14.54052208962744
N	8.95835090687060	8.20665111346977	13.83860470517188
C	9.77121492672803	7.83217360103532	14.88086903965588
O	9.81973934843191	12.41946118243820	13.23525394792630
N	8.30111727450901	12.50341090865042	11.51650490738082
C	9.93924971886917	6.57900163862685	15.51419971754770
C	10.88216978711896	6.50599205507041	16.56380147858352
H	11.04707711220295	5.53679653947173	17.06053359922637
C	11.62038819937519	7.63082361708392	16.98403090599472
H	12.34526533682240	7.52579609980629	17.80623576160753
C	11.45517296024180	8.89330825350572	16.36143602419590
H	12.02688810104524	9.77886754995983	16.67516625841447
C	10.53307001364564	8.95174242864982	15.32093436544885
C	9.21202792495851	9.51065060841582	13.64267500985478
C	8.87616072763054	11.72199329110917	12.44486820341398
C	8.63477403380312	10.35851543138239	12.68258108778970
H	7.89177387775078	9.89027842051681	12.02650547844646
C	9.37259531168572	16.09020650060361	11.43355460583671
H	9.20152408973811	17.05511798891591	10.93057273182276
C	9.07878852978032	5.41122177170135	15.05974637387137
H	8.78350716309755	5.65669438248339	14.01577697314503
C	9.80743225947011	13.69964953431890	12.75434959354009
C	10.30448897111589	16.01813059193503	12.48858099202202
H	10.85211877843783	16.92478958970497	12.78953084619622
C	10.54314232857473	14.80248590194735	13.17714821503819
H	11.26375027819944	14.73151350242734	14.00490779261476
C	8.63963393691596	14.96136033171671	11.00385681148400
C	8.86429985861638	13.74460993066803	11.68848000834714
C	7.77424555551363	5.26490943664149	15.84379286915776
C	6.77179895133695	4.40009946815257	15.35355568822101
H	6.94847372685580	3.85799300063008	14.41057184833976
C	5.56818247471584	4.22078155979431	16.04952113392029
H	4.79749303570073	3.54414601709138	15.64691571997444
C	6.32945039346505	5.77623107297466	17.74865088497010
H	6.16012079294108	6.32827634286812	18.68681250398718
C	5.34216604249708	4.90902412637683	17.25549756893690
H	4.39600036483359	4.77373692428677	17.80298204846579
C	9.88166309849058	4.11530742200971	15.02373988641460
C	7.53508738296083	5.95357147329504	17.04713897182634
H	8.30587304639467	6.64079676587042	17.42628222632931
C	11.57071521246567	2.73246184670281	13.91591211683905
H	12.25717865429157	2.57052110507344	13.06966149521760

C	10.77302387293678	3.88500607474147	13.95444543086730
H	10.84175222727313	4.63428941455644	13.14975524530426
C	10.61589129696126	2.00629974095796	16.02530968760713
H	10.54965899036053	1.27292420023066	16.84492046574997
C	11.49641104200471	1.78599164688398	14.95420683177428
H	12.12256943523777	0.88027801274817	14.92607257023158
C	7.66478985613610	14.97286723636870	9.83760007879682
H	6.98852731023748	14.10787433552229	10.01591350065095
C	9.81344109726808	3.15998512601865	16.05756153787351
H	9.11677808695025	3.32447332477975	16.89355467531733
C	8.32293887518607	14.72117359162296	8.48062877761421
C	7.50643968243723	14.44734368208903	7.36185311500593
H	6.41307551430465	14.41195411427010	7.49453880922936
C	8.06564125579517	14.22800075158686	6.09522289804983
H	7.41021080361403	14.01196366812241	5.23636849882198
C	9.46103981178885	14.27648552169364	5.92289309754841
H	9.90423207840601	14.10070205661566	4.92994073400309
C	10.28250018649049	14.54143819288432	7.02965768832280
H	11.37710301692476	14.57225148921557	6.90936402544382
C	9.71748944867893	14.76043318958825	8.29851472627143
H	10.35999551233535	14.95783319038526	9.16933575784720
C	6.81344759608412	16.23803947719884	9.84360409408572
C	7.09812493966411	17.33958707487585	9.01196817004108
H	7.92778021266050	17.26710306472041	8.29244790234383
C	6.33285084743128	18.51617591673705	9.08914789966999
H	6.57356248485597	19.36729964476622	8.43215491203659
C	5.26521881067311	18.60818522150602	9.99622798112157
H	4.66250270513922	19.52824880816313	10.05452056933517
C	4.97123451052703	17.51339480597996	10.82954321516282
H	4.13554735989959	17.57308729559270	11.54484704725317
C	5.74050900885151	16.34351210707352	10.75391120877390
H	5.52097238662221	15.49142261850258	11.41679336103859

[(*Z*,*Z*)-($^{4\text{-BzhH2}}$Box$_2$CH)]$^{-}$ (11i) (G = –1838.81127688 E_h)

O	10.54338894235867	10.51092934029994	14.99406370137314
N	9.21067263529927	8.74690030510914	14.40106304243397
C	9.94927844219659	8.39836847692078	15.50989557730115
O	10.42674509098285	12.80523591592794	13.46928017597407
N	8.98345422048119	12.85378907209763	11.69322415932299
C	9.97960533484150	7.21122929602099	16.28574353443134
C	10.86515895383296	7.19494913755127	17.38703421174516
H	10.90813329139240	6.28260439091648	18.00505906215957
C	11.68273427070207	8.29087694571095	17.72621677894462
H	12.35167742255868	8.22038042350117	18.59780945693060
C	11.65101432803592	9.48289597911528	16.96495239799467
H	12.27476208356986	10.35439625558650	17.21094413059284
C	10.77822452698360	9.49587675112905	15.88148802831471
C	9.57469304623845	10.00504212526867	14.10393231099462
C	9.46077994932287	12.11578379431655	12.70866833792280
C	9.09965019652281	10.80203849118602	13.04955508900910
H	8.34499009030724	10.33562698603268	12.40574768578389
C	10.40414915383550	16.30510221325271	11.33000359230597
H	10.36769832213291	17.22113589700372	10.71703980520211
C	9.12727661531396	5.99328700363528	15.95593250872120
H	9.24016190396564	5.30143010440878	16.82115263878863
C	10.53801725433106	14.03383224387862	12.87674050818982
C	11.27764831651738	16.25426115805469	12.43405794030831
H	11.90667249849411	17.12656708873922	12.66973366229636
C	11.36142620480660	15.09478600674522	13.24032718472905
H	12.03828343592271	15.02718648640661	14.10426073536039
C	9.56889243728518	15.22566931405443	10.96331716244929
C	9.63788873401091	14.06286006555673	11.77277454879063
C	7.62529608324377	6.28565464349307	15.85174159010695
C	6.77018087003272	5.39835502550777	15.16667612977741
H	7.20167190338444	4.52681238058894	14.65128824384127
C	5.38456633729396	5.62045459344436	15.12250784213570
H	4.73662632201442	4.91707705603112	14.57523849177678
C	5.67112213001852	7.62900872781485	16.44920651717260
H	5.25010565279677	8.51781142721626	16.94522646031964
C	4.82745135140769	6.73941685597975	15.76323640834556
H	3.74217860751897	6.92272474045890	15.72012402220607
C	9.67060843226816	5.24330044496490	14.74179730467135
C	7.05529961595318	7.40326741360160	16.49229511371210
H	7.71337570970474	8.10970095507248	17.01893503239601
C	10.09607260544127	5.10579234927695	12.34256341320505
H	10.02562450531947	5.55634993835895	11.33974790728762
C	9.58044614110374	5.80021470119272	13.44647900260256
H	9.12433574316138	6.79863617653297	13.33590644141475
C	10.79537159259832	3.28683233185170	13.79084424098761
H	11.26850330284521	2.30198687233979	13.93380453889869
C	10.70321906667368	3.84700980892853	12.50673077599448
H	11.10498309192961	3.30559882702979	11.63538212179688
C	8.63315708818071	15.33824231917922	9.76736004211238
H	8.92186808242244	16.27784525563966	9.24410019489714
C	10.28185901752988	3.98420349522248	14.89819020152952
H	10.35459129457656	3.54406166210248	15.90660284690064
C	8.79830014535077	14.21908211557963	8.73174586219323
C	7.76882128509447	13.94081477562398	7.80952121737549
H	6.81875864246696	14.49166021271551	7.88256716729231
C	7.93544651238480	12.96476091352566	6.81412779650336
H	7.11435531171313	12.75957807907603	6.10859523725232
C	9.13847422039102	12.24531766425319	6.72425968535795
H	9.26765320696958	11.47059615818893	5.95199047150775
C	10.17068035916012	12.51592654332541	7.63807379406728
H	11.11468309109126	11.95057952849972	7.58804175689116
C	10.00108737184183	13.49283991195569	8.63084811031994
H	10.80665418595956	13.69173563715750	9.35264420736859
C	7.18372658871187	15.52878875558389	10.20851925848322
C	6.54188436599672	16.76908455669372	10.02635745812890
H	7.09639339501233	17.59643984722629	9.55316584271189
C	5.21134128621933	16.96225287054173	10.43689963134833
H	4.72627547347095	17.93976337175257	10.28394797929237
C	4.50491509874620	15.90919457258198	11.03964065273355
H	3.46232365442591	16.05507465315153	11.36446276617393
C	5.14006541341177	14.66765623833354	11.22741622355828
H	4.59589734810548	13.83789816955930	11.70594035678420
C	6.46624728212438	14.47345590826408	10.81480410708215
H	6.98475351168835	13.51355024615275	10.97851756511270

Xray Crystallographic Analysis

	**6**	**7**	**8m**
Empirical formula	$C_{41} H_{30} N_2 O_2$	$C_{48} H_{37} Na N_2 O_2$	$C_{51.50} H_{41} K N_2 O_2$
CCDC number	2031907	2031908	2031909
Formula weight	582.67	696.78	758.96
Temperature [K]	100(2)	100(2)	100(2)
Wavelength [Å]	0.71073	0.56086	0.71073
Crystal system	Monoclinic	Monoclinic	Triclinic
Space group	$P2_1/c$	$P2_1/n$	$P\bar{1}$
a [Å]	13.281(3)	15.287(2)	8.225(2)
b [Å]	30.091(6)	9.789(2)	9.434(2)
c [Å]	7.754(2)	23.990(3)	27.089(4)
α [°]	90	90	82.78(3)
β [°]	105.81(2)	90.16(3)	82.16(2)
γ [°]	90	90	69.27(2)
V [Å^3]	2981.6(12)	3590.0(10)	1940.7(7)
Z	4	4	2
ρ [Mgm^{-3}]	1.298	1.289	1.299
μ [mm^{-1}]	0.080	0.056	0.183
F (000)	1224	1464	798
Crystal size [mm]	0.179 × 0.142 × 0.085	0.291 × 0.285 × 0.213	0.220 × 0.113 × 0.073
θ-area [°]	1.353 to 25.349	1.340 to 20.068	1.523 to 26.028
Index ranges	$-15 \le h \le 15$, $-36 \le k \le 36$, $-9 \le l \le 9$	$-18 \le h \le 18$, $-11 \le k \le 11$, $-29 \le l \le 29$	$-10 \le h \le 10$, $-11 \le k \le 11$, $-33 \le l \le 33$
Total number reflect.	57812	89033	39440
Unique reflections	5458	6854	7636
R_{int}	0.0786	0.0682	0.0647
Max. and min. transmission	0.7452 and 0.6990	0.7444 and 0.6936	0.7453 and 0.6869
Data / restraints / parameters	5458 / 0 / 407	6854 / 0 / 479	7636 / 84 / 543
Goodness-of-fit on F^2	1.028	1.055	1.028
*R*1 [I>2σ(I)]	0.0403	0.0415	0.0436
*wR*2 [I>2σ(I)]	0.0836	0.0923	0.0881
*R*1 [all data]	0.0640	0.0574	0.0712
*wR*2 [all data]	0.0955	0.1002	0.0980
Extinction coefficient	0.0026(4)	-	
Largest diff. peak and hole max. / min. [e·Å^{-3}]	0.288 and -0.209	0.241 and -0.304	0.248 and -0.328

	**8d**	**9**	**10**
Empirical formula	C_{103} H_{82} K_2 N_4 O_4	C_{103} H_{82} Rb_2 N_4 O_4	$C_{97.75}$ H_{76} Cs_2 N_4 O_4
CCDC number	2031910	2031911	2031912
Formula weight	1517.92	1610.66	1636.44
Temperature [K]	100(2)	100(2)	100(2)
Wavelength [Å]	0.56087	0.71073	0.71073
Crystal system	Monoclinic	Monoclinic	Monoclinic
Space group	$P2_1/c$	$P2_1/c$	$P2_1/c$
a [Å]	13.806(2)	13.848(2)	27.438(4)
b [Å]	21.863(4	21.699(3)	21.780(3)
c [Å]	26.528(4)	26.849(3)	26.586(3)
α [°]	90	90	90
β [°]	101.79(3)	102.49(3)	103.84(2)
γ [°]	90	90	90
V [Å^3]	7838(2)	7877(2)	15427(4)
Z	4	4	8
ρ [Mgm^{-3}]	1.286	1.358	1.409
μ [mm^{-1}]	0.102	1.302	1.003
F (000)	3192	3336	6660
Crystal size [mm^3]	0.219 x 0.114 x 0.076	0.256 × 0.131 × 0.085	0.215 × 0.117 × 0.085
θ-area [°]	1.286 to 19.932	1.218 to 25.594	0.764 to 25.381
Index ranges	$-16 \le h \le 16$,<br>$-26 \le k \le 26$,<br>$-32 \le l \le 31$	$-16 \le h \le 16$,<br>$-26 \le k \le 24$,<br>$-32 \le l \le 32$	$-33 \le h \le 33$,<br>$-26 \le k \le 26$,<br>$-32 \le l \le 32$
Total number reflect.	207083	97550	341920
Unique reflections	14566	14651	28314
R_{int}	0.1415	0.0850	0.1293
Max. and min. transmission	0.7444 and 0.6071	0.7452 and 0.5487	0.7455 and 0.6929
Data / restraints / parameters	14566 / 455 / 1035	14651 / 1747 / 1149	28314 / 5263 / 2224
Goodness-of-fit on F^2	1.044	1.133	1.012
$R1$ [I>2σ(I)]	0.0570	0.0787	0.0407
$wR2$ [I>2σ(I)]	0.1361	0.1824	0.0781
$R1$ [all data]	0.0982	0.1065	0.0734
$wR2$ [all data]	0.1596	0.1958	0.0914
Largest diff. peak and hole max. / min. [e·Å^{-3}]	0.530 and -0.518	2.874 and -0.942	0.699 and -0.618

	**11**	**12**	
Empirical formula	$C_{61}H_{69}KN_2O_{10}$	$C_{81}H_{107}K_3N_2O_{18}$	
CCDC number	2031913	2031914	
Formula weight	1029.28	1513.98	
Temperature [K]	100(2)	100(2)	
Wavelength [Å]	0.71073	0.56086	
Crystal system	Triclinic	Monoclinic	
Space group	$P\bar{1}$	$P2/n$	
a [Å]	10.519(2)	18.494(3)	
b [Å]	14.871(2)	10.533(2)	
c [Å]	19.043(3)	19.977(3)	
α [°]	72.70(2)	90	
β [°]	77.38(3)	94.83(2)	
γ [°]	82.15(2)	90	
V [$Å^3$]	2767.2(9)	3877.6(11)	
Z	2	2	
ρ [Mgm^{-3}]	1.235	1.297	
μ [mm^{-1}]	0.156	0.135	
F (000)	1096	1616	
Crystal size [mm^3]	0.253 × 0.141 × 0.075	0.300 × 0.268 × 0.172	
θ-area [°]	1.140 to 26.392	1.615 to 19.826	
Index ranges	$-13 \le h \le 13$,<br>$-18 \le k \le 18$,<br>$-23 \le l \le 23$	$-22 \le h \le 22$,<br>$-12 \le k \le 12$,<br>$-24 \le l \le 24$	
Total number reflect.	57066	169763	
Unique reflections	11321	7151	
R_{int}	0.0418	0.0885	
Max. and min. transmission	0.7454 and 0.6942	0.7444 and 0.6571	
Data / restraints / parameters	11321 / 1038 / 779	7151 / 2461 / 681	
Goodness-of-fit on F^2	1.021	1.116	
*R*1 [I>2σ(I)]	0.0415	0.0627	
*wR*2 [I>2σ(I)]	0.0974	0.1725	
*R*1 [all data]	0.0609	0.0773	
*wR*2 [all data]	0.1081	0.1854	
Largest diff. peak and hole max. / min. [e·$Å^{-3}$]	0.474 and -0.360	0.534 and -0.379	

XRAY: Compound 6

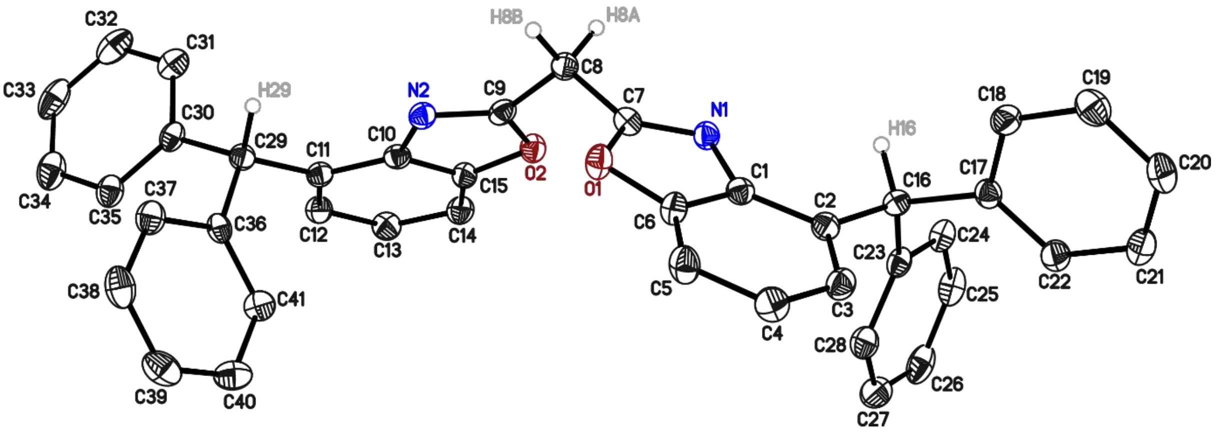

Figure S99. Asymmetric unit of 6. Displacement parameters are depicted at 50% probability. Hydrogen atoms except the hydrogen atoms bound to the methylene backbone (H8) and benzylic groups (H16, H29) are omitted for clarity.

Table S2. Bond lengths [Å] and angles [°] for **6**.

O(1)-C(7)	1.385(2)	C(10)-C(15)	1.385(2)
O(1)-C(6)	1.3863(19)	C(10)-C(11)	1.401(2)
C(1)-C(6)	1.386(2)	C(11)-C(12)	1.390(2)
C(1)-C(2)	1.400(2)	C(11)-C(29)	1.522(2)
C(1)-N(1)	1.412(2)	C(12)-C(13)	1.398(2)
N(1)-C(7)	1.289(2)	C(13)-C(14)	1.388(2)
N(2)-C(9)	1.291(2)	C(14)-C(15)	1.382(2)
N(2)-C(10)	1.413(2)	C(16)-C(23)	1.524(2)
O(2)-C(9)	1.3877(19)	C(16)-C(17)	1.536(2)
O(2)-C(15)	1.3946(19)	C(17)-C(22)	1.391(2)
C(2)-C(3)	1.395(2)	C(17)-C(18)	1.396(2)
C(2)-C(16)	1.523(2)	C(18)-C(19)	1.390(2)
C(6)-C(5)	1.383(2)	C(19)-C(20)	1.385(3)
C(5)-C(4)	1.387(2)	C(20)-C(21)	1.383(2)
C(4)-C(3)	1.402(2)	C(21)-C(22)	1.391(2)
C(7)-C(8)	1.491(2)	C(23)-C(28)	1.395(2)
C(8)-C(9)	1.497(2)	C(23)-C(24)	1.401(2)

C(24)-C(25)	1.382(2)	N(1)-C(7)-O(1)	115.58(14)
C(25)-C(26)	1.395(3)	N(1)-C(7)-C(8)	128.21(15)
C(26)-C(27)	1.383(3)	O(1)-C(7)-C(8)	116.15(14)
C(27)-C(28)	1.394(2)	C(7)-C(8)-C(9)	115.71(14)
C(29)-C(30)	1.531(2)	N(2)-C(9)-O(2)	115.54(14)
C(29)-C(36)	1.533(2)	N(2)-C(9)-C(8)	127.65(15)
C(30)-C(31)	1.392(2)	O(2)-C(9)-C(8)	116.60(14)
C(30)-C(35)	1.397(2)	C(15)-C(10)-C(11)	120.98(15)
C(31)-C(32)	1.390(3)	C(15)-C(10)-N(2)	109.02(14)
C(32)-C(33)	1.388(3)	C(11)-C(10)-N(2)	129.97(15)
C(33)-C(34)	1.382(3)	C(12)-C(11)-C(10)	115.48(15)
C(34)-C(35)	1.399(2)	C(12)-C(11)-C(29)	123.47(15)
C(36)-C(41)	1.389(2)	C(10)-C(11)-C(29)	121.02(14)
C(36)-C(37)	1.400(2)	C(11)-C(12)-C(13)	122.60(16)
C(37)-C(38)	1.385(2)	C(14)-C(13)-C(12)	121.86(16)
C(38)-C(39)	1.390(3)	C(15)-C(14)-C(13)	115.11(16)
C(39)-C(40)	1.381(3)	C(14)-C(15)-C(10)	123.97(15)
C(40)-C(41)	1.393(2)	C(14)-C(15)-O(2)	128.43(15)
		C(10)-C(15)-O(2)	107.56(14)
C(7)-O(1)-C(6)	103.51(12)	C(2)-C(16)-C(23)	113.95(13)
C(6)-C(1)-C(2)	120.98(15)	C(2)-C(16)-C(17)	110.79(13)
C(6)-C(1)-N(1)	108.62(14)	C(23)-C(16)-C(17)	111.89(13)
C(2)-C(1)-N(1)	130.36(15)	C(22)-C(17)-C(18)	118.24(15)
C(7)-N(1)-C(1)	104.40(14)	C(22)-C(17)-C(16)	122.85(15)
C(9)-N(2)-C(10)	104.36(14)	C(18)-C(17)-C(16)	118.87(15)
C(9)-O(2)-C(15)	103.51(12)	C(19)-C(18)-C(17)	121.06(16)
C(3)-C(2)-C(1)	115.62(15)	C(20)-C(19)-C(18)	119.97(17)
C(3)-C(2)-C(16)	122.59(14)	C(21)-C(20)-C(19)	119.54(16)
C(1)-C(2)-C(16)	121.76(15)	C(20)-C(21)-C(22)	120.49(17)
C(5)-C(6)-C(1)	123.86(16)	C(21)-C(22)-C(17)	120.69(16)
C(5)-C(6)-O(1)	128.24(15)	C(28)-C(23)-C(24)	118.60(16)
C(1)-C(6)-O(1)	107.89(14)	C(28)-C(23)-C(16)	122.81(15)
C(6)-C(5)-C(4)	115.43(16)	C(24)-C(23)-C(16)	118.47(15)
C(5)-C(4)-C(3)	121.65(16)	C(25)-C(24)-C(23)	120.97(17)
C(2)-C(3)-C(4)	122.41(16)	C(24)-C(25)-C(26)	119.89(18)

C(27)-C(26)-C(25)	119.73(17)	C(34)-C(33)-C(32)	119.74(18)
C(26)-C(27)-C(28)	120.39(18)	C(33)-C(34)-C(35)	120.24(18)
C(27)-C(28)-C(23)	120.35(17)	C(30)-C(35)-C(34)	120.30(17)
C(11)-C(29)-C(30)	111.11(13)	C(41)-C(36)-C(37)	118.22(16)
C(11)-C(29)-C(36)	112.24(13)	C(41)-C(36)-C(29)	122.61(15)
C(30)-C(29)-C(36)	112.15(13)	C(37)-C(36)-C(29)	119.10(15)
C(31)-C(30)-C(35)	118.78(16)	C(38)-C(37)-C(36)	120.78(17)
C(31)-C(30)-C(29)	119.20(15)	C(37)-C(38)-C(39)	120.25(17)
C(35)-C(30)-C(29)	121.99(15)	C(40)-C(39)-C(38)	119.54(17)
C(32)-C(31)-C(30)	120.71(18)	C(39)-C(40)-C(41)	120.13(17)
C(33)-C(32)-C(31)	120.21(18)	C(36)-C(41)-C(40)	121.00(17)

XRAY: Compound 7

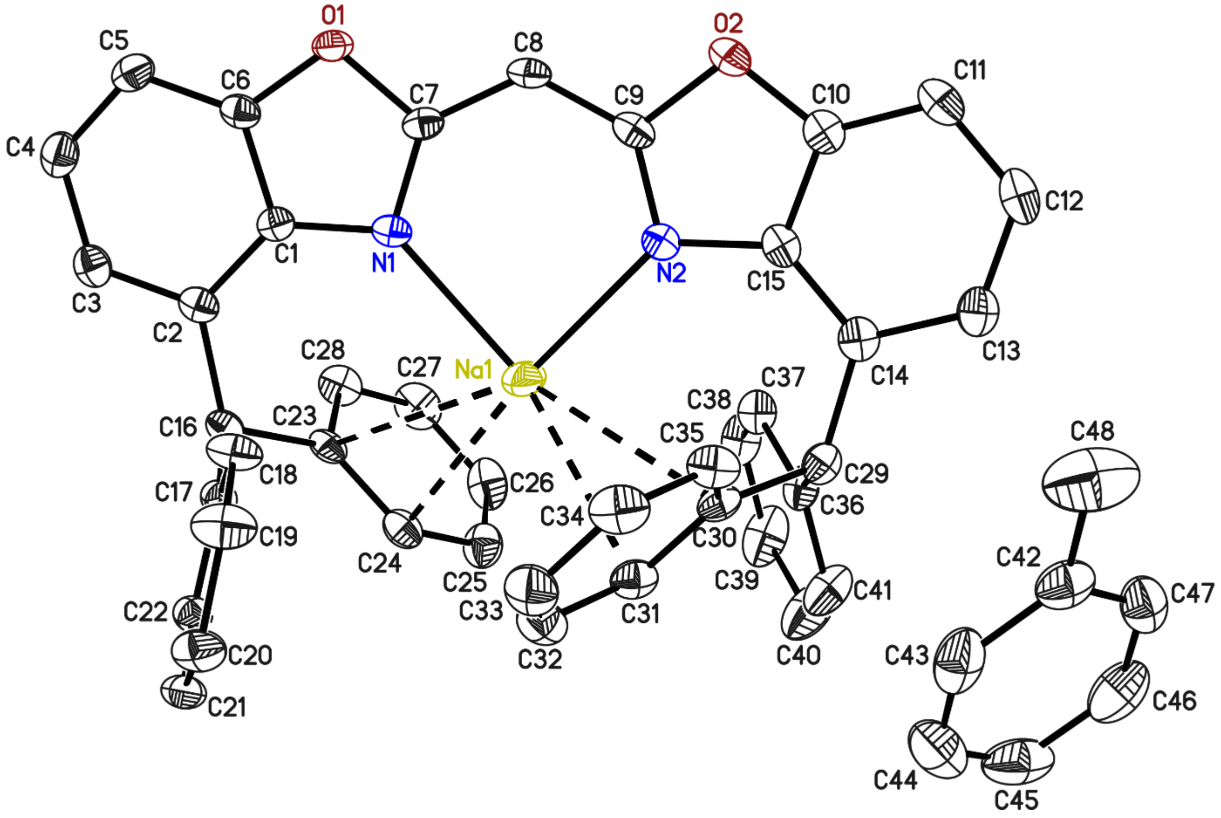

Figure S100. Asymmetric unit of 7. Displacement parameters are depicted at 50% probability. Hydrogen atoms are omitted for clarity.

Table S3 Bond lengths [Å] and angles [°] for **7**.

Na(1)-N(1)	2.3392(16)	C(1)-C(6)	1.389(2)
Na(1)-N(2)	2.3456(15)	C(1)-C(2)	1.403(2)
Na(1)-C(24)	2.8280(18)	N(2)-C(9)	1.328(2)
Na(1)-C(31)	2.8305(18)	N(2)-C(15)	1.401(2)
Na(1)-C(30)	3.0414(19)	O(2)-C(10)	1.380(2)
Na(1)-C(23)	3.0914(18)	O(2)-C(9)	1.3980(19)
O(1)-C(6)	1.375(2)	C(2)-C(3)	1.392(2)
O(1)-C(7)	1.3911(19)	C(2)-C(16)	1.515(2)
N(1)-C(7)	1.329(2)	C(3)-C(4)	1.387(3)
N(1)-C(1)	1.404(2)	C(4)-C(5)	1.383(3)

C(5)-C(6)	1.378(2)	C(38)-C(39)	1.377(3)
C(7)-C(8)	1.390(2)	C(39)-C(40)	1.378(3)
C(9)-C(8)	1.388(2)	C(40)-C(41)	1.389(3)
C(10)-C(11)	1.370(2)	C(42)-C(47)	1.384(3)
C(10)-C(15)	1.395(2)	C(42)-C(43)	1.384(3)
C(11)-C(12)	1.388(3)	C(42)-C(48)	1.489(3)
C(12)-C(13)	1.391(3)	C(43)-C(44)	1.382(3)
C(13)-C(14)	1.399(2)	C(44)-C(45)	1.363(3)
C(14)-C(15)	1.397(2)	C(45)-C(46)	1.377(3)
C(14)-C(29)	1.518(2)	C(46)-C(47)	1.372(3)
C(16)-C(17)	1.529(2)		
C(16)-C(23)	1.533(2)	N(1)-Na(1)-N(2)	86.53(5)
C(17)-C(18)	1.387(2)	N(1)-Na(1)-C(24)	98.73(6)
C(17)-C(22)	1.391(2)	N(2)-Na(1)-C(24)	162.94(6)
C(18)-C(19)	1.383(2)	N(1)-Na(1)-C(31)	153.19(6)
C(19)-C(20)	1.378(3)	N(2)-Na(1)-C(31)	98.07(6)
C(20)-C(21)	1.385(2)	C(24)-Na(1)-C(31)	84.58(6)
C(21)-C(22)	1.386(2)	N(1)-Na(1)-C(30)	144.52(6)
C(23)-C(24)	1.393(2)	N(2)-Na(1)-C(30)	71.08(5)
C(23)-C(28)	1.397(2)	C(24)-Na(1)-C(30)	110.77(5)
C(24)-C(25)	1.396(2)	C(31)-Na(1)-C(30)	27.03(5)
C(26)-C(27)	1.381(3)	N(1)-Na(1)-C(23)	72.22(5)
C(26)-C(25)	1.386(3)	N(2)-Na(1)-C(23)	151.09(5)
C(27)-C(28)	1.382(3)	C(24)-Na(1)-C(23)	26.74(5)
C(29)-C(36)	1.529(2)	C(31)-Na(1)-C(23)	109.78(5)
C(29)-C(30)	1.536(2)	C(30)-Na(1)-C(23)	136.67(5)
C(30)-C(31)	1.387(2)	C(6)-O(1)-C(7)	105.15(12)
C(30)-C(35)	1.399(2)	C(7)-N(1)-C(1)	104.96(13)
C(31)-C(32)	1.396(3)	C(7)-N(1)-Na(1)	120.60(11)
C(32)-C(33)	1.380(3)	C(1)-N(1)-Na(1)	134.44(10)
C(33)-C(34)	1.381(3)	C(6)-C(1)-C(2)	118.78(15)
C(34)-C(35)	1.383(3)	C(6)-C(1)-N(1)	108.90(14)
C(36)-C(41)	1.389(3)	C(2)-C(1)-N(1)	132.30(15)
C(36)-C(37)	1.391(2)	C(9)-N(2)-C(15)	105.09(13)
C(37)-C(38)	1.387(2)	C(9)-N(2)-Na(1)	120.90(11)

C(15)-N(2)-Na(1)	133.85(11)	C(22)-C(17)-C(16)	118.15(15)
C(10)-O(2)-C(9)	104.84(12)	C(19)-C(18)-C(17)	120.47(16)
C(3)-C(2)-C(1)	116.42(15)	C(20)-C(19)-C(18)	120.80(17)
C(3)-C(2)-C(16)	119.09(15)	C(19)-C(20)-C(21)	119.40(16)
C(1)-C(2)-C(16)	124.50(15)	C(20)-C(21)-C(22)	119.84(16)
C(4)-C(3)-C(2)	123.14(16)	C(21)-C(22)-C(17)	121.05(16)
C(5)-C(4)-C(3)	120.95(16)	C(24)-C(23)-C(28)	117.92(16)
C(6)-C(5)-C(4)	115.52(16)	C(24)-C(23)-C(16)	123.60(15)
N(1)-C(7)-C(8)	132.74(15)	C(28)-C(23)-C(16)	118.27(15)
N(1)-C(7)-O(1)	112.98(14)	C(24)-C(23)-Na(1)	66.03(9)
C(8)-C(7)-O(1)	114.27(14)	C(28)-C(23)-Na(1)	106.03(11)
O(1)-C(6)-C(5)	126.78(15)	C(16)-C(23)-Na(1)	102.53(10)
O(1)-C(6)-C(1)	108.00(14)	C(23)-C(24)-C(25)	120.79(16)
C(5)-C(6)-C(1)	125.18(16)	C(23)-C(24)-Na(1)	87.23(10)
N(2)-C(9)-C(8)	132.13(15)	C(25)-C(24)-Na(1)	97.41(11)
N(2)-C(9)-O(2)	113.15(14)	C(27)-C(26)-C(25)	118.99(17)
C(8)-C(9)-O(2)	114.71(14)	C(26)-C(25)-C(24)	120.39(18)
C(9)-C(8)-C(7)	126.77(15)	C(26)-C(27)-C(28)	120.84(17)
C(11)-C(10)-O(2)	127.35(16)	C(27)-C(28)-C(23)	121.04(18)
C(11)-C(10)-C(15)	124.76(17)	C(14)-C(29)-C(36)	113.09(14)
O(2)-C(10)-C(15)	107.87(14)	C(14)-C(29)-C(30)	112.83(14)
C(10)-C(11)-C(12)	115.90(17)	C(36)-C(29)-C(30)	112.99(14)
C(11)-C(12)-C(13)	120.85(17)	C(31)-C(30)-C(35)	117.70(16)
C(12)-C(13)-C(14)	122.86(18)	C(31)-C(30)-C(29)	123.20(15)
C(15)-C(14)-C(13)	116.34(16)	C(35)-C(30)-C(29)	118.93(15)
C(15)-C(14)-C(29)	123.66(15)	C(31)-C(30)-Na(1)	67.98(9)
C(13)-C(14)-C(29)	120.00(16)	C(35)-C(30)-Na(1)	104.44(11)
C(10)-C(15)-C(14)	119.28(15)	C(29)-C(30)-Na(1)	101.42(10)
C(10)-C(15)-N(2)	109.04(15)	C(30)-C(31)-C(32)	120.84(16)
C(14)-C(15)-N(2)	131.67(15)	C(30)-C(31)-Na(1)	84.99(10)
C(2)-C(16)-C(17)	113.00(13)	C(32)-C(31)-Na(1)	96.82(11)
C(2)-C(16)-C(23)	113.49(13)	C(33)-C(32)-C(31)	120.45(18)
C(17)-C(16)-C(23)	114.03(14)	C(32)-C(33)-C(34)	119.38(18)
C(18)-C(17)-C(22)	118.41(16)	C(33)-C(34)-C(35)	120.21(17)
C(18)-C(17)-C(16)	123.41(15)	C(34)-C(35)-C(30)	121.41(17)

C(41)-C(36)-C(37)	118.44(16)	C(47)-C(42)-C(43)	118.02(19)
C(41)-C(36)-C(29)	118.77(15)	C(47)-C(42)-C(48)	121.3(2)
C(37)-C(36)-C(29)	122.78(16)	C(43)-C(42)-C(48)	120.7(2)
C(38)-C(37)-C(36)	120.54(18)	C(44)-C(43)-C(42)	121.1(2)
C(39)-C(38)-C(37)	120.34(18)	C(45)-C(44)-C(43)	119.7(2)
C(38)-C(39)-C(40)	119.82(18)	C(44)-C(45)-C(46)	120.1(2)
C(39)-C(40)-C(41)	120.06(19)	C(47)-C(46)-C(45)	120.1(2)
C(36)-C(41)-C(40)	120.79(18)	C(46)-C(47)-C(42)	120.9(2)

XRAY: Compound 8m

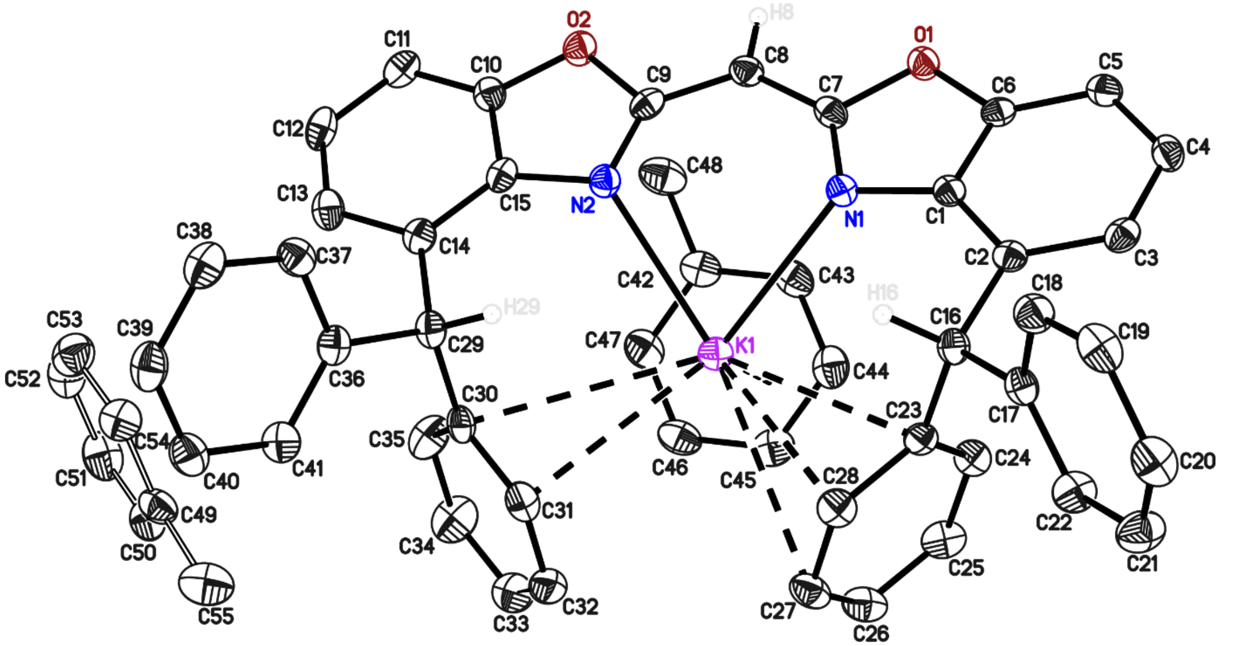

Figure 101. Asymmetric unit of 8m. Displacement parameters are depicted at 50% probability. Hydrogen atoms except the hydrogen atoms bound to the methylene backbone (H8) and benzylic groups (H16, H29) are omitted for clarity.

The toluene molecule C49 to C55 is disordered about an inversion centre. The disordered group was refined with distance restraints and restraints for the anisotropic displacement parameter.

Table 4. Bond lengths [Å] and angles [°] for **8m**.

K(1)-N(2)	2.6982(18)	O(1)-C(6)	1.378(2)
K(1)-N(1)	2.7768(18)	O(1)-C(7)	1.409(2)
K(1)-C(44)	3.098(2)	N(1)-C(7)	1.330(2)
K(1)-C(45)	3.152(2)	N(1)-C(1)	1.397(2)
K(1)-C(31)	3.224(2)	C(1)-C(6)	1.394(3)
K(1)-C(28)	3.236(2)	C(1)-C(2)	1.401(3)
K(1)-C(23)	3.314(2)	C(2)-C(3)	1.399(3)
K(1)-C(43)	3.344(2)	C(2)-C(16)	1.523(3)
K(1)-C(46)	3.417(2)	N(2)-C(9)	1.325(2)
K(1)-C(30)	3.467(2)	N(2)-C(15)	1.404(2)
K(1)-C(9)	3.486(2)	O(2)-C(10)	1.379(2)
K(1)-C(27)	3.503(2)	O(2)-C(9)	1.400(2)

C(3)-C(4)	1.397(3)	C(36)-C(41)	1.385(3)
C(6)-C(5)	1.376(3)	C(36)-C(37)	1.396(3)
C(5)-C(4)	1.394(3)	C(37)-C(38)	1.388(3)
C(7)-C(8)	1.391(3)	C(38)-C(39)	1.384(3)
C(9)-C(8)	1.396(3)	C(39)-C(40)	1.376(3)
C(8)-H(8)	0.9500	C(40)-C(41)	1.400(3)
C(10)-C(11)	1.376(3)	C(42)-C(43)	1.389(3)
C(10)-C(15)	1.396(3)	C(42)-C(47)	1.398(3)
C(11)-C(12)	1.393(3)	C(42)-C(48)	1.508(3)
C(12)-C(13)	1.391(3)	C(43)-C(44)	1.386(3)
C(13)-C(14)	1.401(3)	C(44)-C(45)	1.389(3)
C(14)-C(15)	1.390(3)	C(45)-C(46)	1.379(3)
C(14)-C(29)	1.522(3)	C(46)-C(47)	1.387(3)
C(16)-C(23)	1.525(3)	C(49)-C(54)	1.388(8)
C(16)-C(17)	1.532(3)	C(49)-C(50)	1.394(8)
C(17)-C(22)	1.387(3)	C(49)-C(55)	1.497(7)
C(17)-C(18)	1.397(3)	C(50)-C(51)	1.373(8)
C(18)-C(19)	1.388(3)	C(51)-C(52)	1.378(8)
C(19)-C(20)	1.385(3)	C(52)-C(53)	1.372(8)
C(20)-C(21)	1.378(3)	C(53)-C(54)	1.382(7)
C(21)-C(22)	1.390(3)		
C(23)-C(24)	1.392(3)	N(2)-K(1)-N(1)	70.73(5)
C(23)-C(28)	1.399(3)	N(2)-K(1)-C(44)	110.84(6)
C(24)-C(25)	1.395(3)	N(1)-K(1)-C(44)	89.55(6)
C(25)-C(26)	1.378(3)	N(2)-K(1)-C(45)	120.92(6)
C(26)-C(27)	1.385(3)	N(1)-K(1)-C(45)	114.96(6)
C(27)-C(28)	1.389(3)	C(44)-K(1)-C(45)	25.65(5)
C(30)-C(35)	1.394(3)	N(2)-K(1)-C(31)	94.37(6)
C(30)-C(31)	1.397(3)	N(1)-K(1)-C(31)	147.13(5)
C(30)-C(29)	1.528(3)	C(44)-K(1)-C(31)	123.31(6)
C(29)-C(36)	1.531(3)	C(45)-K(1)-C(31)	97.87(6)
C(31)-C(32)	1.386(3)	N(2)-K(1)-C(28)	136.96(5)
C(32)-C(33)	1.382(3)	N(1)-K(1)-C(28)	89.21(6)
C(33)-C(34)	1.383(3)	C(44)-K(1)-C(28)	106.50(6)
C(34)-C(35)	1.385(3)	C(45)-K(1)-C(28)	101.98(6)

C(31)-K(1)-C(28)	82.22(6)
N(2)-K(1)-C(23)	132.30(5)
N(1)-K(1)-C(23)	68.45(5)
C(44)-K(1)-C(23)	92.54(6)
C(45)-K(1)-C(23)	98.29(6)
C(31)-K(1)-C(23)	106.85(6)
C(28)-K(1)-C(23)	24.63(5)
N(2)-K(1)-C(43)	86.40(6)
N(1)-K(1)-C(43)	80.10(5)
C(44)-K(1)-C(43)	24.46(5)
C(45)-K(1)-C(43)	43.29(6)
C(31)-K(1)-C(43)	129.40(6)
C(28)-K(1)-C(43)	128.32(6)
C(23)-K(1)-C(43)	109.43(6)
N(2)-K(1)-C(46)	103.82(5)
N(1)-K(1)-C(46)	128.28(5)
C(44)-K(1)-C(46)	42.75(6)
C(45)-K(1)-C(46)	23.79(5)
C(31)-K(1)-C(46)	83.04(6)
C(28)-K(1)-C(46)	118.14(6)
C(23)-K(1)-C(46)	120.51(5)
C(43)-K(1)-C(46)	48.27(5)
N(2)-K(1)-C(30)	72.81(5)
N(1)-K(1)-C(30)	138.60(5)
C(44)-K(1)-C(30)	121.73(6)
C(45)-K(1)-C(30)	100.23(6)
C(31)-K(1)-C(30)	23.74(5)
C(28)-K(1)-C(30)	104.72(5)
C(23)-K(1)-C(30)	129.07(5)
C(43)-K(1)-C(30)	116.52(5)
C(46)-K(1)-C(30)	79.16(5)
N(2)-K(1)-C(9)	19.99(5)
N(1)-K(1)-C(9)	58.09(5)
C(44)-K(1)-C(9)	94.07(6)
C(45)-K(1)-C(9)	109.86(6)
C(31)-K(1)-C(9)	113.39(5)
C(28)-K(1)-C(9)	141.44(5)
C(23)-K(1)-C(9)	125.99(5)
C(43)-K(1)-C(9)	69.88(5)
C(46)-K(1)-C(9)	99.20(5)
C(30)-K(1)-C(9)	90.70(5)
N(2)-K(1)-C(27)	153.75(5)
N(1)-K(1)-C(27)	109.97(5)
C(44)-K(1)-C(27)	95.39(6)
C(45)-K(1)-C(27)	83.37(6)
C(31)-K(1)-C(27)	70.77(5)
C(28)-K(1)-C(27)	23.36(5)
C(23)-K(1)-C(27)	41.60(5)
C(43)-K(1)-C(27)	119.81(6)
C(46)-K(1)-C(27)	95.90(5)
C(30)-K(1)-C(27)	94.49(5)
C(9)-K(1)-C(27)	164.71(5)
C(6)-O(1)-C(7)	104.64(14)
C(7)-N(1)-C(1)	104.68(15)
C(7)-N(1)-K(1)	112.71(12)
C(1)-N(1)-K(1)	128.82(12)
C(6)-C(1)-N(1)	109.79(16)
C(6)-C(1)-C(2)	119.81(17)
N(1)-C(1)-C(2)	130.38(17)
C(3)-C(2)-C(1)	116.41(17)
C(3)-C(2)-C(16)	123.95(17)
C(1)-C(2)-C(16)	119.64(17)
C(9)-N(2)-C(15)	105.01(16)
C(9)-N(2)-K(1)	115.87(12)
C(15)-N(2)-K(1)	125.40(12)
C(10)-O(2)-C(9)	104.74(14)
C(4)-C(3)-C(2)	122.44(18)
C(5)-C(6)-O(1)	127.93(17)
C(5)-C(6)-C(1)	124.36(18)
O(1)-C(6)-C(1)	107.71(16)

C(6)-C(5)-C(4)	115.83(18)	C(19)-C(18)-C(17)	121.09(19)
C(5)-C(4)-C(3)	121.14(18)	C(20)-C(19)-C(18)	119.95(19)
N(1)-C(7)-C(8)	132.23(18)	C(21)-C(20)-C(19)	119.60(19)
N(1)-C(7)-O(1)	113.12(16)	C(20)-C(21)-C(22)	120.4(2)
C(8)-C(7)-O(1)	114.62(16)	C(17)-C(22)-C(21)	121.00(19)
N(1)-C(7)-K(1)	46.83(9)	C(24)-C(23)-C(28)	118.05(18)
C(8)-C(7)-K(1)	92.72(12)	C(24)-C(23)-C(16)	122.56(17)
O(1)-C(7)-K(1)	142.69(12)	C(28)-C(23)-C(16)	119.36(17)
N(2)-C(9)-C(8)	132.10(18)	C(24)-C(23)-K(1)	91.39(12)
N(2)-C(9)-O(2)	113.33(17)	C(28)-C(23)-K(1)	74.57(11)
C(8)-C(9)-O(2)	114.55(16)	C(16)-C(23)-K(1)	105.15(11)
N(2)-C(9)-K(1)	44.14(9)	C(23)-C(24)-C(25)	120.73(19)
C(8)-C(9)-K(1)	93.69(11)	C(26)-C(25)-C(24)	120.4(2)
O(2)-C(9)-K(1)	142.51(12)	C(25)-C(26)-C(27)	119.64(19)
H(8)-C(8)-C(7)	117.1	C(26)-C(27)-C(28)	120.1(2)
H(8)-C(8)-C(9)	117.1	C(26)-C(27)-K(1)	91.79(12)
C(7)-C(8)-C(9)	125.72(18)	C(28)-C(27)-K(1)	67.50(11)
C(11)-C(10)-O(2)	127.99(18)	C(27)-C(28)-C(23)	121.02(19)
C(11)-C(10)-C(15)	124.06(19)	C(27)-C(28)-K(1)	89.14(12)
O(2)-C(10)-C(15)	107.95(16)	C(23)-C(28)-K(1)	80.80(11)
C(10)-C(11)-C(12)	115.43(18)	C(35)-C(30)-C(31)	117.69(19)
C(13)-C(12)-C(11)	121.75(19)	C(35)-C(30)-C(29)	122.31(18)
C(12)-C(13)-C(14)	122.09(19)	C(31)-C(30)-C(29)	119.99(18)
C(15)-C(14)-C(13)	116.35(18)	C(35)-C(30)-K(1)	111.32(13)
C(15)-C(14)-C(29)	120.68(17)	C(31)-C(30)-K(1)	68.33(11)
C(13)-C(14)-C(29)	122.98(18)	C(29)-C(30)-K(1)	90.87(11)
C(14)-C(15)-C(10)	120.31(17)	C(14)-C(29)-C(30)	112.43(16)
C(14)-C(15)-N(2)	130.74(17)	C(14)-C(29)-C(36)	112.01(16)
C(10)-C(15)-N(2)	108.96(17)	C(30)-C(29)-C(36)	114.06(16)
C(2)-C(16)-C(23)	113.08(15)	C(32)-C(31)-C(30)	121.2(2)
C(2)-C(16)-C(17)	112.48(16)	C(32)-C(31)-K(1)	102.54(13)
C(23)-C(16)-C(17)	112.22(15)	C(30)-C(31)-K(1)	87.93(12)
C(22)-C(17)-C(18)	117.98(18)	C(33)-C(32)-C(31)	120.2(2)
C(22)-C(17)-C(16)	122.84(17)	C(32)-C(33)-C(34)	119.4(2)
C(18)-C(17)-C(16)	119.18(17)	C(33)-C(34)-C(35)	120.4(2)

C(41)-C(36)-C(37)	118.18(18)	C(45)-C(44)-K(1)	79.37(12)
C(41)-C(36)-C(29)	123.87(18)	C(46)-C(45)-C(44)	119.7(2)
C(37)-C(36)-C(29)	117.90(18)	C(46)-C(45)-K(1)	88.94(13)
C(34)-C(35)-C(30)	121.1(2)	C(44)-C(45)-K(1)	74.98(12)
C(38)-C(37)-C(36)	121.15(19)	C(45)-C(46)-C(47)	120.2(2)
C(39)-C(38)-C(37)	120.01(19)	C(45)-C(46)-K(1)	67.27(12)
C(40)-C(39)-C(38)	119.61(19)	C(47)-C(46)-K(1)	87.36(13)
C(39)-C(40)-C(41)	120.4(2)	C(46)-C(47)-C(42)	120.9(2)
C(36)-C(41)-C(40)	120.65(19)	C(54)-C(49)-C(50)	117.8(5)
C(43)-C(42)-C(47)	118.2(2)	C(54)-C(49)-C(55)	121.7(6)
C(43)-C(42)-C(48)	121.44(19)	C(50)-C(49)-C(55)	120.4(6)
C(47)-C(42)-C(48)	120.4(2)	C(51)-C(50)-C(49)	121.3(7)
C(44)-C(43)-C(42)	121.0(2)	C(50)-C(51)-C(52)	120.0(7)
C(44)-C(43)-K(1)	67.76(12)	C(53)-C(52)-C(51)	119.6(6)
C(42)-C(43)-K(1)	90.17(13)	C(52)-C(53)-C(54)	120.5(7)
C(43)-C(44)-C(45)	120.0(2)	C(53)-C(54)-C(49)	120.7(7)
C(43)-C(44)-K(1)	87.77(13)		

XRAY: Compound 8d

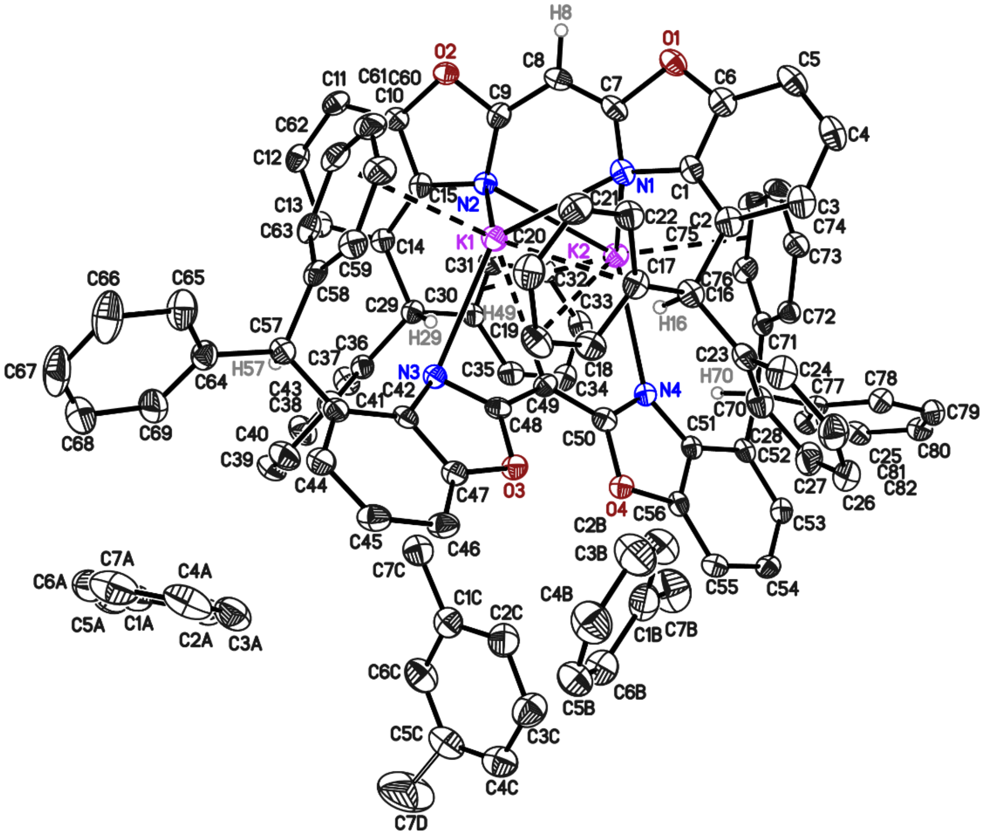

Figure S102. Asymmetric unit of 8d. Displacement parameters are depicted at 50% probability. Hydrogen atoms except the hydrogen atoms bound to the methylene backbone (H8, H49) and benzylic groups (H16, H29, H57, H70) are omitted for clarity.

The methyl group of the toluene molecule C1C to C7C is disordered over two postions. The occupancy of the minor position refined to 0.150(8). All toluene molecules are refined with distance restraints and the disordered one additionally with restraints for the anisotropic displacement parameter. The hydrogen atom H49 was refined without any restraints or constraints.

Table S5. Bond lengths [Å] and angles [°] for **8d**.

N(1)-C(7)	1.328(4)	O(1)-C(6)	1.378(4)
N(1)-C(1)	1.406(4)	O(1)-C(7)	1.397(3)
N(1)-K(1)	2.812(2)	C(1)-C(6)	1.384(4)
N(1)-K(2)	3.015(2)	C(1)-C(2)	1.394(4)

K(1)-N(3)	2.775(2)	C(4)-C(5)	1.387(4)
K(1)-H(49)	2.89(3)	N(4)-C(50)	1.323(4)
K(1)-N(2)	2.914(2)	N(4)-C(51)	1.402(3)
K(1)-C(60)	3.134(3)	C(5)-C(6)	1.378(4)
K(1)-C(61)	3.151(3)	C(7)-C(8)	1.392(4)
K(1)-C(48)	3.170(3)	C(8)-C(9)	1.396(4)
K(1)-C(49)	3.254(3)	C(10)-C(11)	1.371(4)
K(1)-C(62)	3.267(3)	C(10)-C(15)	1.385(4)
K(1)-C(59)	3.267(3)	C(11)-C(12)	1.385(4)
K(1)-C(17)	3.357(3)	C(12)-C(13)	1.390(4)
K(1)-C(63)	3.403(3)	C(13)-C(14)	1.396(4)
K(2)-H(49)	2.77(3)	C(14)-C(15)	1.399(4)
K(2)-N(4)	2.812(2)	C(14)-C(29)	1.521(4)
K(2)-N(2)	2.989(2)	C(16)-C(23)	1.512(4)
K(2)-C(76)	3.208(3)	C(16)-C(17)	1.535(4)
K(2)-C(75)	3.235(3)	C(17)-C(18)	1.386(4)
K(2)-C(31)	3.338(3)	C(17)-C(22)	1.399(4)
K(2)-C(30)	3.358(3)	C(18)-C(19)	1.383(4)
K(2)-C(71)	3.379(3)	C(19)-C(20)	1.393(4)
K(2)-C(32)	3.398(3)	C(20)-C(21)	1.372(4)
K(2)-C(74)	3.410(3)	C(21)-C(22)	1.395(4)
K(2)-C(35)	3.439(3)	C(23)-C(24)	1.390(4)
C(2)-C(3)	1.402(4)	C(23)-C(28)	1.396(4)
C(2)-C(16)	1.517(4)	C(24)-C(25)	1.378(5)
O(2)-C(10)	1.383(3)	C(25)-C(26)	1.388(5)
O(2)-C(9)	1.393(3)	C(26)-C(27)	1.383(5)
N(2)-C(9)	1.332(4)	C(27)-C(28)	1.373(4)
N(2)-C(15)	1.403(4)	C(29)-C(36)	1.516(4)
N(3)-C(48)	1.335(4)	C(29)-C(30)	1.532(4)
N(3)-C(42)	1.389(3)	C(30)-C(31)	1.392(4)
C(3)-C(4)	1.386(4)	C(30)-C(35)	1.398(4)
O(3)-C(47)	1.385(3)	C(31)-C(32)	1.385(4)
O(3)-C(48)	1.387(3)	C(32)-C(33)	1.391(4)
O(4)-C(50)	1.378(3)	C(33)-C(34)	1.378(4)
O(4)-C(56)	1.388(3)	C(34)-C(35)	1.388(4)

C(36)-C(41)	1.373(4)	C(67)-C(68)	1.385(6)
C(36)-C(37)	1.396(4)	C(68)-C(69)	1.394(5)
C(37)-C(38)	1.384(4)	C(70)-C(71)	1.528(4)
C(38)-C(39)	1.367(5)	C(70)-C(77)	1.529(4)
C(39)-C(40)	1.388(5)	C(71)-C(76)	1.390(4)
C(40)-C(41)	1.388(5)	C(71)-C(72)	1.393(4)
C(42)-C(47)	1.384(4)	C(72)-C(73)	1.389(4)
C(42)-C(43)	1.400(4)	C(73)-C(74)	1.387(4)
C(43)-C(44)	1.392(4)	C(74)-C(75)	1.384(4)
C(43)-C(57)	1.520(4)	C(75)-C(76)	1.391(4)
C(44)-C(45)	1.392(4)	C(77)-C(78)	1.388(4)
C(45)-C(46)	1.392(4)	C(77)-C(82)	1.390(4)
C(46)-C(47)	1.373(4)	C(78)-C(79)	1.382(4)
C(48)-C(49)	1.391(4)	C(79)-C(80)	1.389(4)
C(49)-H(49)	1.01(3)	C(80)-C(81)	1.377(4)
C(49)-C(50)	1.400(4)	C(81)-C(82)	1.391(4)
C(51)-C(56)	1.385(4)	C(1A)-C(2A)	1.385(5)
C(51)-C(52)	1.401(4)	C(1A)-C(6A)	1.386(5)
C(52)-C(53)	1.394(4)	C(1A)-C(7A)	1.498(5)
C(52)-C(70)	1.523(4)	C(2A)-C(3A)	1.385(5)
C(53)-C(54)	1.395(4)	C(3A)-C(4A)	1.372(5)
C(54)-C(55)	1.386(4)	C(4A)-C(5A)	1.383(6)
C(55)-C(56)	1.374(4)	C(5A)-C(6A)	1.387(5)
C(57)-C(58)	1.516(4)	C(1B)-C(2B)	1.388(5)
C(57)-C(64)	1.526(4)	C(1B)-C(6B)	1.396(5)
C(58)-C(63)	1.388(4)	C(1B)-C(7B)	1.494(5)
C(58)-C(59)	1.401(4)	C(2B)-C(3B)	1.370(5)
C(59)-C(60)	1.376(4)	C(3B)-C(4B)	1.376(5)
C(60)-C(61)	1.377(5)	C(4B)-C(5B)	1.377(5)
C(61)-C(62)	1.393(5)	C(5B)-C(6B)	1.380(5)
C(62)-C(63)	1.390(4)	C(1C)-C(2C)	1.387(4)
C(64)-C(69)	1.375(5)	C(1C)-C(6C)	1.398(4)
C(64)-C(65)	1.402(4)	C(1C)-C(7C)	1.486(5)
C(65)-C(66)	1.382(5)	C(2C)-C(3C)	1.386(5)
C(66)-C(67)	1.366(6)	C(3C)-C(4C)	1.390(5)

C(4C)-C(5C)	1.370(5)
C(5C)-C(6C)	1.375(5)
C(5C)-C(7D)	1.483(13)
C(7)-N(1)-C(1)	104.7(2)
C(7)-N(1)-K(1)	115.36(18)
C(1)-N(1)-K(1)	128.23(17)
C(7)-N(1)-K(2)	97.49(16)
C(1)-N(1)-K(2)	115.06(16)
K(1)-N(1)-K(2)	91.42(7)
C(6)-O(1)-C(7)	104.8(2)
C(6)-C(1)-C(2)	120.4(3)
C(6)-C(1)-N(1)	109.1(3)
C(2)-C(1)-N(1)	130.4(3)
N(3)-K(1)-N(1)	135.95(7)
N(3)-K(1)-H(49)	54.9(5)
N(1)-K(1)-H(49)	83.9(5)
N(3)-K(1)-N(2)	104.84(7)
N(1)-K(1)-N(2)	67.57(7)
H(49)-K(1)-N(2)	68.1(6)
N(3)-K(1)-C(60)	97.58(8)
N(1)-K(1)-C(60)	125.23(8)
H(49)-K(1)-C(60)	150.7(5)
N(2)-K(1)-C(60)	116.48(8)
N(3)-K(1)-C(61)	108.74(8)
N(1)-K(1)-C(61)	114.61(8)
H(49)-K(1)-C(61)	145.4(5)
N(2)-K(1)-C(61)	91.36(8)
C(60)-K(1)-C(61)	25.32(8)
N(3)-K(1)-C(48)	24.84(7)
N(1)-K(1)-C(48)	112.02(7)
H(49)-K(1)-C(48)	39.1(5)
N(2)-K(1)-C(48)	103.92(7)
C(60)-K(1)-C(48)	118.42(8)
C(61)-K(1)-C(48)	133.29(8)
N(3)-K(1)-C(49)	46.54(7)
N(1)-K(1)-C(49)	89.48(7)
H(49)-K(1)-C(49)	17.6(5)
N(2)-K(1)-C(49)	85.65(7)
C(60)-K(1)-C(49)	143.27(8)
C(61)-K(1)-C(49)	152.48(8)
C(48)-K(1)-C(49)	24.98(7)
N(3)-K(1)-C(62)	93.64(8)
N(1)-K(1)-C(62)	123.38(8)
H(49)-K(1)-C(62)	120.4(5)
N(2)-K(1)-C(62)	75.82(8)
C(60)-K(1)-C(62)	43.70(8)
C(61)-K(1)-C(62)	24.98(8)
C(48)-K(1)-C(62)	117.83(8)
C(49)-K(1)-C(62)	129.71(8)
N(3)-K(1)-C(59)	72.98(7)
N(1)-K(1)-C(59)	148.37(7)
H(49)-K(1)-C(59)	127.2(5)
N(2)-K(1)-C(59)	124.83(7)
C(60)-K(1)-C(59)	24.72(8)
C(61)-K(1)-C(59)	43.86(8)
C(48)-K(1)-C(59)	93.94(8)
C(49)-K(1)-C(59)	118.62(8)
C(62)-K(1)-C(59)	50.05(8)
N(3)-K(1)-C(17)	96.19(8)
N(1)-K(1)-C(17)	69.82(7)
H(49)-K(1)-C(17)	94.6(5)
N(2)-K(1)-C(17)	135.24(7)
C(60)-K(1)-C(17)	98.85(8)
C(61)-K(1)-C(17)	118.75(8)
C(48)-K(1)-C(17)	79.86(8)
C(49)-K(1)-C(17)	80.88(7)
C(62)-K(1)-C(17)	142.32(8)
C(59)-K(1)-C(17)	98.85(8)
N(3)-K(1)-C(63)	69.78(7)

N(1)-K(1)-C(63)	145.05(7)	C(76)-K(2)-C(30)	116.55(8)
H(49)-K(1)-C(63)	105.3(5)	C(75)-K(2)-C(30)	132.31(8)
N(2)-K(1)-C(63)	84.44(7)	C(31)-K(2)-C(30)	24.00(7)
C(60)-K(1)-C(63)	49.65(8)	H(49)-K(2)-C(71)	115.2(6)
C(61)-K(1)-C(63)	42.95(8)	N(4)-K(2)-C(71)	59.80(7)
C(48)-K(1)-C(63)	94.23(7)	N(2)-K(2)-C(71)	176.18(7)
C(49)-K(1)-C(63)	109.55(7)	N(1)-K(2)-C(71)	115.42(7)
C(62)-K(1)-C(63)	23.94(8)	C(76)-K(2)-C(71)	24.19(7)
C(59)-K(1)-C(63)	41.94(7)	C(75)-K(2)-C(71)	42.87(7)
C(17)-K(1)-C(63)	140.26(7)	C(31)-K(2)-C(71)	116.57(7)
H(49)-K(2)-N(4)	55.5(6)	C(30)-K(2)-C(71)	118.78(7)
H(49)-K(2)-N(2)	68.6(6)	H(49)-K(2)-C(32)	100.0(6)
N(4)-K(2)-N(2)	124.01(7)	N(4)-K(2)-C(32)	100.91(7)
H(49)-K(2)-N(1)	82.4(6)	N(2)-K(2)-C(32)	84.98(7)
N(4)-K(2)-N(1)	107.88(7)	N(1)-K(2)-C(32)	145.97(7)
N(2)-K(2)-N(1)	64.07(6)	C(76)-K(2)-C(32)	80.68(8)
H(49)-K(2)-C(76)	137.2(6)	C(75)-K(2)-C(32)	90.86(8)
N(4)-K(2)-C(76)	82.11(7)	C(31)-K(2)-C(32)	23.71(7)
N(2)-K(2)-C(76)	152.35(7)	C(30)-K(2)-C(32)	41.93(7)
N(1)-K(2)-C(76)	120.43(7)	C(71)-K(2)-C(32)	94.48(7)
H(49)-K(2)-C(75)	156.8(6)	H(49)-K(2)-C(74)	141.7(6)
N(4)-K(2)-C(75)	102.45(7)	N(4)-K(2)-C(74)	99.93(7)
N(2)-K(2)-C(75)	133.33(7)	N(2)-K(2)-C(74)	128.23(7)
N(1)-K(2)-C(75)	100.06(7)	N(1)-K(2)-C(74)	78.33(7)
C(76)-K(2)-C(75)	24.92(8)	C(76)-K(2)-C(74)	42.43(8)
H(49)-K(2)-C(31)	82.5(6)	C(75)-K(2)-C(74)	23.85(8)
N(4)-K(2)-C(31)	103.80(7)	C(31)-K(2)-C(74)	135.13(8)
N(2)-K(2)-C(31)	63.55(7)	C(30)-K(2)-C(74)	156.16(8)
N(1)-K(2)-C(31)	127.48(7)	C(71)-K(2)-C(74)	48.69(7)
C(76)-K(2)-C(31)	104.38(8)	C(32)-K(2)-C(74)	114.48(8)
C(75)-K(2)-C(31)	112.68(8)	H(49)-K(2)-C(35)	55.4(6)
H(49)-K(2)-C(30)	59.1(6)	N(4)-K(2)-C(35)	62.85(7)
N(4)-K(2)-C(30)	84.82(7)	N(2)-K(2)-C(35)	84.61(7)
N(2)-K(2)-C(30)	63.05(7)	N(1)-K(2)-C(35)	135.04(7)
N(1)-K(2)-C(30)	122.71(7)	C(76)-K(2)-C(35)	102.46(8)

C(75)-K(2)-C(35)	124.77(8)
C(31)-K(2)-C(35)	41.29(7)
C(30)-K(2)-C(35)	23.70(7)
C(71)-K(2)-C(35)	97.84(7)
C(32)-K(2)-C(35)	47.65(7)
C(74)-K(2)-C(35)	144.54(7)
C(1)-C(2)-C(3)	115.8(3)
C(1)-C(2)-C(16)	120.3(3)
C(3)-C(2)-C(16)	123.8(3)
C(10)-O(2)-C(9)	105.0(2)
C(9)-N(2)-C(15)	104.3(2)
C(9)-N(2)-K(1)	103.48(17)
C(15)-N(2)-K(1)	116.60(16)
C(9)-N(2)-K(2)	107.94(17)
C(15)-N(2)-K(2)	131.43(17)
K(1)-N(2)-K(2)	89.98(7)
C(48)-N(3)-C(42)	104.3(2)
C(48)-N(3)-K(1)	94.36(16)
C(42)-N(3)-K(1)	123.12(17)
C(4)-C(3)-C(2)	122.4(3)
C(47)-O(3)-C(48)	104.4(2)
C(50)-O(4)-C(56)	104.1(2)
C(3)-C(4)-C(5)	121.8(3)
C(50)-N(4)-C(51)	104.1(2)
C(50)-N(4)-K(2)	109.99(17)
C(51)-N(4)-K(2)	145.07(17)
C(6)-C(5)-C(4)	115.2(3)
C(5)-C(6)-O(1)	127.6(3)
C(5)-C(6)-C(1)	124.3(3)
O(1)-C(6)-C(1)	108.1(2)
N(1)-C(7)-C(8)	131.6(3)
N(1)-C(7)-O(1)	113.2(2)
C(8)-C(7)-O(1)	115.2(2)
N(1)-C(7)-K(2)	60.08(14)
C(8)-C(7)-K(2)	93.34(17)
O(1)-C(7)-K(2)	124.31(17)
C(7)-C(8)-C(9)	125.4(3)
N(2)-C(9)-O(2)	113.4(2)
N(2)-C(9)-C(8)	131.9(3)
O(2)-C(9)-C(8)	114.7(2)
N(2)-C(9)-K(1)	54.63(14)
O(2)-C(9)-K(1)	128.39(17)
C(8)-C(9)-K(1)	95.21(17)
C(11)-C(10)-O(2)	127.5(3)
C(11)-C(10)-C(15)	125.0(3)
O(2)-C(10)-C(15)	107.5(2)
C(10)-C(11)-C(12)	115.5(3)
C(11)-C(12)-C(13)	121.3(3)
C(12)-C(13)-C(14)	122.5(3)
C(13)-C(14)-C(15)	116.2(3)
C(13)-C(14)-C(29)	123.6(3)
C(15)-C(14)-C(29)	120.1(2)
C(10)-C(15)-C(14)	119.4(3)
C(10)-C(15)-N(2)	109.8(2)
C(14)-C(15)-N(2)	130.7(3)
C(23)-C(16)-C(2)	112.9(2)
C(23)-C(16)-C(17)	112.2(2)
C(2)-C(16)-C(17)	112.6(2)
C(18)-C(17)-C(22)	118.4(3)
C(18)-C(17)-C(16)	119.2(3)
C(22)-C(17)-C(16)	122.4(3)
C(18)-C(17)-K(1)	84.45(17)
C(22)-C(17)-K(1)	91.09(17)
C(16)-C(17)-K(1)	94.88(16)
C(19)-C(18)-C(17)	121.4(3)
C(19)-C(18)-K(1)	97.40(19)
C(17)-C(18)-K(1)	72.37(16)
C(18)-C(19)-C(20)	120.0(3)
C(21)-C(20)-C(19)	119.3(3)
C(20)-C(21)-C(22)	121.0(3)

C(21)-C(22)-C(17)	120.0(3)
C(24)-C(23)-C(28)	117.9(3)
C(24)-C(23)-C(16)	122.5(3)
C(28)-C(23)-C(16)	119.6(3)
C(25)-C(24)-C(23)	120.9(3)
C(24)-C(25)-C(26)	120.5(3)
C(27)-C(26)-C(25)	119.2(3)
C(28)-C(27)-C(26)	120.2(3)
C(27)-C(28)-C(23)	121.4(3)
C(36)-C(29)-C(14)	114.4(2)
C(36)-C(29)-C(30)	111.1(2)
C(14)-C(29)-C(30)	111.4(2)
C(31)-C(30)-C(35)	118.0(3)
C(31)-C(30)-C(29)	123.5(3)
C(35)-C(30)-C(29)	118.6(3)
C(31)-C(30)-K(2)	77.18(16)
C(35)-C(30)-K(2)	81.37(16)
C(29)-C(30)-K(2)	111.25(16)
C(32)-C(31)-C(30)	121.0(3)
C(32)-C(31)-K(2)	80.56(17)
C(30)-C(31)-K(2)	78.82(16)
C(31)-C(32)-C(33)	120.2(3)
C(31)-C(32)-K(2)	75.73(17)
C(33)-C(32)-K(2)	81.50(17)
C(34)-C(33)-C(32)	119.5(3)
C(34)-C(33)-K(2)	79.52(17)
C(32)-C(33)-K(2)	75.18(17)
C(33)-C(34)-C(35)	120.2(3)
C(33)-C(34)-K(2)	77.69(17)
C(35)-C(34)-K(2)	76.05(16)
C(34)-C(35)-C(30)	121.1(3)
C(34)-C(35)-K(2)	80.89(17)
C(30)-C(35)-K(2)	74.93(16)
C(41)-C(36)-C(37)	118.1(3)
C(41)-C(36)-C(29)	120.3(3)
C(37)-C(36)-C(29)	121.6(3)
C(38)-C(37)-C(36)	120.6(3)
C(39)-C(38)-C(37)	120.8(3)
C(38)-C(39)-C(40)	119.2(3)
C(41)-C(40)-C(39)	119.9(3)
C(36)-C(41)-C(40)	121.4(3)
C(47)-C(42)-N(3)	109.9(2)
C(47)-C(42)-C(43)	120.6(3)
N(3)-C(42)-C(43)	129.6(3)
C(44)-C(43)-C(42)	115.6(3)
C(44)-C(43)-C(57)	125.5(3)
C(42)-C(43)-C(57)	118.9(2)
C(45)-C(44)-C(43)	122.5(3)
C(44)-C(45)-C(46)	121.8(3)
C(47)-C(46)-C(45)	115.0(3)
C(46)-C(47)-C(42)	124.4(3)
C(46)-C(47)-O(3)	127.8(3)
C(42)-C(47)-O(3)	107.7(2)
N(3)-C(48)-O(3)	113.6(2)
N(3)-C(48)-C(49)	125.3(3)
O(3)-C(48)-C(49)	121.0(2)
N(3)-C(48)-K(1)	60.80(14)
O(3)-C(48)-K(1)	131.33(17)
C(49)-C(48)-K(1)	80.89(16)
H(49)-C(49)-C(48)	116.2(15)
H(49)-C(49)-C(50)	114.2(15)
C(48)-C(49)-C(50)	128.8(3)
H(49)-C(49)-K(1)	60.2(15)
C(48)-C(49)-K(1)	74.13(16)
C(50)-C(49)-K(1)	142.74(19)
H(49)-C(49)-K(2)	39.9(15)
C(48)-C(49)-K(2)	150.13(19)
C(50)-C(49)-K(2)	79.68(17)
K(1)-C(49)-K(2)	76.80(7)
N(4)-C(50)-O(4)	114.6(2)

N(4)-C(50)-C(49)	125.6(3)	C(63)-C(62)-C(61)	120.1(3)
O(4)-C(50)-C(49)	119.8(2)	C(63)-C(62)-K(1)	83.54(17)
N(4)-C(50)-K(2)	49.16(14)	C(61)-C(62)-K(1)	72.87(17)
O(4)-C(50)-K(2)	162.15(18)	C(58)-C(63)-C(62)	121.1(3)
C(49)-C(50)-K(2)	77.10(17)	C(58)-C(63)-K(1)	79.48(17)
C(56)-C(51)-C(52)	120.3(2)	C(62)-C(63)-K(1)	72.52(17)
C(56)-C(51)-N(4)	109.3(2)	C(69)-C(64)-C(65)	117.9(3)
C(52)-C(51)-N(4)	130.5(3)	C(69)-C(64)-C(57)	119.8(3)
C(53)-C(52)-C(51)	115.3(3)	C(65)-C(64)-C(57)	122.2(3)
C(53)-C(52)-C(70)	123.1(2)	C(66)-C(65)-C(64)	120.9(3)
C(51)-C(52)-C(70)	121.5(2)	C(67)-C(66)-C(65)	120.4(4)
C(52)-C(53)-C(54)	123.0(3)	C(66)-C(67)-C(68)	119.8(3)
C(55)-C(54)-C(53)	121.6(3)	C(67)-C(68)-C(69)	119.8(4)
C(56)-C(55)-C(54)	114.9(3)	C(64)-C(69)-C(68)	121.2(4)
C(55)-C(56)-C(51)	125.0(3)	C(52)-C(70)-C(71)	114.5(2)
C(55)-C(56)-O(4)	127.2(2)	C(52)-C(70)-C(77)	113.1(2)
C(51)-C(56)-O(4)	107.9(2)	C(71)-C(70)-C(77)	109.1(2)
C(58)-C(57)-C(43)	112.4(2)	C(76)-C(71)-C(72)	118.5(3)
C(58)-C(57)-C(64)	112.8(2)	C(76)-C(71)-C(70)	119.4(3)
C(43)-C(57)-C(64)	112.1(2)	C(72)-C(71)-C(70)	122.0(2)
C(63)-C(58)-C(59)	118.0(3)	C(76)-C(71)-K(2)	71.00(16)
C(63)-C(58)-C(57)	120.8(3)	C(72)-C(71)-K(2)	85.55(16)
C(59)-C(58)-C(57)	121.2(3)	C(70)-C(71)-K(2)	116.04(17)
C(63)-C(58)-K(1)	77.09(16)	C(73)-C(72)-C(71)	120.5(3)
C(59)-C(58)-K(1)	71.36(16)	C(74)-C(73)-C(72)	120.4(3)
C(57)-C(58)-K(1)	123.07(17)	C(75)-C(74)-C(73)	119.5(3)
C(60)-C(59)-C(58)	120.7(3)	C(75)-C(74)-K(2)	70.96(18)
C(60)-C(59)-K(1)	72.23(17)	C(73)-C(74)-K(2)	84.45(18)
C(58)-C(59)-K(1)	84.66(17)	C(74)-C(75)-C(76)	120.0(3)
C(59)-C(60)-C(61)	121.2(3)	C(74)-C(75)-K(2)	85.19(18)
C(59)-C(60)-K(1)	83.05(17)	C(76)-C(75)-K(2)	76.44(17)
C(61)-C(60)-K(1)	78.04(18)	C(71)-C(76)-C(75)	121.0(3)
C(60)-C(61)-C(62)	118.9(3)	C(71)-C(76)-K(2)	84.82(17)
C(60)-C(61)-K(1)	76.64(18)	C(75)-C(76)-K(2)	78.63(17)
C(62)-C(61)-K(1)	82.14(18)	C(78)-C(77)-C(82)	118.7(3)

C(78)-C(77)-C(70)	121.1(2)	C(6B)-C(1B)-C(7B)	121.8(3)
C(82)-C(77)-C(70)	120.2(2)	C(3B)-C(2B)-C(1B)	121.1(3)
C(79)-C(78)-C(77)	121.1(3)	C(2B)-C(3B)-C(4B)	120.6(4)
C(78)-C(79)-C(80)	119.9(3)	C(3B)-C(4B)-C(5B)	119.5(4)
C(81)-C(80)-C(79)	119.5(3)	C(4B)-C(5B)-C(6B)	120.2(3)
C(80)-C(81)-C(82)	120.6(3)	C(5B)-C(6B)-C(1B)	120.7(3)
C(77)-C(82)-C(81)	120.2(3)	C(2C)-C(1C)-C(6C)	117.8(3)
C(2A)-C(1A)-C(6A)	118.1(3)	C(2C)-C(1C)-C(7C)	122.7(3)
C(2A)-C(1A)-C(7A)	120.6(3)	C(6C)-C(1C)-C(7C)	119.6(3)
C(6A)-C(1A)-C(7A)	121.3(3)	C(3C)-C(2C)-C(1C)	120.7(3)
C(1A)-C(2A)-C(3A)	121.6(3)	C(2C)-C(3C)-C(4C)	120.3(3)
C(4A)-C(3A)-C(2A)	119.9(4)	C(5C)-C(4C)-C(3C)	119.4(3)
C(3A)-C(4A)-C(5A)	119.5(4)	C(4C)-C(5C)-C(6C)	120.4(3)
C(4A)-C(5A)-C(6A)	120.4(4)	C(4C)-C(5C)-C(7D)	125.1(12)
C(1A)-C(6A)-C(5A)	120.6(4)	C(6C)-C(5C)-C(7D)	113.9(12)
C(2B)-C(1B)-C(6B)	117.9(3)	C(5C)-C(6C)-C(1C)	121.4(3)
C(2B)-C(1B)-C(7B)	120.4(3)		

XRAY: Compound 9

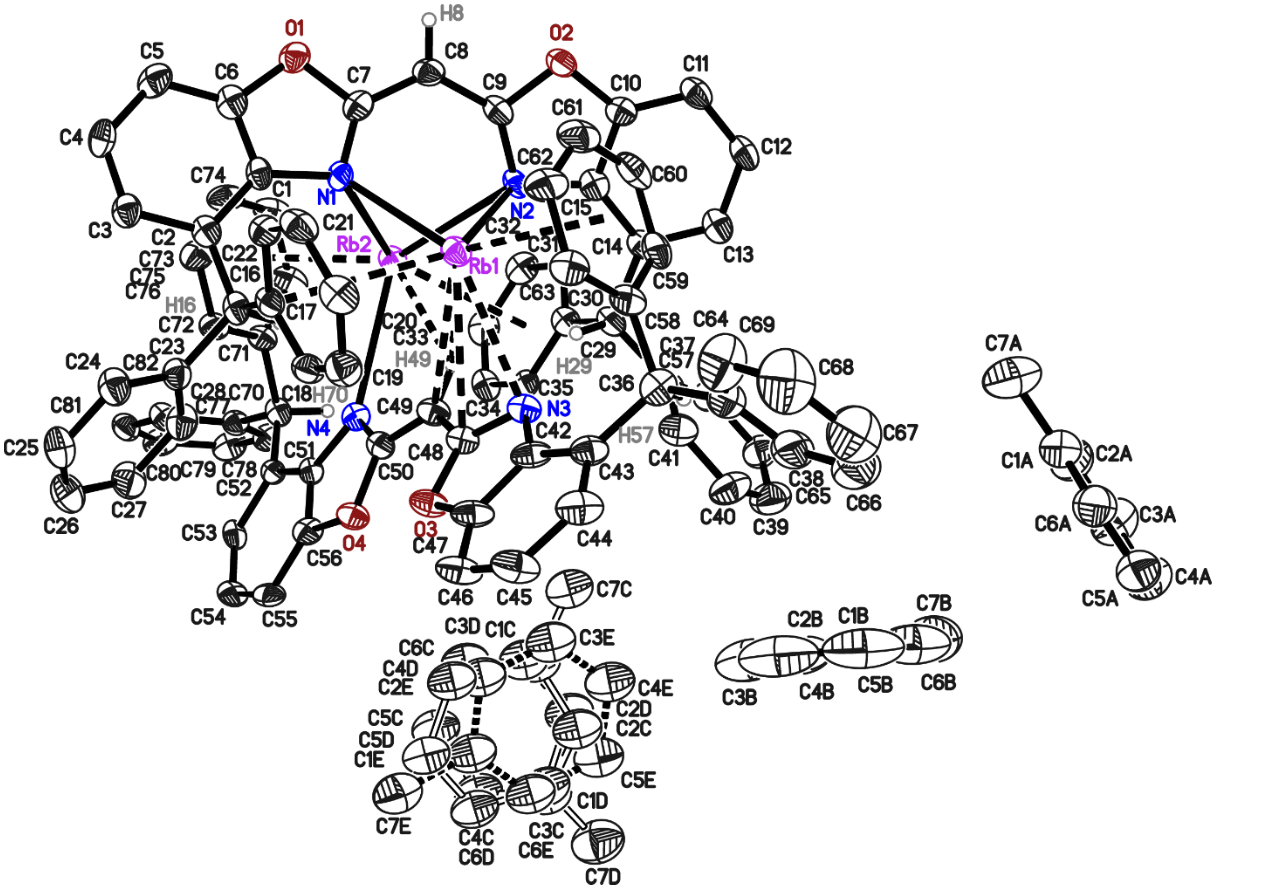

Figure S103. Asymmetric unit of 9. Displacement parameters are depicted at 50% probability. Hydrogen atoms except the hydrogen atoms bound to the methylene backbone (H8, H49) and benzylic groups (H16, H29, H57, H70) are omitted for clarity.

One of the three toluene molecules is disordered over three positions. The occupancies refined to 0.549(3), 0.351(3) and 0.100(3), respectively. All toluene molecules were refined with distance restraints and with restraints for the anisotropic displacement parameter.

The data were collected on a split crystal. An integration with two orientation matrices did not improve the model. Therefore, relatively high residual remained close to the Rb positions.

Table S6. Bond lengths [Å] and angles [°] for **9**.

Rb(1)-N(3)	2.905(5)	Rb(1)-C(62)	3.231(6)
Rb(1)-N(1)	2.924(5)	Rb(1)-C(61)	3.252(6)
Rb(1)-N(2)	3.026(5)	Rb(1)-C(48)	3.297(6)

Rb(1)-C(17)	3.318(6)	C(4)-C(5)	1.393(9)
Rb(1)-C(63)	3.343(6)	N(4)-C(50)	1.311(7)
Rb(1)-C(60)	3.359(6)	N(4)-C(51)	1.399(7)
Rb(1)-C(49)	3.394(6)	O(4)-C(50)	1.376(7)
Rb(1)-C(18)	3.407(6)	O(4)-C(56)	1.388(7)
Rb(1)-C(59)	3.477(6)	C(5)-C(6)	1.373(9)
Rb(2)-N(4)	2.948(5)	C(7)-C(8)	1.397(8)
Rb(2)-N(1)	3.040(5)	C(8)-C(9)	1.390(8)
Rb(2)-N(2)	3.063(4)	C(10)-C(11)	1.381(8)
Rb(2)-C(76)	3.280(6)	C(10)-C(15)	1.386(8)
Rb(2)-C(75)	3.311(7)	C(11)-C(12)	1.382(9)
Rb(2)-C(30)	3.391(6)	C(12)-C(13)	1.391(9)
Rb(2)-C(31)	3.400(6)	C(13)-C(14)	1.394(8)
Rb(2)-C(35)	3.424(6)	C(14)-C(15)	1.394(8)
Rb(2)-C(71)	3.432(6)	C(14)-C(29)	1.516(8)
Rb(2)-C(32)	3.447(6)	C(16)-C(17)	1.522(8)
Rb(2)-C(33)	3.469(6)	C(16)-C(23)	1.523(8)
Rb(2)-C(34)	3.470(6)	C(17)-C(18)	1.379(9)
C(1)-C(6)	1.379(9)	C(17)-C(22)	1.396(8)
C(1)-N(1)	1.404(7)	C(18)-C(19)	1.381(9)
C(1)-C(2)	1.409(8)	C(19)-C(20)	1.405(9)
N(1)-C(7)	1.328(8)	C(20)-C(21)	1.372(10)
O(1)-C(6)	1.381(7)	C(21)-C(22)	1.386(9)
O(1)-C(7)	1.400(7)	C(23)-C(28)	1.388(8)
C(2)-C(3)	1.400(8)	C(23)-C(24)	1.391(8)
C(2)-C(16)	1.508(9)	C(24)-C(25)	1.386(9)
N(2)-C(9)	1.332(8)	C(25)-C(26)	1.374(10)
N(2)-C(15)	1.406(7)	C(26)-C(27)	1.384(10)
O(2)-C(10)	1.374(7)	C(27)-C(28)	1.388(9)
O(2)-C(9)	1.398(7)	C(29)-C(36)	1.515(9)
C(3)-C(4)	1.384(9)	C(29)-C(30)	1.531(8)
N(3)-C(48)	1.348(8)	C(30)-C(31)	1.374(9)
N(3)-C(42)	1.383(8)	C(30)-C(35)	1.402(8)
O(3)-C(48)	1.380(7)	C(31)-C(32)	1.392(9)
O(3)-C(47)	1.394(7)	C(32)-C(33)	1.387(9)

C(33)-C(34)	1.366(9)	C(66)-C(67)	1.383(14)
C(34)-C(35)	1.381(8)	C(67)-C(68)	1.349(14)
C(36)-C(41)	1.368(9)	C(68)-C(69)	1.380(12)
C(36)-C(37)	1.406(8)	C(70)-C(77)	1.520(8)
C(37)-C(38)	1.389(9)	C(70)-C(71)	1.527(8)
C(38)-C(39)	1.370(11)	C(71)-C(76)	1.384(8)
C(39)-C(40)	1.390(11)	C(71)-C(72)	1.393(8)
C(40)-C(41)	1.384(10)	C(72)-C(73)	1.395(8)
C(42)-C(47)	1.387(9)	C(73)-C(74)	1.367(10)
C(42)-C(43)	1.406(9)	C(74)-C(75)	1.393(10)
C(43)-C(44)	1.390(9)	C(75)-C(76)	1.388(9)
C(43)-C(57)	1.539(9)	C(77)-C(82)	1.383(8)
C(44)-C(45)	1.392(10)	C(77)-C(78)	1.388(8)
C(45)-C(46)	1.392(9)	C(78)-C(79)	1.383(9)
C(46)-C(47)	1.369(9)	C(79)-C(80)	1.378(10)
C(48)-C(49)	1.379(8)	C(80)-C(81)	1.391(9)
C(49)-C(50)	1.410(8)	C(81)-C(82)	1.398(9)
C(51)-C(56)	1.378(8)	C(1A)-C(6A)	1.377(8)
C(51)-C(52)	1.412(8)	C(1A)-C(2A)	1.378(9)
C(52)-C(53)	1.402(8)	C(1A)-C(7A)	1.492(10)
C(52)-C(70)	1.508(8)	C(2A)-C(3A)	1.375(9)
C(53)-C(54)	1.377(9)	C(3A)-C(4A)	1.373(9)
C(54)-C(55)	1.390(8)	C(4A)-C(5A)	1.372(9)
C(55)-C(56)	1.374(8)	C(5A)-C(6A)	1.369(9)
C(57)-C(58)	1.508(9)	C(1B)-C(2B)	1.373(10)
C(57)-C(64)	1.521(9)	C(1B)-C(6B)	1.387(10)
C(58)-C(59)	1.369(9)	C(1B)-C(7B)	1.484(11)
C(58)-C(63)	1.405(9)	C(2B)-C(3B)	1.401(10)
C(59)-C(60)	1.383(10)	C(3B)-C(4B)	1.364(11)
C(60)-C(61)	1.401(10)	C(4B)-C(5B)	1.395(11)
C(61)-C(62)	1.375(10)	C(5B)-C(6B)	1.374(11)
C(62)-C(63)	1.381(9)	C(1C)-C(2C)	1.375(14)
C(64)-C(65)	1.364(10)	C(1C)-C(6C)	1.377(14)
C(64)-C(69)	1.398(10)	C(1C)-C(7C)	1.494(17)
C(65)-C(66)	1.391(11)	C(2C)-C(3C)	1.377(14)

C(3C)-C(4C)	1.378(14)
C(4C)-C(5C)	1.375(14)
C(5C)-C(6C)	1.376(14)
C(1D)-C(2D)	1.373(12)
C(1D)-C(6D)	1.374(12)
C(1D)-C(7D)	1.489(14)
C(2D)-C(3D)	1.374(12)
C(3D)-C(4D)	1.361(12)
C(4D)-C(5D)	1.375(12)
C(5D)-C(6D)	1.382(12)
C(1E)-C(2E)	1.372(13)
C(1E)-C(6E)	1.378(13)
C(1E)-C(7E)	1.502(16)
C(2E)-C(3E)	1.378(13)
C(3E)-C(4E)	1.382(13)
C(4E)-C(5E)	1.386(13)
C(5E)-C(6E)	1.382(13)
N(3)-Rb(1)-N(1)	133.04(13)
N(3)-Rb(1)-N(2)	102.97(13)
N(1)-Rb(1)-N(2)	65.15(13)
N(3)-Rb(1)-C(62)	97.28(16)
N(1)-Rb(1)-C(62)	129.45(15)
N(2)-Rb(1)-C(62)	114.54(16)
N(3)-Rb(1)-C(61)	106.75(16)
N(1)-Rb(1)-C(61)	118.01(16)
N(2)-Rb(1)-C(61)	90.22(16)
C(62)-Rb(1)-C(61)	24.48(18)
N(3)-Rb(1)-C(48)	24.04(14)
N(1)-Rb(1)-C(48)	110.44(14)
N(2)-Rb(1)-C(48)	102.26(13)
C(62)-Rb(1)-C(48)	118.12(16)
C(61)-Rb(1)-C(48)	130.67(17)
N(3)-Rb(1)-C(17)	97.85(14)
N(1)-Rb(1)-C(17)	67.48(14)

N(2)-Rb(1)-C(17)	130.13(14)
C(62)-Rb(1)-C(17)	106.92(16)
C(61)-Rb(1)-C(17)	126.02(17)
C(48)-Rb(1)-C(17)	80.28(14)
N(3)-Rb(1)-C(63)	73.41(14)
N(1)-Rb(1)-C(63)	152.42(14)
N(2)-Rb(1)-C(63)	123.22(14)
C(62)-Rb(1)-C(63)	24.17(16)
C(61)-Rb(1)-C(63)	42.65(17)
C(48)-Rb(1)-C(63)	93.98(15)
C(17)-Rb(1)-C(63)	105.99(16)
N(3)-Rb(1)-C(60)	91.09(16)
N(1)-Rb(1)-C(60)	124.88(16)
N(2)-Rb(1)-C(60)	75.32(15)
C(62)-Rb(1)-C(60)	42.46(18)
C(61)-Rb(1)-C(60)	24.41(18)
C(48)-Rb(1)-C(60)	114.39(17)
C(17)-Rb(1)-C(60)	149.19(16)
C(63)-Rb(1)-C(60)	48.70(17)
N(3)-Rb(1)-C(49)	44.32(14)
N(1)-Rb(1)-C(49)	88.72(14)
N(2)-Rb(1)-C(49)	84.64(13)
C(62)-Rb(1)-C(49)	141.31(16)
C(61)-Rb(1)-C(49)	147.51(17)
C(48)-Rb(1)-C(49)	23.72(14)
C(17)-Rb(1)-C(49)	79.60(14)
C(63)-Rb(1)-C(49)	117.16(15)
C(60)-Rb(1)-C(49)	124.99(17)
N(3)-Rb(1)-C(18)	76.39(14)
N(1)-Rb(1)-C(18)	89.56(14)
N(2)-Rb(1)-C(18)	145.26(14)
C(62)-Rb(1)-C(18)	99.82(17)
C(61)-Rb(1)-C(18)	123.67(17)
C(48)-Rb(1)-C(18)	63.13(14)
C(17)-Rb(1)-C(18)	23.61(15)

C(63)-Rb(1)-C(18)	90.36(16)	N(4)-Rb(2)-C(35)	62.76(13)
C(60)-Rb(1)-C(18)	139.00(17)	N(1)-Rb(2)-C(35)	133.85(14)
C(49)-Rb(1)-C(18)	70.70(14)	N(2)-Rb(2)-C(35)	83.54(13)
N(3)-Rb(1)-C(59)	68.02(15)	C(76)-Rb(2)-C(35)	101.41(15)
N(1)-Rb(1)-C(59)	144.95(14)	C(75)-Rb(2)-C(35)	123.18(16)
N(2)-Rb(1)-C(59)	84.23(14)	C(30)-Rb(2)-C(35)	23.74(14)
C(62)-Rb(1)-C(59)	48.22(16)	C(31)-Rb(2)-C(35)	40.78(15)
C(61)-Rb(1)-C(59)	41.77(17)	N(4)-Rb(2)-C(71)	59.13(14)
C(48)-Rb(1)-C(59)	91.69(15)	N(1)-Rb(2)-C(71)	116.42(13)
C(17)-Rb(1)-C(59)	145.59(15)	N(2)-Rb(2)-C(71)	179.24(14)
C(63)-Rb(1)-C(59)	40.80(15)	C(76)-Rb(2)-C(71)	23.66(14)
C(60)-Rb(1)-C(59)	23.26(16)	C(75)-Rb(2)-C(71)	41.89(15)
C(49)-Rb(1)-C(59)	105.74(15)	C(30)-Rb(2)-C(71)	118.48(14)
C(18)-Rb(1)-C(59)	125.20(15)	C(31)-Rb(2)-C(71)	117.47(13)
N(4)-Rb(2)-N(1)	107.12(13)	C(35)-Rb(2)-C(71)	97.06(14)
N(4)-Rb(2)-N(2)	121.60(14)	N(4)-Rb(2)-C(32)	101.69(14)
N(1)-Rb(2)-N(2)	63.34(12)	N(1)-Rb(2)-C(32)	144.36(14)
N(4)-Rb(2)-C(76)	80.87(14)	N(2)-Rb(2)-C(32)	83.88(14)
N(1)-Rb(2)-C(76)	122.18(15)	C(76)-Rb(2)-C(32)	82.43(16)
N(2)-Rb(2)-C(76)	155.76(15)	C(75)-Rb(2)-C(32)	91.82(17)
N(4)-Rb(2)-C(75)	100.85(15)	C(30)-Rb(2)-C(32)	41.33(15)
N(1)-Rb(2)-C(75)	102.71(15)	C(31)-Rb(2)-C(32)	23.46(15)
N(2)-Rb(2)-C(75)	137.36(15)	C(35)-Rb(2)-C(32)	47.31(16)
C(76)-Rb(2)-C(75)	24.32(16)	C(71)-Rb(2)-C(32)	96.18(14)
N(4)-Rb(2)-C(30)	84.47(14)	N(4)-Rb(2)-C(33)	81.06(15)
N(1)-Rb(2)-C(30)	121.21(13)	N(1)-Rb(2)-C(33)	166.75(14)
N(2)-Rb(2)-C(30)	62.03(13)	N(2)-Rb(2)-C(33)	103.57(14)
C(76)-Rb(2)-C(30)	116.51(15)	C(76)-Rb(2)-C(33)	68.73(16)
C(75)-Rb(2)-C(30)	132.19(16)	C(75)-Rb(2)-C(33)	85.52(17)
N(4)-Rb(2)-C(31)	103.37(14)	C(30)-Rb(2)-C(33)	48.00(15)
N(1)-Rb(2)-C(31)	125.94(13)	C(31)-Rb(2)-C(33)	40.88(14)
N(2)-Rb(2)-C(31)	62.73(13)	C(35)-Rb(2)-C(33)	40.44(16)
C(76)-Rb(2)-C(31)	105.82(15)	C(71)-Rb(2)-C(33)	76.64(14)
C(75)-Rb(2)-C(31)	114.03(16)	C(32)-Rb(2)-C(33)	23.13(15)
C(30)-Rb(2)-C(31)	23.35(15)	N(4)-Rb(2)-C(34)	61.40(14)

N(1)-Rb(2)-C(34)	156.13(15)	C(3)-C(4)-C(5)	122.1(6)
N(2)-Rb(2)-C(34)	103.30(13)	C(50)-N(4)-C(51)	104.5(5)
C(76)-Rb(2)-C(34)	78.49(16)	C(50)-N(4)-Rb(2)	111.1(3)
C(75)-Rb(2)-C(34)	100.15(16)	C(51)-N(4)-Rb(2)	143.0(4)
C(30)-Rb(2)-C(34)	41.30(14)	C(50)-O(4)-C(56)	104.3(4)
C(31)-Rb(2)-C(34)	47.23(14)	C(6)-C(5)-C(4)	114.6(6)
C(35)-Rb(2)-C(34)	23.09(14)	C(5)-C(6)-C(1)	125.3(6)
C(71)-Rb(2)-C(34)	77.19(14)	C(5)-C(6)-O(1)	127.1(6)
C(32)-Rb(2)-C(34)	40.31(16)	C(1)-C(6)-O(1)	107.6(5)
C(33)-Rb(2)-C(34)	22.70(15)	N(1)-C(7)-C(8)	131.3(6)
C(6)-C(1)-N(1)	110.1(5)	N(1)-C(7)-O(1)	113.4(5)
C(6)-C(1)-C(2)	119.8(5)	C(8)-C(7)-O(1)	115.2(5)
N(1)-C(1)-C(2)	130.0(6)	N(1)-C(7)-Rb(2)	59.6(3)
C(7)-N(1)-C(1)	104.1(5)	C(8)-C(7)-Rb(2)	94.6(3)
C(7)-N(1)-Rb(1)	114.9(4)	O(1)-C(7)-Rb(2)	123.2(3)
C(1)-N(1)-Rb(1)	128.3(4)	N(1)-C(7)-Rb(1)	46.0(3)
C(7)-N(1)-Rb(2)	98.3(3)	C(8)-C(7)-Rb(1)	89.7(4)
C(1)-N(1)-Rb(2)	113.6(3)	O(1)-C(7)-Rb(1)	145.5(3)
Rb(1)-N(1)-Rb(2)	93.63(13)	Rb(2)-C(7)-Rb(1)	74.60(11)
C(6)-O(1)-C(7)	104.7(5)	C(9)-C(8)-C(7)	125.6(6)
C(3)-C(2)-C(1)	115.6(6)	N(2)-C(9)-C(8)	132.5(5)
C(3)-C(2)-C(16)	124.0(5)	N(2)-C(9)-O(2)	112.7(5)
C(1)-C(2)-C(16)	120.2(5)	C(8)-C(9)-O(2)	114.7(5)
C(9)-N(2)-C(15)	105.0(5)	N(2)-C(9)-Rb(1)	56.3(3)
C(9)-N(2)-Rb(1)	102.2(3)	C(8)-C(9)-Rb(1)	95.3(4)
C(15)-N(2)-Rb(1)	115.2(4)	O(2)-C(9)-Rb(1)	126.6(4)
C(9)-N(2)-Rb(2)	108.1(4)	N(2)-C(9)-Rb(2)	51.9(3)
C(15)-N(2)-Rb(2)	131.5(3)	C(8)-C(9)-Rb(2)	85.9(3)
Rb(1)-N(2)-Rb(2)	91.18(12)	O(2)-C(9)-Rb(2)	146.3(3)
C(10)-O(2)-C(9)	105.0(4)	Rb(1)-C(9)-Rb(2)	73.65(11)
C(4)-C(3)-C(2)	122.5(6)	O(2)-C(10)-C(11)	128.1(5)
C(48)-N(3)-C(42)	104.3(5)	O(2)-C(10)-C(15)	108.3(5)
C(48)-N(3)-Rb(1)	94.5(4)	C(11)-C(10)-C(15)	123.6(6)
C(42)-N(3)-Rb(1)	119.5(3)	C(10)-C(11)-C(12)	116.0(6)
C(48)-O(3)-C(47)	105.1(4)	C(11)-C(12)-C(13)	121.1(6)

C(12)-C(13)-C(14)	122.8(6)	C(36)-C(29)-C(14)	114.7(5)
C(15)-C(14)-C(13)	115.7(5)	C(36)-C(29)-C(30)	111.6(5)
C(15)-C(14)-C(29)	120.4(5)	C(14)-C(29)-C(30)	111.4(5)
C(13)-C(14)-C(29)	123.7(5)	C(31)-C(30)-C(35)	117.8(6)
C(10)-C(15)-C(14)	120.5(5)	C(31)-C(30)-C(29)	123.9(5)
C(10)-C(15)-N(2)	108.9(5)	C(35)-C(30)-C(29)	118.3(5)
C(14)-C(15)-N(2)	130.5(5)	C(31)-C(30)-Rb(2)	78.7(4)
C(2)-C(16)-C(17)	112.9(5)	C(35)-C(30)-Rb(2)	79.4(3)
C(2)-C(16)-C(23)	113.1(5)	C(29)-C(30)-Rb(2)	111.7(3)
C(17)-C(16)-C(23)	111.8(5)	C(30)-C(31)-C(32)	121.5(6)
C(18)-C(17)-C(22)	117.6(6)	C(30)-C(31)-Rb(2)	78.0(3)
C(18)-C(17)-C(16)	119.4(5)	C(32)-C(31)-Rb(2)	80.2(4)
C(22)-C(17)-C(16)	123.0(6)	C(33)-C(32)-C(31)	119.4(6)
C(18)-C(17)-Rb(1)	81.8(3)	C(33)-C(32)-Rb(2)	79.3(4)
C(22)-C(17)-Rb(1)	86.9(3)	C(31)-C(32)-Rb(2)	76.4(3)
C(16)-C(17)-Rb(1)	100.9(3)	C(34)-C(33)-C(32)	120.0(6)
C(17)-C(18)-C(19)	122.4(6)	C(34)-C(33)-Rb(2)	78.7(3)
C(17)-C(18)-Rb(1)	74.6(3)	C(32)-C(33)-Rb(2)	77.5(3)
C(19)-C(18)-Rb(1)	91.1(4)	C(33)-C(34)-C(35)	120.4(6)
C(18)-C(19)-C(20)	119.2(6)	C(33)-C(34)-Rb(2)	78.6(3)
C(18)-C(19)-Rb(1)	67.0(4)	C(35)-C(34)-Rb(2)	76.6(3)
C(20)-C(19)-Rb(1)	87.4(4)	C(34)-C(35)-C(30)	120.9(6)
C(21)-C(20)-C(19)	118.9(6)	C(34)-C(35)-Rb(2)	80.3(3)
C(20)-C(21)-C(22)	121.1(6)	C(30)-C(35)-Rb(2)	76.8(3)
C(21)-C(22)-C(17)	120.7(6)	C(41)-C(36)-C(37)	118.6(6)
C(21)-C(22)-Rb(1)	90.5(4)	C(41)-C(36)-C(29)	120.5(6)
C(17)-C(22)-Rb(1)	69.8(3)	C(37)-C(36)-C(29)	120.8(6)
C(28)-C(23)-C(24)	118.5(6)	C(38)-C(37)-C(36)	120.3(6)
C(28)-C(23)-C(16)	119.6(5)	C(39)-C(38)-C(37)	120.2(7)
C(24)-C(23)-C(16)	121.9(5)	C(38)-C(39)-C(40)	119.8(7)
C(25)-C(24)-C(23)	120.4(6)	C(41)-C(40)-C(39)	119.9(7)
C(26)-C(25)-C(24)	120.4(6)	C(36)-C(41)-C(40)	121.2(7)
C(25)-C(26)-C(27)	120.0(6)	N(3)-C(42)-C(47)	110.5(5)
C(26)-C(27)-C(28)	119.6(6)	N(3)-C(42)-C(43)	130.1(6)
C(27)-C(28)-C(23)	121.0(6)	C(47)-C(42)-C(43)	119.5(6)

C(44)-C(43)-C(42)	116.1(6)	C(56)-C(55)-C(54)	114.7(6)
C(44)-C(43)-C(57)	125.3(6)	C(55)-C(56)-C(51)	125.3(5)
C(42)-C(43)-C(57)	118.6(5)	C(55)-C(56)-O(4)	127.2(5)
C(43)-C(44)-C(45)	122.1(6)	C(51)-C(56)-O(4)	107.5(5)
C(44)-C(45)-C(46)	122.5(6)	C(58)-C(57)-C(64)	113.0(5)
C(47)-C(46)-C(45)	114.1(6)	C(58)-C(57)-C(43)	113.3(5)
C(46)-C(47)-C(42)	125.6(6)	C(64)-C(57)-C(43)	111.2(5)
C(46)-C(47)-O(3)	127.4(6)	C(59)-C(58)-C(63)	118.1(6)
C(42)-C(47)-O(3)	106.9(5)	C(59)-C(58)-C(57)	120.6(6)
N(3)-C(48)-C(49)	125.0(5)	C(63)-C(58)-C(57)	121.2(6)
N(3)-C(48)-O(3)	113.2(5)	C(59)-C(58)-Rb(1)	77.6(4)
C(49)-C(48)-O(3)	121.7(5)	C(63)-C(58)-Rb(1)	71.8(4)
N(3)-C(48)-Rb(1)	61.4(3)	C(57)-C(58)-Rb(1)	122.3(4)
C(49)-C(48)-Rb(1)	82.1(3)	C(58)-C(59)-C(60)	121.9(6)
O(3)-C(48)-Rb(1)	128.6(3)	C(58)-C(59)-Rb(1)	79.8(4)
C(48)-C(49)-C(50)	128.5(6)	C(60)-C(59)-Rb(1)	73.6(4)
C(48)-C(49)-Rb(1)	74.2(3)	C(59)-C(60)-C(61)	119.8(6)
C(50)-C(49)-Rb(1)	140.6(4)	C(59)-C(60)-Rb(1)	83.2(4)
C(48)-C(49)-Rb(2)	150.1(4)	C(61)-C(60)-Rb(1)	73.5(4)
C(50)-C(49)-Rb(2)	79.4(4)	C(62)-C(61)-C(60)	118.7(7)
Rb(1)-C(49)-Rb(2)	76.61(12)	C(62)-C(61)-Rb(1)	76.9(4)
N(4)-C(50)-O(4)	114.3(5)	C(60)-C(61)-Rb(1)	82.0(4)
N(4)-C(50)-C(49)	126.3(5)	C(61)-C(62)-C(63)	121.1(6)
O(4)-C(50)-C(49)	119.3(5)	C(61)-C(62)-Rb(1)	78.6(4)
N(4)-C(50)-Rb(2)	49.2(3)	C(63)-C(62)-Rb(1)	82.4(4)
O(4)-C(50)-Rb(2)	160.8(4)	C(62)-C(63)-C(58)	120.3(6)
C(49)-C(50)-Rb(2)	78.2(3)	C(62)-C(63)-Rb(1)	73.4(4)
C(56)-C(51)-N(4)	109.3(5)	C(58)-C(63)-Rb(1)	84.6(4)
C(56)-C(51)-C(52)	120.0(5)	C(65)-C(64)-C(69)	117.9(7)
N(4)-C(51)-C(52)	130.6(5)	C(65)-C(64)-C(57)	120.3(6)
C(53)-C(52)-C(51)	114.8(5)	C(69)-C(64)-C(57)	121.7(7)
C(53)-C(52)-C(70)	123.4(5)	C(64)-C(65)-C(66)	121.5(8)
C(51)-C(52)-C(70)	121.8(5)	C(67)-C(66)-C(65)	120.2(9)
C(54)-C(53)-C(52)	123.4(5)	C(68)-C(67)-C(66)	117.9(9)
C(53)-C(54)-C(55)	121.8(5)	C(67)-C(68)-C(69)	122.9(9)

C(68)-C(69)-C(64)	119.4(9)	C(2A)-C(1A)-C(7A)	119.5(7)
C(52)-C(70)-C(77)	114.3(5)	C(3A)-C(2A)-C(1A)	120.8(7)
C(52)-C(70)-C(71)	115.0(5)	C(4A)-C(3A)-C(2A)	120.5(7)
C(77)-C(70)-C(71)	108.8(5)	C(5A)-C(4A)-C(3A)	118.7(7)
C(76)-C(71)-C(72)	118.7(5)	C(6A)-C(5A)-C(4A)	121.0(7)
C(76)-C(71)-C(70)	119.4(5)	C(5A)-C(6A)-C(1A)	120.7(7)
C(72)-C(71)-C(70)	121.6(5)	C(2B)-C(1B)-C(6B)	118.8(9)
C(76)-C(71)-Rb(2)	72.0(3)	C(2B)-C(1B)-C(7B)	122.2(9)
C(72)-C(71)-Rb(2)	85.7(3)	C(6B)-C(1B)-C(7B)	119.0(9)
C(70)-C(71)-Rb(2)	116.6(4)	C(1B)-C(2B)-C(3B)	121.8(9)
C(71)-C(72)-C(73)	120.0(6)	C(4B)-C(3B)-C(2B)	119.5(10)
C(71)-C(72)-Rb(2)	71.7(3)	C(3B)-C(4B)-C(5B)	118.5(10)
C(73)-C(72)-Rb(2)	78.9(4)	C(6B)-C(5B)-C(4B)	122.2(10)
C(74)-C(73)-C(72)	120.8(6)	C(5B)-C(6B)-C(1B)	119.2(10)
C(74)-C(73)-Rb(2)	73.7(4)	C(2C)-C(1C)-C(6C)	119.7(16)
C(72)-C(73)-Rb(2)	78.8(4)	C(2C)-C(1C)-C(7C)	120.6(19)
C(73)-C(74)-C(75)	119.5(6)	C(6C)-C(1C)-C(7C)	119.7(19)
C(73)-C(74)-Rb(2)	84.1(4)	C(1C)-C(2C)-C(3C)	120.5(18)
C(75)-C(74)-Rb(2)	71.5(4)	C(2C)-C(3C)-C(4C)	119.7(18)
C(76)-C(75)-C(74)	119.8(6)	C(5C)-C(4C)-C(3C)	119.3(18)
C(76)-C(75)-Rb(2)	76.6(4)	C(4C)-C(5C)-C(6C)	120.5(18)
C(74)-C(75)-Rb(2)	85.0(4)	C(5C)-C(6C)-C(1C)	119.7(18)
C(71)-C(76)-C(75)	121.0(6)	C(2D)-C(1D)-C(6D)	119.7(11)
C(71)-C(76)-Rb(2)	84.3(4)	C(2D)-C(1D)-C(7D)	119.3(12)
C(75)-C(76)-Rb(2)	79.1(4)	C(6D)-C(1D)-C(7D)	120.9(12)
C(82)-C(77)-C(78)	118.5(6)	C(1D)-C(2D)-C(3D)	121.2(12)
C(82)-C(77)-C(70)	120.4(5)	C(4D)-C(3D)-C(2D)	118.9(12)
C(78)-C(77)-C(70)	121.1(5)	C(3D)-C(4D)-C(5D)	120.8(12)
C(79)-C(78)-C(77)	120.6(6)	C(4D)-C(5D)-C(6D)	120.1(12)
C(80)-C(79)-C(78)	120.8(6)	C(1D)-C(6D)-C(5D)	119.2(11)
C(79)-C(80)-C(81)	119.6(6)	C(2E)-C(1E)-C(6E)	121.3(14)
C(80)-C(81)-C(82)	119.2(6)	C(2E)-C(1E)-C(7E)	118.0(14)
C(77)-C(82)-C(81)	121.3(5)	C(6E)-C(1E)-C(7E)	120.3(14)
C(6A)-C(1A)-C(2A)	118.3(7)	C(1E)-C(2E)-C(3E)	119.4(15)
C(6A)-C(1A)-C(7A)	122.2(7)	C(2E)-C(3E)-C(4E)	120.7(15)

C(3E)-C(4E)-C(5E)	118.9(15)	C(1E)-C(6E)-C(5E)	118.8(15)
C(6E)-C(5E)-C(4E)	120.9(15)		

XRAY: Compound 10

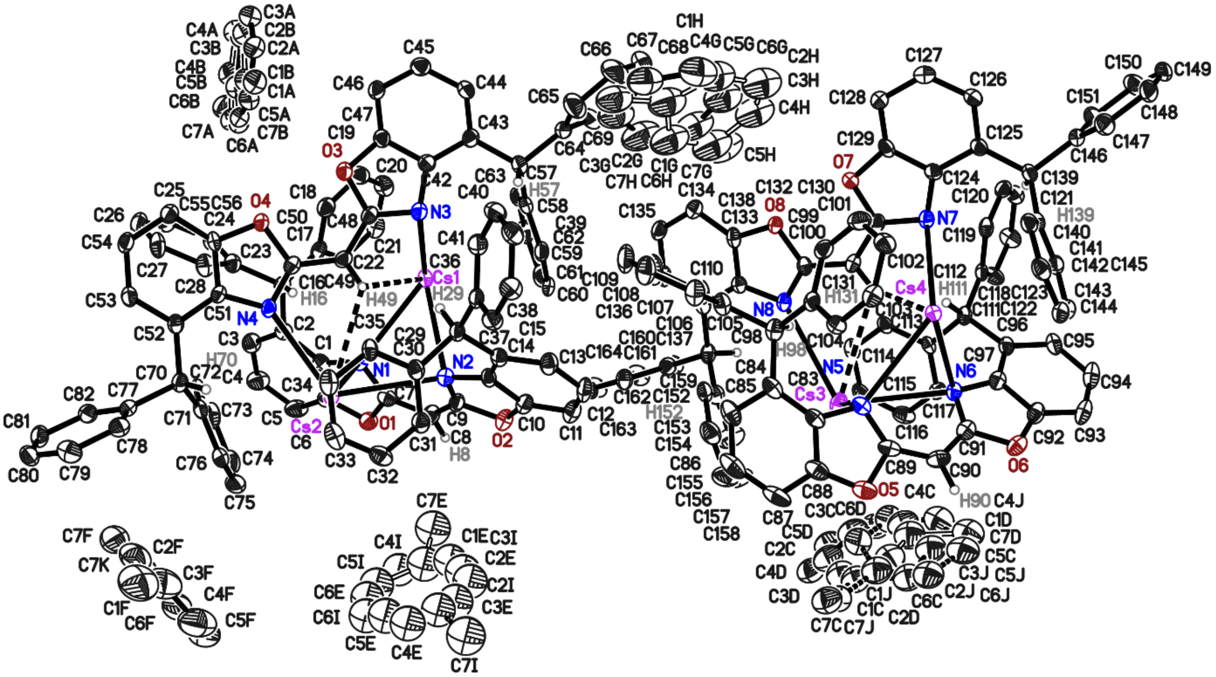

Figure S104. Asymmetric unit of 10. Displacement parameters are depicted at 50% probability. Hydrogen atoms except the hydrogen atoms bound to the methylene backbone (H8, H49, H90, H131) and benzylic groups (H16, H29, H57, H70, H98, H111, H139, H152) are omitted for clarity.

All toluene molecule are disordered over two or even three positions. The occupancies refined to 0.733(5) / 0.267(5) (molecule A / B), 0.347(3) / 0.345(3) / 0.309(3) (molecule C / D / J), 0.190(6) / 0.310(6) (molecule I / E with additional disorder over an inversion center), and 0.747(8) / 0.253(8) (molecule F / K). All toluene molecules were refined with distance restraints and restraints for the anisotropic displacement parameters.

The positions of the hydrogen atoms H49 and H131 were refined without any constraint or restraint.

Table S7. Bond lengths [Å] and angles [°] for **10**.

Cs(1)-N(3)	3.033(3)	Cs(1)-C(17)	3.447(4)
Cs(1)-N(1)	3.050(3)	Cs(1)-C(60)	3.457(4)
Cs(1)-N(2)	3.189(3)	Cs(1)-C(63)	3.491(4)
Cs(1)-H(49)	3.34(4)	Cs(1)-C(18)	3.517(4)
Cs(1)-C(61)	3.418(4)	Cs(1)-C(59)	3.533(4)
Cs(1)-C(62)	3.427(4)	O(1)-C(6)	1.388(5)
Cs(1)-C(48)	3.436(4)	O(1)-C(7)	1.399(5)

N(1)-C(7)	1.327(5)	O(3)-C(48)	1.387(5)
N(1)-C(1)	1.406(5)	O(3)-C(47)	1.391(5)
N(1)-Cs(2)	3.124(3)	N(3)-C(48)	1.339(5)
C(1)-C(6)	1.379(6)	N(3)-C(42)	1.388(5)
C(1)-C(2)	1.394(5)	C(3)-C(4)	1.398(6)
C(1)-Cs(2)	3.885(4)	Cs(4)-N(7)	3.045(3)
O(2)-C(10)	1.374(5)	Cs(4)-N(5)	3.063(3)
O(2)-C(9)	1.403(5)	Cs(4)-N(6)	3.154(3)
Cs(2)-H(49)	3.10(4)	Cs(4)-H(131)	3.23(4)
Cs(2)-N(4)	3.107(3)	Cs(4)-C(143)	3.418(4)
Cs(2)-N(2)	3.186(3)	Cs(4)-C(130)	3.441(4)
Cs(2)-C(75)	3.496(5)	Cs(4)-C(144)	3.447(4)
Cs(2)-C(76)	3.497(4)	Cs(4)-C(99)	3.455(4)
Cs(2)-C(31)	3.532(4)	Cs(4)-C(142)	3.457(4)
Cs(2)-C(30)	3.534(4)	Cs(4)-C(100)	3.465(4)
Cs(2)-C(35)	3.551(4)	Cs(4)-C(141)	3.525(4)
Cs(2)-C(32)	3.552(4)	Cs(4)-C(145)	3.535(4)
Cs(2)-C(74)	3.563(5)	O(4)-C(56)	1.385(4)
Cs(2)-C(33)	3.567(4)	O(4)-C(50)	1.398(5)
N(2)-C(9)	1.334(5)	N(4)-C(50)	1.322(5)
N(2)-C(15)	1.410(5)	N(4)-C(51)	1.395(5)
C(2)-C(3)	1.398(6)	C(4)-C(5)	1.382(6)
C(2)-C(16)	1.523(5)	O(5)-C(88)	1.379(5)
Cs(3)-N(5)	3.165(3)	O(5)-C(89)	1.410(5)
Cs(3)-N(8)	3.202(3)	N(5)-C(89)	1.322(5)
Cs(3)-N(6)	3.285(3)	N(5)-C(83)	1.409(5)
Cs(3)-H(131)	3.33(4)	C(5)-C(6)	1.368(6)
Cs(3)-C(113)	3.530(4)	O(6)-C(92)	1.380(5)
Cs(3)-C(114)	3.554(4)	O(6)-C(91)	1.404(5)
Cs(3)-C(3J)	3.58(2)	N(6)-C(91)	1.329(5)
Cs(3)-C(89)	3.597(4)	N(6)-C(97)	1.398(5)
Cs(3)-C(154)	3.604(5)	O(7)-C(129)	1.388(5)
Cs(3)-C(153)	3.630(4)	O(7)-C(130)	1.397(5)
Cs(3)-C(112)	3.635(4)	N(7)-C(130)	1.329(5)
Cs(3)-C(155)	3.657(6)	N(7)-C(124)	1.388(5)

C(7)-C(8)	1.396(6)	C(36)-C(37)	1.387(5)
O(8)-C(133)	1.380(4)	C(36)-C(41)	1.394(6)
O(8)-C(132)	1.385(5)	C(37)-C(38)	1.376(6)
N(8)-C(132)	1.323(5)	C(38)-C(39)	1.381(6)
N(8)-C(138)	1.392(5)	C(39)-C(40)	1.386(6)
C(8)-C(9)	1.390(6)	C(40)-C(41)	1.393(6)
C(10)-C(11)	1.378(6)	C(42)-C(47)	1.388(5)
C(10)-C(15)	1.395(6)	C(42)-C(43)	1.402(5)
C(11)-C(12)	1.383(6)	C(43)-C(44)	1.389(6)
C(12)-C(13)	1.388(6)	C(43)-C(57)	1.525(5)
C(13)-C(14)	1.404(5)	C(44)-C(45)	1.391(6)
C(14)-C(15)	1.395(6)	C(45)-C(46)	1.394(6)
C(14)-C(29)	1.519(5)	C(46)-C(47)	1.378(6)
C(16)-C(23)	1.518(5)	C(48)-C(49)	1.398(6)
C(16)-C(17)	1.536(6)	C(49)-H(49)	0.89(4)
C(17)-C(22)	1.382(5)	C(49)-C(50)	1.402(5)
C(17)-C(18)	1.402(6)	C(51)-C(56)	1.393(5)
C(18)-C(19)	1.398(6)	C(51)-C(52)	1.399(5)
C(19)-C(20)	1.380(6)	C(52)-C(53)	1.400(5)
C(20)-C(21)	1.385(6)	C(52)-C(70)	1.530(5)
C(21)-C(22)	1.388(6)	C(53)-C(54)	1.396(6)
C(23)-C(28)	1.390(6)	C(54)-C(55)	1.393(6)
C(23)-C(24)	1.396(6)	C(55)-C(56)	1.369(5)
C(24)-C(25)	1.391(6)	C(57)-C(58)	1.519(6)
C(25)-C(26)	1.382(6)	C(57)-C(64)	1.524(6)
C(26)-C(27)	1.380(6)	C(58)-C(63)	1.389(6)
C(27)-C(28)	1.396(6)	C(58)-C(59)	1.397(6)
C(29)-C(36)	1.530(6)	C(59)-C(60)	1.391(6)
C(29)-C(30)	1.531(5)	C(60)-C(61)	1.378(6)
C(30)-C(31)	1.385(6)	C(61)-C(62)	1.377(6)
C(30)-C(35)	1.397(5)	C(62)-C(63)	1.389(6)
C(31)-C(32)	1.388(6)	C(64)-C(69)	1.379(6)
C(32)-C(33)	1.382(6)	C(64)-C(65)	1.394(7)
C(33)-C(34)	1.382(6)	C(65)-C(66)	1.401(7)
C(34)-C(35)	1.387(6)	C(66)-C(67)	1.369(8)

C(67)-C(68)	1.370(8)	C(99)-C(100)	1.399(5)
C(68)-C(69)	1.409(7)	C(100)-C(101)	1.380(6)
C(70)-C(77)	1.523(5)	C(101)-C(102)	1.381(6)
C(70)-C(71)	1.530(6)	C(102)-C(103)	1.378(6)
C(71)-C(72)	1.379(6)	C(103)-C(104)	1.393(5)
C(71)-C(76)	1.389(6)	C(105)-C(106)	1.386(6)
C(72)-C(73)	1.393(6)	C(105)-C(110)	1.389(6)
C(73)-C(74)	1.381(7)	C(106)-C(107)	1.388(6)
C(74)-C(75)	1.373(7)	C(107)-C(108)	1.385(7)
C(75)-C(76)	1.392(6)	C(108)-C(109)	1.381(7)
C(77)-C(78)	1.380(6)	C(109)-C(110)	1.387(6)
C(77)-C(82)	1.397(5)	C(111)-C(118)	1.524(5)
C(78)-C(79)	1.396(6)	C(111)-C(112)	1.527(5)
C(79)-C(80)	1.381(6)	C(112)-C(117)	1.389(5)
C(80)-C(81)	1.385(6)	C(112)-C(113)	1.391(5)
C(81)-C(82)	1.380(6)	C(113)-C(114)	1.381(5)
C(83)-C(88)	1.386(6)	C(114)-C(115)	1.382(6)
C(83)-C(84)	1.393(6)	C(115)-C(116)	1.385(6)
C(84)-C(85)	1.399(6)	C(116)-C(117)	1.391(6)
C(84)-C(98)	1.524(6)	C(118)-C(123)	1.388(5)
C(85)-C(86)	1.391(6)	C(118)-C(119)	1.390(5)
C(86)-C(87)	1.383(7)	C(119)-C(120)	1.383(6)
C(87)-C(88)	1.381(6)	C(120)-C(121)	1.380(6)
C(89)-C(90)	1.386(6)	C(121)-C(122)	1.385(6)
C(90)-C(91)	1.384(6)	C(122)-C(123)	1.383(6)
C(92)-C(93)	1.371(6)	C(124)-C(129)	1.389(5)
C(92)-C(97)	1.394(5)	C(124)-C(125)	1.403(5)
C(93)-C(94)	1.384(6)	C(125)-C(126)	1.396(6)
C(94)-C(95)	1.390(6)	C(125)-C(139)	1.516(5)
C(95)-C(96)	1.395(5)	C(126)-C(127)	1.397(6)
C(96)-C(97)	1.397(6)	C(127)-C(128)	1.396(6)
C(96)-C(111)	1.520(5)	C(128)-C(129)	1.370(6)
C(98)-C(105)	1.515(6)	C(130)-C(131)	1.390(5)
C(98)-C(99)	1.532(6)	C(131)-H(131)	0.90(4)
C(99)-C(104)	1.387(5)	C(131)-C(132)	1.404(5)

C(133)-C(134)	1.374(5)	C(1A)-C(6A)	1.392(9)
C(133)-C(138)	1.390(5)	C(1A)-C(2A)	1.395(8)
C(134)-C(135)	1.388(6)	C(1A)-C(7A)	1.503(8)
C(135)-C(136)	1.383(6)	C(2A)-C(3A)	1.380(8)
C(136)-C(137)	1.391(5)	C(3A)-C(4A)	1.383(8)
C(137)-C(138)	1.405(5)	C(4A)-C(5A)	1.386(7)
C(137)-C(152)	1.524(6)	C(5A)-C(6A)	1.371(8)
C(139)-C(146)	1.519(5)	C(1B)-C(6B)	1.387(12)
C(139)-C(140)	1.527(5)	C(1B)-C(2B)	1.390(12)
C(140)-C(145)	1.386(6)	C(1B)-C(7B)	1.531(13)
C(140)-C(141)	1.389(6)	C(2B)-C(3B)	1.380(13)
C(141)-C(142)	1.390(6)	C(3B)-C(4B)	1.372(13)
C(142)-C(143)	1.377(6)	C(4B)-C(5B)	1.399(13)
C(143)-C(144)	1.378(6)	C(5B)-C(6B)	1.392(13)
C(144)-C(145)	1.384(6)	C(1C)-C(2C)	1.380(12)
C(146)-C(147)	1.385(6)	C(1C)-C(6C)	1.401(12)
C(146)-C(151)	1.400(6)	C(1C)-C(7C)	1.484(13)
C(147)-C(148)	1.381(6)	C(2C)-C(3C)	1.394(12)
C(148)-C(149)	1.375(7)	C(3C)-C(4C)	1.402(12)
C(149)-C(150)	1.386(7)	C(4C)-C(5C)	1.411(12)
C(150)-C(151)	1.386(6)	C(5C)-C(6C)	1.380(12)
C(152)-C(159)	1.533(5)	C(1D)-C(2D)	1.387(12)
C(152)-C(153)	1.538(6)	C(1D)-C(6D)	1.401(12)
C(153)-C(158)	1.371(6)	C(1D)-C(7D)	1.602(13)
C(153)-C(154)	1.386(6)	C(2D)-C(3D)	1.385(12)
C(154)-C(155)	1.381(7)	C(3D)-C(4D)	1.392(12)
C(155)-C(156)	1.383(7)	C(4D)-C(5D)	1.396(12)
C(156)-C(157)	1.375(7)	C(5D)-C(6D)	1.403(12)
C(157)-C(158)	1.394(6)	C(1E)-C(6E)	1.389(12)
C(159)-C(160)	1.383(6)	C(1E)-C(2E)	1.412(13)
C(159)-C(164)	1.397(6)	C(1E)-C(7E)	1.566(14)
C(160)-C(161)	1.386(6)	C(2E)-C(3E)	1.400(13)
C(161)-C(162)	1.381(6)	C(3E)-C(4E)	1.351(13)
C(162)-C(163)	1.383(6)	C(4E)-C(5E)	1.440(13)
C(163)-C(164)	1.387(6)	C(5E)-C(6E)	1.420(13)

C(1F)-C(2F)	1.376(7)	C(5J)-C(6J)	1.413(12)
C(1F)-C(6F)	1.390(7)		
C(1F)-C(7F)	1.522(8)	N(3)-Cs(1)-N(1)	126.35(9)
C(2F)-C(3F)	1.377(7)	N(3)-Cs(1)-N(2)	96.16(9)
C(3F)-C(4F)	1.382(8)	N(1)-Cs(1)-N(2)	63.52(8)
C(3F)-C(7K)	1.548(13)	N(3)-Cs(1)-H(49)	47.2(7)
C(4F)-C(5F)	1.385(8)	N(1)-Cs(1)-H(49)	80.0(7)
C(5F)-C(6F)	1.364(7)	N(2)-Cs(1)-H(49)	66.1(7)
C(1G)-C(6G)	1.315(9)	N(3)-Cs(1)-C(61)	105.67(10)
C(1G)-C(2G)	1.414(9)	N(1)-Cs(1)-C(61)	122.19(10)
C(1G)-C(7G)	1.601(10)	N(2)-Cs(1)-C(61)	90.10(10)
C(2G)-C(3G)	1.398(9)	H(49)-Cs(1)-C(61)	137.0(7)
C(3G)-C(4G)	1.370(8)	N(3)-Cs(1)-C(62)	97.50(10)
C(4G)-C(5G)	1.363(9)	N(1)-Cs(1)-C(62)	135.94(10)
C(5G)-C(6G)	1.423(9)	N(2)-Cs(1)-C(62)	112.81(10)
C(1H)-C(6H)	1.388(13)	H(49)-Cs(1)-C(62)	141.7(7)
C(1H)-C(2H)	1.394(13)	C(61)-Cs(1)-C(62)	23.21(10)
C(1H)-C(7H)	1.538(14)	N(3)-Cs(1)-C(48)	22.81(9)
C(2H)-C(3H)	1.426(13)	N(1)-Cs(1)-C(48)	106.10(9)
C(3H)-C(4H)	1.373(13)	N(2)-Cs(1)-C(48)	96.43(9)
C(4H)-C(5H)	1.385(13)	H(49)-Cs(1)-C(48)	33.4(7)
C(5H)-C(6H)	1.380(13)	C(61)-Cs(1)-C(48)	128.42(10)
C(1I)-C(6I)	1.379(13)	C(62)-Cs(1)-C(48)	117.76(10)
C(1I)-C(2I)	1.388(13)	N(3)-Cs(1)-C(17)	98.69(9)
C(1I)-C(7I)	1.486(14)	N(1)-Cs(1)-C(17)	64.28(9)
C(2I)-C(3I)	1.399(14)	N(2)-Cs(1)-C(17)	124.01(9)
C(3I)-C(4I)	1.417(14)	H(49)-Cs(1)-C(17)	86.4(7)
C(4I)-C(5I)	1.409(14)	C(61)-Cs(1)-C(17)	135.43(11)
C(5I)-C(6I)	1.392(14)	C(62)-Cs(1)-C(17)	118.16(10)
C(1J)-C(2J)	1.392(12)	C(48)-Cs(1)-C(17)	79.70(9)
C(1J)-C(6J)	1.393(12)	N(3)-Cs(1)-C(60)	90.71(10)
C(1J)-C(7J)	1.490(13)	N(1)-Cs(1)-C(60)	124.53(9)
C(2J)-C(3J)	1.343(12)	N(2)-Cs(1)-C(60)	74.01(9)
C(3J)-C(4J)	1.381(13)	H(49)-Cs(1)-C(60)	114.1(7)
C(4J)-C(5J)	1.460(13)	C(61)-Cs(1)-C(60)	23.11(10)

C(62)-Cs(1)-C(60)	40.49(11)
C(48)-Cs(1)-C(60)	112.79(10)
C(17)-Cs(1)-C(60)	158.11(10)
N(3)-Cs(1)-C(63)	74.80(9)
N(1)-Cs(1)-C(63)	158.85(9)
N(2)-Cs(1)-C(63)	119.11(9)
H(49)-Cs(1)-C(63)	120.9(7)
C(61)-Cs(1)-C(63)	40.59(10)
C(62)-Cs(1)-C(63)	23.13(10)
C(48)-Cs(1)-C(63)	94.63(10)
C(17)-Cs(1)-C(63)	116.88(10)
C(60)-Cs(1)-C(63)	46.80(10)
N(3)-Cs(1)-C(18)	78.06(9)
N(1)-Cs(1)-C(18)	85.37(9)
N(2)-Cs(1)-C(18)	136.72(9)
H(49)-Cs(1)-C(18)	80.1(7)
C(61)-Cs(1)-C(18)	132.98(11)
C(62)-Cs(1)-C(18)	110.47(11)
C(48)-Cs(1)-C(18)	62.56(10)
C(17)-Cs(1)-C(18)	23.21(9)
C(60)-Cs(1)-C(18)	147.70(10)
C(63)-Cs(1)-C(18)	100.91(10)
N(3)-Cs(1)-C(59)	67.99(9)
N(1)-Cs(1)-C(59)	141.52(9)
N(2)-Cs(1)-C(59)	80.54(9)
H(49)-Cs(1)-C(59)	98.5(7)
C(61)-Cs(1)-C(59)	40.33(10)
C(62)-Cs(1)-C(59)	46.65(10)
C(48)-Cs(1)-C(59)	90.43(10)
C(17)-Cs(1)-C(59)	154.17(10)
C(60)-Cs(1)-C(59)	22.93(10)
C(63)-Cs(1)-C(59)	39.66(10)
C(18)-Cs(1)-C(59)	132.61(10)
C(6)-O(1)-C(7)	104.8(3)
C(7)-N(1)-C(1)	104.9(3)
C(7)-N(1)-Cs(1)	108.6(2)
C(1)-N(1)-Cs(1)	128.8(2)
C(7)-N(1)-Cs(2)	101.1(2)
C(1)-N(1)-Cs(2)	112.5(2)
Cs(1)-N(1)-Cs(2)	97.77(9)
C(6)-C(1)-C(2)	120.5(4)
C(6)-C(1)-N(1)	109.5(4)
C(2)-C(1)-N(1)	129.8(4)
C(6)-C(1)-Cs(2)	116.7(3)
C(2)-C(1)-Cs(2)	106.3(2)
N(1)-C(1)-Cs(2)	47.99(18)
C(10)-O(2)-C(9)	105.3(3)
H(49)-Cs(2)-N(4)	49.5(8)
H(49)-Cs(2)-N(1)	82.6(7)
N(4)-Cs(2)-N(1)	107.37(8)
H(49)-Cs(2)-N(2)	69.0(8)
N(4)-Cs(2)-N(2)	118.39(8)
N(1)-Cs(2)-N(2)	62.76(8)
H(49)-Cs(2)-C(75)	146.9(8)
N(4)-Cs(2)-C(75)	97.36(10)
N(1)-Cs(2)-C(75)	111.30(11)
N(2)-Cs(2)-C(75)	144.13(10)
H(49)-Cs(2)-C(76)	126.3(8)
N(4)-Cs(2)-C(76)	77.64(9)
N(1)-Cs(2)-C(76)	128.62(10)
N(2)-Cs(2)-C(76)	158.82(10)
C(75)-Cs(2)-C(76)	22.96(10)
H(49)-Cs(2)-C(31)	81.0(8)
N(4)-Cs(2)-C(31)	101.26(9)
N(1)-Cs(2)-C(31)	124.07(9)
N(2)-Cs(2)-C(31)	61.41(9)
C(75)-Cs(2)-C(31)	111.33(12)
C(76)-Cs(2)-C(31)	103.67(10)
H(49)-Cs(2)-C(30)	58.8(8)
N(4)-Cs(2)-C(30)	82.73(9)

N(1)-Cs(2)-C(30)	119.14(9)
N(2)-Cs(2)-C(30)	60.27(9)
C(75)-Cs(2)-C(30)	127.05(12)
C(76)-Cs(2)-C(30)	112.24(10)
C(31)-Cs(2)-C(30)	22.61(9)
H(49)-Cs(2)-C(35)	53.6(8)
N(4)-Cs(2)-C(35)	61.99(9)
N(1)-Cs(2)-C(35)	131.40(9)
N(2)-Cs(2)-C(35)	80.70(9)
C(75)-Cs(2)-C(35)	116.97(11)
C(76)-Cs(2)-C(35)	96.93(10)
C(31)-Cs(2)-C(35)	39.38(10)
C(30)-Cs(2)-C(35)	22.75(9)
H(49)-Cs(2)-C(32)	97.0(8)
N(4)-Cs(2)-C(32)	100.09(10)
N(1)-Cs(2)-C(32)	142.61(9)
N(2)-Cs(2)-C(32)	82.10(9)
C(75)-Cs(2)-C(32)	89.29(12)
C(76)-Cs(2)-C(32)	81.36(10)
C(31)-Cs(2)-C(32)	22.59(9)
C(30)-Cs(2)-C(32)	39.83(9)
C(35)-Cs(2)-C(32)	45.71(10)
H(49)-Cs(2)-C(74)	140.8(8)
N(4)-Cs(2)-C(74)	97.58(10)
N(1)-Cs(2)-C(74)	90.10(10)
N(2)-Cs(2)-C(74)	139.35(10)
C(75)-Cs(2)-C(74)	22.41(11)
C(76)-Cs(2)-C(74)	39.62(11)
C(31)-Cs(2)-C(74)	132.49(11)
C(30)-Cs(2)-C(74)	149.45(11)
C(35)-Cs(2)-C(74)	136.51(10)
C(32)-Cs(2)-C(74)	111.17(11)
H(49)-Cs(2)-C(33)	92.4(7)
N(4)-Cs(2)-C(33)	80.44(10)
N(1)-Cs(2)-C(33)	163.55(9)
N(2)-Cs(2)-C(33)	100.81(9)
C(75)-Cs(2)-C(33)	81.27(12)
C(76)-Cs(2)-C(33)	66.61(11)
C(31)-Cs(2)-C(33)	39.49(10)
C(30)-Cs(2)-C(33)	46.30(10)
C(35)-Cs(2)-C(33)	39.26(10)
C(32)-Cs(2)-C(33)	22.39(10)
C(74)-Cs(2)-C(33)	103.41(11)
C(9)-N(2)-C(15)	104.7(3)
C(9)-N(2)-Cs(2)	109.1(2)
C(15)-N(2)-Cs(2)	132.2(2)
C(9)-N(2)-Cs(1)	96.9(2)
C(15)-N(2)-Cs(1)	114.8(2)
Cs(2)-N(2)-Cs(1)	93.70(9)
C(1)-C(2)-C(3)	115.3(4)
C(1)-C(2)-C(16)	120.5(4)
C(3)-C(2)-C(16)	123.9(4)
N(5)-Cs(3)-N(8)	101.87(8)
N(5)-Cs(3)-N(6)	60.79(8)
N(8)-Cs(3)-N(6)	112.66(8)
N(5)-Cs(3)-H(131)	77.1(7)
N(8)-Cs(3)-H(131)	47.7(7)
N(6)-Cs(3)-H(131)	65.1(7)
N(5)-Cs(3)-C(113)	127.30(9)
N(8)-Cs(3)-C(113)	63.20(9)
N(6)-Cs(3)-C(113)	78.38(8)
H(131)-Cs(3)-C(113)	55.1(7)
N(5)-Cs(3)-C(114)	149.51(9)
N(8)-Cs(3)-C(114)	64.73(9)
N(6)-Cs(3)-C(114)	97.70(9)
H(131)-Cs(3)-C(114)	74.0(7)
C(113)-Cs(3)-C(114)	22.49(9)
N(5)-Cs(3)-C(3J)	98.2(3)
N(8)-Cs(3)-C(3J)	159.8(3)
N(6)-Cs(3)-C(3J)	78.8(3)

H(131)-Cs(3)-C(3J)	141.0(8)	C(154)-Cs(3)-C(112)	116.72(11)
C(113)-Cs(3)-C(3J)	105.3(3)	C(153)-Cs(3)-C(112)	116.99(9)
C(114)-Cs(3)-C(3J)	98.1(3)	N(5)-Cs(3)-C(155)	112.04(11)
N(5)-Cs(3)-C(89)	21.33(9)	N(8)-Cs(3)-C(155)	96.06(10)
N(8)-Cs(3)-C(89)	123.08(9)	N(6)-Cs(3)-C(155)	151.14(10)
N(6)-Cs(3)-C(89)	53.78(9)	H(131)-Cs(3)-C(155)	143.3(7)
H(131)-Cs(3)-C(89)	91.9(7)	C(113)-Cs(3)-C(155)	119.38(11)
C(113)-Cs(3)-C(89)	131.14(9)	C(114)-Cs(3)-C(155)	97.03(11)
C(114)-Cs(3)-C(89)	151.47(9)	C(3J)-Cs(3)-C(155)	74.7(3)
C(3J)-Cs(3)-C(89)	77.1(3)	C(89)-Cs(3)-C(155)	108.43(11)
N(5)-Cs(3)-C(154)	127.66(11)	C(154)-Cs(3)-C(155)	21.91(11)
N(8)-Cs(3)-C(154)	77.89(9)	C(153)-Cs(3)-C(155)	38.64(10)
N(6)-Cs(3)-C(154)	166.10(9)	C(112)-Cs(3)-C(155)	132.14(11)
H(131)-Cs(3)-C(154)	125.3(7)	C(48)-O(3)-C(47)	104.5(3)
C(113)-Cs(3)-C(154)	99.77(11)	C(48)-N(3)-C(42)	104.7(3)
C(114)-Cs(3)-C(154)	78.27(11)	C(48)-N(3)-Cs(1)	95.8(2)
C(3J)-Cs(3)-C(154)	88.6(3)	C(42)-N(3)-Cs(1)	110.7(2)
C(89)-Cs(3)-C(154)	129.03(11)	C(4)-C(3)-C(2)	122.4(4)
N(5)-Cs(3)-C(153)	119.10(9)	N(7)-Cs(4)-N(5)	126.62(9)
N(8)-Cs(3)-C(153)	57.53(9)	N(7)-Cs(4)-N(6)	98.96(9)
N(6)-Cs(3)-C(153)	170.19(9)	N(5)-Cs(4)-N(6)	63.35(8)
H(131)-Cs(3)-C(153)	105.2(7)	N(7)-Cs(4)-H(131)	47.9(7)
C(113)-Cs(3)-C(153)	95.69(9)	N(5)-Cs(4)-H(131)	79.9(7)
C(114)-Cs(3)-C(153)	78.26(9)	N(6)-Cs(4)-H(131)	67.6(7)
C(3J)-Cs(3)-C(153)	110.5(3)	N(7)-Cs(4)-C(143)	104.24(10)
C(89)-Cs(3)-C(153)	129.99(10)	N(5)-Cs(4)-C(143)	126.13(10)
C(154)-Cs(3)-C(153)	22.09(10)	N(6)-Cs(4)-C(143)	95.29(10)
N(5)-Cs(3)-C(112)	115.05(8)	H(131)-Cs(4)-C(143)	139.8(7)
N(8)-Cs(3)-C(112)	82.48(8)	N(7)-Cs(4)-C(130)	22.60(8)
N(6)-Cs(3)-C(112)	58.34(8)	N(5)-Cs(4)-C(130)	105.92(9)
H(131)-Cs(3)-C(112)	58.9(7)	N(6)-Cs(4)-C(130)	98.16(9)
C(113)-Cs(3)-C(112)	22.33(9)	H(131)-Cs(4)-C(130)	33.5(7)
C(114)-Cs(3)-C(112)	39.37(9)	C(143)-Cs(4)-C(130)	126.61(10)
C(3J)-Cs(3)-C(112)	90.5(3)	N(7)-Cs(4)-C(144)	90.62(9)
C(89)-Cs(3)-C(112)	112.12(9)	N(5)-Cs(4)-C(144)	128.91(10)

N(6)-Cs(4)-C(144)	78.94(9)
H(131)-Cs(4)-C(144)	117.3(7)
C(143)-Cs(4)-C(144)	23.15(10)
C(130)-Cs(4)-C(144)	112.75(10)
N(7)-Cs(4)-C(99)	97.05(9)
N(5)-Cs(4)-C(99)	62.81(9)
N(6)-Cs(4)-C(99)	122.47(9)
H(131)-Cs(4)-C(99)	84.5(7)
C(143)-Cs(4)-C(99)	132.87(10)
C(130)-Cs(4)-C(99)	78.47(9)
C(144)-Cs(4)-C(99)	155.38(10)
N(7)-Cs(4)-C(142)	94.67(9)
N(5)-Cs(4)-C(142)	138.65(9)
N(6)-Cs(4)-C(142)	117.87(9)
H(131)-Cs(4)-C(142)	141.0(7)
C(143)-Cs(4)-C(142)	23.11(10)
C(130)-Cs(4)-C(142)	114.23(10)
C(144)-Cs(4)-C(142)	40.43(10)
C(99)-Cs(4)-C(142)	115.33(10)
N(7)-Cs(4)-C(100)	76.16(9)
N(5)-Cs(4)-C(100)	84.11(9)
N(6)-Cs(4)-C(100)	135.68(9)
H(131)-Cs(4)-C(100)	78.2(7)
C(143)-Cs(4)-C(100)	128.86(11)
C(130)-Cs(4)-C(100)	61.09(9)
C(144)-Cs(4)-C(100)	143.95(10)
C(99)-Cs(4)-C(100)	23.32(9)
C(142)-Cs(4)-C(100)	106.46(10)
N(7)-Cs(4)-C(141)	71.93(9)
N(5)-Cs(4)-C(141)	160.81(9)
N(6)-Cs(4)-C(141)	123.35(9)
H(131)-Cs(4)-C(141)	119.2(7)
C(143)-Cs(4)-C(141)	40.31(10)
C(130)-Cs(4)-C(141)	91.34(9)
C(144)-Cs(4)-C(141)	46.56(10)
C(99)-Cs(4)-C(141)	114.17(10)
C(142)-Cs(4)-C(141)	22.95(9)
C(100)-Cs(4)-C(141)	97.42(10)
N(7)-Cs(4)-C(145)	67.85(9)
N(5)-Cs(4)-C(145)	145.53(9)
N(6)-Cs(4)-C(145)	84.62(9)
H(131)-Cs(4)-C(145)	100.3(7)
C(143)-Cs(4)-C(145)	40.20(10)
C(130)-Cs(4)-C(145)	90.15(9)
C(144)-Cs(4)-C(145)	22.82(9)
C(99)-Cs(4)-C(145)	151.60(9)
C(142)-Cs(4)-C(145)	46.45(10)
C(100)-Cs(4)-C(145)	130.00(9)
C(141)-Cs(4)-C(145)	39.41(9)
C(56)-O(4)-C(50)	103.9(3)
C(50)-N(4)-C(51)	105.0(3)
C(50)-N(4)-Cs(2)	109.1(2)
C(51)-N(4)-Cs(2)	142.4(2)
C(5)-C(4)-C(3)	121.6(4)
C(88)-O(5)-C(89)	104.2(3)
C(89)-N(5)-C(83)	105.3(3)
C(89)-N(5)-Cs(4)	108.8(2)
C(83)-N(5)-Cs(4)	131.5(2)
C(89)-N(5)-Cs(3)	98.1(2)
C(83)-N(5)-Cs(3)	107.5(2)
Cs(4)-N(5)-Cs(3)	100.85(9)
C(6)-C(5)-C(4)	115.1(4)
C(92)-O(6)-C(91)	105.4(3)
C(91)-N(6)-C(97)	105.2(3)
C(91)-N(6)-Cs(4)	95.8(2)
C(97)-N(6)-Cs(4)	112.2(2)
C(91)-N(6)-Cs(3)	107.9(2)
C(97)-N(6)-Cs(3)	133.2(2)
Cs(4)-N(6)-Cs(3)	96.39(8)
C(5)-C(6)-C(1)	124.9(4)

C(5)-C(6)-O(1)	127.3(4)
C(1)-C(6)-O(1)	107.8(3)
C(129)-O(7)-C(130)	104.4(3)
C(130)-N(7)-C(124)	105.0(3)
C(130)-N(7)-Cs(4)	95.7(2)
C(124)-N(7)-Cs(4)	118.3(2)
N(1)-C(7)-C(8)	132.4(4)
N(1)-C(7)-O(1)	113.0(3)
C(8)-C(7)-O(1)	114.6(4)
N(1)-C(7)-Cs(2)	57.8(2)
C(8)-C(7)-Cs(2)	95.2(3)
O(1)-C(7)-Cs(2)	124.9(2)
N(1)-C(7)-Cs(1)	51.5(2)
C(8)-C(7)-Cs(1)	87.8(3)
O(1)-C(7)-Cs(1)	142.6(2)
Cs(2)-C(7)-Cs(1)	78.95(8)
C(133)-O(8)-C(132)	104.0(3)
C(132)-N(8)-C(138)	104.8(3)
C(132)-N(8)-Cs(3)	112.1(2)
C(138)-N(8)-Cs(3)	140.0(2)
C(9)-C(8)-C(7)	127.7(4)
C(9)-C(8)-Cs(1)	67.4(2)
C(7)-C(8)-Cs(1)	71.2(2)
N(2)-C(9)-C(8)	132.7(4)
N(2)-C(9)-O(2)	112.9(4)
C(8)-C(9)-O(2)	114.5(4)
N(2)-C(9)-Cs(1)	61.5(2)
C(8)-C(9)-Cs(1)	91.7(3)
O(2)-C(9)-Cs(1)	124.3(2)
N(2)-C(9)-Cs(2)	51.7(2)
C(8)-C(9)-Cs(2)	86.4(2)
O(2)-C(9)-Cs(2)	147.0(3)
Cs(1)-C(9)-Cs(2)	77.35(8)
O(2)-C(10)-C(11)	127.5(4)
O(2)-C(10)-C(15)	108.0(3)

C(11)-C(10)-C(15)	124.5(4)
C(10)-C(11)-C(12)	115.1(4)
C(11)-C(12)-C(13)	121.9(4)
C(12)-C(13)-C(14)	122.7(4)
C(15)-C(14)-C(13)	115.6(4)
C(15)-C(14)-C(29)	120.5(4)
C(13)-C(14)-C(29)	123.9(4)
C(10)-C(15)-C(14)	120.1(4)
C(10)-C(15)-N(2)	109.1(4)
C(14)-C(15)-N(2)	130.8(4)
C(23)-C(16)-C(2)	113.4(3)
C(23)-C(16)-C(17)	111.4(3)
C(2)-C(16)-C(17)	111.4(3)
C(22)-C(17)-C(18)	118.5(4)
C(22)-C(17)-C(16)	123.5(4)
C(18)-C(17)-C(16)	118.0(4)
C(22)-C(17)-Cs(1)	84.0(2)
C(18)-C(17)-Cs(1)	81.2(2)
C(16)-C(17)-Cs(1)	104.3(2)
C(19)-C(18)-C(17)	120.4(4)
C(19)-C(18)-Cs(1)	86.1(3)
C(17)-C(18)-Cs(1)	75.6(2)
C(20)-C(19)-C(18)	120.0(4)
C(20)-C(19)-Cs(1)	84.3(3)
C(18)-C(19)-Cs(1)	71.8(2)
C(19)-C(20)-C(21)	119.7(4)
C(19)-C(20)-Cs(1)	74.6(3)
C(21)-C(20)-Cs(1)	77.0(3)
C(20)-C(21)-C(22)	120.4(4)
C(20)-C(21)-Cs(1)	81.9(3)
C(22)-C(21)-Cs(1)	72.1(2)
C(17)-C(22)-C(21)	120.9(4)
C(17)-C(22)-Cs(1)	73.4(2)
C(21)-C(22)-Cs(1)	86.3(2)
C(28)-C(23)-C(24)	118.3(4)

C(28)-C(23)-C(16)	120.1(4)	C(37)-C(38)-C(39)	120.8(4)
C(24)-C(23)-C(16)	121.6(4)	C(38)-C(39)-C(40)	119.0(4)
C(25)-C(24)-C(23)	120.7(4)	C(39)-C(40)-C(41)	120.3(4)
C(26)-C(25)-C(24)	120.4(4)	C(40)-C(41)-C(36)	120.5(4)
C(27)-C(26)-C(25)	119.6(4)	N(3)-C(42)-C(47)	109.7(3)
C(26)-C(27)-C(28)	120.2(4)	N(3)-C(42)-C(43)	130.2(4)
C(23)-C(28)-C(27)	120.9(4)	C(47)-C(42)-C(43)	120.1(4)
C(14)-C(29)-C(36)	114.2(3)	N(3)-C(42)-Cs(1)	49.06(19)
C(14)-C(29)-C(30)	112.3(3)	C(47)-C(42)-Cs(1)	111.0(3)
C(36)-C(29)-C(30)	110.5(3)	C(43)-C(42)-Cs(1)	109.3(3)
C(31)-C(30)-C(35)	118.1(4)	C(44)-C(43)-C(42)	116.4(4)
C(31)-C(30)-C(29)	123.9(4)	C(44)-C(43)-C(57)	125.1(4)
C(35)-C(30)-C(29)	118.0(4)	C(42)-C(43)-C(57)	118.5(4)
C(31)-C(30)-Cs(2)	78.6(2)	C(43)-C(44)-C(45)	122.2(4)
C(35)-C(30)-Cs(2)	79.3(2)	C(44)-C(45)-C(46)	122.0(4)
C(29)-C(30)-Cs(2)	111.3(2)	C(47)-C(46)-C(45)	115.0(4)
C(30)-C(31)-C(32)	121.0(4)	C(46)-C(47)-C(42)	124.4(4)
C(30)-C(31)-Cs(2)	78.8(2)	C(46)-C(47)-O(3)	128.0(4)
C(32)-C(31)-Cs(2)	79.5(2)	C(42)-C(47)-O(3)	107.6(3)
C(33)-C(32)-C(31)	120.0(4)	N(3)-C(48)-O(3)	113.4(3)
C(33)-C(32)-Cs(2)	79.4(3)	N(3)-C(48)-C(49)	125.4(4)
C(31)-C(32)-Cs(2)	77.9(2)	O(3)-C(48)-C(49)	121.1(3)
C(34)-C(33)-C(32)	120.2(4)	N(3)-C(48)-Cs(1)	61.4(2)
C(34)-C(33)-Cs(2)	78.9(2)	O(3)-C(48)-Cs(1)	120.6(2)
C(32)-C(33)-Cs(2)	78.2(3)	C(49)-C(48)-Cs(1)	86.9(2)
C(33)-C(34)-C(35)	119.4(4)	H(49)-C(49)-C(48)	115(3)
C(33)-C(34)-Cs(2)	78.7(2)	H(49)-C(49)-C(50)	116(3)
C(35)-C(34)-Cs(2)	78.0(2)	C(48)-C(49)-C(50)	128.7(4)
C(34)-C(35)-C(30)	121.3(4)	H(49)-C(49)-Cs(1)	63(3)
C(34)-C(35)-Cs(2)	79.5(2)	C(48)-C(49)-Cs(1)	70.5(2)
C(30)-C(35)-Cs(2)	78.0(2)	C(50)-C(49)-Cs(1)	140.3(3)
C(37)-C(36)-C(41)	118.2(4)	H(49)-C(49)-Cs(2)	41(3)
C(37)-C(36)-C(29)	121.6(4)	C(48)-C(49)-Cs(2)	148.1(3)
C(41)-C(36)-C(29)	120.2(4)	C(50)-C(49)-Cs(2)	80.5(2)
C(38)-C(37)-C(36)	121.1(4)	Cs(1)-C(49)-Cs(2)	78.37(8)

N(4)-C(50)-O(4)	113.9(3)	C(60)-C(61)-Cs(1)	80.0(2)
N(4)-C(50)-C(49)	126.8(4)	C(61)-C(62)-C(63)	120.2(4)
O(4)-C(50)-C(49)	119.2(4)	C(61)-C(62)-Cs(1)	78.0(2)
N(4)-C(50)-Cs(2)	51.5(2)	C(63)-C(62)-Cs(1)	81.0(2)
O(4)-C(50)-Cs(2)	159.1(2)	C(62)-C(63)-C(58)	121.3(4)
C(49)-C(50)-Cs(2)	77.9(2)	C(62)-C(63)-Cs(1)	75.8(2)
C(56)-C(51)-N(4)	109.1(3)	C(58)-C(63)-Cs(1)	81.9(2)
C(56)-C(51)-C(52)	120.0(3)	C(69)-C(64)-C(65)	118.0(4)
N(4)-C(51)-C(52)	130.9(4)	C(69)-C(64)-C(57)	123.9(4)
C(51)-C(52)-C(53)	115.6(4)	C(65)-C(64)-C(57)	118.0(4)
C(51)-C(52)-C(70)	121.5(3)	C(64)-C(65)-C(66)	120.7(5)
C(53)-C(52)-C(70)	122.9(3)	C(67)-C(66)-C(65)	120.3(6)
C(54)-C(53)-C(52)	122.9(4)	C(66)-C(67)-C(68)	119.9(5)
C(55)-C(54)-C(53)	121.2(4)	C(67)-C(68)-C(69)	120.1(6)
C(56)-C(55)-C(54)	115.3(4)	C(64)-C(69)-C(68)	120.9(5)
C(55)-C(56)-O(4)	126.9(4)	C(77)-C(70)-C(71)	109.0(3)
C(55)-C(56)-C(51)	125.0(4)	C(77)-C(70)-C(52)	112.8(3)
O(4)-C(56)-C(51)	108.1(3)	C(71)-C(70)-C(52)	114.8(3)
C(58)-C(57)-C(64)	112.8(3)	C(72)-C(71)-C(76)	118.4(4)
C(58)-C(57)-C(43)	111.9(3)	C(72)-C(71)-C(70)	123.5(4)
C(64)-C(57)-C(43)	112.6(3)	C(76)-C(71)-C(70)	118.1(4)
C(63)-C(58)-C(59)	117.6(4)	C(72)-C(71)-Cs(2)	80.8(2)
C(63)-C(58)-C(57)	122.1(4)	C(76)-C(71)-Cs(2)	75.4(2)
C(59)-C(58)-C(57)	120.3(4)	C(70)-C(71)-Cs(2)	117.1(2)
C(63)-C(58)-Cs(1)	75.4(2)	C(71)-C(72)-C(73)	121.0(4)
C(59)-C(58)-Cs(1)	77.1(2)	C(71)-C(72)-Cs(2)	77.1(2)
C(57)-C(58)-Cs(1)	118.3(2)	C(73)-C(72)-Cs(2)	78.4(2)
C(60)-C(59)-C(58)	121.1(4)	C(74)-C(73)-C(72)	119.9(5)
C(60)-C(59)-Cs(1)	75.5(2)	C(74)-C(73)-Cs(2)	77.0(3)
C(58)-C(59)-Cs(1)	80.2(2)	C(72)-C(73)-Cs(2)	79.4(2)
C(61)-C(60)-C(59)	120.0(4)	C(75)-C(74)-C(73)	120.0(4)
C(61)-C(60)-Cs(1)	76.9(2)	C(75)-C(74)-Cs(2)	76.1(3)
C(59)-C(60)-Cs(1)	81.6(2)	C(73)-C(74)-Cs(2)	80.9(3)
C(62)-C(61)-C(60)	119.7(4)	C(74)-C(75)-C(76)	119.9(5)
C(62)-C(61)-Cs(1)	78.8(2)	C(74)-C(75)-Cs(2)	81.5(3)

C(76)-C(75)-Cs(2)	78.6(3)
C(71)-C(76)-C(75)	120.9(5)
C(71)-C(76)-Cs(2)	82.0(2)
C(75)-C(76)-Cs(2)	78.5(3)
C(78)-C(77)-C(82)	118.2(4)
C(78)-C(77)-C(70)	121.3(4)
C(82)-C(77)-C(70)	120.5(4)
C(77)-C(78)-C(79)	121.0(4)
C(80)-C(79)-C(78)	120.0(4)
C(79)-C(80)-C(81)	119.6(4)
C(82)-C(81)-C(80)	120.1(4)
C(81)-C(82)-C(77)	121.2(4)
C(88)-C(83)-C(84)	120.7(4)
C(88)-C(83)-N(5)	108.7(4)
C(84)-C(83)-N(5)	130.5(4)
C(88)-C(83)-Cs(3)	111.4(3)
C(84)-C(83)-Cs(3)	107.8(2)
N(5)-C(83)-Cs(3)	52.00(18)
C(83)-C(84)-C(85)	115.9(4)
C(83)-C(84)-C(98)	120.2(4)
C(85)-C(84)-C(98)	123.7(4)
C(86)-C(85)-C(84)	122.0(4)
C(87)-C(86)-C(85)	122.1(4)
C(88)-C(87)-C(86)	115.3(4)
O(5)-C(88)-C(87)	127.5(4)
O(5)-C(88)-C(83)	108.7(4)
C(87)-C(88)-C(83)	123.8(4)
N(5)-C(89)-C(90)	132.4(4)
N(5)-C(89)-O(5)	113.1(4)
C(90)-C(89)-O(5)	114.4(4)
N(5)-C(89)-Cs(3)	60.6(2)
C(90)-C(89)-Cs(3)	97.9(3)
O(5)-C(89)-Cs(3)	118.3(2)
N(5)-C(89)-Cs(4)	51.5(2)
C(90)-C(89)-Cs(4)	86.0(2)

O(5)-C(89)-Cs(4)	146.6(2)
Cs(3)-C(89)-Cs(4)	82.18(8)
C(91)-C(90)-C(89)	126.8(4)
C(91)-C(90)-Cs(4)	66.3(2)
C(89)-C(90)-Cs(4)	73.1(2)
N(6)-C(91)-C(90)	132.7(4)
N(6)-C(91)-O(6)	112.5(3)
C(90)-C(91)-O(6)	114.7(4)
N(6)-C(91)-Cs(4)	62.3(2)
C(90)-C(91)-Cs(4)	92.7(3)
O(6)-C(91)-Cs(4)	121.8(2)
N(6)-C(91)-Cs(3)	53.2(2)
C(90)-C(91)-Cs(3)	85.1(3)
O(6)-C(91)-Cs(3)	147.5(2)
Cs(4)-C(91)-Cs(3)	80.10(8)
C(93)-C(92)-O(6)	127.9(4)
C(93)-C(92)-C(97)	124.7(4)
O(6)-C(92)-C(97)	107.3(4)
C(92)-C(93)-C(94)	115.4(4)
C(93)-C(94)-C(95)	121.7(4)
C(94)-C(95)-C(96)	122.1(4)
C(95)-C(96)-C(97)	116.6(4)
C(95)-C(96)-C(111)	123.8(4)
C(97)-C(96)-C(111)	119.6(3)
C(92)-C(97)-C(96)	119.2(4)
C(92)-C(97)-N(6)	109.6(4)
C(96)-C(97)-N(6)	131.1(4)
C(92)-C(97)-Cs(4)	112.5(3)
C(96)-C(97)-Cs(4)	109.4(2)
N(6)-C(97)-Cs(4)	48.43(18)
C(105)-C(98)-C(84)	114.0(3)
C(105)-C(98)-C(99)	110.4(3)
C(84)-C(98)-C(99)	112.0(3)
C(104)-C(99)-C(100)	118.1(4)
C(104)-C(99)-C(98)	123.6(4)

C(100)-C(99)-C(98)	118.3(4)	C(111)-C(112)-Cs(3)	111.2(2)
C(104)-C(99)-Cs(4)	82.8(2)	C(114)-C(113)-C(112)	121.8(4)
C(100)-C(99)-Cs(4)	78.8(2)	C(114)-C(113)-Cs(3)	79.7(2)
C(98)-C(99)-Cs(4)	107.0(2)	C(112)-C(113)-Cs(3)	83.1(2)
C(101)-C(100)-C(99)	120.9(4)	C(113)-C(114)-C(115)	119.7(4)
C(101)-C(100)-Cs(4)	83.7(2)	C(113)-C(114)-Cs(3)	77.8(2)
C(99)-C(100)-Cs(4)	77.9(2)	C(115)-C(114)-Cs(3)	83.5(2)
C(100)-C(101)-C(102)	120.4(4)	C(114)-C(115)-C(116)	119.7(4)
C(100)-C(101)-Cs(4)	73.8(2)	C(114)-C(115)-Cs(3)	74.5(2)
C(102)-C(101)-Cs(4)	83.3(2)	C(116)-C(115)-Cs(3)	83.2(3)
C(103)-C(102)-C(101)	119.5(4)	C(115)-C(116)-C(117)	120.0(4)
C(103)-C(102)-Cs(4)	79.1(2)	C(115)-C(116)-Cs(3)	75.4(3)
C(101)-C(102)-Cs(4)	74.8(2)	C(117)-C(116)-Cs(3)	78.5(2)
C(102)-C(103)-C(104)	120.2(4)	C(112)-C(117)-C(116)	121.0(4)
C(102)-C(103)-Cs(4)	79.4(2)	C(112)-C(117)-Cs(3)	74.9(2)
C(104)-C(103)-Cs(4)	73.7(2)	C(116)-C(117)-Cs(3)	80.1(3)
C(99)-C(104)-C(103)	120.8(4)	C(123)-C(118)-C(119)	118.5(4)
C(99)-C(104)-Cs(4)	74.4(2)	C(123)-C(118)-C(111)	121.6(3)
C(103)-C(104)-Cs(4)	84.2(2)	C(119)-C(118)-C(111)	119.9(3)
C(106)-C(105)-C(110)	118.8(4)	C(120)-C(119)-C(118)	120.8(4)
C(106)-C(105)-C(98)	121.5(4)	C(121)-C(120)-C(119)	120.4(4)
C(110)-C(105)-C(98)	119.7(4)	C(120)-C(121)-C(122)	119.2(4)
C(105)-C(106)-C(107)	120.6(4)	C(123)-C(122)-C(121)	120.4(4)
C(108)-C(107)-C(106)	119.9(4)	C(122)-C(123)-C(118)	120.7(4)
C(109)-C(108)-C(107)	120.1(4)	N(7)-C(124)-C(129)	109.7(3)
C(108)-C(109)-C(110)	119.7(5)	N(7)-C(124)-C(125)	129.5(4)
C(109)-C(110)-C(105)	120.9(4)	C(129)-C(124)-C(125)	120.8(4)
C(96)-C(111)-C(118)	113.1(3)	N(7)-C(124)-Cs(4)	43.46(18)
C(96)-C(111)-C(112)	112.4(3)	C(129)-C(124)-Cs(4)	114.1(2)
C(118)-C(111)-C(112)	112.2(3)	C(125)-C(124)-Cs(4)	110.1(2)
C(117)-C(112)-C(113)	117.7(4)	C(126)-C(125)-C(124)	115.3(4)
C(117)-C(112)-C(111)	123.6(4)	C(126)-C(125)-C(139)	126.0(4)
C(113)-C(112)-C(111)	118.7(3)	C(124)-C(125)-C(139)	118.6(3)
C(117)-C(112)-Cs(3)	83.5(2)	C(125)-C(126)-C(127)	122.6(4)
C(113)-C(112)-Cs(3)	74.6(2)	C(128)-C(127)-C(126)	121.7(4)

C(129)-C(128)-C(127)	115.1(4)	C(125)-C(139)-C(140)	112.9(3)
C(128)-C(129)-O(7)	128.0(4)	C(146)-C(139)-C(140)	111.8(3)
C(128)-C(129)-C(124)	124.4(4)	C(145)-C(140)-C(141)	118.2(4)
O(7)-C(129)-C(124)	107.6(3)	C(145)-C(140)-C(139)	119.6(4)
N(7)-C(130)-C(131)	126.5(4)	C(141)-C(140)-C(139)	122.2(4)
N(7)-C(130)-O(7)	113.3(3)	C(145)-C(140)-Cs(4)	76.0(2)
C(131)-C(130)-O(7)	120.2(3)	C(141)-C(140)-Cs(4)	75.5(2)
N(7)-C(130)-Cs(4)	61.7(2)	C(139)-C(140)-Cs(4)	120.0(2)
C(131)-C(130)-Cs(4)	84.0(2)	C(140)-C(141)-C(142)	120.9(4)
O(7)-C(130)-Cs(4)	127.5(2)	C(140)-C(141)-Cs(4)	82.0(2)
H(131)-C(131)-C(130)	114(3)	C(142)-C(141)-Cs(4)	75.8(2)
H(131)-C(131)-C(132)	117(3)	C(143)-C(142)-C(141)	119.8(4)
C(130)-C(131)-C(132)	128.5(4)	C(143)-C(142)-Cs(4)	76.9(2)
H(131)-C(131)-Cs(4)	61(3)	C(141)-C(142)-Cs(4)	81.3(2)
C(130)-C(131)-Cs(4)	73.3(2)	C(142)-C(143)-C(144)	120.0(4)
C(132)-C(131)-Cs(4)	140.8(3)	C(142)-C(143)-Cs(4)	80.0(2)
N(8)-C(132)-O(8)	114.1(3)	C(144)-C(143)-Cs(4)	79.6(2)
N(8)-C(132)-C(131)	128.0(4)	C(143)-C(144)-C(145)	120.0(4)
O(8)-C(132)-C(131)	117.8(3)	C(143)-C(144)-Cs(4)	77.2(2)
N(8)-C(132)-Cs(3)	49.5(2)	C(145)-C(144)-Cs(4)	82.2(2)
O(8)-C(132)-Cs(3)	158.3(2)	C(144)-C(145)-C(140)	121.1(4)
C(131)-C(132)-Cs(3)	80.8(2)	C(144)-C(145)-Cs(4)	75.0(2)
C(134)-C(133)-O(8)	126.7(4)	C(140)-C(145)-Cs(4)	81.7(2)
C(134)-C(133)-C(138)	125.0(4)	C(147)-C(146)-C(151)	118.5(4)
O(8)-C(133)-C(138)	108.3(3)	C(147)-C(146)-C(139)	118.9(4)
C(133)-C(134)-C(135)	115.2(4)	C(151)-C(146)-C(139)	122.6(4)
C(136)-C(135)-C(134)	121.1(4)	C(148)-C(147)-C(146)	121.1(4)
C(135)-C(136)-C(137)	123.6(4)	C(149)-C(148)-C(147)	120.2(5)
C(136)-C(137)-C(138)	115.5(4)	C(148)-C(149)-C(150)	119.7(4)
C(136)-C(137)-C(152)	122.8(4)	C(149)-C(150)-C(151)	120.3(4)
C(138)-C(137)-C(152)	121.7(4)	C(150)-C(151)-C(146)	120.2(4)
C(133)-C(138)-N(8)	108.8(3)	C(137)-C(152)-C(159)	113.3(3)
C(133)-C(138)-C(137)	119.5(4)	C(137)-C(152)-C(153)	114.6(3)
N(8)-C(138)-C(137)	131.6(4)	C(159)-C(152)-C(153)	108.5(3)
C(125)-C(139)-C(146)	114.2(3)	C(158)-C(153)-C(154)	118.4(4)

C(158)-C(153)-C(152)	123.6(4)
C(154)-C(153)-C(152)	117.9(4)
C(158)-C(153)-Cs(3)	81.6(2)
C(154)-C(153)-Cs(3)	77.9(3)
C(152)-C(153)-Cs(3)	114.0(2)
C(155)-C(154)-C(153)	121.2(5)
C(155)-C(154)-Cs(3)	81.2(3)
C(153)-C(154)-Cs(3)	80.0(3)
C(154)-C(155)-C(156)	119.8(5)
C(154)-C(155)-Cs(3)	76.9(3)
C(156)-C(155)-Cs(3)	80.8(3)
C(157)-C(156)-C(155)	119.7(5)
C(157)-C(156)-Cs(3)	79.6(3)
C(155)-C(156)-Cs(3)	77.5(3)
C(156)-C(157)-C(158)	119.8(5)
C(156)-C(157)-Cs(3)	78.9(3)
C(158)-C(157)-Cs(3)	78.4(2)
C(153)-C(158)-C(157)	121.1(4)
C(153)-C(158)-Cs(3)	76.8(2)
C(157)-C(158)-Cs(3)	79.9(3)
C(160)-C(159)-C(164)	119.2(4)
C(160)-C(159)-C(152)	120.7(4)
C(164)-C(159)-C(152)	120.1(4)
C(159)-C(160)-C(161)	119.8(4)
C(162)-C(161)-C(160)	121.1(4)
C(161)-C(162)-C(163)	119.5(4)
C(162)-C(163)-C(164)	119.9(4)
C(163)-C(164)-C(159)	120.5(4)
C(6A)-C(1A)-C(2A)	117.7(6)
C(6A)-C(1A)-C(7A)	121.2(6)
C(2A)-C(1A)-C(7A)	121.0(6)
C(3A)-C(2A)-C(1A)	121.0(7)
C(2A)-C(3A)-C(4A)	120.3(7)
C(3A)-C(4A)-C(5A)	119.3(6)
C(6A)-C(5A)-C(4A)	120.3(7)
C(5A)-C(6A)-C(1A)	121.5(7)
C(6B)-C(1B)-C(2B)	120.1(13)
C(6B)-C(1B)-C(7B)	122.0(13)
C(2B)-C(1B)-C(7B)	117.9(13)
C(3B)-C(2B)-C(1B)	119.3(14)
C(4B)-C(3B)-C(2B)	120.2(14)
C(3B)-C(4B)-C(5B)	121.8(14)
C(6B)-C(5B)-C(4B)	117.3(14)
C(1B)-C(6B)-C(5B)	121.1(14)
C(2C)-C(1C)-C(6C)	123.7(12)
C(2C)-C(1C)-C(7C)	116.5(12)
C(6C)-C(1C)-C(7C)	119.4(12)
C(1C)-C(2C)-C(3C)	117.6(12)
C(2C)-C(3C)-C(4C)	120.7(13)
C(2C)-C(3C)-Cs(3)	103.0(8)
C(4C)-C(3C)-Cs(3)	84.7(10)
C(3C)-C(4C)-C(5C)	118.6(13)
C(3C)-C(4C)-Cs(3)	74.0(9)
C(5C)-C(4C)-Cs(3)	117.8(12)
C(6C)-C(5C)-C(4C)	121.3(13)
C(5C)-C(6C)-C(1C)	117.1(12)
C(2D)-C(1D)-C(6D)	113.7(11)
C(2D)-C(1D)-C(7D)	113.0(12)
C(6D)-C(1D)-C(7D)	132.0(13)
C(3D)-C(2D)-C(1D)	125.2(13)
C(2D)-C(3D)-C(4D)	119.6(13)
C(3D)-C(4D)-C(5D)	117.3(12)
C(4D)-C(5D)-C(6D)	120.6(12)
C(1D)-C(6D)-C(5D)	122.8(12)
C(1D)-C(6D)-Cs(3)	90.2(10)
C(5D)-C(6D)-Cs(3)	93.3(10)
C(6E)-C(1E)-C(2E)	116.3(14)
C(6E)-C(1E)-C(7E)	121.6(15)
C(2E)-C(1E)-C(7E)	121.7(14)
C(3E)-C(2E)-C(1E)	123.1(14)

C(4E)-C(3E)-C(2E)	119.9(15)	C(1H)-C(2H)-C(3H)	123.4(16)
C(3E)-C(4E)-C(5E)	120.0(14)	C(4H)-C(3H)-C(2H)	117.2(16)
C(6E)-C(5E)-C(4E)	118.5(14)	C(3H)-C(4H)-C(5H)	118.9(16)
C(1E)-C(6E)-C(5E)	122.1(15)	C(6H)-C(5H)-C(4H)	119.7(17)
C(2F)-C(1F)-C(6F)	118.8(6)	C(5H)-C(6H)-C(1H)	123.2(16)
C(2F)-C(1F)-C(7F)	120.6(6)	C(6I)-C(1I)-C(2I)	120.5(15)
C(6F)-C(1F)-C(7F)	120.6(6)	C(6I)-C(1I)-C(7I)	124.2(18)
C(1F)-C(2F)-C(3F)	121.1(6)	C(2I)-C(1I)-C(7I)	115.2(17)
C(2F)-C(3F)-C(4F)	119.1(6)	C(1I)-C(2I)-C(3I)	120.5(17)
C(2F)-C(3F)-C(7K)	116.9(11)	C(2I)-C(3I)-C(4I)	119.4(17)
C(4F)-C(3F)-C(7K)	120.3(11)	C(5I)-C(4I)-C(3I)	118.9(16)
C(3F)-C(4F)-C(5F)	120.4(7)	C(6I)-C(5I)-C(4I)	120.2(17)
C(6F)-C(5F)-C(4F)	119.6(6)	C(1I)-C(6I)-C(5I)	120.1(17)
C(5F)-C(6F)-C(1F)	120.9(6)	C(2J)-C(1J)-C(6J)	119.1(12)
C(6G)-C(1G)-C(2G)	117.9(8)	C(2J)-C(1J)-C(7J)	115.8(13)
C(6G)-C(1G)-C(7G)	116.8(8)	C(6J)-C(1J)-C(7J)	124.9(13)
C(2G)-C(1G)-C(7G)	125.3(7)	C(3J)-C(2J)-C(1J)	119.9(13)
C(3G)-C(2G)-C(1G)	122.8(8)	C(3J)-C(2J)-Cs(3)	70.0(8)
C(4G)-C(3G)-C(2G)	118.0(8)	C(1J)-C(2J)-Cs(3)	123.7(9)
C(5G)-C(4G)-C(3G)	118.7(8)	C(2J)-C(3J)-C(4J)	121.8(14)
C(4G)-C(5G)-C(6G)	122.6(8)	C(2J)-C(3J)-Cs(3)	89.4(9)
C(1G)-C(6G)-C(5G)	119.5(8)	C(4J)-C(3J)-Cs(3)	110.2(11)
C(6H)-C(1H)-C(2H)	114.2(14)	C(3J)-C(4J)-C(5J)	121.9(13)
C(6H)-C(1H)-C(7H)	117.3(16)	C(6J)-C(5J)-C(4J)	112.7(13)
C(2H)-C(1H)-C(7H)	127.7(17)	C(1J)-C(6J)-C(5J)	124.3(13)

XRAY: Compound 11

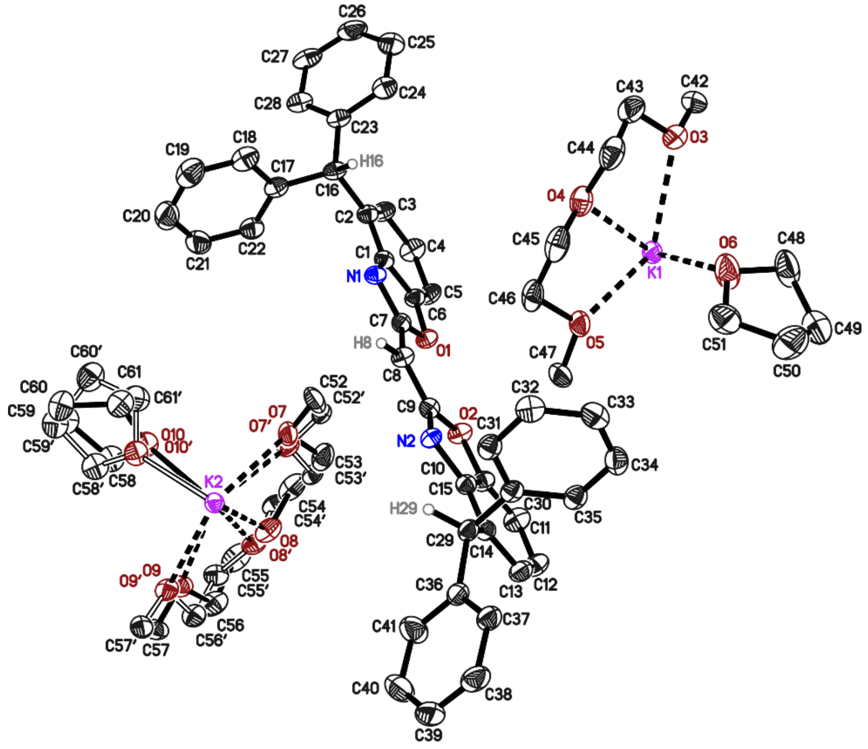

Figure S105. Asymmetric unit of 11. Displacement parameters are depicted at 50% probability. Hydrogen atoms except the hydrogen atoms bound to the methylene backbone (H8) and benzylic groups (H16, H29) are omitted for clarity.

The thf molecule and the crown coordinated to K2 are disordered about two positions. The occupancy of the minor position refined to 0.232(3). The disordered groups are refined with distance restraints and restraints for the anisotropic displacement parameter.

Table S8. Bond lengths [Å] and angles [°] for **11**.

K(1)-O(5)	2.7329(13)	C(1)-C(6)	1.391(2)
K(1)-O(6)	2.7758(16)	C(1)-C(2)	1.400(2)
K(1)-O(4)	2.7985(13)	O(2)-C(10)	1.3888(19)
K(1)-O(3)	2.8135(14)	O(2)-C(9)	1.4023(19)
O(1)-C(6)	1.3856(18)	K(2)-O(10')	2.641(10)
O(1)-C(7)	1.3972(19)	K(2)-O(9')	2.724(11)
N(1)-C(7)	1.329(2)	K(2)-O(10)	2.745(3)
N(1)-C(1)	1.383(2)	K(2)-O(9)	2.750(3)

K(2)-O(7)	2.766(3)	C(25)-C(24)	1.389(3)
K(2)-O(7')	2.807(11)	C(27)-C(26)	1.373(3)
N(2)-C(9)	1.327(2)	C(27)-C(28)	1.391(2)
N(2)-C(15)	1.380(2)	C(29)-C(30)	1.520(2)
C(2)-C(3)	1.392(2)	C(29)-C(36)	1.526(2)
C(2)-C(16)	1.523(2)	C(30)-C(31)	1.391(2)
C(3)-C(4)	1.394(2)	C(30)-C(35)	1.396(2)
O(3)-C(43)	1.418(2)	C(31)-C(32)	1.387(3)
O(3)-C(42)	1.424(2)	C(32)-C(33)	1.384(3)
C(4)-C(5)	1.399(2)	C(33)-C(34)	1.386(3)
O(4)-C(44)	1.424(2)	C(34)-C(35)	1.389(3)
O(4)-C(45)	1.428(2)	C(36)-C(37)	1.383(2)
O(5)-C(46)	1.418(2)	C(36)-C(41)	1.385(3)
O(5)-C(47)	1.421(2)	C(37)-C(38)	1.390(2)
C(5)-C(6)	1.371(2)	C(38)-C(39)	1.367(3)
C(7)-C(8)	1.390(2)	C(39)-C(40)	1.379(3)
C(9)-C(8)	1.389(2)	C(40)-C(41)	1.383(3)
C(13)-C(12)	1.395(2)	C(42)-C(47)#1	1.482(3)
C(13)-C(14)	1.396(2)	C(43)-C(44)	1.483(3)
C(16)-C(17)	1.524(2)	C(45)-C(46)	1.482(3)
C(16)-C(23)	1.526(2)	O(6)-C(48)	1.428(2)
C(10)-C(11)	1.373(2)	O(6)-C(51)	1.431(2)
C(10)-C(15)	1.391(2)	C(48)-C(49)	1.494(3)
C(12)-C(11)	1.396(2)	C(49)-C(50)	1.500(3)
C(14)-C(15)	1.398(2)	C(50)-C(51)	1.506(3)
C(14)-C(29)	1.521(2)	C(52)-O(7)	1.435(4)
C(17)-C(22)	1.387(3)	C(52)-C(57)#2	1.479(5)
C(17)-C(18)	1.394(2)	O(7)-C(53)	1.417(4)
C(18)-C(19)	1.381(3)	C(53)-C(54)	1.497(5)
C(19)-C(20)	1.386(3)	C(54)-O(8)	1.429(4)
C(21)-C(20)	1.378(3)	O(8)-C(55)	1.419(4)
C(21)-C(22)	1.389(3)	C(55)-C(56)	1.494(5)
C(23)-C(24)	1.385(3)	C(56)-O(9)	1.417(4)
C(23)-C(28)	1.387(2)	O(9)-C(57)	1.426(4)
C(25)-C(26)	1.376(3)	C(52')-O(7')	1.416(10)

C(52')-C(57')#2	1.504(9)
O(7')-C(53')	1.404(9)
C(53')-C(54')	1.491(10)
C(54')-O(8')	1.423(10)
O(8')-C(55')	1.426(10)
C(55')-C(56')	1.493(10)
C(56')-O(9')	1.414(9)
O(9')-C(57')	1.423(10)
O(10)-C(58)	1.426(3)
O(10)-C(61)	1.463(4)
C(58)-C(59)	1.526(4)
C(59)-C(60)	1.529(3)
C(60)-C(61)	1.508(4)
O(10')-C(61')	1.405(12)
O(10')-C(58')	1.432(11)
C(58')-C(59')	1.507(11)
C(59')-C(60')	1.503(11)
C(60')-C(61')	1.485(12)

O(5)#1-K(1)-O(5)	180.0
O(5)#1-K(1)-O(6)	98.88(5)
O(5)-K(1)-O(6)	81.12(5)
O(6)-K(1)-O(6)#1	180.00(6)
O(5)#1-K(1)-O(4)	118.57(4)
O(5)-K(1)-O(4)	61.43(4)
O(6)-K(1)-O(4)	81.03(5)
O(6)#1-K(1)-O(4)	98.97(5)
O(4)-K(1)-O(4)#1	180.00(6)
O(5)#1-K(1)-O(3)	59.70(4)
O(5)-K(1)-O(3)	120.30(4)
O(6)-K(1)-O(3)	78.56(5)
O(6)#1-K(1)-O(3)	101.44(5)
O(4)-K(1)-O(3)	60.25(4)
O(4)#1-K(1)-O(3)	119.75(4)
O(3)-K(1)-O(3)#1	180.0

C(6)-O(1)-C(7)	103.94(12)
C(7)-N(1)-C(1)	104.75(13)
N(1)-C(1)-C(6)	109.79(13)
N(1)-C(1)-C(2)	129.70(15)
C(6)-C(1)-C(2)	120.50(14)
C(10)-O(2)-C(9)	104.13(12)
O(10')#2-K(2)-O(10')	180.0(3)
O(10')#2-K(2)-O(9')	97.9(3)
O(10')-K(2)-O(9')	82.1(3)
O(9')#2-K(2)-O(9')	180.0(4)
O(10)-K(2)-O(10)#2	180.00(9)
O(10)-K(2)-O(9)#2	95.45(9)
O(10)-K(2)-O(9)	84.54(9)
O(9)#2-K(2)-O(9)	180.0
O(10)-K(2)-O(7)#2	98.12(8)
O(9)-K(2)-O(7)#2	62.19(8)
O(10)-K(2)-O(7)	81.88(8)
O(9)-K(2)-O(7)	117.81(8)
O(7)#2-K(2)-O(7)	180.00(14)
O(10')#2-K(2)-O(7')	89.9(3)
O(10')-K(2)-O(7')	90.1(3)
O(9')#2-K(2)-O(7')	62.0(2)
O(9')-K(2)-O(7')	118.0(2)
O(7')#2-K(2)-O(7')	180.0
C(9)-N(2)-C(15)	105.20(13)
C(3)-C(2)-C(1)	115.91(15)
C(3)-C(2)-C(16)	124.64(14)
C(1)-C(2)-C(16)	119.44(14)
C(2)-C(3)-C(4)	122.56(15)
C(43)-O(3)-C(42)	113.89(15)
C(43)-O(3)-K(1)	115.82(11)
C(42)-O(3)-K(1)	113.80(11)
C(3)-C(4)-C(5)	121.44(15)
C(44)-O(4)-C(45)	111.14(14)
C(44)-O(4)-K(1)	111.08(10)

C(45)-O(4)-K(1)	112.25(10)	C(20)-C(21)-C(22)	120.5(2)
C(46)-O(5)-C(47)	111.62(14)	C(21)-C(20)-C(19)	119.1(2)
C(46)-O(5)-K(1)	115.71(10)	C(24)-C(23)-C(28)	118.15(17)
C(47)-O(5)-K(1)	118.37(10)	C(24)-C(23)-C(16)	119.37(16)
C(6)-C(5)-C(4)	115.41(15)	C(28)-C(23)-C(16)	122.38(16)
C(5)-C(6)-O(1)	128.02(15)	C(17)-C(22)-C(21)	120.85(18)
C(5)-C(6)-C(1)	124.18(15)	C(26)-C(25)-C(24)	120.3(2)
O(1)-C(6)-C(1)	107.79(13)	C(23)-C(24)-C(25)	121.05(19)
N(1)-C(7)-C(8)	125.40(14)	C(26)-C(27)-C(28)	120.79(19)
N(1)-C(7)-O(1)	113.73(13)	C(27)-C(26)-C(25)	119.22(18)
C(8)-C(7)-O(1)	120.85(14)	C(30)-C(29)-C(14)	111.10(13)
N(2)-C(9)-C(8)	125.56(14)	C(30)-C(29)-C(36)	113.59(13)
N(2)-C(9)-O(2)	113.22(13)	C(14)-C(29)-C(36)	114.20(14)
C(8)-C(9)-O(2)	121.16(14)	C(23)-C(28)-C(27)	120.48(19)
C(9)-C(8)-C(7)	131.34(15)	C(31)-C(30)-C(35)	118.20(16)
C(12)-C(13)-C(14)	121.89(16)	C(31)-C(30)-C(29)	120.12(14)
C(2)-C(16)-C(17)	113.65(14)	C(35)-C(30)-C(29)	121.68(15)
C(2)-C(16)-C(23)	110.52(13)	C(32)-C(31)-C(30)	121.11(16)
C(17)-C(16)-C(23)	112.89(14)	C(33)-C(32)-C(31)	120.25(17)
C(11)-C(10)-O(2)	128.84(15)	C(32)-C(33)-C(34)	119.31(17)
C(11)-C(10)-C(15)	123.64(15)	C(33)-C(34)-C(35)	120.48(17)
O(2)-C(10)-C(15)	107.52(14)	C(34)-C(35)-C(30)	120.64(16)
C(13)-C(12)-C(11)	121.63(16)	C(37)-C(36)-C(41)	117.64(16)
C(10)-C(11)-C(12)	115.85(16)	C(37)-C(36)-C(29)	123.35(16)
C(13)-C(14)-C(15)	116.31(14)	C(41)-C(36)-C(29)	118.93(15)
C(13)-C(14)-C(29)	125.30(14)	C(36)-C(37)-C(38)	121.00(18)
C(15)-C(14)-C(29)	118.31(14)	C(39)-C(38)-C(37)	120.47(18)
N(2)-C(15)-C(10)	109.90(14)	C(38)-C(39)-C(40)	119.44(18)
N(2)-C(15)-C(14)	129.43(14)	C(39)-C(40)-C(41)	120.0(2)
C(10)-C(15)-C(14)	120.66(15)	C(40)-C(41)-C(36)	121.49(19)
C(22)-C(17)-C(18)	118.09(17)	O(3)-C(42)-C(47)#1	107.75(15)
C(22)-C(17)-C(16)	122.69(15)	O(3)-C(43)-C(44)	107.77(16)
C(18)-C(17)-C(16)	119.22(16)	O(4)-C(44)-C(43)	109.05(16)
C(19)-C(18)-C(17)	120.96(19)	O(4)-C(45)-C(46)	110.17(14)
C(18)-C(19)-C(20)	120.4(2)	O(5)-C(46)-C(45)	108.89(15)

O(5)-C(47)-C(42)#1	108.74(15)	O(8')-C(54')-C(53')	108.6(8)
C(48)-O(6)-C(51)	108.53(16)	C(54')-O(8')-C(55')	112.4(9)
C(48)-O(6)-K(1)	107.07(13)	C(54')-O(8')-K(2)	112.3(6)
C(51)-O(6)-K(1)	128.30(12)	C(55')-O(8')-K(2)	111.5(6)
O(6)-C(48)-C(49)	106.58(17)	O(8')-C(55')-C(56')	109.8(8)
O(6)-C(48)-K(1)	49.80(10)	O(9')-C(56')-C(55')	108.1(8)
C(49)-C(48)-K(1)	124.91(15)	C(56')-O(9')-C(57')	112.1(8)
C(48)-C(49)-C(50)	100.56(19)	C(56')-O(9')-K(2)	119.7(6)
C(49)-C(50)-C(51)	103.44(18)	C(57')-O(9')-K(2)	113.2(6)
O(6)-C(51)-C(50)	105.96(17)	O(9')-C(57')-K(2)	45.1(5)
O(7)-C(52)-C(57)#2	109.8(3)	C(52')#2-C(57')-K(2)	78.9(6)
C(53)-O(7)-C(52)	112.4(3)	C(58)-O(10)-C(61)	108.2(2)
C(53)-O(7)-K(2)	115.9(2)	C(58)-O(10)-K(2)	132.81(19)
C(52)-O(7)-K(2)	112.16(19)	C(61)-O(10)-K(2)	107.76(19)
O(7)-C(53)-C(54)	108.8(3)	O(10)-C(58)-C(59)	106.2(2)
O(8)-C(54)-C(53)	108.4(3)	C(58)-C(59)-C(60)	102.5(2)
C(55)-O(8)-C(54)	111.7(3)	C(61)-C(60)-C(59)	99.8(2)
C(55)-O(8)-K(2)	112.8(2)	O(10)-C(61)-C(60)	107.0(3)
C(54)-O(8)-K(2)	113.4(2)	O(10)-C(61)-K(2)	48.65(15)
O(8)-C(55)-C(56)	108.6(3)	C(60)-C(61)-K(2)	132.9(2)
O(9)-C(56)-C(55)	108.6(3)	C(61')-O(10')-C(58')	111.5(9)
C(56)-O(9)-C(57)	112.7(3)	C(61')-O(10')-K(2)	110.6(7)
C(56)-O(9)-K(2)	119.9(2)	C(58')-O(10')-K(2)	137.9(7)
C(57)-O(9)-K(2)	113.2(2)	O(10')-C(58')-C(59')	101.0(8)
O(9)-C(57)-C(52)#2	108.8(3)	C(60')-C(59')-C(58')	101.5(7)
C(53')-O(7')-C(52')	115.3(9)	C(61')-C(60')-C(59')	102.7(7)
C(53')-O(7')-K(2)	117.3(7)	O(10')-C(61')-C(60')	105.5(9)
C(52')-O(7')-K(2)	110.9(6)	O(10')-C(61')-K(2)	46.7(6)
O(7')-C(53')-C(54')	108.6(9)	C(60')-C(61')-K(2)	152.1(7)

Symmetry transformations used to generate equivalent atoms:

#1 -x+2,-y+1,-z+1   #2 -x,-y+1,-z+2

XRAY: Compound 12

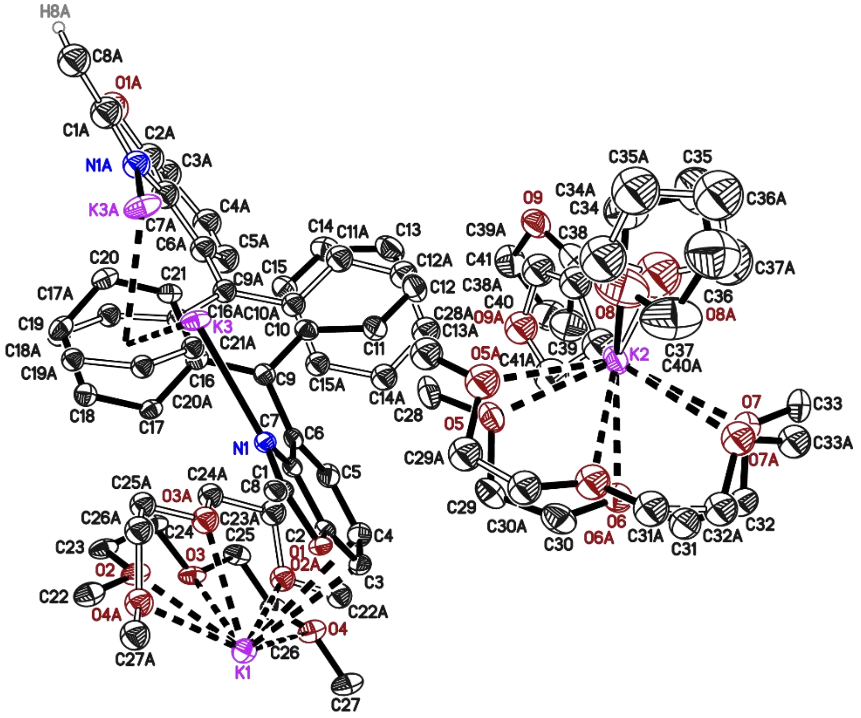

Figure S106. Asymmetric unit of 12. Displacement parameters are depicted at 50% probability. Hydrogen atoms except the hydrogen atoms bound to the methylene backbone (H8, H8A) are omitted for clarity.

The compound crystalises in space group *P/n* containing one half of a formula unit in the asymmetric unit. The whole unit except atoms K2 and K3 is disordered over two positions and was refined with distance restraints and restraints for the displacement parameter. The occupancy of the minor position refined to 0.0943(14). All atoms of this position except atom K1’ were refined isotropically.

Table S9. Bond lengths [Å] and angles [°] for **12**.

K(2)-O(2)	2.747(2)	K(3)-O(8A)	2.72(3)
K(2)-O(2A)	2.751(19)	K(3)-O(8)	2.733(4)
K(2)-O(3A)	2.80(2)	K(3)-O(6)	2.769(3)
K(2)-O(3)	2.800(2)	K(3)-O(5)	2.779(2)
K(2)-O(4)	2.834(2)	K(1)-N(1)	2.686(3)
K(2)-O(4A)	2.84(2)	K(1)-C(16)	2.930(3)
K(3)-O(6A)	2.71(3)	K(1)-C(21)	2.993(3)
K(3)-O(5A)	2.72(3)	K(1)-C(17)	3.084(3)

K(1)-C(20)	3.154(3)	C(23)-C(24)	1.495(5)
K(1)-C(18)	3.255(3)	C(25)-C(26)	1.502(5)
O(1)-C(6)	1.370(4)	O(5)-C(28)	1.417(4)
O(1)-C(7)	1.397(4)	O(5)-C(29)	1.422(5)
O(2)-C(22)	1.421(4)	O(6)-C(31)	1.411(4)
O(2)-C(23)	1.425(4)	O(6)-C(30)	1.416(5)
O(3)-C(24)	1.419(4)	O(7)-C(33)	1.418(4)
O(3)-C(25)	1.426(4)	O(7)-C(32)	1.421(4)
O(4)-C(27)	1.422(4)	C(28)-C(33)#2	1.491(5)
O(4)-C(26)	1.423(4)	C(29)-C(30)	1.482(5)
N(1)-C(7)	1.324(4)	C(31)-C(32)	1.506(6)
N(1)-C(1)	1.403(4)	O(8)-C(37)	1.381(7)
C(1)-C(6)	1.403(4)	O(8)-C(34)	1.404(6)
C(1)-C(2)	1.405(5)	C(34)-C(35)	1.508(8)
C(2)-C(3)	1.410(4)	C(35)-C(36)	1.452(8)
C(2)-C(9)	1.478(4)	C(36)-C(37)	1.520(8)
C(3)-C(4)	1.391(5)	O(9)-C(38)	1.408(6)
C(4)-C(5)	1.395(6)	O(9)-C(41)	1.428(5)
C(5)-C(6)	1.379(5)	C(38)-C(39)	1.507(6)
C(7)-C(8)	1.393(4)	C(39)-C(40)	1.470(7)
C(9)-C(16)	1.426(4)	C(40)-C(41)	1.508(6)
C(9)-C(10)	1.459(5)	K(1A)-N(1A)	2.66(2)
C(10)-C(15)	1.405(5)	K(1A)-C(16A)	2.92(3)
C(10)-C(11)	1.421(5)	K(1A)-C(21A)	3.00(3)
C(11)-C(12)	1.373(5)	K(1A)-C(17A)	3.08(3)
C(12)-C(13)	1.381(6)	K(1A)-C(20A)	3.16(3)
C(13)-C(14)	1.387(5)	K(1A)-C(18A)	3.28(3)
C(14)-C(15)	1.389(5)	O(1A)-C(6A)	1.364(17)
C(16)-C(21)	1.428(4)	O(1A)-C(7A)	1.388(18)
C(16)-C(17)	1.431(4)	O(2A)-C(22A)	1.414(14)
C(17)-C(18)	1.380(4)	O(2A)-C(23A)	1.419(14)
C(18)-C(19)	1.397(4)	O(3A)-C(24A)	1.416(14)
C(19)-C(20)	1.394(5)	O(3A)-C(25A)	1.417(14)
C(20)-C(21)	1.381(5)	O(4A)-C(27A)	1.413(14)
C(22)-C(27)#1	1.496(5)	O(4A)-C(26A)	1.425(14)

N(1A)-C(7A)	1.324(17)	C(31A)-C(32A)	1.498(15)
N(1A)-C(1A)	1.403(17)	O(8A)-C(37A)	1.405(16)
C(1A)-C(2A)	1.402(17)	O(8A)-C(34A)	1.426(16)
C(1A)-C(6A)	1.417(17)	C(34A)-C(35A)	1.503(16)
C(2A)-C(3A)	1.400(18)	C(35A)-C(36A)	1.453(18)
C(2A)-C(9A)	1.475(16)	C(36A)-C(37A)	1.510(16)
C(3A)-C(4A)	1.391(19)	O(9A)-C(41A)	1.397(15)
C(4A)-C(5A)	1.39(2)	O(9A)-C(38A)	1.402(15)
C(5A)-C(6A)	1.376(18)	C(38A)-C(39A)	1.502(16)
C(7A)-C(8A)	1.385(17)	C(39A)-C(40A)	1.453(18)
C(9A)-C(16A)	1.428(15)	C(40A)-C(41A)	1.502(16)
C(9A)-C(10A)	1.452(16)		
C(10A)-C(15A)	1.406(15)	O(2)-K(2)-O(2)#1	180.00(7)
C(10A)-C(11A)	1.417(15)	O(2A)-K(2)-O(3A)	60.3(4)
C(11A)-C(12A)	1.387(16)	O(2A)#1-K(2)-O(3A)	119.7(4)
C(12A)-C(13A)	1.382(16)	O(3A)#1-K(2)-O(3A)	180.0
C(13A)-C(14A)	1.383(16)	O(2)-K(2)-O(3)	60.63(7)
C(14A)-C(15A)	1.393(16)	O(2)#1-K(2)-O(3)	119.37(7)
C(16A)-C(21A)	1.415(14)	O(3)-K(2)-O(3)#1	180.0
C(16A)-C(17A)	1.428(15)	O(2)-K(2)-O(4)	119.93(7)
C(17A)-C(18A)	1.376(15)	O(2)#1-K(2)-O(4)	60.07(7)
C(18A)-C(19A)	1.395(15)	O(3)-K(2)-O(4)	60.52(6)
C(19A)-C(20A)	1.400(15)	O(3)#1-K(2)-O(4)	119.48(6)
C(20A)-C(21A)	1.380(15)	O(2)-K(2)-O(4)#1	60.07(7)
C(22A)-C(27A)#1	1.496(15)	O(4)-K(2)-O(4)#1	180.0
C(23A)-C(24A)	1.492(14)	O(2A)-K(2)-O(4A)	119.7(5)
C(25A)-C(26A)	1.499(15)	O(2A)#1-K(2)-O(4A)	60.3(5)
O(5A)-C(28A)	1.421(14)	O(3A)#1-K(2)-O(4A)	119.4(4)
O(5A)-C(29A)	1.423(15)	O(3A)-K(2)-O(4A)	60.6(4)
O(6A)-C(31A)	1.419(14)	O(6A)-K(3)-O(5A)	62.2(5)
O(6A)-C(30A)	1.422(14)	O(6A)#2-K(3)-O(5A)	117.8(5)
O(7A)-C(32A)	1.411(15)	O(5A)#2-K(3)-O(5A)	180.0
O(7A)-C(33A)	1.428(14)	O(6A)-K(3)-O(8A)#2	84.0(11)
C(28A)-C(33A)#2	1.492(15)	O(5A)-K(3)-O(8A)#2	86.9(10)
C(29A)-C(30A)	1.479(15)	O(6A)-K(3)-O(8A)	96.0(11)

O(5A)-K(3)-O(8A)	93.1(10)	C(21)-K(1)-C(20)	25.80(8)
O(8A)#2-K(3)-O(8A)	180.0	C(17)-K(1)-C(20)	52.58(8)
O(8)#2-K(3)-O(8)	180.0	C(17)#3-K(1)-C(20)	118.98(8)
O(8)#2-K(3)-O(6)	83.30(10)	N(1)-K(1)-C(20)#3	149.68(8)
O(8)-K(3)-O(6)	96.70(10)	N(1)#3-K(1)-C(20)#3	117.53(8)
O(6)-K(3)-O(6)#2	180.00(11)	C(16)-K(1)-C(20)#3	99.12(9)
O(8)#2-K(3)-O(5)	97.69(11)	C(21)-K(1)-C(20)#3	73.31(9)
O(8)-K(3)-O(5)	82.31(11)	C(17)-K(1)-C(20)#3	118.98(8)
O(6)-K(3)-O(5)	60.25(8)	C(20)-K(1)-C(20)#3	69.17(12)
O(6)#2-K(3)-O(5)	119.76(8)	N(1)-K(1)-C(18)#3	116.26(8)
O(5)-K(3)-O(5)#2	180.0	C(16)-K(1)-C(18)#3	123.65(9)
N(1)-K(1)-N(1)#3	72.78(11)	C(21)-K(1)-C(18)#3	107.74(9)
N(1)-K(1)-C(16)	72.91(8)	C(17)-K(1)-C(18)#3	151.03(8)
N(1)#3-K(1)-C(16)	143.02(8)	C(20)-K(1)-C(18)#3	111.92(9)
C(16)-K(1)-C(16)#3	143.45(12)	N(1)-K(1)-C(18)	84.26(8)
N(1)-K(1)-C(21)#3	167.98(8)	N(1)#3-K(1)-C(18)	116.26(8)
C(16)-K(1)-C(21)#3	115.57(9)	C(16)-K(1)-C(18)	46.56(8)
N(1)-K(1)-C(21)	100.55(8)	C(16)#3-K(1)-C(18)	123.65(8)
C(16)-K(1)-C(21)	27.88(8)	C(21)#3-K(1)-C(18)	107.74(9)
C(21)#3-K(1)-C(21)	87.69(13)	C(21)-K(1)-C(18)	52.30(8)
N(1)-K(1)-C(17)	66.12(8)	C(17)-K(1)-C(18)	24.95(8)
N(1)#3-K(1)-C(17)	121.68(8)	C(17)#3-K(1)-C(18)	151.03(8)
C(16)-K(1)-C(17)	27.39(8)	C(20)-K(1)-C(18)	43.66(8)
C(16)#3-K(1)-C(17)	147.25(8)	C(20)#3-K(1)-C(18)	111.92(9)
C(21)#3-K(1)-C(17)	125.42(9)	C(18)#3-K(1)-C(18)	155.44(12)
C(21)-K(1)-C(17)	46.57(8)	C(6)-O(1)-C(7)	104.9(2)
N(1)-K(1)-C(17)#3	121.68(8)	C(22)-O(2)-C(23)	112.9(3)
C(16)-K(1)-C(17)#3	147.25(8)	C(22)-O(2)-K(2)	117.42(18)
C(16)#3-K(1)-C(17)#3	27.39(8)	C(23)-O(2)-K(2)	117.10(18)
C(17)-K(1)-C(17)#3	171.42(12)	C(24)-O(3)-C(25)	111.4(2)
N(1)-K(1)-C(20)	117.54(8)	C(24)-O(3)-K(2)	113.75(17)
N(1)#3-K(1)-C(20)	149.68(8)	C(25)-O(3)-K(2)	114.21(17)
C(16)-K(1)-C(20)	47.55(8)	C(27)-O(4)-C(26)	112.3(2)
C(16)#3-K(1)-C(20)	99.12(9)	C(27)-O(4)-K(2)	112.8(2)
C(21)#3-K(1)-C(20)	73.31(9)	C(26)-O(4)-K(2)	113.63(19)

C(7)-N(1)-C(1)	105.6(3)	C(9)-C(16)-C(17)	121.0(3)
C(7)-N(1)-K(1)	128.3(2)	C(21)-C(16)-C(17)	114.4(3)
C(1)-N(1)-K(1)	126.03(19)	C(9)-C(16)-K(1)	104.53(19)
C(6)-C(1)-N(1)	108.0(3)	C(21)-C(16)-K(1)	78.53(18)
C(6)-C(1)-C(2)	120.8(3)	C(17)-C(16)-K(1)	82.30(18)
N(1)-C(1)-C(2)	131.1(3)	C(18)-C(17)-C(16)	122.3(3)
C(1)-C(2)-C(3)	114.2(3)	C(18)-C(17)-K(1)	84.48(18)
C(1)-C(2)-C(9)	125.0(3)	C(16)-C(17)-K(1)	70.31(16)
C(3)-C(2)-C(9)	120.7(3)	C(18)-C(17)-H(17)	118.8
C(4)-C(3)-C(2)	124.1(3)	C(16)-C(17)-H(17)	118.8
C(3)-C(4)-C(5)	121.3(3)	K(1)-C(17)-H(17)	116.7
C(3)-C(4)-K(2)	106.8(2)	C(17)-C(18)-C(19)	121.6(3)
C(5)-C(4)-K(2)	74.4(4)	C(17)-C(18)-K(1)	70.56(17)
C(6)-C(5)-C(4)	115.1(4)	C(19)-C(18)-K(1)	79.12(19)
C(6)-C(5)-K(2)	102.7(4)	C(20)-C(19)-C(18)	117.4(3)
C(4)-C(5)-K(2)	81.1(3)	C(20)-C(19)-K(1)	72.06(18)
O(1)-C(6)-C(5)	126.9(3)	C(18)-C(19)-K(1)	76.25(19)
O(1)-C(6)-C(1)	108.4(3)	C(21)-C(20)-C(19)	121.7(3)
C(5)-C(6)-C(1)	124.6(4)	C(21)-C(20)-K(1)	70.59(17)
N(1)-C(7)-C(8)	132.0(3)	C(19)-C(20)-K(1)	83.07(19)
N(1)-C(7)-O(1)	113.0(3)	C(20)-C(21)-C(16)	122.3(3)
C(8)-C(7)-O(1)	114.9(3)	C(20)-C(21)-K(1)	83.61(19)
C(7)-C(8)-C(7)#3	126.6(4)	C(16)-C(21)-K(1)	73.59(17)
C(16)-C(9)-C(10)	122.6(3)	O(2)-C(22)-C(27)#1	108.6(3)
C(16)-C(9)-C(2)	120.5(3)	O(2)-C(23)-C(24)	109.1(3)
C(10)-C(9)-C(2)	116.9(3)	O(3)-C(24)-C(23)	109.2(3)
C(15)-C(10)-C(11)	115.7(3)	O(3)-C(25)-C(26)	108.8(3)
C(15)-C(10)-C(9)	124.2(3)	O(4)-C(26)-C(25)	108.6(3)
C(11)-C(10)-C(9)	120.2(3)	O(4)-C(27)-C(22)#1	107.8(3)
C(12)-C(11)-C(10)	122.4(4)	C(28)-O(5)-C(29)	110.9(3)
C(11)-C(12)-C(13)	120.5(4)	C(28)-O(5)-K(3)	114.9(2)
C(12)-C(13)-C(14)	119.0(4)	C(29)-O(5)-K(3)	115.4(2)
C(13)-C(14)-C(15)	120.9(4)	C(31)-O(6)-C(30)	111.4(3)
C(14)-C(15)-C(10)	121.6(3)	C(31)-O(6)-K(3)	116.2(2)
C(9)-C(16)-C(21)	124.5(3)	C(30)-O(6)-K(3)	116.1(2)

C(33)-O(7)-C(32)	110.8(3)	C(17A)#3-K(1A)-C(17A)	171.1(9)
C(33)-O(7)-K(3)	114.3(2)	N(1A)-K(1A)-C(20A)	118.1(5)
C(32)-O(7)-K(3)	114.1(2)	N(1A)#3-K(1A)-C(20A)	147.9(6)
O(5)-C(28)-C(33)#2	109.5(3)	C(16A)-K(1A)-C(20A)	47.4(4)
O(5)-C(29)-C(30)	109.1(3)	C(16A)#3-K(1A)-C(20A)	98.2(6)
O(6)-C(30)-C(29)	109.4(3)	C(21A)-K(1A)-C(20A)	25.7(3)
O(6)-C(31)-C(32)	109.2(3)	C(21A)#3-K(1A)-C(20A)	72.9(7)
O(7)-C(32)-C(31)	108.7(3)	C(17A)#3-K(1A)-C(20A)	118.8(7)
O(7)-C(33)-C(28)#2	109.6(3)	C(17A)-K(1A)-C(20A)	52.6(5)
C(37)-O(8)-C(34)	110.1(4)	N(1A)-K(1A)-C(18A)	84.4(6)
C(37)-O(8)-K(3)	113.2(3)	N(1A)#3-K(1A)-C(18A)	115.2(7)
C(34)-O(8)-K(3)	122.5(3)	C(16A)-K(1A)-C(18A)	46.3(4)
O(8)-C(34)-C(35)	104.9(5)	C(16A)#3-K(1A)-C(18A)	123.9(5)
C(36)-C(35)-C(34)	104.6(5)	C(21A)-K(1A)-C(18A)	52.1(5)
C(35)-C(36)-C(37)	102.1(5)	C(21A)#3-K(1A)-C(18A)	108.4(7)
O(8)-C(37)-C(36)	108.2(5)	C(17A)#3-K(1A)-C(18A)	151.2(5)
O(8)-C(37)-K(3)	45.6(3)	C(17A)-K(1A)-C(18A)	24.7(3)
C(36)-C(37)-K(3)	139.1(4)	C(20A)-K(1A)-C(18A)	43.7(5)
C(38)-O(9)-C(41)	108.5(3)	C(20A)#3-K(1A)-C(18A)	112.7(7)
O(9)-C(38)-C(39)	107.5(4)	C(6A)-O(1A)-C(7A)	104.5(16)
C(40)-C(39)-C(38)	102.2(4)	C(22A)-O(2A)-C(23A)	113.2(15)
C(39)-C(40)-C(41)	102.3(4)	C(22A)-O(2A)-K(2)	116.6(16)
O(9)-C(41)-C(40)	104.9(4)	C(23A)-O(2A)-K(2)	117.9(12)
N(1A)-K(1A)-C(16A)	73.7(5)	C(24A)-O(3A)-C(25A)	111.0(15)
N(1A)#3-K(1A)-C(16A)	143.6(6)	C(24A)-O(3A)-K(2)	115.5(13)
N(1A)-K(1A)-C(21A)	101.1(5)	C(25A)-O(3A)-K(2)	114.5(14)
N(1A)#3-K(1A)-C(21A)	167.1(7)	C(27A)-O(4A)-C(26A)	113.1(16)
C(16A)-K(1A)-C(21A)	27.6(3)	C(27A)-O(4A)-K(2)	113.1(18)
C(16A)#3-K(1A)-C(21A)	114.3(7)	C(26A)-O(4A)-K(2)	112.4(13)
N(1A)-K(1A)-C(17A)	66.6(5)	C(7A)-N(1A)-C(1A)	105.5(15)
N(1A)#3-K(1A)-C(17A)	121.4(7)	C(7A)-N(1A)-K(1A)	128.5(13)
C(16A)-K(1A)-C(17A)	27.4(3)	C(1A)-N(1A)-K(1A)	126.0(11)
C(16A)#3-K(1A)-C(17A)	146.9(6)	C(2A)-C(1A)-N(1A)	130.5(17)
C(21A)-K(1A)-C(17A)	46.4(5)	C(2A)-C(1A)-C(6A)	122.3(16)
C(21A)#3-K(1A)-C(17A)	125.2(7)	N(1A)-C(1A)-C(6A)	107.2(14)

C(3A)-C(2A)-C(1A)	113.2(16)	C(17A)-C(18A)-K(1A)	69.4(14)
C(3A)-C(2A)-C(9A)	121.6(17)	C(19A)-C(18A)-K(1A)	78.6(16)
C(1A)-C(2A)-C(9A)	124.9(17)	C(18A)-C(19A)-C(20A)	118.3(15)
C(4A)-C(3A)-C(2A)	123(2)	C(18A)-C(19A)-K(1A)	77.0(16)
C(3A)-C(4A)-C(5A)	123(2)	C(20A)-C(19A)-K(1A)	71.9(15)
C(6A)-C(5A)-C(4A)	114(2)	C(21A)-C(20A)-C(19A)	120.8(14)
O(1A)-C(6A)-C(5A)	128(2)	C(21A)-C(20A)-K(1A)	70.5(15)
O(1A)-C(6A)-C(1A)	108.9(15)	C(19A)-C(20A)-K(1A)	83.2(16)
C(5A)-C(6A)-C(1A)	123(2)	C(20A)-C(21A)-C(16A)	122.5(14)
N(1A)-C(7A)-C(8A)	130(2)	C(20A)-C(21A)-K(1A)	83.7(16)
N(1A)-C(7A)-O(1A)	114.0(16)	C(16A)-C(21A)-K(1A)	73.2(16)
C(8A)-C(7A)-O(1A)	115.6(19)	O(2A)-C(23A)-C(24A)	111.2(16)
C(16A)-C(9A)-C(10A)	123.3(15)	O(3A)-C(24A)-C(23A)	109.6(16)
C(16A)-C(9A)-C(2A)	120.0(15)	O(3A)-C(25A)-C(26A)	108.9(16)
C(10A)-C(9A)-C(2A)	116.6(16)	O(4A)-C(26A)-C(25A)	108.7(16)
C(16A)-C(9A)-K(1A)	54.0(15)	C(28A)-O(5A)-C(29A)	108.9(17)
C(10A)-C(9A)-K(1A)	121(2)	C(28A)-O(5A)-K(3)	118(2)
C(2A)-C(9A)-K(1A)	98.9(11)	C(29A)-O(5A)-K(3)	115.4(16)
C(15A)-C(10A)-C(11A)	116.4(17)	C(31A)-O(6A)-C(30A)	110.0(17)
C(15A)-C(10A)-C(9A)	121.8(16)	C(31A)-O(6A)-K(3)	116.7(17)
C(11A)-C(10A)-C(9A)	121.7(16)	C(30A)-O(6A)-K(3)	116.3(16)
C(12A)-C(11A)-C(10A)	120.9(18)	C(32A)-O(7A)-C(33A)	110.4(18)
C(12A)-C(13A)-C(14A)	121(2)	C(32A)-O(7A)-K(3)	112.8(15)
C(13A)-C(14A)-C(15A)	118.4(18)	C(33A)-O(7A)-K(3)	114(2)
C(14A)-C(15A)-C(10A)	122.5(17)	O(5A)-C(29A)-C(30A)	110.6(18)
C(21A)-C(16A)-C(9A)	124.3(14)	O(6A)-C(30A)-C(29A)	112.5(18)
C(21A)-C(16A)-C(17A)	114.8(14)	O(6A)-C(31A)-C(32A)	110.5(18)
C(9A)-C(16A)-C(17A)	120.7(13)	O(7A)-C(32A)-C(31A)	110.7(18)
C(21A)-C(16A)-K(1A)	79.2(17)	C(37A)-O(8A)-C(34A)	107(2)
C(9A)-C(16A)-K(1A)	102.7(17)	C(37A)-O(8A)-K(3)	132(2)
C(17A)-C(16A)-K(1A)	82.5(16)	C(34A)-O(8A)-K(3)	116(2)
C(18A)-C(17A)-C(16A)	122.6(14)	O(8A)-C(34A)-C(35A)	103.4(17)
C(18A)-C(17A)-K(1A)	85.9(16)	C(36A)-C(35A)-C(34A)	106.2(15)
C(16A)-C(17A)-K(1A)	70.1(15)	C(35A)-C(36A)-C(37A)	105.4(13)
C(17A)-C(18A)-C(19A)	120.7(15)	O(8A)-C(37A)-C(36A)	105.3(17)

C(41A)-O(9A)-C(38A)	112.9(16)
O(9A)-C(38A)-C(39A)	104.2(15)
C(40A)-C(39A)-C(38A)	104.9(15)
C(39A)-C(40A)-C(41A)	104.5(15)
O(9A)-C(41A)-C(40A)	105.1(15)

____

Symmetry transformations used to generate equivalent atoms:
#1 -x+1,-y+1,-z   #2 -x+1,-y+1,-z+1   #3 -x+3/2,y,-z+1/2
#4 x,y-1,z

References

[1] R. Breslow, W. Chu, *J. Am. Chem. Soc.* **1970**, *92*, 2165.

[2] a) A. Kütt, I. Leito, I. Kaljurand, L. Sooväli, V. M. Vlasov, L. M. Yagupolskii, I. A. Koppel, *J. Org. Chem.* **2006**, *71*, 2829–2838; b) I. Leito, T. Rodima, I. A. Koppel, R. Schwesinger, V. M. Vlasov, *The Journal of organic chemistry* **1997**, *62*, 8479–8483.

[3] J. Kretsch, I. Koehne, M. Lõkov, I. Leito, D. Stalke, *Eur. J. Inorg. Chem.* **2019**, *2019*, 3258–3264.

[4] a) F. Neese, *Wiley Interdiscip. Rev.-Comput. Mol. Sci.* **2012**, *2*, 73–78; b) F. Neese, *Wiley Interdiscip. Rev.-Comput. Mol. Sci.* **2017**, *8*.

[5] S. Grimme, S. Ehrlich, L. Goerigk, *J. Comput. Chem.* **2011**, *32*, 1456–1465.

[6] a) A. D. Becke, *Phys. Rev. A* **1988**, *38*, 3098–3100; b) J. P. Perdew, *Phys. Rev. B* **1986**, 8822; c) A. Schäfer, C. Huber, R. Ahlrichs, *J. Chem. Phys.* **1994**, *100*, 5829–5835; d) F. Weigend, R. Ahlrichs, *Phys. Chem. Chem. Phys.* **2005**, *7*, 3297–3305; e) K. Eichkorn, F. Weigend, O. Treutler, R. Ahlrichs, *Theor. Chem. Acc.* **1997**, *97*, 119–124.

[7] A. V. Marenich, C. J. Cramer, D. G. Truhlar, *J. Phys. Chem. B* **2009**, *113*, 6378–6396.

[8] Marcus D Hanwell, Donald E Curtis, David C Lonie, Tim Vandermeersch, Eva Zurek, Geoffrey R Hutchison, *J. Cheminformatics* **2012**, *4*, 1–17.

[9] A. K. Rappe, C. J. Casewit, K. S. Colwell, W. A. Goddard, W. M. Skiff, *J. Am. Chem. Soc.* **1992**, *114*, 10024–10035.

[10] G. Knizia, *J. Chem. Theory Comput.* **2013**, *9*, 4834–4843.
